# Endogenous Alpha-Synuclein is Essential for the Transfer of Pathology by Exosome-Enriched Extracellular Vesicles, Following Inoculation with Preformed Fibrils *in vivo*

**DOI:** 10.14336/AD.2023.0614

**Published:** 2024-04-01

**Authors:** Katerina Melachroinou, Georgios Divolis, George Tsafaras, Mantia Karampetsou, Sotirios Fortis, Yannis Stratoulias, Gina Papadopoulou, Anastasios G Kriebardis, Martina Samiotaki, Kostas Vekrellis

**Affiliations:** ^1^Center of Basic Research, Biomedical Research Foundation of the Academy of Athens, Athens, Greece.; ^2^Center for Clinical, Experimental Surgery and Translational Research, Biomedical Research Foundation of the Academy of Athens, Athens, Greece.; ^3^Laboratory of Reliability and Quality Control in Laboratory Hematology (HemQcR), Department of Biomedical Sciences, School of Health & Welfare Sciences, University of West Attica (UniWA), Egaleo, Greece.; ^4^Cancer Immunology and Immunotherapy Center, Cancer Research Center, Saint Savas Cancer Hospital, 11522 Athens, Greece.; ^5^Biomedical Sciences Research Center "Alexander Fleming", Vari, Greece.

**Keywords:** Alpha-Synuclein (α-Syn), exosomes, extracellular vesicles, Parkinson’s disease, pathology transmission, spreading, α-Syn preformed fibrils (PFF)

## Abstract

The main pathological hallmark of Parkinson’s disease (PD) and related synucleinopathies is the presence of intracellular proteinaceous aggregates, enriched in the presynaptic protein alpha-Synuclein (α-Syn). α-Syn association with exosomes has been previously documented both as a physiological process of secretion and as a pathological process of disease transmission, however, critical information about the mechanisms governing this interplay is still lacking. To address this, we utilized the α-Syn preformed fibril (PFF) mouse model of PD, as a source of brain-derived exosome-enriched extracellular vesicles (ExE-EVs) and assessed their pathogenic capacity following intrastriatal injections in host wild type (WT) mouse brain. We further investigated the impact of the fibrillar α-Syn on the exosomal cargo independent of the endogenous α-Syn, by isolating ExE-EVs from PFF-injected α-Syn knockout mice. Although PFF inoculation does not alter the morphology, size distribution, and quantity of brain-derived ExE-EVs, it triggers changes in the exosomal proteome related to synaptic and mitochondrial function, as well as metabolic processes. Importantly, we showed that the presence of the endogenous α-Syn is essential for the ExE-EVs to acquire a pathogenic capacity, allowing them to mediate disease transmission by inducing phosphorylated-α-Syn pathology. Notably, misfolded α-Syn containing ExE-EVs when injected in WT mice were able to induce astrogliosis and synaptic alterations in the host brain, at very early stages of α-Syn pathology, preceding the formation of the insoluble α-Syn accumulations. Collectively, our data suggest that exosomal cargo defines their ability to spread α-Syn pathology.

## INTRODUCTION

Abnormal α-synuclein (α-Syn) deposition and spreading comprise a common denominator in a wide range of neurodegenerative disorders, collectively termed synucleinopathies, including Parkinson’s disease (PD), multiple system atrophy (MSA), and dementia with Lewy bodies (DLB) [1, 2]. However, up to date, the processes that drive the spread of α-Syn pathology have not been deciphered.

α-Syn secretion in the extracellular milieu has been extensively studied, not only as a physiological paracrine process but also as a means of disease transmission under pathological conditions [3,7]. Importantly, we and others have shown that α-Syn is associated with a specific type of nano-sized intraluminal membrane vesicles (50-100 nm), termed exosomes [6]-[8]; these originate from the inward budding of multi-vesicular bodies and are released to the extracellular space upon their fusion with the plasma membrane in an exocytic manner [9]. They are secreted from a wide range of cell types, including neurons and glial cells [10, 11], therefore their biochemical profile reflects the tissue of origin [12], [13]. Importantly, once released, exosomes can fuse with membranes of neighboring cells, delivering cargo proteins, mRNAs, and microRNAs from one cell to another, thus facilitating intercellular communication [14, 15].

α-Syn aggregation has been shown to impair intracellular proteostatic mechanisms [16] and enhance its secretion via exosomes [17], supplying the extracellular space with potential pathogenic cargo. α-Syn spreading capacity coupled with its association with exosomes has shifted the interest towards the latter, as potential “Trojan horses” for pathology propagation. Indeed, exosomes per se can provide a milieu for a local α-Syn concentration gradient, protected from degradation [6], due to their unique biochemical features. There is a large body of evidence stemming from both *in vitro* and *in vivo* studies, showing that exosome-associated α-Syn can be internalized by neuronal [18, 19] and glial cells [20] and is sufficient to propagate pathology [17, 21, 23]. More specifically, intravenous administration or stereotactic injection of exosomes containing aberrant forms of α-Syn, isolated from either brain tissue [22], serum [21], or blood plasma [20] from synucleinopathy patients (PD, DLB, or both), was able to induce intracellular protein accumulation, characterized by the presence of phos-phorylated proteins, such as p-α-Syn, pTau, p62, and ubiquitin, as well as microglia activation [20, 23]. Consistently, intranasal administration of extracellular vesicles isolated from cerebrospinal fluid (CSF) of PD patients triggered both behavioural deficits and histopathological alterations, with phosphorylated-α-Syn (phospho-α-Syn) positive accumulations along the nigrostriatal axis, of wild type mice [24]. On the other hand, we have shown that intrastriatal inoculation of wild-type (WT) mice with exosomes isolated from symptomatic A53T transgenic mice, failed to provoke endogenous α-Syn misfolding and accumulation in host mice [19]. Nonetheless, whether the role of exosomes on disease transmission and progression is fundamental or complementary remains to be clarified.

In the current study, we have used the well-established recombinant α-Syn preformed fibril (PFF) mouse model, as a source of brain-derived exosome-enriched extracellular vesicles (ExE-EVs). This model comprises a unique *in vivo* tool, based on the seeding capacity of PFFs, which when intracerebrally injected are able to convert normal endogenous α-Syn into insoluble neuronal aggregates throughout interconnected brain areas [25], mimicking the major histo-pathological hallmark of PD. In addition, protein accumulation is accompanied by neuroinflammation and at late time-points neurodegeneration and motor deficits [26]. In this respect, we sought to investigate, for the first time, how PFFs, per se in this model, alter the protein cargo of ExE-EVs, in the presence or absence of the endogenous α-Syn. Importantly, we explored whether endogenous α-Syn was critical in converting ExE-EVs to pathogenic vehicles. Hence, we isolated interstitial brain ExE-EVs from WT and α-Syn knockout (KO) mice post-PFF intrastriatal injections, following an optimized multistep protocol, previously described by our group and others [19, 27, 28] and assessed their seeding capacity *in vivo* in WT host mice.

Overall, our study shows, for the first time, that α-Syn PFFs cause significant alterations of the proteomic exosome cargo *in vivo*, in both WT and α-Syn KO backgrounds. However, the presence of endogenous α-Syn is necessary in order for these changes to confer the pathogenic capacity to ExE-EVs and facilitate the propagation of pathology, thus supporting the role of exosomes/ ExE-EVs in a prion-like mode of infection.

## MATERIALS AND METHODS

### Animals and ethical approval

For this study 5-6-month-old wild-type (WT) C57BL6/C3H (Jackson Laboratory, Bar Harbor, Main) and α-Syn knockout mice (α-Syn KO) (C57BL6/JOlaHsd mice, Harlan Laboratories) were used. Animals were housed in individually ventilated cages with free access to food and water, under a controlled light-dark cycle (12 h light-12 h dark) and temperature (21+1 ^o^C), at the animal facility of the Biomedical Research Foundation of the Academy of Athens. All experimental protocols were performed in accordance with the EU directive guidelines for the care and use of experimental animals (86/609/EEC; 27/01/1992, No. 116) and were approved by the Institutional Ethics Committee for Use of Laboratory Animals, and an authorized veterinarian committee in accordance to Greek legislation (Presidential Decree 56/2013, in compliance with the European Directive 2010/63).

### Stereotaxic Injections

Unilateral striatal injections were performed under general isoflurane anesthesia by an apparatus adjusted to the stereotaxic frame (Kopf Instruments, United States). The right dorsal *striatum* was targeted using the following coordinates from bregma: anteroposterior of +0.5 mm, mediolateral of +2.0 mm, and dorsoventral in two depths of 3.2 and 3.4 mm, according to the mouse stereotaxic atlas (“The Mouse Brain in Stereotaxic Coordinates”, G.Paxinos & K.B.J. Franklin) [29]. The injection material was administered at a constant flow rate of 0.27 μl/min, whereas a 5-min interval was followed between target depths and the needle was slowly retracted 5 min after the injection procedure was completed. For the exosome-erniched extracellular vesicle isolation (ExE-EV) isolation, 5-6-month-old WT C57BI6/C3H and α-Syn KO mice C57BL6/JOlaHsd were injected with 2.6 μg of mouse PFFs diluted to a final volume of 2 μl or 2 μl of phosphate-buffered saline (PBS), (3 mice/WT group, 3 mice/KO group). ExE-EV-donor mice were sacrificed at one-month post-injection. For the immunohistochemical analysis of the ExE-EV-donor mice, the same experimental scheme was followed for 3 mice/group. For all the exosome inoculation experiments, 5-6 months old WT C57BL6/C3H mice were intrastriatally injected with 5.5 μg of the exosome-enriched fraction C (*Whole-Brain Exosome-enriched Extracellular Vesicle Isolation, Purification and labeling*) isolated from PFF- or PBS-injected WT or α-Syn KO mice, as previously described [19, 22, 30, 31]. The host mice were sacrificed at two-months post-injection.

### Whole-Brain Exosome-enriched extracellular vesicle Isolation, Purification and Labelling

WT C57BI6/C3H and α-Syn KO mice C57BL6/JOlaHsd mice were inoculated with mouse PFFs or PBS (control group) in the right dorsal *striatum*, as described in the previous section. One-month post-injection, mice were sacrificed, brains were excised and ExE-EVs were isolated from the injected hemispheres as previously described [28], with slight modifications [19, 27]. The injected hemi-brains were enzymatically dissociated with papain (20 units/ml, Worthington) diluted in Hibernate A solution (5 ml/brain; BrainBits) at 37^o^C for 15 min. Papain activity was halted by the addition of two volumes of cold Hibernate A solution and brain tissue was homogenized by 10 strokes in a glass Teflon homogenizer. The suspension was filtered through a 40-μm cell strainer and centrifuged at 300 x *g* (20 min, 4^o^C). The supernatant was sequentially filtered through a 0.45-μm and 0.2-μm syringe filter and further centrifuged at 2,000 x *g* (10 min, 4^o^C), 10,000 x *g* (30 min, 4^o^C) and finally at 100,000 x *g* (70 min, 4^o^C). The resulting crude exosome-enriched pellet was rinsed in 22-24 ml of cold PBS and spun down at 100,000 x *g* (70 min, 4^o^C). The exosome-enriched pellet was further purified, upon dilution in 1.5 ml of 0.95 M sucrose solution, loading on a sucrose gradient column comprised of six fractions (2.0 M, 1.65 M, 1.3 M, 0.95 M, 0.60 M, 0.25 M, 1.5 ml each), and centrifugation at 200,000 x *g* (16 h, 4^o^C). Seven fractions were separated according to the gradient (a-g from top to bottom). Fractions B, C and D were collected individually, diluted in PBS to 22 ml final volume and centrifuged at 100,000 x *g* (70 min, 4^o^C). The resulting pellets were re-suspended in PBS and stored at -80^o^C until used. For the labelling of the lipid membrane of ExE-EVs the lipophilic dye 1,1'-Dioctadecyl-3,3,3',3'-Tetramethy-lindocarbocyanine Perchlorate ('DiI'; Invitrogen) was used. Briefly, ExE-EVs were diluted in 1ml DiI solution (2 μM DiI in PBS) and incubated at 37°C for 5 min. Following incubation at 4°C for 15 min, 2 volumes of PBS were added to the mix, which was spun at 100,000xg for 70 min at 4°C. An additional PBS wash for 70 min 100,000xg was performed to remove the residual dye. The final pellet was reconstituted in PBS and stored at -80^o^C.

### Electron Microscopy

Exosome-enriched fractions B, C, and D were fixed with 4% paraformaldehyde overnight at 4^o^C. Five microliters of the preparations were loaded on 300-mesh copper grids with carbon-coated formvar film and incubated for 20 min. Following a rinse in PBS, the grids were incubated with 1% glutaraldehyde for 5 min, stained with uranyl oxalate (pH 7, 5 min) and methyl cellulose-uranyl acetate (10 min on ice) and allowed to dry. For the assessment of the size and morphology of PFFs by EM, 5 μl of PFFs were loaded on Formvar-coated 400 mesh copper grids and following brief fixation with 0.5% glutaraldehyde (5μl), they were negatively stained with 2% uranyl acetate (Sigma-Aldrich, USA). Samples were examined with a Philips 420 Transmission Electron Microscope at an acceleration voltage of 60 kV and photographed with a Megaview G2CCD camera (Olympus SIS, Münster, Germany) and iTEM image capture software. Size distribution of exosomes was evaluated using image Fiji v2.0.0 software.

### Acetylcholinesterase activity assay

Acetylcholinesterase (AChE) activity was measured in the exosome-enriched fractions (B, C, and D), as described by Savina et al. [32]. Briefly, 10 μl of each fraction or known concentrations of recombinant AChE were added to individual wells on a 96-well flat-bottomed microplate; 1.25 mM of acetylthiocholine and 0.1 mM of 5,50-dithiobis (2-nitrobenzoic acid) in PBS were added to a final volume of 250 μl. Following 30-min incubation at RT, under agitation, in the dark, the absorbance at 405 nm was measured. The ratio of the ng AChE/μl of each fraction normalized to the respective protein load (in μg/μl), as measured by Bradford protein assay, is indicative of the enrichment in exosomes.

### Nanoparticle Tracking Analysis of exosomal preparations

NanoSight NS300 instrument (Malvern Instruments, Amesbury, UK), equipped with a 532 nm laser (green), a high sensitivity sCMOS camera and a syringe pump, was used for Nanoparticle tracking analysis (NTA). The ExE-EV preparations were diluted in particle-free PBS (0.22 µm filtered) to obtain a concentration within the recommended measurement range (1-10 × 10^8^ particles/ mL), corresponding to approximately 0.8-1.0 μg of total protein. Each of the diluted samples was loaded on a 1 ml syringe, which was then placed to the pump. Autofocus was adjusted so that blurry particles were avoided. For each measurement, 5 videos with a duration of 30 seconds each, were captured under the following stable conditions: cell temperature: 25°C; Syringe speed: 100 µl/s. NanoSight NTA 3.4 build 3.4.4 software (Copyright 2020, Malvern) was used for the analysis of the acquired videos, following capture in script control mode. A total of 1500 frames were examined per sample.

### Western Immunoblotting and Dot blot analysis

Fifty μg of sucrose gradient purified fractions (B, C, and D), diluted in PBS, were sonicated for 5 min in a water-bath sonicator, and run on a 13% SDS-PAGE Tris-glycine gel. Proteins were subsequently transferred onto nitrocellulose membranes (Amersham, 106000001) and analyzed by immunoblotting. For dot blot analysis, 20 μg of pre-sonicated fractions C were loaded on a multi-well device, and proteins were bound to a nitrocellulose membrane (Amersham, 10600001), under vacuum for 30 min at RT. Membranes were blocked in blocking buffer (5 % non-fat milk, 0.05% Tween-20 in TBS), for 1 h at RT and probed overnight at 4^o^C with the following primary antibodies: anti-α-Syn C20 (Santa Cruz, 1:1000), anti-α-syn Syn1 (BD biosciences, 1:1,000), anti-pS129 α-Syn (Abcam, 1:1,000), anti-Flotillin-1 (Santa Cruz, 1:1,000), and recombinant anti-α-Syn aggregate antibody (conformation-specific MJFR-14-6-4-2, Abcam, 1:50,000). Following 10 min washes with 0.05% Tween-20 in TBS (TBS-T) thrice, secondary antibodies (Merck-Millipore) diluted in blocking buffer were added for 2 h at RT. Membranes were rinsed again as above and Clarity Western ECL Substrate (Bio-Rad) was used for the detection of the proteins bound to nitrocellulose membranes (Supplementary Table 6).

### Immunohistochemistry

Mice were anesthetized with isoflurane anesthesia and intracardially perfused with ice-cold PBS (30 ml/mouse), followed by 4% paraformaldehyde (30 ml/mouse) in PBS, under a constant flow rate using a pump. Brains were post-fixed overnight at 4^o^C, gradually dehydrated by sequential incubation in 15% and 30% sucrose in PBS at 4^o^C, snap-frozen by immersion into frozen iso-pentane (-45 ^o^C) for 30 sec and stored at -80 °C. Free-floating cryo-sections of 30 μm were collected, using a Leica CM3050S cryostat at -25 °C. For immunohistochemistry, following treatment with antigen retrieval solution (citrate buffer, pH = 6) at 80 °C for 20 min, 3 x 10 min PBS washes and a 60 min blocking in 5% normal goat serum (NGS) in PBS containing 0.1% Triton X (blocking buffer), sections were incubated with the primary antibodies in blocking buffer for 24 h at 4 °C. Following 3 x 10 min PBS washes, secondary antibodies (Invitrogen) diluted in blocking buffer were added for 2 h at RT. The sections were rinsed again as above and mounted on Poly-D-lysine slides (VWR) using Vectashield vibrance anti-fade mounting medium (Vector-labs). For the conformation specific antibody MJFR-14-6-4-2, following incubation with the citrate buffer, sections were treated with Proteinase K (PK) (Sigma-Aldrich, USA) at a final concentration of 5 μg/ml in PBS for 10 min at RT to expose antigenic sites [33].The following primary antibodies were used: tyrosine hydroxylase (TH) (rabbit polyclonal, 1:2,000, Merck-Millipore; mouse monoclonal, 1:2,000, Merck-Millipore), phospho-S129 α-Syn (rabbit monoclonal, 1:2,000, Abcam; mouse monoclonal, gift from Omar El Agnaf, 1:1,000, MJFR-14-6-4-2 (rabbit monoclonal, abcam, 1:2,000), GFAP (rabbit polyclonal, DAKO, 1:750), Iba-1 (mouse monoclonal, Merck-Millipore, 1:500), PSD-95 (rabbit monoclonal, 1:500, Cell Signaling), anti-synaptobrevin (VAMP2, mouse monoclonal, 1:500, SySy), anti-p62 (SQSTM1, rabbit polyclonal, 1:1,000, MBL), anti-Ubiquitin (rabbit polyclonal, 1:200, Abcam) and anti-TUJ1 (mouse monoclonal, 1:2,000, Covance) (Supplementary Table 6). DAPI dihydrochloride (0.5 μg/ml) was used for nuclear staining.

### Confocal Microscopy and Image Analysis

Images were obtained with both Leica SP5-II upright and inverted confocal microscopes and Fiji v2.0.0 software was used to perform background subtraction and thresholding. For the counting of phospho-α-Syn accumulations in the *SNpc*, tiled confocal images of five sections/animal, starting from 2.66-mm anteroposterior distance relative to bregma [29] and collected every four, were counted using Fiji v2.0.0 software. For the assessment of gliosis in the dorsal *striatum* of the injected mice, six sections/animal with a 5-section interval along the rostrocaudal axis, 30-μm thick, were co-stained with antibodies against GFAP and Iba-1, and the obtained low magnification (10x) confocal images were analyzed with Fiji v2.0.0 software. For each brain slice, the ratio of the Raw Integrated Intensity (Raw Int Intensity), normalized to the surface area corresponding to the ipsilateral *striatum*, was measured. Gliosis in each animal was estimated as the average value of the Raw Int Intensity/ surface area of the six sections. For the quantification of phospho-α-Syn in the *striatum* and *SNpc* of the ExE-EV-inoculated mice, confocal images of phospho-α-Syn/TH co-staining (three sections per animal) were analyzed by Imaris 9.5.1 software (Bitplane, South Windsor, CT, USA). A 3D reconstruction of the TH positive somata (*SNpc*) and axons (*striatum*) was created using the surface tool and via the mask tool, the signal of phospho-α-Syn channel within the TH surfaces was selected. Following the construction of a surface corresponding to the selected phospho-α-Syn channel signal, the respective voxels were calculated and normalized to the total volume of the image stack. For the analysis of the synaptic integrity, confocal images of the PSD-95/VAMP2 were processed with the Huygens DeconvolutionLab2 plugin [34], as previously described [35]. For the co-localization analysis of the synaptic markers, 3D reconstructions of confocal images were performed by Imaris 9.5.1 software (Bitplane, South Windsor, CT, USA) and were further analyzed using the surface function. The density of each synaptic marker was quantified as the number of voxels occupied by the respective channel normalized to the total number of voxels that corresponded to the image stack. The Manders’ coefficients (M1, M2), which are measures of co-occurrence, were used to quantify PSD-95/VAMP2 co-localization.

### Assessment of the nigrostriatal axis integrity

For the assessment of the integrity of the nigrostriatal route, 30-μm thick sections were collected along the rostrocaudal axis of the *striatum* and midbrain of three mice/group. Specifically, six sections for the *striatum* and ten sections for the ventral midbrain per animal, with a 5- and a 3-section interval, respectively, were stained for TH. Following incubation with the secondary antibody (Vectastain Elite ABC kit, Vector labs), 3,30-diaminobenzidine (DAB; Dako) was used as chromogen, as previously described [36]. Mounted striatal sections were scanned using the slide scanner Primo Histo XE and the dopaminergic TH fiber density was measured with Fiji v2.0.0 software. The total number of TH-positive neurons in the *SNpc* was counted using the Stereo Investigator v10.0 software (MBF Bioscience, United States). The counting contours were drawn under a 2.5 x objective, whilst the counting was performed using a 63 x glycerol immersion objective. For the analysis, the following parameters were set: optical dissector height (12 μm), grid size (100 μm), counting frame (50 μm), and acceptable coefficient of error (Gundersen, *m* = 1) ≤ 0.1.

### Behavioral Analysis

The motor phenotype of the ExE-EV-inoculated mice was assessed two-months post-injection. One week prior to behavioral analysis, mice were handled on a daily basis to minimize stress and anxiety. Before each behavioral experimentation, mice were allowed to acclimate to the testing room for a minimum of 30 min. A battery of tests was implemented to evaluate locomotion, forelimb asymmetry, grip strength and neuromuscular co-ordination as previously reported [36]. Open Field (spontaneous locomotor activity): Mice were placed separately in the center of an open-field apparatus that consisted of a squared Plexiglass chamber. Free-moving animals within the arena were monitored individually by an overhead digital camera (Panasonic WV-BP332), for 10 min. Locomotor activity was analyzed using Ethovision XT 8.5 (Noldus) software and expressed as total distance traveled and mean velocity. Rotarod (motor coordination and balance): Mice were placed on 7-cm diameter rotating rods and were left walking face forward under steady rotation speed (4 rpm), for at least one min (revolutions per min). Past training time, the speed of the rotation was progressively increased from 0 to 40 rpm over 5 min. Latency to fall or passive rotation were recorded. Three 5-min trials were conducted with 1 h resting intervals in between. The mean latency for all three trials was used for analysis. Cylinder test (forelimb asymmetry): Mice were placed in a transparent acrylic cylinder and their innate tendency to rear up, touching the cylinder wall was recorded for 5 min, as previously described [37]. The number of times that each mouse touched the cylinder wall was scored for the left, right, or both paws. The results were expressed as the ratio of the usage of contralateral forelimb paw *versus* the use of ipsilateral paw. The index of unilateral trauma is reflected by the preferential use of contralateral forelimb paw.

### LC/MS and proteomic analysis data

Sample preparation: The ExE-EV samples were subjected to complete cell lysis using a buffer consisting of 4% SDS, 100 mm Tris/HCl, 100 mm DTT, pH 7.6 and incubated at 95 °C for 5 min. The lysed samples were further sonicated for 30 min in a water bath. The protein extracts were purified from debris by centrifugation for 20 min at 17,000 x g. The supernatants were transferred to clean tubes and processed according to the Single-Pot Solid-Phase-enhanced Sample Preparation (SP3) method of Hughes, without acidification and including a step of protein alkylation in 100 mM Iodoacetamide. Digestion was carried out by continuous shaking at 1,400 rpm at 37 °C using 0.25 μg Trypsin/LysC mixture (Promega) in a 25 mM ammonium bicarbonate buffer. Next day, the magnetic beads were removed and the peptidic samples were further purified by Sp3 peptide cleanup (REF) and evaporated to dryness in a vacuum centrifuge. The dried samples were solubilized in Buffer A, sonicated for 5 min and the peptide concentration was determined by measuring the absorbance at 280 nm using a nanodrop.

Ultra high pressure nanoLC: Each biological sample was analyzed three times (technical replicas). Samples were run on a liquid chromatography tandem mass spectrometry (LC-MS/MS) setup consisting of a Dionex Ultimate 3000 nano RSLC online with a Thermo Q Exactive HF-X Orbitrap mass spectrometer. Peptidic samples were directly injected and separated on an 25 cm-long analytical C18 column (PepSep, 1.9μm3 beads, 75 µm ID) using an one-hour long run, starting with a gradient of 7% Buffer B (0.1% Formic acid in 80% Acetonitrile) to 35% for 40 min and followed by an increase to 45% in 5 min and a second increase to 99% in 0.5 min and then kept constant for equilibration for 14.5min.

MS/MS: The eluted peptides were ionized by a nanospray source and detected by a Q Exactive HF-X mass spectrometer (Thermo Fisher Scientific, Waltham, MA, USA) operating in a data dependent mode (DDA). The peptides were measured from 350-1,500 m/z, using a resolving power of 120K for MS1, AGC at 3e6, maximum injection time of 100ms, followed by 12 MS/MS of the most abundant 2^+^-4^+^ charged ions using a resolving power of 15K, AGC at 1e^5^, maximum injection time of 22ms, and an isolation window of 1.2 m/z at 28 NCE and a dynamic exclusion of 30s. The software Xcalibur (Thermo Fisher Scientific) was used to control the system and acquire the raw files and internal calibration was activated using a lock mass of m/z 445.12003.

Data Analysis. The raw files were searched, and the identified peptides and proteins were quantified using Label Free Quantitation (LFQ) in MaxQuant (version 1.6.17.0), using search against the Mouse uniprot reviewed protein database (downloaded 16/04/2021) and against the default contaminants database. Search parameters included a molecular weight ranging from 350 to 5,000 Da, a precursor mass tolerance of 20 ppm, an MS/MS fragment tolerance of 0.5 Da, a maximum of two missed cleavages by trypsin, and methionine oxidation, deamidation of asparagine and glutamine and protein N-terminal acetylation were set as variable modifications. Carbamidomethyl was set as fixed cysteine modification. The protein and peptide false discovery rate (FDR) was set at 1%. The match-between-run function was enabled. The statistical evaluation between groups was performed using the Perseus software (version 1.6.10.43). Proteins identified as “potential contaminants”, “reverse” and “only identified by site” were filtered out. The LFQ intensities were transformed to logarithmic. Zero intensity was imputed i.e., replaced by normal distribution, assuming that the corresponding protein is present in low amounts in the sample. Three biological replicas plus three corresponding technical replicas were grouped for each treatment and a two-sided Student T-test of the grouped proteins was performed using p value (>0.05) for truncation. Enrichment analysis was performed for the significantly differentiated proteins, using DAVID [38, 39] and Ingenuity Pathway Analysis (IPA,Quiagen).The mass spectrometry proteomics data have been deposited to the ProteomeXchange Consortium via the PRIDE [40] partner repository with the dataset identifier PXD036301.

### RNA isolation, RNA sequencing and Transcriptomic data analysis

The ipsilateral *striatum* of exosome-inoculated mice was excised, and total RNA was isolated using the PureLink® RNA Mini Kit, according to the manufacturer’s instructions. The purity and the concentration of RNA were determined by electrophoresis and spectrophotometric analysis at 260/280 nm, using a NanoDrop-1000 (ThermoFisher Scientific, USA). 750 ng of total RNA were used for the preparation of cDNA libraries, using the NebNext Ultra II RNA library prep kit for Illumina (NEB #E7775), according to the manufacturer’s instructions. The quality of libraries was evaluated using the Agilent DNA 1000 Kit (Agilent #5067-1504) with an Agilent 2100 Bioanalyzer. Quantification was carried out with Qubit dsDNA HS Assay Kit (ThermoFisher Scientific #Q32851) and isomolar quantities of up to 24 cDNA libraries, barcoded with different adaptors, were multiplexed. Sequencing was performed with a NovaSeq SP 100c kit (Illumina) in a single-end manner at the Greek Genome Center (BRFAA), generating 100 bp long reads and about 24-30 million reads per library. Three biological replicates were sequenced per condition. Raw sequence data in FastQ format were uploaded to the Galaxy web platform, and standard tools of the public server “usegalaxy.org” [41] were used for subsequent analysis, as previously described [42]. Briefly, quality control of raw reads was performed with FastQC (v072+galaxy1), followed by removal of adapter sequences and low-quality bases using Trim Galore! (v0.6.3). For alignment of trimmed reads to the mouse reference genome [University of California, Santa Cruz (UCSC) Genome Browser on Mouse July 2007 (NCBI37/mm9)] HISAT2 (v2.1.0+galaxy5) was applied. Assessment of uniform read coverage for exclusion of 5'/3' bias and evaluation of RNA integrity at a transcript level were performed using Gene Body Coverage (v2.6.4.3) and Transcript Integrity Number (v2.6.4.1) tools, respectively. Differential gene expression was determined with DESeq2 (v2.11.40.6), using the count tables generated from HTSeq-count (v0.9.1) as input. Cutoff values for differentially expressed genes were baseMean >30 and adjusted p-value (false discovery rate, FDR) <0.1. Bioinformatics analysis was performed using Ingenuity® Pathway Analysis (IPA®, Qiagen). Venn diagrams were constructed with Venny 2.1 (developed by Oliveros, J.C., 2007) and Venn Diagram Plotter software (Pacific Northwest National Laboratory, U.S. Department of Energy). Heatmaps were generated with Morpheus software (https://software.broadinstitute.org/morpheus). Raw sequence reads for this study can be found in the Sequence Read Archive database of National Center for Biotechnology Information (https://www.ncbi.nlm.nih.gov/sra), under the accession number PRJNA847043.

### Live Calcium Imaging

For live calcium imaging, SH-SY5Y neuroblastoma cells were plated on a 24-well plate (45,000 cells per well) and cultured in the presence of 10 μM all-trans retinoic acid (Calbiochem) in Dulbecco’s modified eagle's medium (DMEM, Hyclone) supplemented with 10% exosome-depleted FBS for 10 days. Eight μg of exosome-enriched fraction C / 10^6^ cells were added on the 6^th^ and 8^th^ day *in vitro*, as we have estimated that this is the amount of exosomes physiologically secreted by SH-SY5Y cells every 48 h. On the 10^th^ day, live calcium imaging was performed, as previously described [43]. Briefly, following loading with 2 μM Fura 2-AM (Calbiochem) in culture medium for 30 min at 37^o^C, cells were returned to culture medium and incubated for additional 2 h at 37^o^C. Fura-2AM-loaded cells were washed once with microscopy buffer (129 mM NaCl, 2mM CaCl_2_, 5 mM KCl, 1 mM MgCl_2_, 30 mM glucose, 1% BSA, 25 mM HEPES pH 7.4) and stimulated in the same buffer with the addition of 55 mM KCl. Sequential fluorescence images, with a 10-sec interval, were obtained at 340 and 380 nm excitation and 510 nm emission by a TE 2000U-inverted fluorescence microscope (Nikon, Osaka, Japan) coupled to a cooled charge-coupled device (CCD) camera (PTI-IC200, Princeton Instruments). Changes in free intracellular calcium were determined using temporal analyses of single cells to express the data as fluorescence ratios [44], with the aid of “the region of interest” tool of Fiji v2.0.0 software. The baseline fluorescence was determined at the beginning of each experiment by obtaining the average of the first 20 data points before KCl stimulation. KCl-induced calcium entry was expressed as an increase relative to the baseline. The graphs represent the average peak response.

### Statistical Analysis

Data are expressed as the mean ± standard error of the mean (SEM). The statistical analysis was performed with the GraphPad Prism 8 software (Version 8.2.0) (San Diego, CA), using Unpaired Student’s *t*-test (N>6) or non-parametric Mann-Whitney test (N<6) for comparison between two treatments, within the same genetic background (WT or α-Syn KO mice). One-way ANOVA followed by Tukey’s post-hoc test (N>6) or non-parametric Kruskal-Wallis followed by Dunn’s test (N<6) were used for comparison between multiple treatments, again within the same genetic background (WT or α-Syn KO mice). Finally, for comparisons between multiple treatments and between two different genetic backgrounds (WT and α-Syn KO mice), two-way ANOVA was applied. For each comparison the p value is stated in the respective figure.

## RESULTS

### Isolation and characterization of whole brain exosome-enriched extracellular vesicles from PFF-inoculated mice

**Figure 1.**
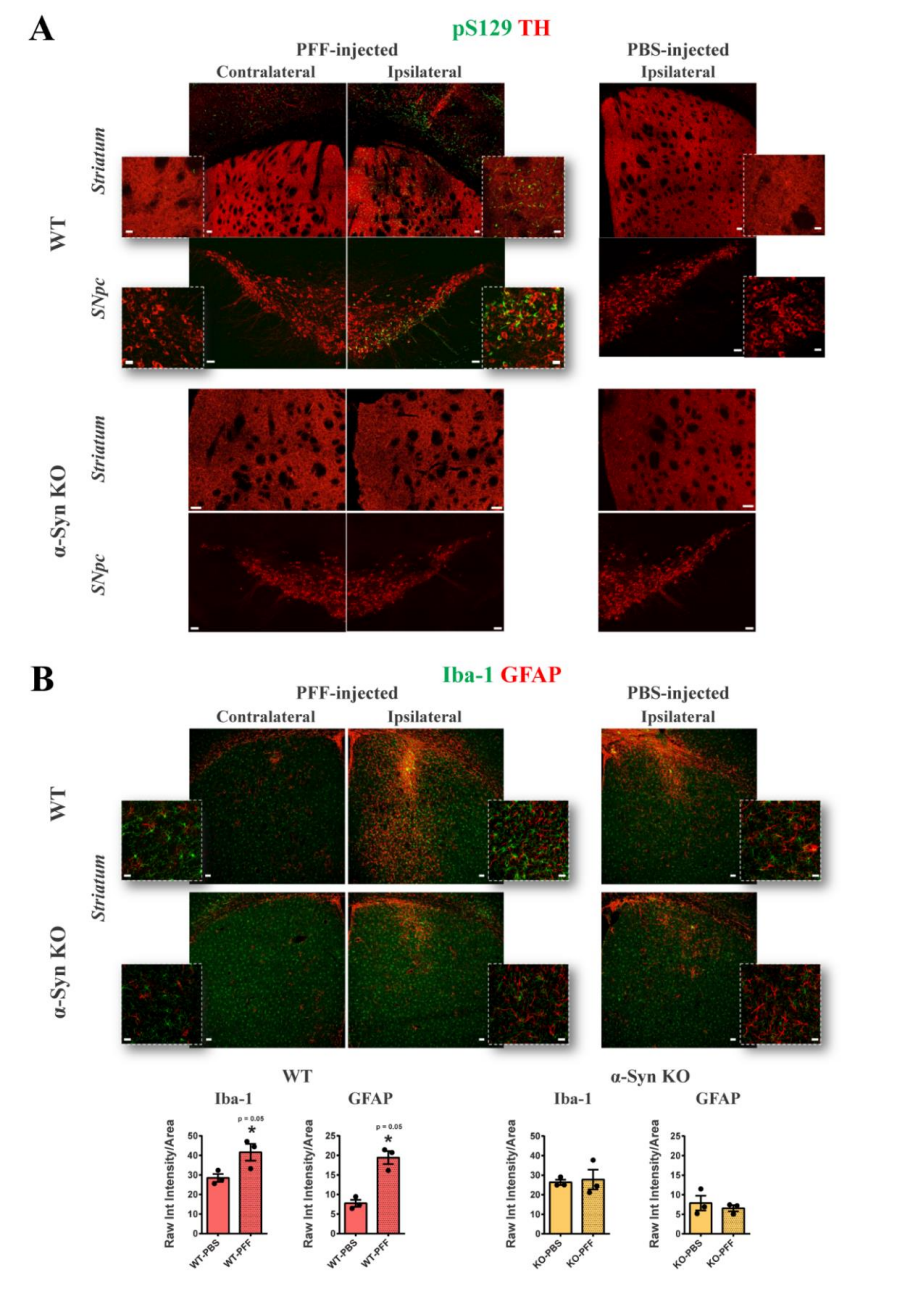
**Intrastriatal inoculation of mouse PFFs results in a robust α-Syn pathology and significant gliosis only in WT mice**. **(A)** Representative tile scan confocal images of the contralateral and ipsilateral *striatum* and *SNpc* of PFF- injected WT (upper panel) and α-Syn KO (lower panel) mice, one-month post-injection, showing double immunostaining of phosphorylated-α-Syn (green) and tyrosine hydroxylase (TH, red). Tile scans were obtained with 20x lens (zoom 1.5, scale bar 50 μm) and high magnification insets with 63x lens (scale bar 20 μm). The respective images of the ipsilateral side of the PBS-injected control groups are included (right panel). **(B)** Representative confocal images of Iba-1 (green) and GFAP (red) in the contralateral and ipsilateral *striatum* of PFF-inoculated WT (upper panel) and α-Syn KO (lower panel) mice, one-month post-injection [(low magnification; 10x, scale bar 50 μm and high magnification; 63x, scale bar 20 μm)]. On the right panel the respective images of the ipsilateral *striatum* of the PBS-injected control groups are shown. (n = 3). Quantification graphs illustrate the estimated microgliosis (left) and astrogliosis (right), at one-month post-intrastriatal injections, expressed as Raw Integrated intensity of Iba-1 and GFAP, respectively, normalized to the section area. (n = 3 animals per group). Data represent mean values ±SEM. Differences were estimated using nonparametric Mann-Whitney. α-Syn: alpha-synuclein, KO: knockout, PFFs: alpha-synuclein preformed fibrils, *SNpc*: *Substantia nigra pars compacta*, WT: wild type.

Six-month-old WT or α-Syn KO mice, inoculated intrastriatally with either mouse PFFs (Supplementary Fig. 1A) or respective volume of PBS (control group), were used as a source of interstitial brain-derived exosome-enriched extracellular vesicles (ExE-EVs), at one-month post-injection. At this time point, WT exosome-donor mice, receiving PFFs, exhibit α-Syn pathology in the ipsilateral *striatum* and *SNpc*, as depicted by the presence of dense phospho-α-Syn immunoreactivity in the TH positive dopaminergic neuronal axons and somata, respectively (Fig. 1A, upper panel). Quantification of the phospho-α-Syn accumulations throughout the ipsilateral *SNpc* revealed that a percentage of 15 ± 0.93% of the dopaminergic neurons were double positive for TH and phospho-α-Syn. Phospho-α-Syn accumulations were also detected in the ipsilateral cortical areas (Fig. 1A, upper panel). In accord with other studies [36, 45], PFF-induced phospho-α-Syn pathology within the TH positive somata was accompanied by accumulation of p62 and ubiquitin, reminiscent of LB-like intraneuronal aggregates (Supplementary Fig. 1B). In addition, concomitant induction of astrogliosis and microgliosis was observed in the ipsilateral *stiatum* of PFF-injected WT mice, as shown by the significant increase of GFAP and Iba-1 immunoreactivity, respectively (Fig. 1B, upper panel).

Of note, we [36] and others [45, 46] have reported that PFF-induced nigrostriatal degeneration commences between 60 to 90 days after stereotactic surgery, depending on the dosage and the origin (human or mouse) of fibrils, and not as early as 30 days. Taken all together, isolating brain-derived ExE-EVs as early as one-month post-injection, allows us to assess whether exosomal cargo, at a time point that reflects only early events of pathology and not end points characterized by neuronal death and motor deficits, could potentially propagate disease phenotype. Finally, in order to investigate whether the presence of endogenous α-Syn is critical for the transmission of pathology, we also isolated ExE-EVs from PFF-injected α-Syn KO mice at one-month post- injection. The latter did not exhibit any sign of either phospho-α-Syn pathology or enhanced gliosis in the injected site (Fig. 1A, B, lower panels), plus they have been shown to be resistant to PFF-induced dopaminergic neuronal demise [45].

To this end, PFF- or PBS-injected hemispheres from WT and α-Syn KO mice were excised, and subjected to enzymatic digestion, sequential centrifugations, and sucrose gradient purification. Following isolation, the three main exosome-enriched fractions, B, C and D, were analyzed for their integrity and purity. As shown by EM (Supplementary Fig. 2A, left panel), all three fractions contained intact double-membrane vesicles, bearing the hallmark cup-shaped, morphology of exosomes. However, size distribution analysis of the EM images revealed that only in fraction C the majority of vesicles ranged between 50 nm - 200 nm, whereas fractions B and D contained a high amount of smaller (<50 nm) and larger (> 200 nm) extracellular vesicles, respectively (Supplementary Fig. 2A, right panel). For further determination of the size distribution and relative concentration of the isolated brain exosomes in the sucrose gradient fractions, we performed Nanoparticle Tracking Analysis (NTA) that verified their high abundance in fraction C (Supplementary Fig. 2B). Additionally, the significantly elevated Acetylcholinesterase (AChE) enzymatic activity (Supplementary Fig. 2C), as well as the increased levels of the exosome-specific marker flotillin (Supplementary Fig. 2D) measured in fraction C, further validated its enrichment in exosomes over the other two fractions.

**Figure 2.**
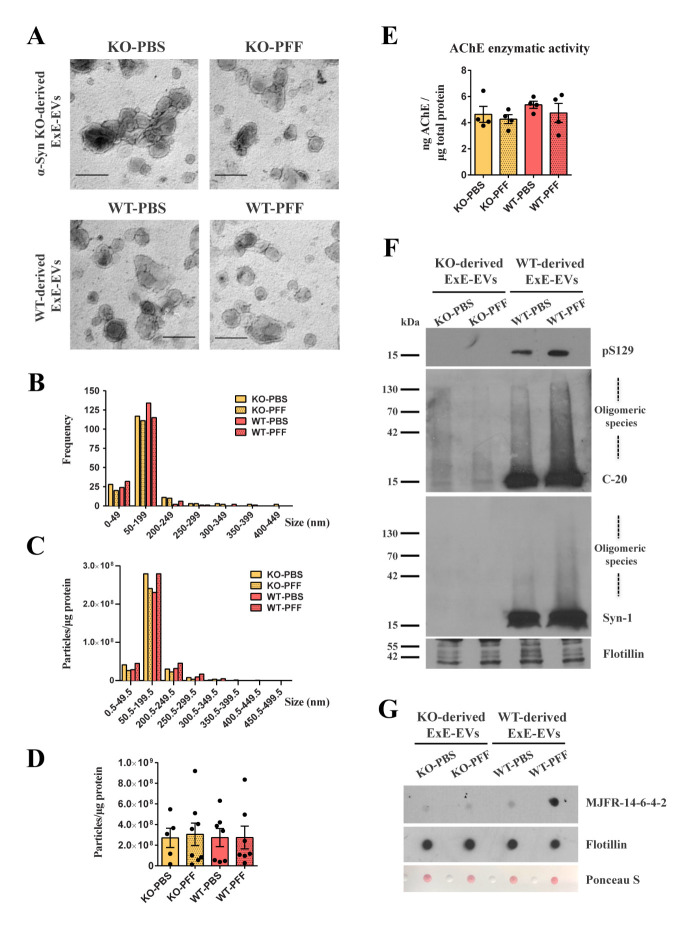
**Characterization of exosome-enriched fraction C isolated from PFF-injected WT and α-Syn KO mouse brains, at one-month post-inoculation**. **(A)** Representative high magnification EM images of exosome-enriched fraction C isolated from the PFF-injected WT and α-Syn KO mice and their respective PBS controls (34,000x, scale bar 200 nm). **(B)** Quantification graph of the EM images depicting the size distribution of the isolated fraction C shown in panel A. y-axis represents the frequency of the different size groups shown along the x-axis. **(C)** Graph demonstrating the size distribution of the isolated fraction C, as measured by NTA. y-axis represents the number of particles/μg of total protein of the different size groups shown along the x-axis. **(D)** Graph depicting the total number of particles/μg of total protein corresponding to the 4 experimental groups. Data represent mean values ± SEM (n = 5-8 independent brain exosome preparations/ group). **(E)** Graph depicting the concentration of AChE enzyme (ng) contained in the four groups of isolated fraction C (KO-PBS, KO-PFF, WT-PBS, WT-PFF), normalized to the total protein content (μg) measured by Bradford assay. Data represent mean values ± SEM (n = 4 independent brain exosome preparations). **(F)** Western blot analysis of the isolated fraction C for the presence of phospho- (pS129) and total α-Syn (C-20, Syn1). The exosome marker flotillin was used as a loading control. **(G)** Dot blot analysis of the brain-derived fraction C against MJFR-14-6-4-2, a specific antibody recognizing α-Syn aberrant forms. Equal loading was assessed by blotting against flotillin and Ponceau-S staining. Two-way ANOVA, was used in all panels. ExE-EVs: Exosome Enriched Extracellular Vesicles, EM: Electron Microscopy, ΝΤΑ: Nanoparticle Tracking Analysis, AChE: Acteylcholinesterase.

Importantly, neither the genetic background (WT or α-Syn KO) nor the PFF inoculation per se altered the disk-like shape of exosomes present in the respective C fractions, as shown by EM (Fig. 2A, Supplementary Fig. 3A). Consistently, the size distribution of vesicles in the three different sucrose gradient purified fractions remained unchanged amongst groups, with fraction C being the one enriched in 50 - 200 nm vesicles as estimated both by EM image analysis (Fig. 2B) and NTA (Fig. 2C, Supplementary Fig. 3A). In addition, no significant alteration was observed in the number of isolated ExE-EVs from the four different groups, as assessed by NTA (Fig. 2D). Consistently, AChE enzymatic activity remained unchanged, further supporting the similar abundance of ExE-EVs in the four different preparations (Fig. 2E). Moreover, as shown in Fig. 2F, only in ExE-EVs isolated from WT mice, we were able to detect both phospho- and total (monomeric and oligomeric) α-Syn, the levels of which were more pronounced in the PFF group. Of note, the observed increase in oligomeric α-Syn was mostly attributed to aberrant/misfolded forms of the protein, as shown by dot blot analysis, where only ExE-EVs isolated from PFF-injected WT mice exhibited immunoreactivity against the conformation-specific MJFR-14-6-4-2 antibody (Fig. 2G).

### Intrastiatal PFF inoculation results in alterations of the proteomic profile of the generated brain-derived ExE-EVs.

To further assess the purity of the isolated fractions C, we performed LC/MS analysis of 12 different exosome-enriched preparations. Proteomic profiling using the Max Quant software identified 1235 proteins (Supplementary Table 1) that were filtered for contaminants and had a 70% occurrence in the 4 experimental groups.

Gene Ontology (GO) analysis of the proteomic data, using the Database for Annotation, Visualization and Integrated Discovery (DAVID), verified “Extracellular exosome” as the top hit component (Supplementary Fig. 2E), which actually included a percentage of 49.7% of the total identified proteins (Supplementary Table 2). More specifically, the analyzed exosomal fractions were enriched in adhesion molecules such as tetraspanins (eg. CD47, CD81, CD82, and CD9) and integrins, membrane transport and fusion proteins, including annexins, flotillins, and Rabs, ESCRT, heat shock and signaling proteins, cytoskeleton and ion transport components, as well as metabolic enzymes (Supplementary Table 2); all these proteins have been found, by MS/MS analysis, to comprise characteristic components of exosomes from different cell types [47], [48]. Consistently, screening of the 614 identified exosome-specific proteins with the Vesiclepedia and ExoCarta databases showed a 71 % and 76% overlap, respectively (Supplementary Fig. 2F). Of note, proteins of organelles that are not related to exosomes and extracellular vesicles in general, such as laminin B1 (LMNB1, nucleus), ribosomal protein S6 and L7 (RSP6, RPL7, ribosome), Golgi matrix protein 130 (GM130, Golgi), carlticulin (CARL, ER) and others [49], [50], were not amongst the detected proteins (Supplementary Table 1), highlighting the lack of contaminants and further validating the enrichment of the described brain-derived preparation in exosomes. Moreover, tissue expression analysis pinpointed the hippocampus, brain and brain cortex as the top tissues of origin, further verifying the source-specificity of the isolated ExE-EVs (Supplementary Fig. 2E, Supplementary Table 3). Overall, these findings verify that the vesicular preparations purified from the 4 experimental groups were highly enriched in brain exosomes and that the latter were found in similar concentrations, therefore any differences detected amongst the groups were not attributed to a heterogeneous starting material.

**Figure 3.**
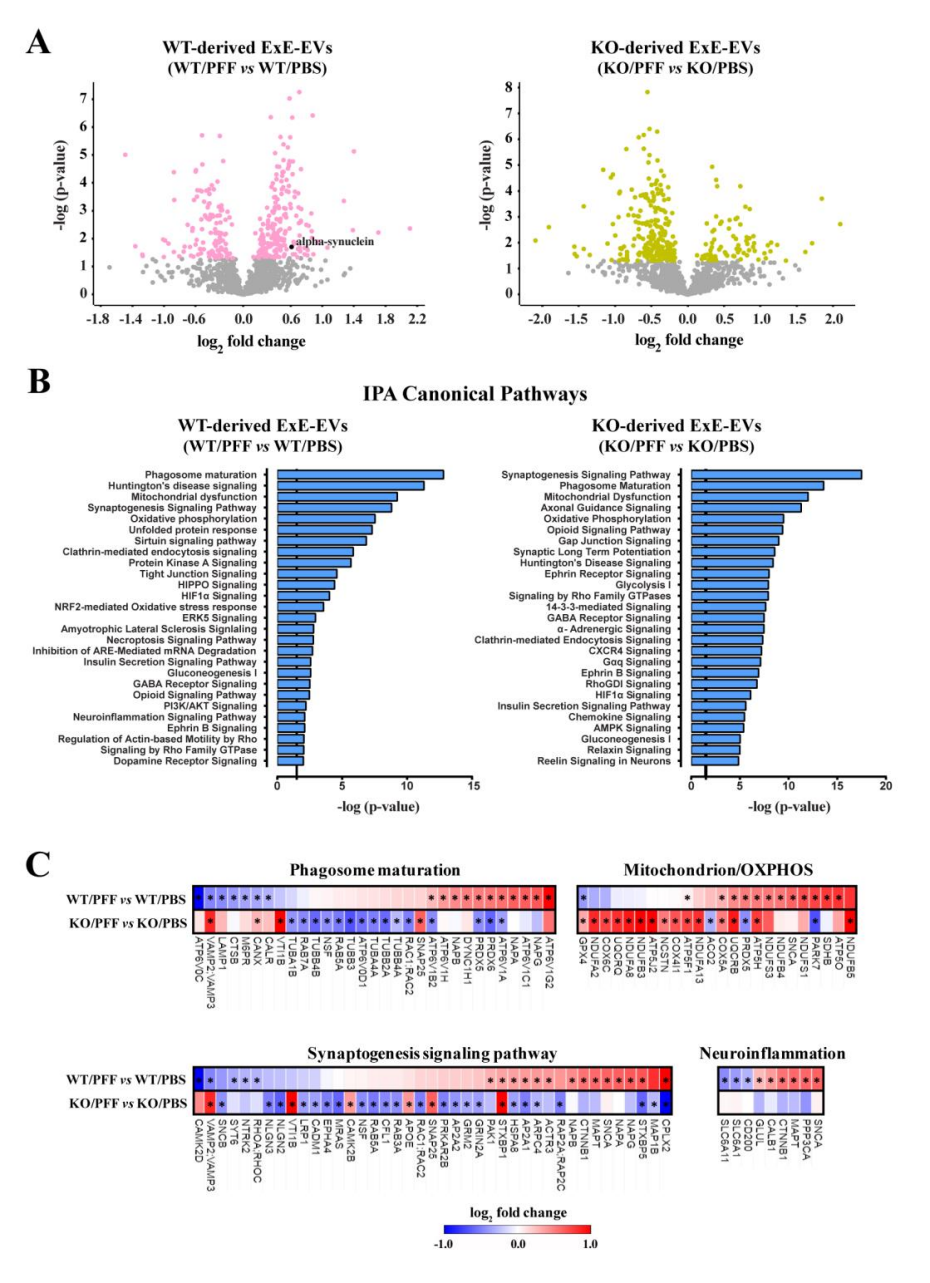
**Intrastiatal PFF inoculation leads to significant alterations of the exosomal protein content in both WT and α-Syn KO backgrounds**. **(A)** Volcano plots illustrating the significantly altered proteins, following PFF inoculation, in the WT (left panel) and α-Syn KO mice (right panel) (Nano LC-MS/MS analysis was performed for n=3 samples per group. **(B)** Graphs depicting the canonical pathways identified by IPA analysis of the differentially expressed proteins, in both WT and α-Syn KO groups. CNS-related pathways, selected among the top regulated, are displayed along the *y*-axis and their statistical significance [-log (p-value)] along the *x*-axis. Black vertical line shows the threshold for statistical significance (p = 0.05) (C) Heatmaps of the top common IPA canonical pathways comparing the expression patterns of the differentially altered proteins between the WT and KO groups. The significantly altered proteins in each background are marked with an asterisk (*). Nano LC-MS/MS: Nanoscale liquid chromatography coupled to tandem mass spectrometry, IPA: Ingenuity Pathway Analysis, CNS: Central Nervous System.

Further proteomic analysis revealed that 315 proteins were found differentially regulated between ExE-EVs derived from PBS- and PFF-injected WT mice and 303 proteins between exosome groups in the mice of α-Syn KO background (Fig. 3A, Supplementary Tables 4A, 5A). Notably, the majority of significantly altered proteins in the WT background were upregulated (60.6%), whilst the majority of significantly altered proteins in the α-Syn KO groups were downregulated (74.25%) (Fig. 3A). Cellular component analysis of the differentially regulated proteins in both WT and α-Syn KO ExE-EV preparations, using DAVID database, revealed extracellular exosome, myelin sheath, membrane, and cytosol, amongst the top regulated GOs (Supplementary Fig. 3B).

Moreover, bioinformatics analysis of the differentially expressed proteins with a fold change > 1.2, between PFF and PBS groups in both WT (270 proteins, Supplementary Table 4B) and α-Syn KO (271 proteins, Supplementary Table 5B) background, using the Ingenuity Pathway Analysis platform (IPA, Qiagen), pinpointed pathways related to synaptic activity [synaptogenesis signaling pathway, opioid signaling pathway, synaptic long term potentiation (LTP)], mitochondrial function [mitochondrial dysfunction, oxidative phosphorylation (OXPHOS)], as well as processes related to cellular metabolism (phagosome maturation, OXPHOS, glycolysis, glyconeogenesis) (Fig. 3B). Of note, these pathways have also been shown to be significantly altered in PD patients [51]. Interestingly, the impact of PFF inoculation on the exosomal cargo, in both backgrounds, is shown to affect similar pathways (Fig. 3B). However, analyzing further the top common IPA canonical pathways namely the Phagosome maturation, Mitochondrion/ OXPHOS, Synaptogenesis signaling pathway and Neuroinflammation, we uncovered an overall reverse pattern of regulation in the majority of the proteins between the WT and α-Syn KO backgrounds (Fig. 3C). Most of the differentially regulated exosomal proteins in the WT background (e.g. ATP6V0C, VAMP3, LAMP1, CALR, RAB7A, TUBA4A, RAC1, PARK7, SNCA, CAMK2, PAK1, HSPA8, ARPC4, ACTR3, RAP2C, CTNNB1, MAPT, PPP3CA, GLUL, SLC6A1, SLC6A11), which are classified in the top regulated IPA pathways (Fig. 3B, C, Supplementary Table 4B), are altered in the same fashion as identified DEGs in PD patients pinpointed by transcriptomics meta-analysis studies [52]. Given the fact that only WT mice develop PD-like pathology following PFF injection, whereas α-Syn KO mice do not, and that exosomes comprise a snapshot of the environment of origin, one could argue that the observed changes in the exosomal protein content may mirror the different response in the same insult.

**Figure 4.**
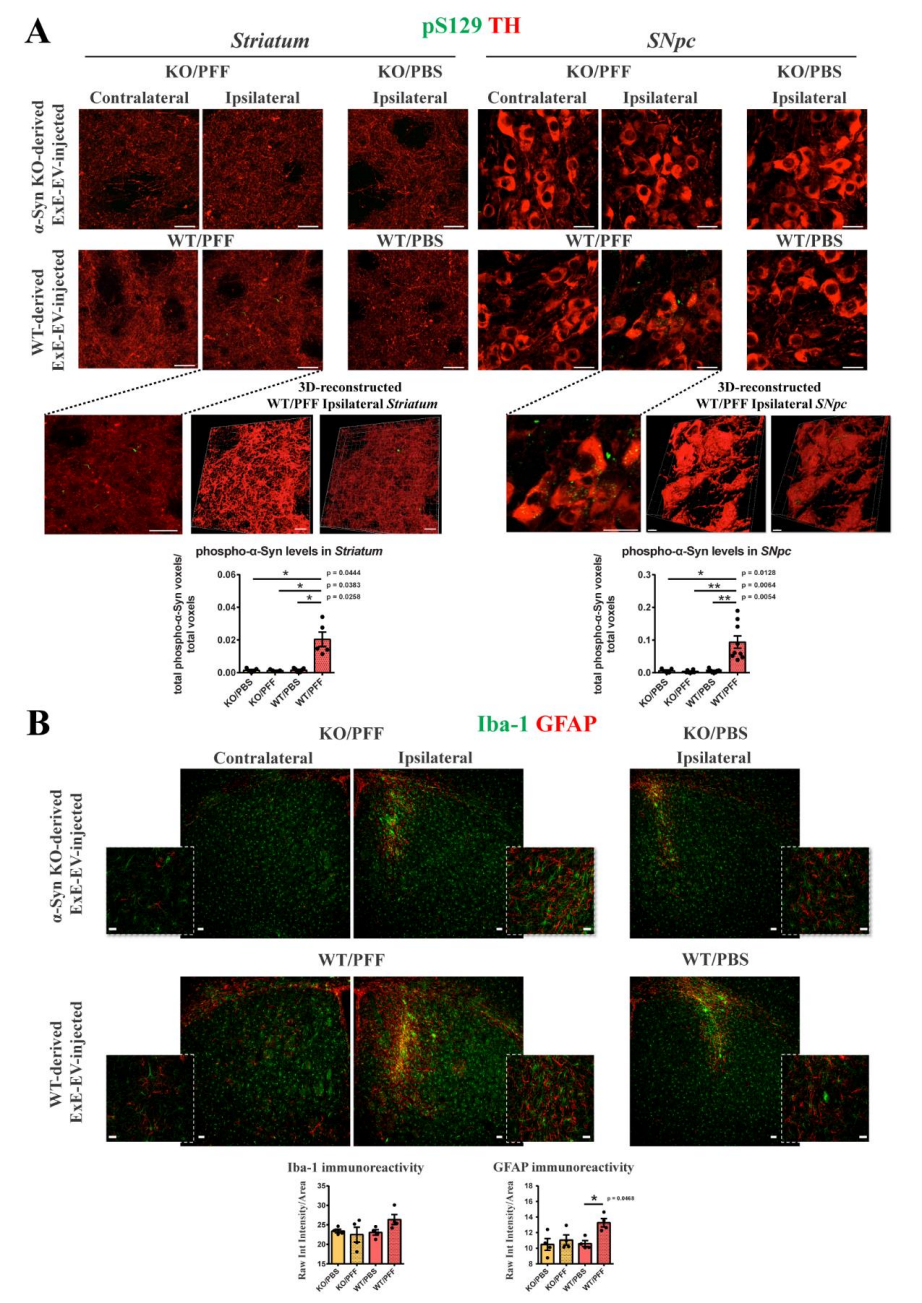
**Assessment of α-Syn pathology in the nigrostriatal axis of WT mice inoculated with isolated brain ExE-EVs, at two-months post-injection**. WT mice were stereotactically injected with 5.5 μg of brain-derived exosomes in the right dorsal *striatum*. **(A)** Representative confocal images showing double immunostaining against phospho-α-Syn (green, pS129) and TH (red) in the contralateral and ipsilateral *striatum* (left panels) and *SNpc* (right panels) of ExE-EV-injected WT mice, [(63x, scale bar, 20 μm)]. 3D reconstruction with Imaris software of the confocal images from the ipsilateral *striatum* and *SNpc* of the WT/PFF ExE-EV-injected mice, verify the presence of phospho-α-Syn species within the TH positive fibers [(bottom left, 63x, scale bar 20 μm, 3D reconstructed images scale bar 10 μm)] and cell somata [(bottom right, 63x, scale bar 20 μm, 3D reconstructed images scale bar 5 μm)]. Quantification graphs depicting the total volume (voxels) of phospho-α-Syn in the *striatum* (left) and *SNpc* (right) normalized to the total image volume, following analysis with Imaris software, (n = 5-9 animals per group). Data represent mean values ± SEM. **(B)** Representative confocal images of Iba-1 (green) and GFAP (red) in the contralateral and ipsilateral *striatum* of WT mice inoculated with KO/PFF (upper panels) or WT/PFF ExE-EVs (lower panels), two-months post-injection [(Low magnification 10x, scale bar 50 μm, high magnification 63x, scale bar 20 μm)]. Graphs illustrate the quantified micro- (left) and astro-gliosis (right), estimated as the ratio of the Raw Integrated intensity of Iba-1 and GFAP, respectively, normalized to the section area. (n =3-4 animals per group). Data represent mean values ± SEM. Kruskal-Wallis, followed by Dunn’s test, was used in all panels. 3D: 3 dimensional.

### Assessment of α-Syn pathology in the nigrostriatal system of mice receiving ExE-EVs from PFF-inoculated mice.

To investigate whether ExE-EVs originating from a pathogenic environment can propagate pathology to a healthy host brain, six-month old WT mice received unilateral intrastriatal injections of 5.5 μg exosome-enriched fraction C isolated from the 2 WT groups, namely WT/PBS, and WT/PFF, a dose previously described by our as well as other groups [19, 22, 30, 31]. Two-months post-injection, the host mice were sacrificed, and α-Syn pathology was assessed in the isolated brain tissue by immunohistochemical analysis. Interestingly, at this time point, phospho-α-Syn immunoreactivity was detected both in the ipsilateral *striatum* and *SNpc* (Fig. 4A), as indicated by its colocalization with TH in dopaminergic terminals and somata, respectively. Three-dimensional representation/reconstruction of the TH positive axons and somata confirmed the presence of phospho-α-Syn within them (Fig. 4A, bottom panel). More specifically, only when transparency of the TH surfaces was increased, phospho-α-Syn puncta were revealed, further verifying that these were enclosed in the dopaminergic neurons (Fig. 4A, bottom panel). Of note, mice receiving a higher dose of the WT/PFF ExE-EVs (15 μg), for the same period, displayed a more robust phospho-α-Syn phenotype (Supplementary Fig. 4A). In particular, these mice exhibited phospho-α-Syn accumulations within the TH positive somata and axons (Supplementary Fig.4A), the fibrillar nature of which was further supported by their immunoreactivity with a conformation specific antibody (Supplementary Fig. 4B). Further analysis revealed that the observed phospho-α-Syn positive dopaminergic soma inclusions were characterized by the accumulation of p62 and ubiquitin, markers related to LB-like structures (Supplementary Fig. 4C).

Next, we assessed the levels of gliosis in the ipsilateral *striatum*, showing that only mice treated with WT/PFF ExE-EVs exhibited an induction of astrogliosis, at two-months post-injection, as demonstrated by the increased GFAP immunoreactivity and subsequent quantification (Fig. 4B). However, the described pathology was not accompanied by the demise of the integrity of the nigrostriatal axis, as evaluated by densitometric analysis of the TH dopaminergic fibers in the *striatum*, as well as stereological counts of the TH positive dopaminergic somata in the *SNpc* (Supplementary Fig. 5A). In addition, we did not observe any significant alteration in any aspect of the motor phenotype tested, either regarding locomotion (open field test), grip strength and muscular co-ordination (rotarod), or forelimb asymmetry (cylinder test) (Supplementary Fig. 5B). Importantly, inoculation with ExE-EVs from either group of α-Syn KO mice, which were shown to be resistant to PFF-induced phenotype, did not confer any phospho-α-Syn pathology (Fig. 4A), induction of astrogliosis (Fig. 4B), neuronal cell death (Supplementary Fig. 5A) or behavioral defects (Supplementary Fig. 5B), at the same time point. No phospho-α-Syn pathology was observed even after inoculation with the higher dose of either KO-derived ExE-EVs in the WT host mice at two-months post-injection (Supplementary Fig. 4A).

Finally, in order to visualize the uptake of WT/PFF ExE-EVs and their homing within the brain, as well as to assess their role in the observed phenotype, 5.5 μg of DiI-labelled WT/PFF ExE-EVs were injected intrastriatally in WT mice. As early as two-weeks post-inoculation, aberrant α-Syn bearing ExE-EVs were found internalized by neurons, astrocytes and microglia in the ipsilateral *striatum* (Supplementary Fig. 6A). No free DiI dye was detected in any of the control-injected mice (Supplementary Fig. 6B).

**Figure 5.**
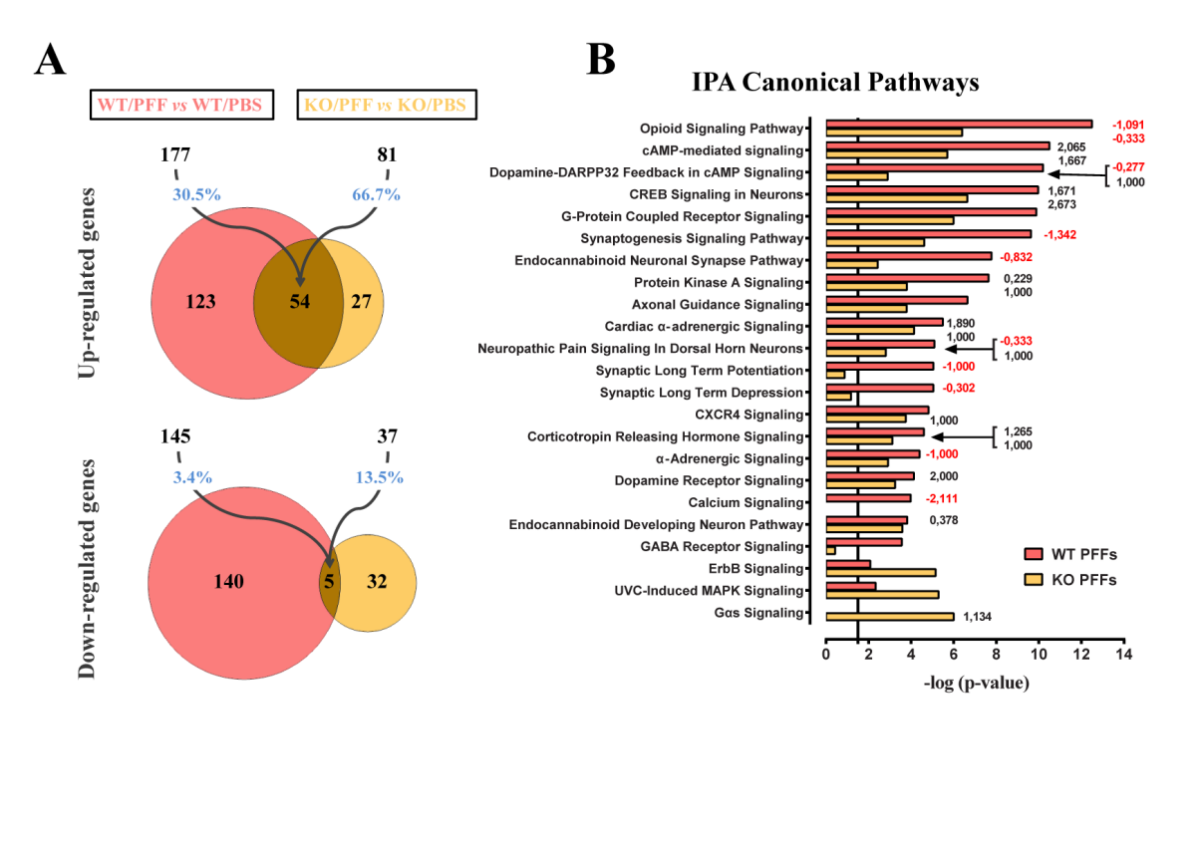
**Alterations in the transcriptomic profiling of WT mouse *striatum*, two-months post-inoculation with brain-derived ExE-EVs**. **(A)** Venn diagrams summarizing RNA-Seq analysis results of the ipsilateral WT striatal tissues. Numbers of statistically significant (base Mean > 30 and FDR < 0.1) up- or down-regulated genes following inoculation with WT/PFF (pink) and KO/PFF (yellow) ExE-EVs, in comparison to their respective PBS controls, are depicted. The indicated percentages highlight the overlap between the two ExE-EV groups. **(B)** Graph demonstrating on the y-axis the CNS-related IPA canonical pathways, selected among the top 45 from RNA-Seq analysis and on the *x*-axis the statistical significance [-log (*P* value)]. The black vertical line is indicative of the threshold for statistical significance (*P* = 0.05). Numbers at the right side of the bars represent calculated IPA z-scores predicting an increased (black numbers) or a decreased (red numbers) pathway activity. FDR: false discovery rate (adjusted p-value), RNA-Seq: RNA-Sequencing.

### Inoculation of WT mice with ExE-EVs derived from PFF-injected mice results in perturbation of synaptic function.

PD pathology is not restricted to the presence of phospho-α-Syn and gliosis, but also extends to other deficits that could even precede neuronal degeneration [53]. In this respect, in order to better characterize the impact of striatal injections with ExE-EVs derived from PFF-injected mouse brains, we investigated potential alterations at the transcriptomic level. To this end, the ipsilateral *striatum* from WT/PBS, WT/PFF, KO/PBS and KO/PFF ExE-EV-inoculated mice were excised two months post-injection, and their transcriptomes were analyzed by RNA-Seq. Three hundred twenty-two (322) genes were found differentially expressed in the *striatum* of WT/PFF ExE-EV-inoculated mice, compared to WT/PBS ExE-EV-inoculated controls, 177 of which were upregulated and 145 were downregulated (Fig. 5A). Of note, we found much fewer differentially expressed genes (DEGs) from the analysis of the respective tissues from KO/PFF ExE-EV-inoculated mice. Specifically, from the 118 DEGs, 81 were upregulated and 37 were downregulated in the *striatum* of KO/PFF ExE-EV-inoculated mice, compared to the respective KO/PBS controls. PFF ExE-EV-injected striatal tissues exhibited highly overlapping transcriptional profiles, since ~66,7% of upregulated genes in KO/PFF ExE-EV-inoculated mice were also upregulated in the WT/PFF group, albeit to a different degree (Fig. 5A). Bioinformatics analysis using the IPA platform demonstrated that the DEGs in either of the groups receiving WT/PFF or KO/PFF ExE-EVs were clustered to canonical pathways mainly related to synaptic function, including, among others, Opioid signaling, Dopamine-DARP32 Feedback in cAMP signaling, Synaptogenesis signaling, Endocannabinoid Neuronal Synapse pathway, LTP, and Synaptic Long-Term Depression (LTD) (Fig. 5B).

**Figure 6.**
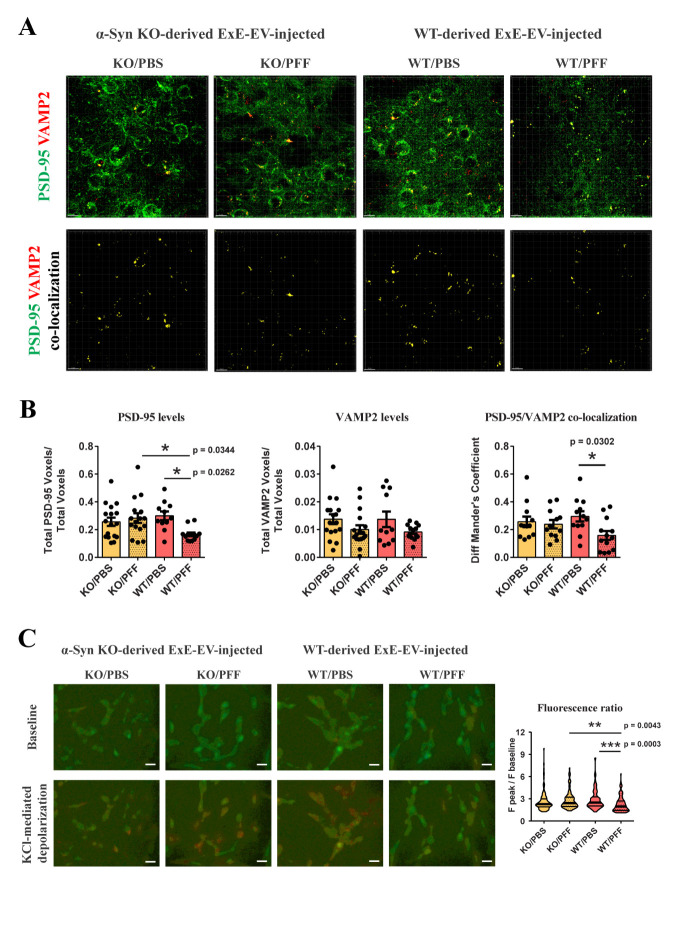
**ExE-EVs isolated from WT PFF-injected mice affect the synaptic integrity both *in vivo* and *in vitro***. **(A)** De-convolved confocal images showing immunofluorescence staining against the postsynaptic marker PSD-95 (green) and the presynaptic Synaptobrevin-2 (VAMP2, red) in the ipsilateral *striatum* of WT mice injected with brain ExE-EVs (upper panel). Images depicting spots that correspond to PSD-95/VAMP2 co-localization, as estimated by Imaris software are shown in the lower panel. **(B)** Quantification graphs measuring the total volume (voxels) of PSD-95 and VAMP2 normalized to the total image volume, following analysis with Imaris software. Co-localization of the two synaptic markers was estimated using Mander’s coefficient. Data represent mean values ± SEM. (n = 3-5 images/mouse, 4 mice/group). **(C)** Differentiated SH-SY5Y neuroblastoma cells were treated with brain-derived ExE-EVs for 4 days and KCl-induced depolarization was measured by fura 2-AM calcium assay, using a fluorescence microscope. Representative images (20x, scale bar 20 μm) illustrating the 350/380 fluorescence ratio before (baseline) and after (peak) KCl stimulation (55 mM) are shown. Quantification graph measuring the fluorescence ratio following KCl administration normalized to baseline fluorescence is shown on the right. Data represent mean values ± SEM. Differences were estimated using one-way ANOVA followed by Tukey’s post-hoc test.

Of note, the majority of the synaptic-related pathways, mainly in the group injected with WT/PFF exosomes, were characterized by negative IPA z-scores, suggestive of a predicted decreased activity. Moreover, CREB, G-Protein Coupled Receptor, Protein Kinase A, Axonal Guidance, Calcium, Dopamine and GABA Receptor signaling pathways, which are crucial for the maintenance of neuronal integrity and striatal homeostasis were also found to be regulated in both WT/PFF and KO/PFF ExE-EV-recipient groups. Overall, the impact of WT/PFF ExE-EVs on the transcriptome of host tissue, two-months post-injection, seems more profound than that of KO/PFF ExE-EVs, as reflected in both the number of DEGs (Fig. 5A) and the statistical significance of IPA canonical pathways (Fig. 5B). Given the above, we further sought to examine the impact of intrastriatal injections of ExE-EVs isolated from PFF-inoculated brains on synaptic integrity in WT host mice, at two-months post-injection. Striatal brain slices from ExE-EV-inoculated mice were co-stained with the postsynaptic density protein 95 (PSD-95) and the presynaptic marker synaptobrevin-2 (vesicle-associated membrane protein 2, VAMP2). As shown in Fig. 6A, B, although the levels of VAMP2 were not affected in any of the host groups, PSD-95 immunoreactivity exhibited a significant reduction only in mice receiving WT/PFF ExE-EVs. In agreement with the above, assessment of the distribution of the aforementioned synaptic markers revealed that only inoculation with WT/PFF ExE-EVs led to a significant decrease of PSD-95 / VAMP2 co-localization (Fig. 6A, B). To further investigate the impact of brain-derived ExE-EVs on synaptic function, we treated differentiated SH-SY5Y cells with the 4 different ExE-EV groups (WT/PBS, WT/PFF, KO/PBS, and KO/PFF). Following a four-day treatment, cells were loaded with the calcium-binding dye Fura-2AM and KCl-induced calcium entry was measured via calcium imaging. In the same line with our previous results, WT/PFF ExE-EVs impaired recipient cell response to KCl, as exhibited by the significant reduction of KCl-mediated calcium entry (Fig. 6C), suggestive of a perturbed depolarization capacity. Collectively, our data suggest that WT/PFF ExE-EVs have a negative effect at the level of the synapse, at a time point in which no significant neurotoxicity is observed.

## DISCUSSION

Over the years, exosome association with pathogenic proteins with seeding capacities, such as amyloid precursor protein [54, 56], α-Syn [17, 22, 57- 59], mutant huntingtin [60, 61], and scrapie isoform of the prion protein [62, 63], has been cemented. That, along with their innate ability to transfer their cargo from cell-to-cell, facilitating intercellular communication [8, 15], have attracted considerable interest in their interplay with the mechanisms underlying the spread of pathology in neurodegenerative disorders.

In this study, we explored the controversial role of exosomes in pathology progression and questioned whether the presence of endogenous α-Syn as exosomal cargo is necessary for the exosomes to acquire a pathogenic identity, following a toxic stimulus, such as PFFs, resulting in a prion-like spreading of α-Syn. In this respect, we isolated intact interstitial brain ExE-EVs from WT and α-Syn KO mice intrastriatally inoculated with mouse PFFs or PBS, at one-month post-injection. WT exosome-donor mice developed phospho-α-Syn pathology along the ipsilateral side of nigrostriatal axis and the ipsilateral cortical areas, with simultaneous accumulation of p62 and ubiquitin within the phospho-α-Syn bearing TH positive somata, mimicking LBs present in PD brains [36], [45]. The observed pathology was accompanied by pronounced gliosis in the ipsilateral *striatum* [26, 36, 45, 46]. In agreement with previous reports and our own work, dopaminergic cell death and behavioral motor deficits were not detected in these mice at the time point examined [19, 36, 64]. Isolating ExE-EVs at this time point allowed us to examine the effect of early pathogenic changes, preceding neuronal cell death, on disease transmission. Importantly, in order to assess whether PFF-induced pathology impacts the exosomal cargo irrespective of the presence of endogenous α-Syn, we also isolated ExE-EVs from α-Syn KO mice injected with PFFs. The latter did not exhibit phospho-α-Syn pathology or increased gliosis and have been shown to lack neurodegeneration [45, 65].

The isolated extracellular nanovesicles displayed cup-shaped morphology and size ranging mostly between 50-200 nm, were enriched in exosomal markers and exhibited increased AChE enzymatic assay, characteristic of exosomes. Importantly, proteomic analysis revealed that almost half of the identified cargo proteins were classified in the extracellular exosome GO, demonstrating a substantial overlap with known extracellular vesicle databases (Vesiclepedia, and ExoCarta), and that brain tissue was identified as the main source of their origin. Taken all together, we established that the isolated vesicular preparations meet the criteria of intact brain extracellular vesicles enriched in exosomes [8, 47, 48] and thus ensured that any ExE-EV-related phenotype could not be attributed to the heterogeneous starting material. Importantly, PFF injection did not alter the quantity, shape and size distribution of the isolated ExE-EVs in both WT and α-Syn KO background, in comparison to the respective controls. In agreement with these findings, Herman et al., did not detect any differences in the number of CSF-derived extracellular vesicles between PD and non-PD subjects [24]. Of note, exosomes purified from WT/PFF-injected mice contained increased levels of phospho-α-Syn, as well as monomeric and oligomeric forms of the protein, in comparison to the respective controls. This finding was further verified by proteomic analysis, which showed a significant induction of α-Syn expression in WT/PFF *versus* WT/PBS ExE-EVs. Similarly, we identified the presence of misfolded α-Syn species in ExE-EVs originating from the pathogenic environment of the PFF-injected WT mice. These results are clinically relevant given the fact that aggregate-prone proteins have been identified as exosomal cargo isolated from postmortem brain tissue of AD or DLB patients [22]. Notably and with respect to synucleinopathies, the levels of phosphorylated and total α-Syn, both monomeric and oligomeric, have been found to be elevated in exosomes isolated from the CSF [17], plasma [20], serum [21] or brain [22] of PD and DLB patients in comparison to non-neurological controls. Moreover, the absence of α-Syn from the ExE-EVs derived from the PFF-injected KO group suggests that the PFF inoculum is cleared in the brain at one-month post-injection. Therefore, the increased α-Syn levels detected in the WT/PFF ExE-EVs probably correspond to the accumulation of the endogenous protein.

Moreover, proteomic analysis of the brain-derived ExE-EVs, revealed that a similar number of proteins were differentially expressed in ExE-EVs isolated from PFF-inoculated mice in both WT and α-Syn KO backgrounds. IPA platform analysis of the significantly altered proteins revealed an overrepresentation of pathways related to phagosome formation, mitochondrial and synaptic functions, metabolism and inflammation. These pathways have been classified amongst the top regulated in a meta-analysis study, conducted by Kelly et al, who by identifying DEGs in published PD microarray datasets, performed pathway and protein-protein interaction analysis [51]. Notably, in our study, the majority of the differentially altered proteins followed an inverse pattern of regulation, between the two different backgrounds. This opposite pattern of expression could be correlated to the presence of α-Syn and LB-like pathology and pronounced gliosis in the PFF-injected WT mice. Based on the above, our study verified from a different scope that the presence of the endogenous α-Syn plays a critical role in the development of PD-like pathology, showing that the altered exosomal content upon PFF inoculation, reflects distinct cellular responses in the same pathological stimulus, which highly depend on the background. Our findings, are further supported by clinical data provided by transcriptomics meta-analysis [52], which clearly show a similar mode of regulation between PD genes and the top differentially altered proteins in mice injected with WT/PFF exosomes.

Remarkably, we were able to detect similarities between the identified proteomic cargo of the mouse brain-derived ExE-EVs and that of extracellular vesicles isolated in other studies either from sera [66, 67] or erythrocytes [68] of PD patients or control subjects. The highest overlap (39.5%) was observed following comparative analysis between our study and that of Lamontagne-Proulx *et al.*, with 297 common proteins out of the total exosomal proteome. Notably, amongst the significantly altered proteins in the WT/PFF ExE-EVs were the dihydropteridine reductase (QDPR), ATP synthase subunit alpha 1 (ATP5A1), Phosphoglycerate kinase 1 (PGK1), Solute carrier family 2 (SLC2A1), and the mitochondrial proteins Protein deglycase DJ-1 (DJ-1), Complement component 1 Q (C1QB1) and NADH-ubiquinone oxidoreductase (NDUFS1) (highlighted in Supplementary Table 4, WT-PFF vs WT-PBS), which are also differentially expressed in PD patient-derived exosomes [14], [66-68] and are related to metabolic, synaptic and mitochondrial functions. The above similarities between the PD patient-derived exosomes and the mouse WT/PFF ExE-EVs further strengthen our hypothesis and provide a valuable correlation of our model to the clinical condition.

Importantly, only WT/PFF ExE-EVs were able to induce phospho-α-Syn pathology in the ipsilateral *striatum* and *SNpc*, accompanied by increased astrogliosis in the ipsilateral *striatum*, at two-months post-injection, indicative of their capacity to transfer the pathology observed in the tissue of origin. Of note and with regards to the spreading capacity of ExE-EVs bearing misfolded α-Syn, we showed that WT mice inoculated with three times higher dose, exhibited more pronounced phospho-α-Syn pathology, in both ipsilateral *striatum* and *SNpc* which was immunoreactive for the conformation-specific antibody MJFR-14-6-4-2, verifying the fibrillar nature of the α-Syn aggregates. The observed intraneuronal inclusions were also characterized by accumulation of the LB-related markers p62 and ubiquitin, a result in agreement with other studies administering *in vivo* and *in vitro* higher doses of PD-derived extracellular vesicles [20, 21, 24]. The overall phenotype of WT mice injected with WT/PFF ExE-EVs lacked nigrostriatal degeneration and motor defects, which are manifested only after 20-30% of dopaminergic neurons are lost [64]. In accord with our results, there are reports showing that exosomes originating from an environment exhibiting α-Syn pathology, contribute to cell-to-cell spreading of the pathologic phenotype [18]. More specifically, Danzer et al. demonstrated that exosome-associated α-Syn is more effectively taken up by recipient cells and exerts a more potent neurotoxic effect compared to free α-Syn, using a well-established luciferase complementation assay [18]. In the same context, Xia *et al.* showed that exosome-associated α-Syn from PD patient blood plasma is first internalized by microglia cells, induces their activation and blocks autophagy, thus facilitating its re-secretion in the extracellular space and re-uptake by neighboring neurons [20]. Similarly, intravenous or intrastriatal administration of exosome-associated α-Syn from PD patient serum results in accumulation of phospho-α-Syn, p62, and ubiquitin in the *SNpc* and concomitant reduction of TH dopaminergic neurons, followed by motor deficits [21]. Consistently, α-Syn-containing brain exosomes isolated from DLB patients, when injected into the mouse hippocampus are taken up by neurons and astrocytes, mediating α-Syn pathology propagation [22]. Similarly, intranasal administration of extracellular vesicles isolated from CSF of PD patients led to the induction of α-Syn pathology in the dopaminergic neurons along the nigrostriatal axis and gliosis in the *striatum* of wild type mice [24]. Interestingly, the punctated pattern of α-Syn pathology presented in the above study was very similar to the one observed in our model, albeit the lack of dopaminergic neuronal demise and concomitant deficits in locomotion. This could be attributed to the different routes of administration, as well as the different time points in which the assessments were performed, given the fact that the amount of vesicles administered in both studies was very similar [24]. On the other hand, we have shown that naturally secreted free, rather than exosome-associated, α-Syn induces calcium deregulation when applied to neuronal cell lines and primary cortical neurons [43]. In addition, we have shown that intrastriatal inoculation of exosome-associated α-Syn isolated from symptomatic transgenic A53T mouse brains was not sufficient to induce seeding of the endogenous α-Syn in WT host mice [19]. These discrepancies may reside in different α-Syn species present in the exosomes. Indeed, it has been proposed that α-Syn exists and exerts its detrimental effects in different strains, leading to different aggregates that cause distinct synucleinopathies, including PD, DLB, and MSA [17], [69]. Finally, we sought to verify WT/PFF ExE-EV uptake in the inoculated mouse brain and correlate its tropism to the observed phospho-α-Syn pathology and gliosis. In this respect, DiI-labelled WT/PFF ExE-EVs were injected intrastriatally in WT mice and their homing was assessed at 2 weeks post injection. In agreement with previous studies, we demonstrated that DiI-labelled ExE-EVs were internalized by TUJ positive neuronal somata and TH dopaminergic axons, as well as microglia and astrocytes in the ipsilateral *striatum* [20], [21], [24], [70]-[74]. In this sense, both phospho-α-Syn pathology and gliosis could be attributed to the direct effect of aberrant α-Syn carrying ExE-EVs on the target cells. To this end, Guo and colleagues showed that following treatment with PFFs, microglia-derived exosomes propagated α-Syn pathology in neurons, a phenomenon partly alleviated via pharmacological inhibition of exosome biogenesis in microglia, pointing that exosomes were indeed carriers of the pathogenic entity [75]. In accord with this, our study shows that increased concentrations of WT/PFF ExE-EVs correlate with increased pathology in the host brain. However, we cannot exclude indirect effects of WT/PFF ExE-EVs in the induction of the observed pathology, such as exosome-induced inflammation.

Notably, RNA-seq analysis revealed that WT mice receiving ExE-EVs derived from PFF-injected mouse brains, irrespective of the background (WT or α-Syn KO) exhibited transcriptomic changes in the ipsilateral *striatum*, at two-months post-inoculation. Subsequent IPA analysis showed an overrepresentation of pathways related to synaptic function, including opioid signaling, Dopamine-DARP32 Feedback in cAMP signaling, synaptogenesis signaling, synaptic LTP and LTD, amongst others. Despite the aforementioned similarities, WT/PFF ExE-EVs had a greater impact on the transcriptome of host striatal tissue, reflected in the higher statistical significance of affected canonical pathways in comparison to the respective of KO/PFF ExE-EVs, as well as in the negative IPA z-scores characterizing the majority of the synaptic-related pathways, indicative of a predicted decreased activity. The effect of WT/PFF ExE-EVs at the level of synapsis was further verified by the significant reduction of both the post-synaptic marker PSD-95 and its co-localization with the pre-synaptic marker VAMP2 in the ipsilateral *striatum* of WT host mice. Consistently, differentiated SH-SY5Y cells treated with WT/PFF ExE-EVs, demonstrated reduced KCl-mediated calcium influx, further underscoring the perturbing abilities of WT/PFF ExE-EVs in synaptic function. No such alterations were observed in the other 3 ExE-EV-treated groups (WT/PBS, KO/PBS, KO/PFF). Synaptic dysfunction has been associated with central nervous system (CNS) neurological disorders and up until recently has been thought to be an endpoint result of the neurodegenerative process. However this notion has been challenged due to emerging evidence suggesting that perturbations in synaptic function may precede cellular loss and even trigger the pathogenesis in both neurodevelopmental and neurodegenerative diseases [76- 79]. This is in accord with our findings unraveling a deregulation at the level of synapsis, at a time point in which cellular demise is not observed. In agreement, Wu *et al*. have shown that treatment of primary hippocampal neurons with PFFs, resulted in the formation of misfolded α-Syn aggregates, which in turn compromised synaptic integrity, as exhibited by reduced co-localization of the presynaptic marker synapsin-1 with the PSD-95 post-synaptic marker [80]. However, these findings were not accompanied by reduction of the neuronal viability up to 7 days post PFF treatment [80]. In the same context, early hippocampal synaptic loss prior to overt amyloid beta plaque deposition was reported in the J20 transgenic mice, modeling AD, as illustrated by decreased levels of PSD-95 and its co-localization with synaptophysin, [81]. Moreover, Galli *et al.*, showed that mice deficient for Wnt signaling exhibited striatal synaptic degeneration in the absence of neuronal death or axonal retraction [82]. In the context of exosomes, one could argue that these nano-sized vesicles can facilitate such changes, given that they selectively carry intracellular components, that may regulate cellular responses [83] including synaptic plasticity [84, 85].

Overall, our work shows for the first time that toxic stimuli, such as PFFs, can impact the composition of the exosomal cargo, independent of the presence of endogenous α-Syn. However, the presence of endogenous α-Syn, following PFF intoxication, appears to be the rate-limiting factor for ExE-EVs to mediate α-Syn prion-like spreading in the host tissue. Moreover, the similarities between exosomes deriving from the PD mouse model described herein and the reported PD patient clinical specimens highlight the clinical relevance of this study. Albeit of the fundamental role of the endogenous α-Syn as exosomal cargo for disease transmission, we cannot not exclude the contribution of other exosomal components in disease progression, including mRNAs, miRNAs, lncRNAs, and circRNAs, which have not been investigated in this study. To conclude, our findings provide new knowledge on the biology of exosomes in the spreading of PD-related pathology. Intervening in the exosomal cargo and/or modifying it accordingly could pave the way for new therapeutic approaches to attenuate or even halt the dilapidating symptoms of PD and related synucleinopathies.

## Supplementary Materials

The Supplementary data can be found online at: www.aginganddisease.org/EN/10.14336/AD.2023.0614



## Data Availability

The proteomic data have been deposited to the ProteomeXchange Consortium via the PRIDE [40] partner repository with the dataset identifier PXD036301 (Username: reviewer_pxd036301@ebi.ac.uk;Password: UjMBXK83). Raw sequence reads for this study can be found in the Sequence Read Archive Database of National Center for Biotechnology Information (www.ncbi.nlm.nih.gov/sra), under the accession number PRJNA 847043. All other data needed to evaluate the conclusions in the paper are available in the paper or the Supplementary Materials. The raw data supporting the findings of this study will be made available by the authors, upon reasonable request.
