# Supplementary material for: Endogenous Alpha-Synuclein is Essential for the Transfer of Pathology by Exosome-Enriched Extracellular Vesicles, Following Inoculation with Preformed Fibrils in vivo
Source: Aging Dis. 2024 Apr 1;15(2):869–92. doi: 10.14336/AD.2023.0614 (PMC10917543; doi:10.14336/AD.2023.0614)
Supplement: Supplementary file 1 [file AD-15-2-869-s.pdf]

SUPPLEMENTARY DATA

Endogenous Alpha-Synuclein is Essential for the Transfer of Pathology by Exosome-Enriched Extracellular Vesicles, Following Inoculation with Preformed Fibrils *in vivo*

Katerina Melachroinou, Georgios Divolis, George Tsafaras, Mantia Karampetsou, Sotirios Fortis, Yannis Stratoulias, Gina Papadopoulou, Anastasios G. Kriebardis, Martina Samiotaki, Kostas Vekrellis

© 2023. Melachroinou K et al. Published online at http://www.aginganddisease.org/EN/10.14336/AD.2023.0614

SUPPLEMENTARY DATA

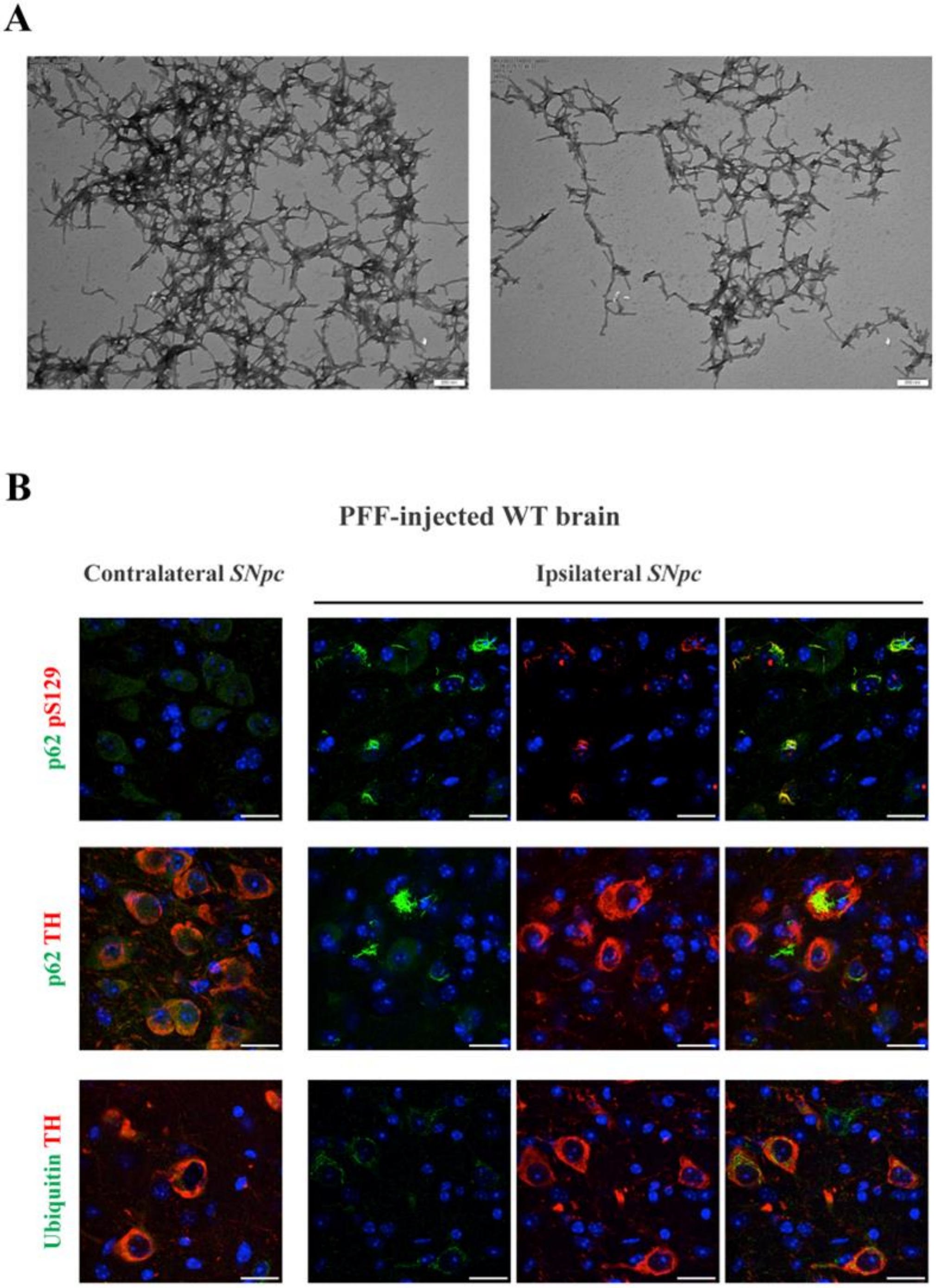

Supplementary Figure 1. Intrastriatal inoculation of PFFs results in the accumulation of p62 and ubiquitin in the dopaminergic neurons of the ipsilateral *SNpc*, one-month post-injection. (A) Negatively stained mouse PFFs visualized by TEM, verified the presence of α-Syn fibrillar forms that were injected in the right dorsal *striatum* (scale bar 200 μm). **(B)** De-convolved confocal images showing accumulation of either p62 or ubiquitin (green) within the TH (red) positive neurons and co-localization of accumulated p62 (green) and aggregated pS129 (5B9 clone, red) in the ipsilateral *SNpc* of WT mice injected with PFFs (63x lens, scale bar 20 μm). α-Syn: alpha-synuclein, PFFs: alpha-synuclein preformed fibrils, TEM: Transmission Electron Microscopy.

© 2023. Melachroinou K et al. Published online at http://www.aginganddisease.org/EN/10.14336/AD.2023.0614

SUPPLEMENTARY DATA

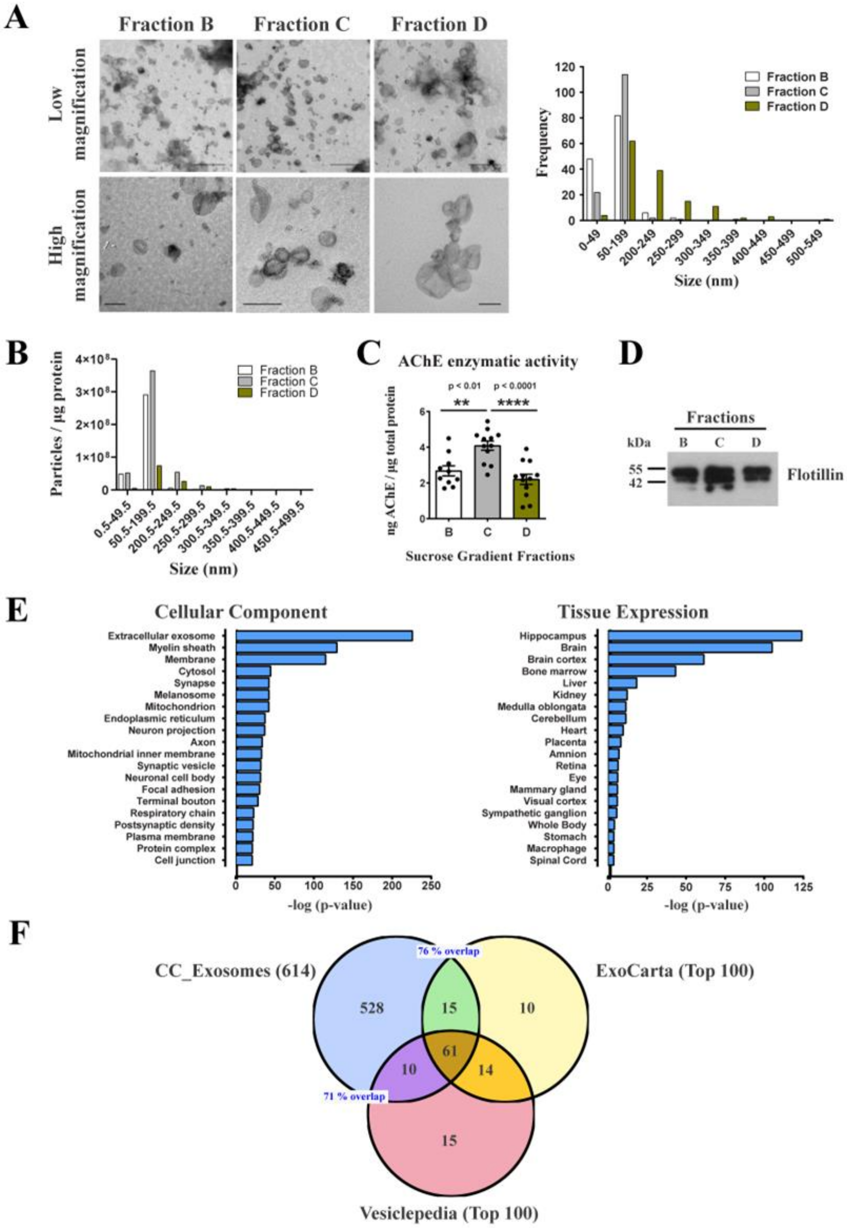

Supplementary Figure 2. Characterization of the brain-derived ExE-EVs, following papain-based enzymatic homogenization, sequential centrifugations and sucrose gradient purification. (**A**) Low (18,000x, scale bar 500 μm) and high magnification (34,000x, scale bar 200 μm, upper panel) EM images depicting the three sucrose gradient purified fractions, (B, C, and D) which are enriched in exosomes. Graph depicting the size distribution of extracellular vesicles isolated from the respective fractions. The x-axis represents the different size groups and the respective frequency is shown on the y-axis (lower panel). (**B**) Histogram illustrating the size distribution of the isolated brain-derived fractions, as measured by nanoparticle tracking analysis. y-axis represents the number of particles/μg of total protein of the different size groups shown along the x-axis. (**C**) Graph depicting the concentration of AChE enzyme (ng) contained in the isolated exosomal fractions normalized to the total protein content (μg) measured by Bradford assay. Data represent mean values ± SEM. Differences were estimated using one-way ANOVA followed by Tukey's post-hoc test, **p<0.01, and ***p<0.001 (n = 10-12 independent brain exosome preparations). (**D**) Representative Western immunoblot of the sucrose gradient purified fractions (B, C, D) against the exosome marker flotillin. (**E**) The brain-derived exosome-enriched fraction C was analyzed by nano LC-MS/MS, followed by DAVID platform analysis. Graphs depicting the cellular components (left) and the tissue specificity (right) along the y-axis and their statistical significance [-log (*P* value)] along the *x*-axis. Exosomes and brain tissue are the top hits following analysis with the DAVID platform. (**F**) Venn diagrams depicting numbers of the exosome-associated proteins identified in our preparations by DAVID in (blue), compared to the top 100 proteins of ExoCARTA (yellow) and Vesiclepedia (pink). The percentages indicate the overlap between the different data sets. ExE-EVs: Exosome Enriched Extracellular Vesicles, EM: Electron Microscopy, AChE: Acetylcholinesterase, nano LC-MS/MS: Nanoscale liquid chromatography coupled to tandem mass spectrometry.

© 2023. Melachroinou K et al. Published online at http://www.aginganddisease.org/EN/10.14336/AD.2023.0614

SUPPLEMENTARY DATA

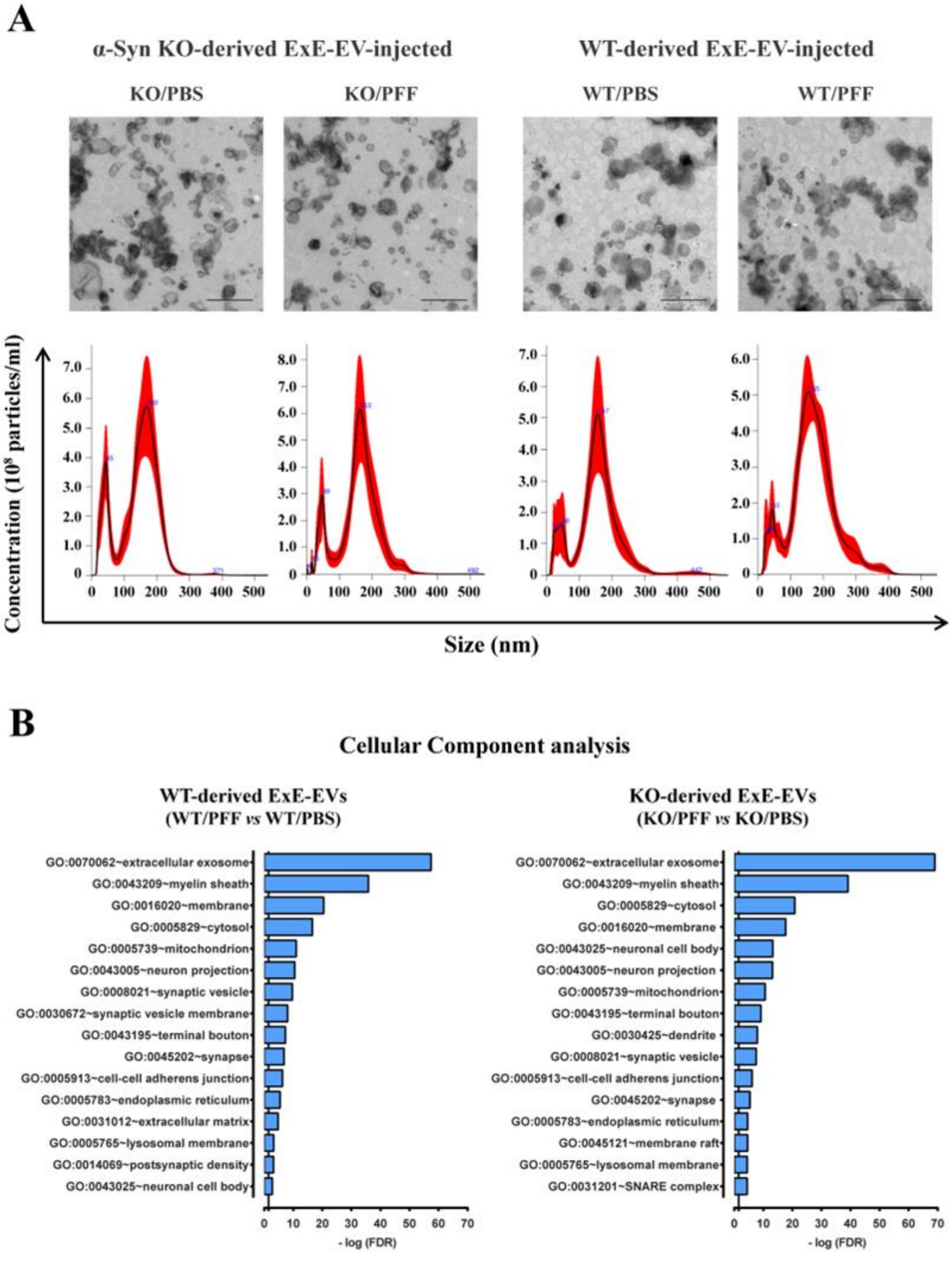

Supplementary Figure 3. Characterization of the ExE-EVs isolated from PFF-injected WT and α-Syn KO mouse brains, at one-month post-inoculation. (**A**) Representative wide-field EM images of exosome-enriched fractions C isolated from the PFF-injected WT and α-Syn KO mice and their respective PBS controls (scale bar 500 nm, upper panel). Representative curves of the size distribution of the isolated brain ExE-EVs from the 4 experimental groups, by nanoparticle tracking analysis (lower panel). (**B**) Cellular component analysis of the differentially altered proteins in the isolated brain ExE-EVs, following analysis with the DAVID platform. Graphs depicting the cellular components along the y-axis and their statistical significance [-log (*P* value)] along the *x*-axis, for ExE-EVs isolated from WT (left) and α-Syn KO (right) brains. WT: wild type, KO: knockout.

© 2023. Melachroinou K et al. Published online at http://www.aginganddisease.org/EN/10.14336/AD.2023.0614

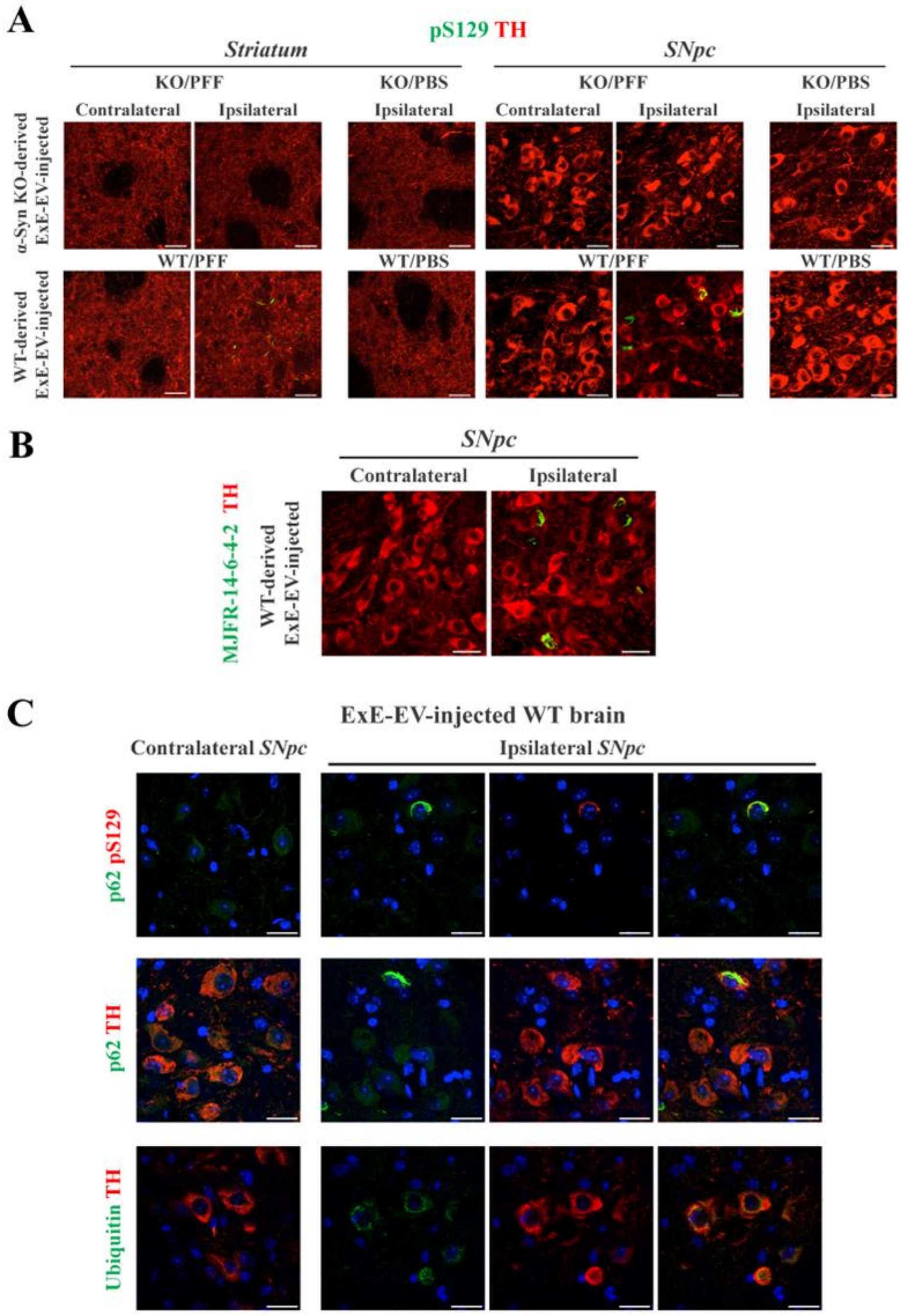

Supplementary Figure 4. Inoculation of WT mice with higher dose of aberrant α-Syn containing ExE-EVs triggers more robust pathology in the nigrostriatal route/axis at two-months post-injection. WT mice were sterotactically injected with 15 μg of brain-derived ExE-EVs in the right dorsal *striatum*. (**A**) Representative confocal images showing double immunostaining against phospho-α-Syn (green, pS129) and TH (red) in the contralateral and ipsilateral *striatum* (left panels) and *SNpc* (right panels) of ExE-EV-injected WT mice, [(63x, scale bar, 20 μm)]. (**B**) Representative double immunostaining against the conformation-specific α-Syn antibody MJFR-14-6-4-2 (green) and TH (red) in the *SNpc* of WT mice inoculated with WT/PFF-exosomes, showing the fibrillar nature of the α-Syn accumulations within the TH neurons (63x, scale bar, 20 μm). (**C**) Representative de-convolved confocal images depicting p62 or ubiquitin (green) accumulation within the TH (red) positive neurons and colocalization of accumulated p62 (green) and aggregated pS129 (5B9 clone, red) in the ipsilateral *SNpc* of WT/PFF-ExE-EEV-injected WT mice (63x lens, scale bar 20 μm). phospho-α-Syn: phosphorylated alpha-synuclein, TH: tyrosine hydroxylase, *SNpc*: *Substantia nigra pars compacta*.

© 2023. Melachroinou K et al. Published online at http://www.aginganddisease.org/EN/10.14336/AD.2023.0614

SUPPLEMENTARY DATA

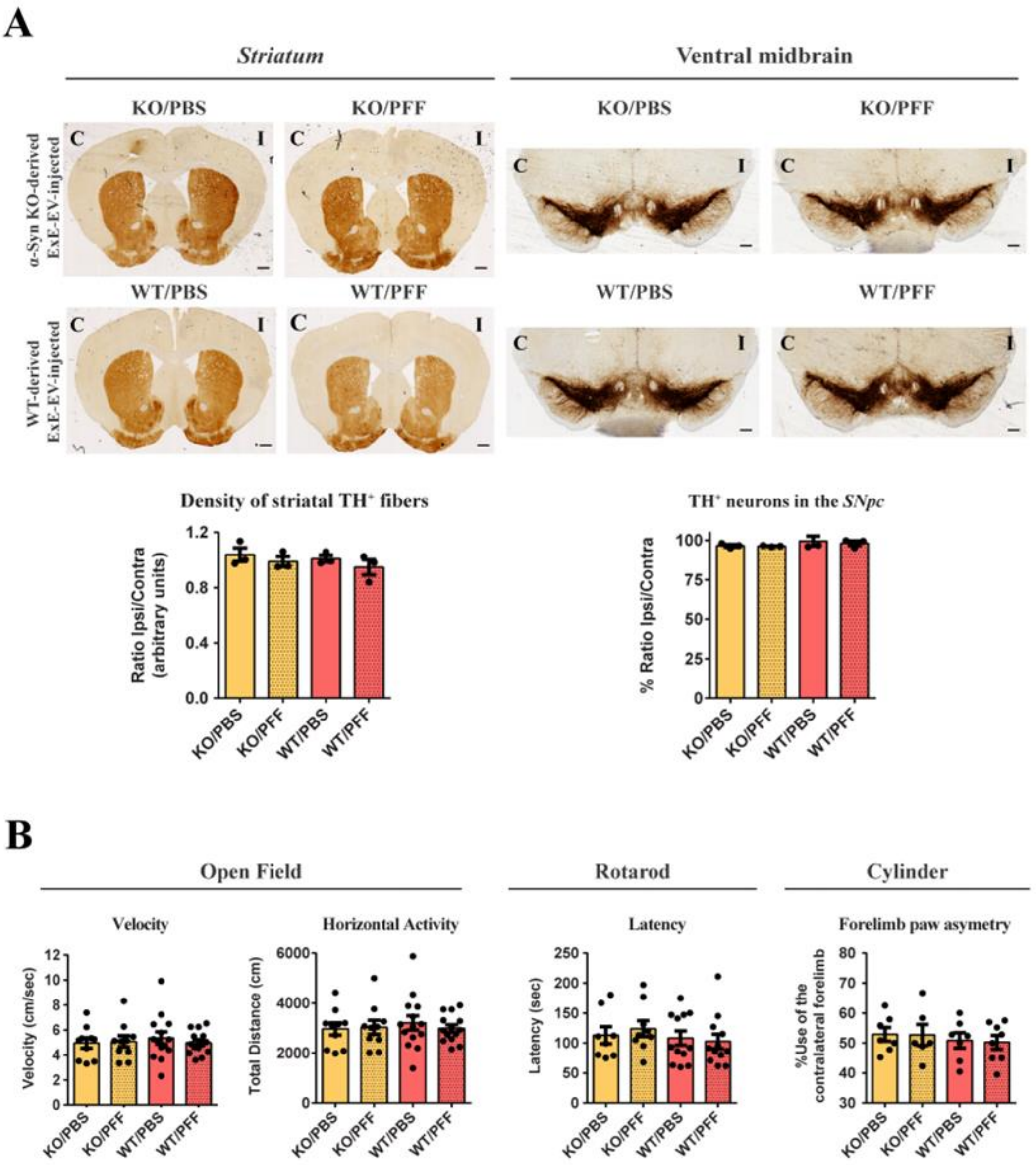

Supplementary Figure 5. Inoculation with brain-derived ExE-EVs does not significantly affect either nigrostriatal integrity or behavioral parameters in WT host mice at two-months post-injections, irrespective of the ExE-EV background. (**A**) Representative images of coronal striatal and nigral sections stained with anti-TH and obtained with the slide scanner (upper panels, scale bar 400 μm). Graphs depicting the densitometric analysis of the TH positive striatal fibers, presented as the ratio of ipsilateral to contralateral side (lower panel, left) and stereological counts of the nigral dopaminergic cell somata, estimated as the percentage of TH positive neurons of the ipsilateral side versus the ones found in the contralateral side (lower panel, right) (n = 3 animals per group). Data represent mean values ± SEM. Differences were estimated using Kruskal-Wallis followed by Dunn's test. (**B**) Brain ExE-EV-injected mice were subjected to open field, rotarod and cylinder tests, for assessment of their locomotor performance. Graphs depicting the mean velocity (cm/sec) and horizontal activity (total distance covered in cm) during open field test, the latency to fall during the rotarod test and the forelimb paw asymmetry, expressed as the percentage of use of the contralateral paw normalized to the ipsilateral, following cylinder test, are shown. Data represent mean values ± SEM. Differences were estimated using one-way ANOVA followed by Tukey's post-hoc test. (n = 7-14 animals per group). C: contralateral side, I: ipsilateral side.

© 2023. Melachroinou K et al. Published online at http://www.aginganddisease.org/EN/10.14336/AD.2023.0614

SUPPLEMENTARY DATA

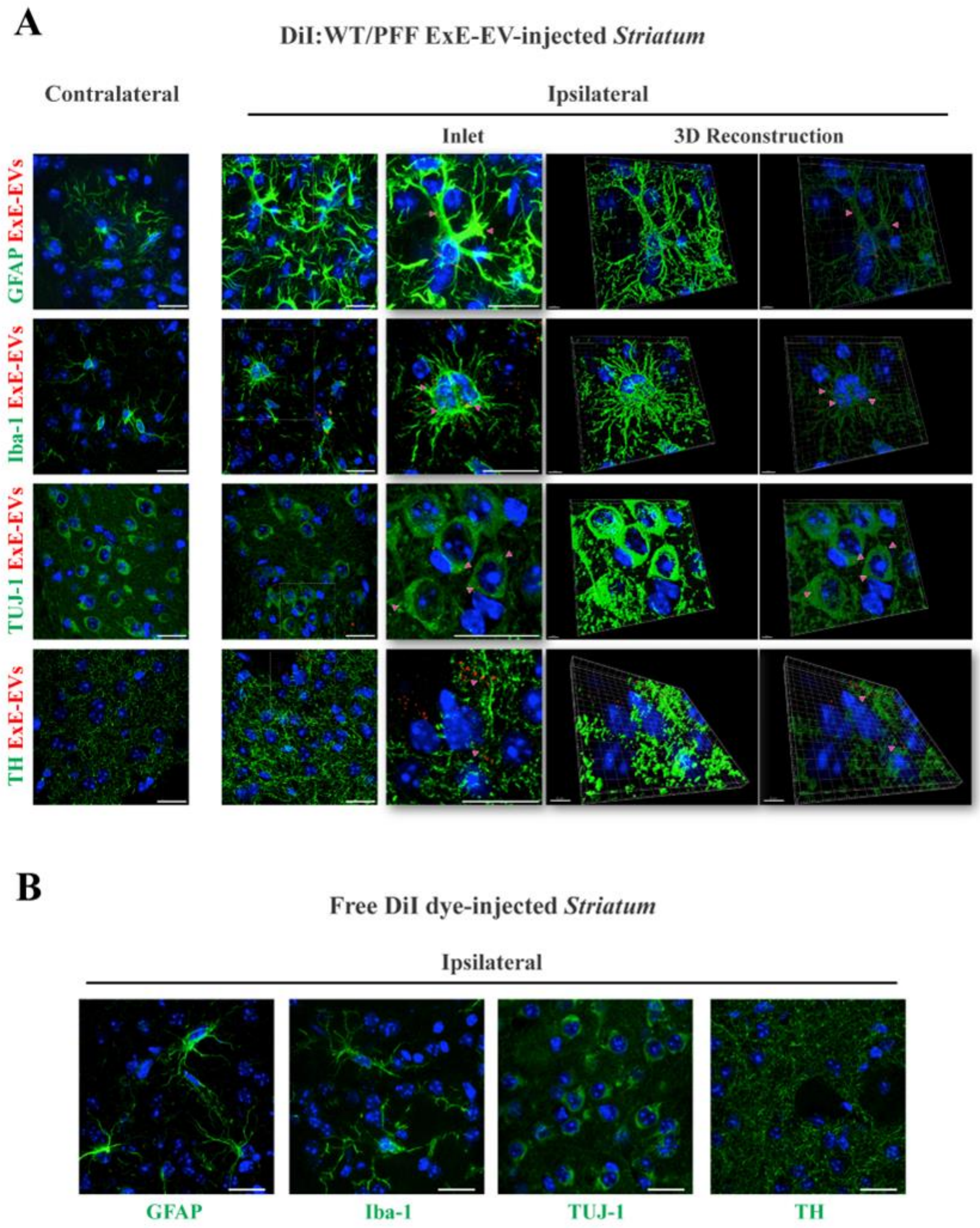

Supplementary Figure 6. DiI-labelled WT/PFF ExE-EVs injected in the right dorsal *striatum* are internalized by neurons, microglia and astrocytes, two-weeks post-injection. (**A**) Representative de-convolved confocal images showing the presence of DiI-labelled (red) WT/PFF ExE-EVs within astrocytes (GFAP, green), microglia (Iba-1, green), neurons (TUJ-1, green) and dopaminergic axons (TH, green) in the ipsilateral *striatum* of ExE-EV-injected WT mice [(63x, scale bar, 20 μm)]. Injections were performed with 5.5 μg of DiI-labelled WT/PFF ExE-EVs. The internalization of the injected ExE-EVs is further verified by 3D reconstruction via Imaris software, presented on the right panel [scale bar 5 μm (GFAP, Iba-1, TH) and 3 μm (TUJ-1)]. Arrows depict internalized ExE-EVs (**B**) Representative de-convolved confocal images. No free DiI dye was detected in any of the control-injected mice. DiI: 1,1'-Dioctadecyl-3,3,3',3'-Tetramethylindocarbocyanine Perchlorate ($DiIC_{18}(3)$).

© 2023. Melachroinou K et al. Published online at http://www.aginganddisease.org/EN/10.14336/AD.2023.0614

SUPPLEMENTARY DATA

Supplementary Table 1. Identified proteins.

C: Pfam	T: Protein IDs	T: Protein names	T: Gene names
GoLoco;Rap_GAP	A2ALS5;A2ALS5-2;A2ALS5-3;A2ALS5-4;Q5SVL6;Q5SVL6-2	Rap1 GTPase-activating protein 1	Rap1gap
C2;EF-hand_7;EF-hand_like;PI-PLC-X;PI-PLC-Y	A2AP18;A2AP18-2;A2AP18-3;A2AP18-4;A2AP18-5;Q4KWH5;Q4KWH5-5;Q4KWH5-4;Q4KWH5-3;Q4KWH5-2	1-phosphatidylinositol 4,5-bisphosphate phosphodiesterase eta-2	Plch2
Dpy19	A6X919;A6X919-2;P0CW70	Probable C-mannosyltransferase DPY19L1	Dpy19l1
fn3;I-set;Y_phosphatase	B0V2N1-4;B0V2N1-3;B0V2N1;B0V2N1-6;B0V2N1-2;B0V2N1-5	Receptor-type tyrosine-protein phosphatase S	Ptprs
DUF3451;Ion_trans;Na_trans_assoc	B1AWN6;Q9ER60	Sodium channel protein	Scn2a1
DOMON	B1AXV0	DOMON domain-containing protein FRRS1L	Frrs1l
G-alpha	B2RSH2	Guanine nucleotide-binding protein G(i) subunit alpha-1	Gnai1
Plexin_cytopl;PSI;Sema;TIG	B2RXS4	Plexin-B2	Plxnb2
Carb_anhydrase;fn3;Y_phosphatase	B9EKR1	Receptor-type tyrosine-protein phosphatase zeta	Ptprz1
	C0HK80;C0HK79		
WSK	D3YVF0	A-kinase anchor protein 5	Akap5
Ank_2;Glutaminase	D3Z7P3;D3Z7P3-2	Glutaminase kidney isoform, mitochondrial	Gls
Dispanin	E9PUL5	Proline-rich transmembrane protein 2	Prrt2
PI3_PI4_kinase;PI3Ka	E9Q3L2		Pi4ka
Ins145_P3_rec;Ion_trans;MIR;RIH_assoc;RR_TM4-6;RYDR_ITPR;RyR;SPRY	E9Q401;E9PZQ0;A2AGL3;A2AGL3-2	Ryanodine receptor 2	Ryr2
	E9Q6P5		Ttc7b
Laminin_G_2;Syndecan	E9Q7X7		Nrxn2
Arm	E9Q912;E9Q912-3		Rap1gds1
C2;DUF3498;RasGAP	F6SEU4	Ras/Rap GTPase-activating protein SynGAP	Syngap1
ATP_Ca_trans_C;Cation_ATPase_C;Cation_ATPase_N;E1-E2_ATPase;Hydrolase	G5E829	Plasma membrane calcium-transporting ATPase 1	Atp2b1
Calpain_III;Peptidase_C2	O08529	Calpain-2 catalytic subunit	Capn2
7tm_1	O08530	Sphingosine 1-phosphate receptor 1	S1pr1
Cache_1;VGCC_alpha2;VWA;VWA_N	O08532-5;O08532-4;O08532;O08532-2;O08532-3	Voltage-dependent calcium channel subunit alpha-2/delta-1;Voltage-dependent calcium channel subunit alpha-2-1;Voltage-dependent calcium channel subunit delta-1	Cacna2d1
BAR;SH3_9	O08539;O08539-2	Myc box-dependent-interacting protein 1	Bin1
Longin;Synaptobrevin	O08547	Vesicle-trafficking protein SEC22b	Sec22b
Amidohydro_1	O08553	Dihydropyrimidinase-related protein 2	Dpysl2
Clathrin_lg_ch	O08585	Clathrin light chain A	Clta
Sec1	O08599	Syntaxin-binding protein 1	Stxbp1
Sec1	O08599-2	Syntaxin-binding protein 1	Stxbp1

© 2023. Melachroinou K et al. Published online at http://www.aginganddisease.org/EN/10.14336/AD.2023.0614

SUPPLEMENTARY DATA

C2;Calpain_III;Peptidase_C2	O08688	Calpain-5	Capn5
1-cysPrx_C;AhpC-TSA	O08709	Peroxiredoxin-6	Prdx6
Pyr_redox;Pyr_redox_2;Pyr_redox_dim	O08749	Dihydrolipoyl dehydrogenase, mitochondrial	Dld
CAP_GLY;Dynactin	O08788;O08788-2	Dynactin subunit 1	Dctn1
EF-hand_5;PRKCSH;PRKCSH-like	O08795-2;O08795	Glucosidase 2 subunit beta	Prkcsh
1-cysPrx_C;AhpC-TSA	O08807	Peroxiredoxin-4	Prdx4
Band_7	O08917	Flotillin-1	Flot1
Ras	O08989	Ras-related protein M-Ras	Mras
PDZ	O08992	Syntenin-1	Sdcbp
HMA	O08997	Copper transport protein ATOX1	Atox1
Proteasome	O09061	Proteasome subunit beta type-1	Psmb1
ESSS	O09111	NADH dehydrogenase [ubiquinone] 1 beta subcomplex subunit 11, mitochondrial	Ndufb11
MARVEL	O09117-2;O09117	Synaptophysin-like protein 1	Sypl1
MARVEL	O09198-2;O09198	Myelin and lymphocyte protein	Mal
Amidohydro_1	O35098;O35098-2	Dihydropyrimidinase-related protein 4	Dpysl4
CD36	O35114	Lysosome membrane protein 2	Scarb2
Band_7	O35129	Prohibitin-2	Phb2
fn3;I-set	O35136;O35136-2	Neural cell adhesion molecule 2	Ncam2
MIF	O35215	D-dopachrome decarboxylase	Ddt
CRAL_TRIO;Y_phosphatase	O35239	Tyrosine-protein phosphatase non-receptor type 9	Ptpn9
LRR_8	O35381;Q64G17;Q9EST5;Q9EST5-2	Acidic leucine-rich nuclear phosphoprotein 32 family member A;Putative acidic leucine-rich nuclear phosphoprotein 32 family member C	Anp32a;Anp32c
	O35405	Phospholipase D3	Pld3
PA;Peptidase_M28;TFR_dimer	O35409	Glutamate carboxypeptidase 2	Folh1
EGF;F5_F8_type_C	O35474	EGF-like repeat and discoidin I-like domain-containing protein 3	Edil3
SNARE;Syntaxin	O35526;Q00262	Syntaxin-1A	Stx1a
Tetraspannin	O35566	CD151 antigen	Cd151
Disintegrin;Pep_M12B_propep	O35598	Disintegrin and metalloproteinase domain-containing protein 10	Adam10
Patched	O35604	Niemann-Pick C1 protein	Npc1
Aa_trans	O35633;O35633-2	Vesicular inhibitory amino acid transporter	Slc32a1
Annexin	O35639	Annexin A3	Anxa3
Adaptin_N;Alpha_adaptinC2;B2-adapt-app_C	O35643	AP-1 complex subunit beta-1	Ap1b1
MAM33	O35658	Complement component 1 Q subcomponent-binding protein, mitochondrial	C1qbp

© 2023. Melachroinou K et al. Published online at http://www.aginganddisease.org/EN/10.14336/AD.2023.0614

SUPPLEMENTARY DATA

C2	O35681;Q9R0N9	Synaptotagmin-3	Syt3
MARVEL	O35682	Myeloid-associated differentiation marker	Myadm
Serpin	O35684	Neuroserpin	Serpini1
CS;Nudc_N	O35685	Nuclear migration protein nudC	Nudc
JAB	O35864	COP9 signalosome complex subunit 5	Cops5
SDF	O35874	Neutral amino acid transporter A	Slc1a4
EF-hand_7	O35887	Calumenin	Calu
DDOST_48kD	O54734	Dolichyl-diphosphooligosaccharide--protein glycosyltransferase 48 kDa subunit	Ddost
Adaptin_N;BLVR	O54774	AP-3 complex subunit delta-1	Ap3d1
DEP;G-gamma;RGS	O54829	Regulator of G-protein signaling 7	Rgs7
Guanylate_cyc;HNOB;HNOBA	O54865	Guanylate cyclase soluble subunit beta-1	Gucy1b3
ig	O54901	OX-2 membrane glycoprotein	Cd200
OCD_Mu_crystall	O54983	Ketimine reductase mu-crystallin	Crym
ArsA_ATPase	O54984	ATPase Asna1	Asna1
Prominin	O54990;O54990-4;O54990-5;O54990-6;O54990-3;O54990-2	Prominin-1	Prom1
F5_F8_type_C;Laminin_G_2	O54991	Contactin-associated protein 1	Cntnap1
Inositol_P	O55023;P29218;P29218-3	Inositol monophosphatase 1	Impa1
GDA1_CD39	O55026;O55026-2	Ectonucleoside triphosphate diphosphohydrolase 2	Entpd2
Synuclein	O55042;O55042-2	Alpha-synuclein	Snca
AKAP7_NLS;AKAP7_RIRII_bdg	O55074;Q7TN79	A-kinase anchor protein 7 isoform alpha;A-kinase anchor protein 7 isoform gamma	Akap7
MARVEL	O55100;O55100-2	Synaptogyrin-1	Syngr1
NIPSNAP	O55125	Protein NipSnap homolog 1	Nipsnap1
NIPSNAP	O55126	Protein NipSnap homolog 2	Gbas
Septin	O55131;Q8C650;Q9DA97;Q8C650-2	Septin-7	Septin7
Cation_ATPase_C;Cation_ATPase_N;E1-E2_ATPase;Hydrolase	O55143;O55143-2;Q8R429	Sarcoplasmic/endoplasmic reticulum calcium ATPase 2	Atp2a2
Proteasome	O55234	Proteasome subunit beta type-5	Psmb5
ERG2_Sigma1R	O55242;O55242-2	Sigma non-opioid intracellular receptor 1	Sigmar1
Glycos_transf_2	O70152	Dolichol-phosphate mannosyltransferase subunit 1	Dpm1
PIP5K	O70172	Phosphatidylinositol 5-phosphate 4-kinase type-2 alpha	Pip4k2a
E1-E2_ATPase;Hydrolase	O70228;P98195;P98195-2	Probable phospholipid-transporting ATPase IIA	Atp9a
4_1_CTD;FA;FERM_C;FERM_M;FERM_N;SAB	O70318	Band 4.1-like protein 2	Epb41l2
GSHPx	O70325;O70325-2;O70325-3	Phospholipid hydroperoxide glutathione peroxidase, mitochondrial	Gpx4
UPF0172	O70378	ER membrane protein complex subunit 8	Emc8

© 2023. Melachroinou K et al. Published online at http://www.aginganddisease.org/EN/10.14336/AD.2023.0614

SUPPLEMENTARY DATA

Proteasome;Proteasome_A_N	O70435	Proteasome subunit alpha type-3	Psma3
G-alpha	O70443	Guanine nucleotide-binding protein G(z) subunit alpha	Gnaz
adh_short	O70503	Very-long-chain 3-oxoacyl-CoA reductase	Hsd17b12
Guanylate_kin;L27;PDZ;Pkinase;SH3_2	O70589;O70589-3;O70589-4;O70589-5;O70589-2	Peripheral plasma membrane protein CASK	Cask
fn3;Ldl_recept_a;Ldl_recept_b	O88307	Sortilin-related receptor	Sorl1
WD40	O88342	WD repeat-containing protein 1	Wdr1
Band_3_cyto;HCO3_cotransp	O88343	Electrogenic sodium bicarbonate cotransporter 1	Slc4a4
V-SNARE	O88384	Vesicle transport through interaction with t-SNAREs homolog 1B	Vti1b
fn3;ig	O88507	Ciliary neurotrophic factor receptor subunit alpha	Cntfr
Palm_thioest	O88531	Palmitoyl-protein thioesterase 1	Ppt1
RRM_1	O88569;O88569-3;O88569-2	Heterogeneous nuclear ribonucleoproteins A2/B1	Hnrnpa2b1
PBD;Pkinase	O88643	Serine/threonine-protein kinase PAK 1	Pak1
cNMP_binding;Ion_trans;Ion_trans_N	O88703;O88705	Potassium/sodium hyperpolarization-activated cyclic nucleotide-gated channel 2	Hcn2
Iso_dh	O88844	Isocitrate dehydrogenase [NADP] cytoplasmic	Idh1
Synapsin;Synapsin_C;Synapsin_N	O88935;O88935-1;O88935-3	Synapsin-1	Syn1
L27;PDZ	O88952	Protein lin-7 homolog C	Lin7c
Glucosamine_iso	O88958	Glucosamine-6-phosphate isomerase 1	Gnpda1
Noelin-1;OLF	O88998;O88998-3;O88998-2;O88998-4	Noelin	Olfm1
Peptidase_M14	O89001	Carboxypeptidase D	Cpd
DUF1899;DUF1900;WD40	O89053	Coronin-1A	Coro1a
LANC_like	O89112	LanC-like protein 1	Lancl1
Cytochrom_B_C;Cytochrom_B_N_2	P00158;Q7YC63	Cytochrome b	Mt-Cyb
COX1	P00397;P41293	Cytochrome c oxidase subunit 1	Mtco1
COX2;COX2_TM	P00405;P41294	Cytochrome c oxidase subunit 2	Mtco2
Pribosyltran	P00493	Hypoxanthine-guanine phosphoribosyltransferase	Hprt1
Carb_anhydrase	P00920	Carbonic anhydrase 2	Ca2
ig	P01831	Thy-1 membrane glycoprotein	Thy1
Globin	P01942	Hemoglobin subunit alpha	Hba
Globin	P02088	Hemoglobin subunit beta-1	Hbb-b1
Oxidored_q1;Oxidored_q5_N	P03911	NADH-ubiquinone oxidoreductase chain 4	Mtnd4
NADH5_C;Oxidored_q1;Oxidored_q1_N	P03921;P41299	NADH-ubiquinone oxidoreductase chain 5	Mtnd5
Myelin_MBP	P04370-4;P04370-5;P04370-10;P04370-9;P04370-6;P04370-13;P04370-7;P04370-11;P04370;P04370-8;P04370-14;P04370-2	Myelin basic protein	Mbp
Prion;Prion_bPrPp	P04925	Major prion protein	Prnp

© 2023. Melachroinou K et al. Published online at http://www.aginganddisease.org/EN/10.14336/AD.2023.0614

SUPPLEMENTARY DATA

Glycolytic	P05063	Fructose-bisphosphate aldolase C	Aldoc
Glycolytic	P05064	Fructose-bisphosphate aldolase A	Aldoa
Pkinase	P05132;P05132-2	cAMP-dependent protein kinase catalytic subunit alpha	Prkaca
Aminotran_1_2	P05201	Aspartate aminotransferase, cytoplasmic	Got1
Aminotran_1_2	P05202	Aspartate aminotransferase, mitochondrial	Got2
Tubulin;Tubulin_C	P05213	Tubulin alpha-1B chain	Tuba1b
Pkinase_Tyr;PSI;Sema;SH2;SH3_1;SH3_9;TIG			
FG-GAP;Integrin_alpha;Integrin_alpha2;VWA	P05555-2;P05555	Integrin alpha-M	Itgam
Ldh_1_C;Ldh_1_N	P06151;P00342	L-lactate dehydrogenase A chain	Ldha
PGI	P06745;CON__Q3ZBD7	Glucose-6-phosphate isomerase	Gpi
malic;Malic_M	P06801	NADP-dependent malic enzyme	Me1
IQ;Neuromodulin;Neuromodulin_N	P06837	Neuromodulin	Gap43
FOLN;Kazal_1;SPARC_Ca_bdg	P07214	SPARC	Sparc
Transthyretin	P07309	Transthyretin	Ttr
Annexin	P07356	Annexin A2	Anxa2
Serum_albumin	P07724	Serum albumin	Alb
Serpin	Q00896;P07758	Alpha-1-antitrypsin 1-3;Alpha-1-antitrypsin 1-1	Serpina1c;Serpina1a
Serpin	P07759;Q03734	Serine protease inhibitor A3K	Serpina3k
HATPase_c;HSP90	P07901;REV__Q8BL66	Heat shock protein HSP 90-alpha	Hsp90aa1
Thioredoxin	P08003	Protein disulfide-isomerase A4	Pdia4
HSP90	P08113	Endoplasmin	Hsp90b1
Apolipoprotein	P08226	Apolipoprotein E	Apoe
Sod_Cu	P08228	Superoxide dismutase [Cu-Zn]	Sod1
Ldh_1_C;Ldh_1_N	P08249	Malate dehydrogenase, mitochondrial	Mdh2
G-alpha	P08752;P20612;P50149;Q3V3I2	Guanine nucleotide-binding protein G(i) subunit alpha-2	Gnai2
EGF_2;Integrin_b_cyt;Integrin_B_tail;Integrin_beta	P09055;P09055-2	Integrin beta-1	Itgb1
Thioredoxin	P09103	Protein disulfide-isomerase	P4hb
Alk_phosphatase	P09242	Alkaline phosphatase, tissue-nonspecific isozyme	Alpl
PGK	P09411;P09041	Phosphoglycerate kinase 1	Pgk1
Ferritin	P09528	Ferritin heavy chain;Ferritin heavy chain, N-terminally processed	Fth1
Sod_Fe_C;Sod_Fe_N	P09671	Superoxide dismutase [Mn], mitochondrial	Sod2
Snf7	P0C0A3	Charged multivesicular body protein 6	Chmp6
I-set;LRR_1;LRR_5;LRR_8;LRRNT	P0C192	Leucine-rich repeat-containing protein 4B	Lrrc4b
Lectin_C	P0C7M9	C-type lectin domain family 2 member L	Clec2l

© 2023. Melachroinou K et al. Published online at http://www.aginganddisease.org/EN/10.14336/AD.2023.0614

SUPPLEMENTARY DATA

Ribosomal_L40e;Ribosomal_S27;ubiquitin	P0CG50;P0CG49;P62983;P62984	Polyubiquitin-C;Ubiquitin;Ubiquitin-related 1;Ubiquitin-related 2;Polyubiquitin-B;Ubiquitin;Ubiquitin-40S ribosomal protein S27a;Ubiquitin;40S ribosomal protein S27a;Ubiquitin-60S ribosomal protein L40;Ubiquitin;60S ribosomal protein L40	Ubc;Ubb;Rps27a; Uba52
EF-hand_7	P0DP28;P0DP27;P0DP26;Q9D6P8;P20801		
	P0DP60		
GTP_EFTU;GTP_EFTU_D2;GTP_EFTU_D3	P10126	Elongation factor 1-alpha 1	Eef1a1
Peptidase_C1;Propeptide_C1	P10605	Cathepsin B;Cathepsin B light chain;Cathepsin B heavy chain	Ctsb
DEAD;Helicase_C	P10630-2;P10630;P60843	Eukaryotic initiation factor 4A-II;Eukaryotic initiation factor 4A-II, N-terminally processed;Eukaryotic initiation factor 4A-I	Eif4a2;Eif4a1
Tubulin-binding	P10637-5;P10637;P10637-2;P10637-6;P10637-4;P10637-3	Microtubule-associated protein tau	Mapt
Thioredoxin	P10639	Thioredoxin	Txn
GST_C;GST_N	P10649;P19639;O35660	Glutathione S-transferase Mu 1	Gstm1
Alpha-amylase	P10852;P10852-2	4F2 cell-surface antigen heavy chain	Slc3a2
PC4	P11031	Activated RNA polymerase II transcriptional coactivator p15	Sub1
Lipocalin	P11404	Fatty acid-binding protein, heart	Fabp3
Lamp	P11438	Lysosome-associated membrane glycoprotein 1	Lamp1
HATPase_c;HSP90	P11499	Heat shock protein HSP 90-beta	Hsp90ab1
Bravo_FIGEY;fn3;I-set	P11627	Neural cell adhesion molecule L1	L1cam
CaMKII_AD;Pkinase	P11798;P11798-2	Calcium/calmodulin-dependent protein kinase type II subunit alpha	Camk2a
Ins145_P3_rec;Ion_trans;MIR;RIH_assoc;RYDR_ITPR	P11881;P11881-2;P11881-8;P11881-7;P11881-6;P11881-5;P11881-4;P11881-3;Q9Z329;Q9Z329-2;Q9Z329-3	Inositol 1,4,5-trisphosphate receptor type 1	Itpr1
Cpn60_TCP1	P11983;P11983-2	T-complex protein 1 subunit alpha	Tcp1
APP_amyloid;APP_Cu_bd;APP_E2;APP_N;Beta-APP;Kunitz_BPTI	P12023;P12023-2;P12023-3	Amyloid beta A4 protein;N-APP;Soluble APP-alpha;Soluble APP-beta;C99;Beta-amyloid protein 42;Beta-amyloid protein 40;C83;P3(42);P3(40);C80;Gamma-secretase C-terminal fragment 59;Gamma-secretase C-terminal fragment 57;Gamma-secretase C-terminal fragment 50;C31	App
cNMP_binding;RIIa	P12367	cAMP-dependent protein kinase type II-alpha regulatory subunit	Prkar2a
PFK	P12382	ATP-dependent 6-phosphofructokinase, liver type	Pfkl

© 2023. Melachroinou K et al. Published online at http://www.aginganddisease.org/EN/10.14336/AD.2023.0614

SUPPLEMENTARY DATA

EF-hand_6;EF-hand_7	P12658	Calbindin	Calb1
COX5A	P12787	Cytochrome c oxidase subunit 5A, mitochondrial	Cox5a
EF-hand_1;EF-hand_6	P12815;P12815-2	Programmed cell death protein 6	Pdcd6
fn3;I-set	P12960	Contactin-1	Cntn1
fn3;I-set	P13595;P13595-3;P13595-4	Neural cell adhesion molecule 1	Ncam1
NAD_Gly3P_dh_C;NAD_Gly3P_dh_N	P13707	Glycerol-3-phosphate dehydrogenase [NAD(+)], cytoplasmic	Gpd1
Na_K-ATPase	P14094	Sodium/potassium-transporting ATPase subunit beta-1	Atp1b1
C1q;Collagen	P14106	Complement C1q subcomponent subunit B	C1qb
Ldh_1_C;Ldh_1_N	P14152	Malate dehydrogenase, cytoplasmic	Mdh1
Calreticulin	P14211	Calreticulin	Calr
Na_K-ATPase	P14231	Sodium/potassium-transporting ATPase subunit beta-2	Atp1b2
Annexin	P14824	Annexin A6	Anxa6
MAP1B_neuraxin	P14873	Microtubule-associated protein 1B;MAP1B heavy chain;MAP1 light chain LC1	Map1b
Gln-synt_C;Gln-synt_N	P15105	Glutamine synthetase	Glul
Cadherin;Cadherin_C;Cadherin_pro	P15116	Cadherin-2	Cdh2
I-set;LRR_8;LRRNT;Pkinase_Tyr	P15209;P15209-4;P15209-3;P15209-2	BDNF/NT-3 growth factors receptor	Ntrk2
NDK	P15532	Nucleoside diphosphate kinase A	Nme1
Ldh_1_C;Ldh_1_N	P16125	L-lactate dehydrogenase B chain	Ldhb
CNPase	P16330;P16330-2	2,3-cyclic-nucleotide 3-phosphodiesterase	Cnp
BTB_2;Ion_trans	P16388;Q61923;B2RQA1	Potassium voltage-gated channel subfamily A member 1	Kcna1
EF-hand_7;EFhand_Ca_insen;SH3_1;Spectrin	P16546;P16546-2	Spectrin alpha chain, non-erythrocytic 1	Sptan1
HSP70	P16627	Heat shock 70 kDa protein 1-like	Hspa1l
Gp_dh_C;Gp_dh_N	P16858;Q64467	Glyceraldehyde-3-phosphate dehydrogenase	Gapdh
Lamp	P17047;P17047-3;P17047-2	Lysosome-associated membrane glycoprotein 2	Lamp2
Enolase_C;Enolase_N	P17182	Alpha-enolase	Eno1
Enolase_C;Enolase_N	P17183	Gamma-enolase	Eno2
Adaptin_N;Alpha_adaptin_C;Alpha_adaptinC2	P17426;P17426-2	AP-2 complex subunit alpha-1	Ap2a1
Adaptin_N;Alpha_adaptin_C;Alpha_adaptinC2	P17427	AP-2 complex subunit alpha-2	Ap2a2
COX7C	P17665	Cytochrome c oxidase subunit 7C, mitochondrial	Cox7c
Hexokinase_1;Hexokinase_2	P17710;P17710-3;P17710-4;P17710-2;O08528;Q91W97;Q3TRM8	Hexokinase-1	Hk1
Pro_isomerase	P17742	Peptidyl-prolyl cis-trans isomerase A;Peptidyl-prolyl cis-trans isomerase A, N-terminally processed	Ppia
TIM	P17751;P17751-2	Triosephosphate isomerase	Tpi1

SUPPLEMENTARY DATA

Sugar_tr	P17809	Solute carrier family 2, facilitated glucose transporter member 1	Slc2a1
Y_phosphatase	P18052;P18052-2	Receptor-type tyrosine-protein phosphatase alpha	Ptpra
A1_Propeptide;Asp	P18242	Cathepsin D	Ctsd
I-set	P18572;P18572-2	Basigin	Bsg
Cofilin_ADF	P18760	Cofilin-1	Cfl1
G-alpha	P18872	Guanine nucleotide-binding protein G(o) subunit alpha	Gnao1
G-alpha	P18872-2	Guanine nucleotide-binding protein G(o) subunit alpha	Gnao1
Acyl_transf_1;ADH_zinc_N;ketoacyl-synt;Ketoacyl-synt_C;KR;Methyltransf_12;PP-binding;PS-DH;Thioesterase	P19096	Fatty acid synthase;[Acyl-carrier-protein] S-acetyltransferase;[Acyl-carrier-protein] S-malonyltransferase;3-oxoacyl-[acyl-carrier-protein] synthase;3-oxoacyl-[acyl-carrier-protein] reductase;3-hydroxyacyl-[acyl-carrier-protein] dehydratase;Enoyl-[acyl-carrier-protein] reductase;Oleoyl-[acyl-carrier-protein] hydrolase	Fasn
GST_C;GST_N	P19157;P46425	Glutathione S-transferase P 1;Glutathione S-transferase P 2	Gstp1;Gstp2
COX5B	P19536	Cytochrome c oxidase subunit 5B, mitochondrial	Cox5b
COX4	P19783	Cytochrome c oxidase subunit 4 isoform 1, mitochondrial	Cox4i1
HSP70	P20029	78 kDa glucose-regulated protein	Hspa5
Glyco_hydro_20;Glycohydro_20b2	P20060	Beta-hexosaminidase subunit beta	Hexb
1-cysPrx_C;AhpC-TSA	P20108	Thioredoxin-dependent peroxide reductase, mitochondrial	Prdx3
Interfer-bind;Tissue_fac	P20352	Tissue factor	F3
MAP2_projctn;Tubulin-binding	P20357	Microtubule-associated protein 2	Map2
C1_1;C2;Pkinase;Pkinase_C	P20444	Protein kinase C alpha type	Prkca
C2-set_2;I-set	P20917;P20917-2	Myelin-associated glycoprotein	Mag
Tropomyosin	P21107-2	Tropomyosin alpha-3 chain	Tpm3
G-alpha	P21278	Guanine nucleotide-binding protein subunit alpha-11	Gna11
G-alpha	P21279;P27600	Guanine nucleotide-binding protein G(q) subunit alpha	Gnaq
ABC_membrane;ABC_tran	P21447	Multidrug resistance protein 1A	Abcb1a
Cystatin	P21460	Cystatin-C	Cst3
P_proprotein;Peptidase_S8	P21661	Neuroendocrine convertase 2	Pcsk2
EGF;F5_F8_type_C;hEGF	P21956-2;P21956	Lactadherin	Mfge8
I-set	P21995	Embigin	Emb
Neur_chan_LBD;Neur_chan_memb	P22723;P22723-2	Gamma-aminobutyric acid receptor subunit gamma-2	Gabrg2
Connexin;Connexin_CCC;Connexin43	P23242	Gap junction alpha-1 protein	Gja1
PCMT	P23506;P23506-2	Protein-L-isoaspartate(D-aspartate) O-methyltransferase	Pcmt1

© 2023. Melachroinou K et al. Published online at http://www.aginganddisease.org/EN/10.14336/AD.2023.0614

SUPPLEMENTARY DATA

ANF_receptor;Lig_chan;Lig_chan-Glu_bd	P23818	Glutamate receptor 1	Gria1
ANF_receptor;Lig_chan;Lig_chan-Glu_bd	P23819-3;P23819-4;P23819;P23819-2	Glutamate receptor 2	Gria2
Crystallin;HSP20	P23927	Alpha-crystallin B chain	Cryab
Catalase;Catalase-rel	P24270	Catalase	Cat
Aminotran_4	P24288	Branched-chain-amino-acid aminotransferase, cytosolic	Bcat1
Pro_isomerase	P24369	Peptidyl-prolyl cis-trans isomerase B	Ppib
GST_C;GST_N	P24472	Glutathione S-transferase A4	Gsta4
Leuk-A4-hydro_C;Peptidase_M1	P24527	Leukotriene A-4 hydrolase	Lta4h
Aldedh	P24549;O35945	Retinal dehydrogenase 1;Aldehyde dehydrogenase, cytosolic 1	Aldh1a1;Aldh1a7
His_Phos_2	P24638	Lysosomal acid phosphatase	Acp2
Man-6-P_recep	P24668	Cation-dependent mannose-6-phosphate receptor	M6pr
ERM;FERM_C;FERM_M;FERM_N	P26043;P26041	Radixin;Moesin	Rdx;Msn
Neur_chan_LBD;Neur_chan_memb	P26048	Gamma-aminobutyric acid receptor subunit alpha-2	Gabra2
Neur_chan_LBD;Neur_chan_memb	P26049	Gamma-aminobutyric acid receptor subunit alpha-3	Gabra3
Prothymosin	P26350	Prothymosin alpha;Prothymosin alpha, N-terminally processed;Thymosin alpha	Ptma
ELFV_dehydrog;ELFV_dehydrog_N	P26443	Glutamate dehydrogenase 1, mitochondrial	Glud1
Seryl_tRNA_N;tRNA-synt_2b	P26638	Serine--tRNA ligase, cytoplasmic	Sars
MARCKS	P26645	Myristoylated alanine-rich C-kinase substrate	Marcks
FKBP_C	P26883	Peptidyl-prolyl cis-trans isomerase FKBP1A	Fkbp1a
G-alpha	P27601	Guanine nucleotide-binding protein subunit alpha-13	Gna13
Thioredoxin	P27773	Protein disulfide-isomerase A3	Pdia3
ADH_N;ADH_zinc_N	P28474;Q64437	Alcohol dehydrogenase class-3	Adh5
SNF	P28571-1;P28571;P28571-2	Sodium- and chloride-dependent glycine transporter 1	Slc6a9
CaMKII_AD;Pkinase	P28652	Calcium/calmodulin-dependent protein kinase type II subunit beta	Camk2b
NAP	P28656	Nucleosome assembly protein 1-like 1	Nap1l1
Nckap1	P28660;P28660-2	Nck-associated protein 1	Nckap1
	P28663	Beta-soluble NSF attachment protein	Napb
Kinesin	P28738;P33175	Kinesin heavy chain isoform 5C;Kinesin heavy chain isoform 5A	Kif5c;Kif5a
WD40	P29387	Guanine nucleotide-binding protein subunit beta-4	Gnb4
Ferritin	P29391;P49945	Ferritin light chain 1;Ferritin light chain 2	Ftl1;Ftl2
C2-set;I-set	P29533;P29533-2	Vascular cell adhesion protein 1	Vcam1
ATP-gua_Ptrans;ATP-gua_PtransN	P30275;Q6P8J7	Creatine kinase U-type, mitochondrial	Ckmt1

© 2023. Melachroinou K et al. Published online at http://www.aginganddisease.org/EN/10.14336/AD.2023.0614

SUPPLEMENTARY DATA

cNMP_binding;RIIa	P31324	cAMP-dependent protein kinase type II-beta regulatory subunit	Prkar2b
SNF	P31648	Sodium- and chloride-dependent GABA transporter 1	Slc6a1
SNF	P31650	Sodium- and chloride-dependent GABA transporter 3	Slc6a11
ACBP	P31786	Acyl-CoA-binding protein	Dbi
Pkinase	P31938;Q63932	Dual specificity mitogen-activated protein kinase kinase 1	Map2k1
Sugar_tr	P32037	Solute carrier family 2, facilitated glucose transporter member 3	Slc2a3
EF-hand_7	P32848	Parvalbumin alpha	Pvalb
Ras	P32883-2;P32883;P01117	GTPase KRas;GTPase KRas, N-terminally processed	Kras
tRNA-synt_1b;WHEP-TRS	P32921;P32921-2	Tryptophan--tRNA ligase, cytoplasmic;T1-TrpRS;T2-TrpRS	Wars
Ran_BP1	P34022	Ran-specific GTPase-activating protein	Ranbp1
MIF	P34884	Macrophage migration inhibitory factor	Mif
SH2;Y_phosphatase	P35235;P35235-1	Tyrosine-protein phosphatase non-receptor type 11	Ptpn11
Ras	P35278	Ras-related protein Rab-5C	Rab5c
Ras	P35279;P35279-2	Ras-related protein Rab-6A	Rab6a
Ras	P35282	Ras-related protein Rab-21	Rab21
Ras	P35293	Ras-related protein Rab-18	Rab18
ANF_receptor;Lig_chan;NMDAR2_C	P35436	Glutamate receptor ionotropic, NMDA 2A	Grin2a
ANF_receptor;CaM_bdg_C0;Lig_chan	P35438;P35438-2	Glutamate receptor ionotropic, NMDA 1	Grin1
Calreticulin	P35564	Calnexin	Canx
1-cysPrx_C;AhpC-TSA	P35700	Peroxiredoxin-1	Prdx1
Tetraspannin	P35762	CD81 antigen	Cd81
Myelin_PLP	P35802	Neuronal membrane glycoprotein M6-a	Gpm6a
Myelin_PLP	P35803-4;P35803-5	Neuronal membrane glycoprotein M6-b	Gpm6b
Myelin_PLP	P35803-8;P35803-3	Neuronal membrane glycoprotein M6-b	Gpm6b
Coprogen_oxidas	P36552	Oxygen-dependent coproporphyrinogen-III oxidase, mitochondrial	Cpox
HSP70	P38647	Stress-70 protein, mitochondrial	Hspa9
Dynamin_M;GED;PH	P39053-3	Dynamin-1	Dnm1
ANF_receptor;Lig_chan;Lig_chan-Glu_bd	P39087;P39087-2	Glutamate receptor ionotropic, kainate 2	Grik2
Pkinase_Tyr;SH2;SH3_1	P39688;P39688-2;Q04736	Tyrosine-protein kinase Fyn;Tyrosine-protein kinase Yes	Fyn;Yes1
CAP_C;CAP_N	P40124	Adenylyl cyclase-associated protein 1	Cap1
Transket_pyr;Transketolase_C;Transketolase_N	P40142	Transketolase	Tkt
Tetraspannin	P40237	CD82 antigen	Cd82

© 2023. Melachroinou K et al. Published online at http://www.aginganddisease.org/EN/10.14336/AD.2023.0614

SUPPLEMENTARY DATA

Tetraspannin	P40240	CD9 antigen	Cd9
Septin	P42208	Septin-2	Σεπ-02
PurA	P42669	Transcriptional activator protein Pur-alpha	Pura
Cpn60_TCP1	P42932	T-complex protein 1 subunit theta	Cct8
SDF	P43006;P43006-2;P43006-3	Excitatory amino acid transporter 2	Slc1a2
COX6A	P43024	Cytochrome c oxidase subunit 6A1, mitochondrial	Cox6a1
FG-GAP;Integrin_alpha;Integrin_alpha2	P43406	Integrin alpha-V;Integrin alpha-V heavy chain;Integrin alpha-V light chain	Itgav
Aldo_ket_red	P45376	Aldose reductase	Akr1b1
Cofilin_ADF	P45591	Cofilin-2	Cfl2
FKBP_C	P45878	Peptidyl-prolyl cis-trans isomerase FKBP2	Fkbp2
C2	P46096	Synaptotagmin-1	Syt1
C2	P46097	Synaptotagmin-2	Syt2
AAA;CDC48_2;CDC48_N;HMG_box	P46460;O54941	Vesicle-fusing ATPase	Nsf
Ras	P46638;P62492;Q9WTL2	Ras-related protein Rab-11B;Ras-related protein Rab-11A	Rab11b;Rab11a
Adenylsucc_synt	P46664	Adenylosuccinate synthetase isozyme 2	Adss
STT3	P46978	Dolichyl-diphosphooligosaccharide--protein glycosyltransferase subunit STT3A	Stt3a
C2	P47708;P70169	Rabphilin-3A	Rph3a
Aldedh	P47738;Q9CZS1;Q62148;Q9JHW9	Aldehyde dehydrogenase, mitochondrial	Aldh2
Aldedh	P47740;P47739	Fatty aldehyde dehydrogenase	Aldh3a2
7tm_1	P47746	Cannabinoid receptor 1	Cnr1
F-actin_cap_A	P47754;P47753	F-actin-capping protein subunit alpha-2	Capza2
F_actin_cap_B	P47757-4;P47757-2;P47757	F-actin-capping protein subunit beta	Capzb
PFK	P47857;P47857-2;P47857-3	ATP-dependent 6-phosphofructokinase, muscle type	Pfkm
Annexin	P48036	Annexin A5	Anxa5
Pyridoxal_deC	P48318	Glutamate decarboxylase 1	Gad1
Pyridoxal_deC	P48320	Glutamate decarboxylase 2	Gad2
HSP70	P48722;P48722-2	Heat shock 70 kDa protein 4L	Hspa4l
adh_short	P48758	Carbonyl reductase [NADPH] 1	Cbr1
COX7a	P48771	Cytochrome c oxidase subunit 7A2, mitochondrial	Cox7a2
GST_C;GST_N	P48774	Glutathione S-transferase Mu 5	Gstm5
Mito_carr	P48962;Q3V132	ADP/ATP translocase 1	Slc25a4
HnRNPA1;RRM_1	P49312;P49312-2	Heterogeneous nuclear ribonucleoprotein A1;Heterogeneous nuclear ribonucleoprotein A1, N-terminally processed	Hnrnpa1

© 2023. Melachroinou K et al. Published online at http://www.aginganddisease.org/EN/10.14336/AD.2023.0614

Inositol_P	P49442	Inositol polyphosphate 1-phosphatase	Inpp1
Pkinase			
Proteasome;Proteasome_A_N	P49722	Proteasome subunit alpha type-2	Psma2
S_100	P50114	Protein S100-B	S100b
adh_short	P50172	Corticosteroid 11-beta-dehydrogenase isozyme 1	Hsd11b1
AdoHcyase;AdoHcyase_NAD	P50247	Adenosylhomocysteinase	Ahcy
GDI	P50396	Rab GDP dissociation inhibitor alpha	Gdi1
ATP-synt_ab;ATP-synt_ab_C;ATP-synt_ab_N	P50516;P50516-2	V-type proton ATPase catalytic subunit A	Atp6v1a
vATP-synt_E	P50518;Q9D593	V-type proton ATPase subunit E 1	Atp6v1e1
Neur_chan_LBD;Neur_chan_memb	P50571	Gamma-aminobutyric acid receptor subunit beta-1	Gabrb1
Ras	P51150	Ras-related protein Rab-7a	Rab7a
Acyl-CoA_dh_1;Acyl-CoA_dh_M;Acyl-CoA_dh_N	P51174	Long-chain specific acyl-CoA dehydrogenase, mitochondrial	Acadl
adh_short;MaoC_dehydratas;SCP2	P51660	Peroxisomal multifunctional enzyme type 2;(3R)-hydroxyacyl-CoA dehydrogenase;Enoyl-CoA hydratase 2	Hsd17b4
CBS;Voltage_CLC	P51791;P51791-2;P51791-4;P51791-5;P51791-3	H(+)/Cl(-) exchange transporter 3	Clcn3
Guanylate_cyc	P51830	Adenylate cyclase type 9	Adcy9
vATP-synt_AC39	P51863	V-type proton ATPase subunit d 1	Atp6v0d1
Mito_carr	P51881	ADP/ATP translocase 2;ADP/ATP translocase 2, N-terminally processed	Slc25a5
SDF	P51906	Excitatory amino acid transporter 3	Slc1a1
Lipocalin_2	P51910	Apolipoprotein D	Apod
PK;PK_C	P52480-2	Pyruvate kinase PKM	Pkm
zf-CHCC	P52503	NADH dehydrogenase [ubiquinone] iron-sulfur protein 6, mitochondrial	Ndufs6
Ribonuc_L-PSP	P52760	Ribonuclease UK114	Hrsp12
Ribosomal_L1	P53026	60S ribosomal protein L10a	Rpl10a
IP_trans	P53810	Phosphatidylinositol transfer protein alpha isoform	Pitpna
IP_trans	P53811	Phosphatidylinositol transfer protein beta isoform	Pitpnb
MFS_1	P53986	Monocarboxylate transporter 1	Slc16a1
Ras	P53994	Ras-related protein Rab-2A	Rab2a
Iso_dh	P54071	Isocitrate dehydrogenase [NADP], mitochondrial	Idh2
Stathmin	P54227	Stathmin	Stmn1
UBA;ubiquitin;XPC-binding	P54728;P54726	UV excision repair protein RAD23 homolog B	Rad23b
AA_permease;AA_permease_N	P55012	Solute carrier family 12 member 2	Slc12a2
Lectin_C;Sushi;V-set;Xlink	P55066	Neurocan core protein	Ncan

© 2023. Melachroinou K et al. Published online at http://www.aginganddisease.org/EN/10.14336/AD.2023.0614

SUPPLEMENTARY DATA

MIP	P55088;P55088-3;P55088-2	Aquaporin-4	Aqp4
Alpha-2-MRAP_C;Alpha-2-MRAP_N	P55302	Alpha-2-macroglobulin receptor-associated protein	Lrpap1
WRW	P56135	ATP synthase subunit f, mitochondrial	Atp5j2
Acylphosphatase	P56376	Acylphosphatase-1	Acyp1
COX6B	P56391	Cytochrome c oxidase subunit 6B1	Cox6b1
UBA;UCH;zf-UBP	P56399	Ubiquitin carboxyl-terminal hydrolase 5	Usp5
ATP-synt_ab;ATP-synt_ab_C;ATP-synt_ab_N	P56480	ATP synthase subunit beta, mitochondrial	Atp5b
SDF	P56564;O35544	Excitatory amino acid transporter 1	Slc1a3
	P56695	Wolframin	Wfs1
Asp	P56818	Beta-secretase 1	Bace1
Nicastrin	P57716	Nicastrin	Ncstn
ATP-synt_D	P57746	V-type proton ATPase subunit D	Atp6v1d
ERp29;ERp29_N	P57759	Endoplasmic reticulum resident protein 29	Erp29
EMP70	P58021	Transmembrane 9 superfamily member 2	Tm9sf2
Metallophos	P58242	Acid sphingomyelinase-like phosphodiesterase 3b	Smpdl3b
EFG_C;EFG_II;EFG_IV;GTP_EFTU;GTP_EFTU_D2	P58252;O08810	Elongation factor 2	Eef2
	P58281;P58281-2	Dynamin-like 120 kDa protein, mitochondrial;Dynamin-like 120 kDa protein, form S1	Opa1
PTPA	P58389	Serine/threonine-protein phosphatase 2A activator	Ppp2r4
C2;Copine	P59108	Copine-2	Cpne2
Lectin_leg-like	P59481	VIP36-like protein	Lman2l
ARPC4	P59999	Actin-related protein 2/3 complex subunit 4	Arpc4
Myelin_PLP	P60202;P60202-2	Myelin proteolipid protein	Plp1
KH_1	P60335;P57722;P57722-2	Poly(rC)-binding protein 1	Pcbp1
Hydrolase_6	P60487	Pyridoxal phosphate phosphatase	Pdxp
Ras	P60766;P60766-1;Q8R527;Q9ER71;Q9ER71-2	Cell division control protein 42 homolog	Cdc42
SNAP-25;SNARE	P60879;O09044	Synaptosomal-associated protein 25	Snap25
SNAP-25;SNARE	P60879-2	Synaptosomal-associated protein 25	Snap25
DnaJ	P60904	DnaJ homolog subfamily C member 5	Dnajc5
Ras	P61021	Ras-related protein Rab-5B	Rab5b
Ras	P61027	Ras-related protein Rab-10	Rab10
UQ_con	P61089	Ubiquitin-conjugating enzyme E2 N	Ube2n
Actin	P61161	Actin-related protein 2	Actr2
Arf	P61205;P84078;Q8BSL7	ADP-ribosylation factor 3;ADP-ribosylation factor 1;ADP-ribosylation factor 2	Arf3;Arf1;Arf2
Ras	P61226	Ras-related protein Rap-2b	Rap2b

© 2023. Melachroinou K et al. Published online at http://www.aginganddisease.org/EN/10.14336/AD.2023.0614

SNARE;Syntaxin	P61264	Syntaxin-1B	Stx1b
Ras	P61294	Ras-related protein Rab-6B	Rab6b
DAD	P61804	Dolichyl-diphosphooligosaccharide--protein glycosyltransferase subunit DAD1	Dad1
Aminotran_3	P61922;P61922-2	4-aminobutyrate aminotransferase, mitochondrial	Abat
NTF2	P61971	Nuclear transport factor 2	Nutf2
KH_1;ROKNT	P61979;P61979-2;P61979-3	Heterogeneous nuclear ribonucleoprotein K	Hnrnpk
14/3/2003	P61982	14-3-3 protein gamma;14-3-3 protein gamma, N-terminally processed	Ywhag
Ras	P62071;P10833	Ras-related protein R-Ras2;Ras-related protein R-Ras	Rras2;Rras
zf-Tim10_DDP	P62075	Mitochondrial import inner membrane translocase subunit Tim13	Timm13
Metallophos	P62141;P62137;P63087;P63088;P63088-2;P63087-2	Serine/threonine-protein phosphatase PP1-beta catalytic subunit;Serine/threonine-protein phosphatase PP1-alpha catalytic subunit;Serine/threonine-protein phosphatase PP1-gamma catalytic subunit	Ppp1cb;Ppp1ca;Ppp1cc
14/3/2003	P62259	14-3-3 protein epsilon	Ywhae
Arf	P62331	ADP-ribosylation factor 6	Arf6
Rdx	P62342	Selenoprotein T	Selt
Aldo_ket_red	P62482	Voltage-gated potassium channel subunit beta-2	Kcnab2
Abi_HHR;SH3_9	P62484	Abl interactor 2	Abi2
GTP_EFTU;GTP_EFTU_D2;GTP_EFTU_D3	P62631	Elongation factor 1-alpha 2	Eef1a2
Clat_adaptor_s	P62743	AP-2 complex subunit sigma	Ap2s1
Ras	P62746	Rho-related GTP-binding protein RhoB	Rhob
EF-hand_7	P62748	Hippocalcin-like protein 1	Hpcal1
EF-hand_1	P62761	Visinin-like protein 1	Vsnl1
Ank_2	P62774	Myotrophin	Mtpn
Histone	P62806	Histone H4	Hist1h4a
Neur_chan_LBD;Neur_chan_memb	P62812	Gamma-aminobutyric acid receptor subunit alpha-1	Gabra1
ATP-synt_ab;ATP-synt_ab_C;ATP-synt_ab_N	P62814	V-type proton ATPase subunit B, brain isoform	Atp6v1b2
Ras	P62821;Q8K386	Ras-related protein Rab-1A	Rab1A
Ras	P62823	Ras-related protein Rab-3C	Rab3c
Ras	P62827;Q61820	GTP-binding nuclear protein Ran;GTP-binding nuclear protein Ran, testis-specific isoform	Ran;Rasl2-9
WD40	P62874;Q61011	Guanine nucleotide-binding protein G(I)/G(S)/G(T) subunit beta-1	Gnb1

© 2023. Melachroinou K et al. Published online at http://www.aginganddisease.org/EN/10.14336/AD.2023.0614

WD40	P62880	Guanine nucleotide-binding protein G(I)/G(S)/G(T) subunit beta-2	Gnb2
WD40	P62881-2;P62881	Guanine nucleotide-binding protein subunit beta-5	Gnb5
Cytochrom_C	P62897;CON__P62894;P00015	Cytochrome c, somatic	Cycs
Ras	P63001;Q05144	Ras-related C3 botulinum toxin substrate 1;Ras-related C3 botulinum toxin substrate 2	Rac1;Rac2
LisH;WD40	P63005;P63005-2	Platelet-activating factor acetylhydrolase IB subunit alpha	Pafah1b1
Ras	P63011	Ras-related protein Rab-3A	Rab3a
HSP70	P63017	Heat shock cognate 71 kDa protein	Hspa8
TCTP	P63028	Translationally-controlled tumor protein	Tpt1
MPC	P63030	Mitochondrial pyruvate carrier 1	Mpc1
CTDII;DnaJ;DnaJ_CXXCXGXG	P63037	DnaJ homolog subfamily A member 1	Dnaja1
Cpn60_TCP1	P63038;P63038-2	60 kDa heat shock protein, mitochondrial	Hspd1
Synaptobrevin	P63044;P63024	Vesicle-associated membrane protein 2;Vesicle-associated membrane protein 3	Vamp2;Vamp3
	P63054	Purkinje cell protein 4	Pcp4
Neur_chan_LBD;Neur_chan_memb	P63080	Gamma-aminobutyric acid receptor subunit beta-3	Gabrb3
ATP-synt_C	P63082	V-type proton ATPase 16 kDa proteolipid subunit	Atp6v0c
Pkinase	P63085;Q61532;Q6P5G0	Mitogen-activated protein kinase 1	Mapk1
G-alpha	P63094;Q6R0H7;P63094-2;Q6R0H7-4;P63094-3	Guanine nucleotide-binding protein G(s) subunit alpha isoforms short;Guanine nucleotide-binding protein G(s) subunit alpha isoforms XLas	Gnas
14/3/2003	P63101	14-3-3 protein zeta/delta	Ywhaz
AA_permease_2	P63115	Asc-type amino acid transporter 1	Slc7a10
Neur_chan_LBD;Neur_chan_memb	P63137;P63137-2	Gamma-aminobutyric acid receptor subunit beta-2	Gabrb2
BTB_2;Ion_trans	P63141	Potassium voltage-gated channel subfamily A member 2	Kcna2
HMG_box;HMG_box_2	P63158	High mobility group protein B1	Hmgb1
G-gamma	P63213	Guanine nucleotide-binding protein G(I)/G(S)/G(O) subunit gamma-2	Gng2
G-gamma	P63216	Guanine nucleotide-binding protein G(I)/G(S)/G(O) subunit gamma-3	Gng3
eIF-5a	P63242;Q8BGY2	Eukaryotic translation initiation factor 5A-1;Eukaryotic translation initiation factor 5A-2	Eif5a;Eif5a2
Actin	P63260	Actin, cytoplasmic 2;Actin, cytoplasmic 2, N-terminally processed	Actg1
C1_1;C2;Pkinase;Pkinase_C	P63318	Protein kinase C gamma type	Prkcg

© 2023. Melachroinou K et al. Published online at http://www.aginganddisease.org/EN/10.14336/AD.2023.0614

SUPPLEMENTARY DATA

Ras	P63321	Ras-related protein Ral-A	Rala
Metallophos	P63328;P63328-2;P48455	Serine/threonine-protein phosphatase 2B catalytic subunit alpha isoform	Ppp3ca
Metallophos	P63330	Serine/threonine-protein phosphatase 2A catalytic subunit alpha isoform	Ppp2ca
Band_7	P67778	Prohibitin	Phb
Actin	P68033;P68134;P62737;P63268	Actin, alpha cardiac muscle 1;Actin, alpha skeletal muscle;Actin, aortic smooth muscle;Actin, gamma-enteric smooth muscle	Actc1;Acta1;Acta2;Actg2
Pkinase	P68181;P68181-4;P68181-2;P68181-3	cAMP-dependent protein kinase catalytic subunit beta	Prkacb
14/3/2003	P68254;P68254-2	14-3-3 protein theta	Ywhaq
Tubulin;Tubulin_C	P68368	Tubulin alpha-4A chain	Tuba4a
Tubulin;Tubulin_C	P68369;P05214	Tubulin alpha-1A chain;Tubulin alpha-3 chain	Tuba1a;Tuba3a
Tubulin;Tubulin_C	P68372	Tubulin beta-4B chain	Tubb4b
C1_1;C2;Pkinase;Pkinase_C	P68404-2;P68404	Protein kinase C beta type	Prkcb
14/3/2003	P68510	14-3-3 protein eta	Ywhah
IBN_N	P70168	Importin subunit beta-1	Kpnb1
Pr_beta_C;Proteasome	P70195	Proteasome subunit beta type-7	Psmb7
Latexin	P70202	Latexin	Lxn
Plexin_cytopl;PSI;Sema;TIG	P70206;P70208;P70208-2	Plexin-A1	Plxna1
Bravo_FIGEY;fn3;I-set	P70232-2;P70232	Neural cell adhesion molecule L1-like protein;Processed neural cell adhesion molecule L1-like protein	Chl1
EBP	P70245	3-beta-hydroxysteroid-Delta(8),Delta(7)-isomerase	Ebp
PBP	P70296;Q8VIN1-2;Q8VIN1	Phosphatidylethanolamine-binding protein 1;Hippocampal cholinergic neurostimulating peptide	Pebp1
SAM_2	P70302	Stromal interaction molecule 1	Stim1
HIT	P70349	Histidine triad nucleotide-binding protein 1	Hint1
Iso_dh	P70404	Isocitrate dehydrogenase [NAD] subunit gamma 1, mitochondrial	Idh3g
Calx-beta;Na_Ca_ex	P70414	Sodium/calcium exchanger 1	Slc8a1
EBP50_C-term;PDZ	P70441	Na(+)/H(+) exchange regulatory cofactor NHE-RF1	Slc9a3r1
FOLN;Kazal_2;SPARC_Ca_bdg	P70663	SPARC-like protein 1	Sparcl1
E1-E2_ATPase	P70704;P70704-3;Q148W0	Phospholipid-transporting ATPase IA	Atp8a1
Cpn60_TCP1	P80313	T-complex protein 1 subunit eta	Cct7
Cpn60_TCP1	P80314	T-complex protein 1 subunit beta	Cct2
Cpn60_TCP1	P80315	T-complex protein 1 subunit delta	Cct4
Cpn60_TCP1	P80316	T-complex protein 1 subunit epsilon	Cct5
Cpn60_TCP1	P80317;Q61390	T-complex protein 1 subunit zeta	Cct6a

© 2023. Melachroinou K et al. Published online at http://www.aginganddisease.org/EN/10.14336/AD.2023.0614

Cpn60_TCP1	P80318	T-complex protein 1 subunit gamma	Cct3
Receptor_IA-2;Y_phosphatase	P80560	Receptor-type tyrosine-protein phosphatase N2	Ptprn2
EF-hand_7	P81117	Nucleobindin-2;Nesfatin-1	Nucb2
Skp1_POZ	P83940	Transcription elongation factor B polypeptide 1	Tceb1
EF-hand_1	P84075	Neuron-specific calcium-binding protein hippocalcin	Hpca
Arf	P84084	ADP-ribosylation factor 5	Arf5
Synaphin	P84086	Complexin-2	Cplx2
Adap_comp_sub;Clat_adaptor_s	P84091	AP-2 complex subunit mu	Ap2m1
Ras	P84096	Rho-related GTP-binding protein RhoG	Rhog
DUF1053;Guanylate_cyc	P84309;P84309-2;Q01341;P51829	Adenylate cyclase type 5	Adcy5
I-set	P97300;P97300-1;P97300-4	Neuroplastin	Nptn
I-set	P97300-3	Neuroplastin	Nptn
LIM	P97315	Cysteine and glycine-rich protein 1	Csrp1
Na_K-ATPase	P97370	Sodium/potassium-transporting ATPase subunit beta-3	Atp1b3
Annexin	P97384	Annexin A11	Anxa11
Amidohydro_1	P97427	Dihydropyrimidinase-related protein 1	Crmp1
Annexin	P97429	Annexin A4	Anxa4
Cation_efflux	P97441	Zinc transporter 3	Slc30a3
Cu2_monoox_C;Cu2_monooxygen;NHL	P97467	Peptidyl-glycine alpha-amidating monooxygenase;Peptidylglycine alpha-hydroxylating monooxygenase;Peptidyl-alpha-hydroxyglycine alpha-amidating lyase	Pam
GRAM;WWbp	P97765;P97765-2	WW domain-binding protein 2	Wbp2
7tm_3;ANF_receptor;GluR_Homer-bdg;NCD3G	P97772;P97772-2;P97772-3	Metabotropic glutamate receptor 1	Grm1
C1-set;V-set	P97797-2;P97797;P97797-4;P97797-3	Tyrosine-protein phosphatase non-receptor type substrate 1	Sirpa
fn3;I-set;Neogenin_C	P97798;P97798-5;P97798-2;P97798-3;P97798-4	Neogenin	Neo1
FumaraseC_C;Lyase_1	P97807;P97807-2	Fumarate hydratase, mitochondrial	Fh
ig	P97952	Sodium channel subunit beta-1	Scn1b
CTP_transf_1	P98191	Phosphatidate cytidylyltransferase 1	Cds1
E1-E2_ATPase	P98200	Phospholipid-transporting ATPase IB	Atp8a2
Tubulin;Tubulin_C	P99024	Tubulin beta-5 chain	Tubb5
Proteasome	P99026	Proteasome subunit beta type-4	Psmb4
UCR_hinge	P99028	Cytochrome b-c1 complex subunit 6, mitochondrial	Uqcrh
Redoxin	P99029;P99029-2	Peroxiredoxin-5, mitochondrial	Prdx5
Peptidase_M14	Q00493	Carboxypeptidase E	Cpe
Serpin	Q00898	Alpha-1-antitrypsin 1-5	Serpina1e

© 2023. Melachroinou K et al. Published online at http://www.aginganddisease.org/EN/10.14336/AD.2023.0614

PDEase_I;PDEase_I_N	Q01065;Q64338;Q64338-2;Q64338-1	Calcium/calmodulin-dependent 3,5-cyclic nucleotide phosphodiesterase 1B	Pde1b
ANF_receptor;Lig_chan;Lig_chan-Glu_bd;NMDAR2_C	Q01097;Q01098;Q03391	Glutamate receptor ionotropic, NMDA 2B	Grin2b
NDK	Q01768	Nucleoside diphosphate kinase B	Nme2
AAA;CDC48_2;CDC48_N;Vps4_C	Q01853	Transitional endoplasmic reticulum ATPase	Vcp
ThiF;UBA_e1_C;UBA_e1_thiolCys;UBACT	Q02053;P31254	Ubiquitin-like modifier-activating enzyme 1	Uba1
Collagen;TNF	Q02105	Complement C1q subcomponent subunit C	C1qc
Arm	Q02248	Catenin beta-1	Ctnnb1
Arm	Q02257	Junction plakoglobin	Jup
EF-hand_7	Q02819	Nucleobindin-1	Nucb1
EphA2_TM;Ephrin_lbd;fn3;Pkinase_Tyr;SAM_2	Q03137;Q03137-2	Ephrin type-A receptor 4	Epha4
APP_amyloid;APP_Cu_bd;APP_E2;APP_N	Q03157	Amyloid-like protein 1;C30	Aplp1
ATP-synt_ab;ATP-synt_ab_C;ATP-synt_ab_N	Q03265	ATP synthase subunit alpha, mitochondrial	Atp5a1
ATP-gua_Ptrans;ATP-gua_PtransN	Q04447	Creatine kinase B-type	Ckb
EF-hand_5;EF-hand_7	Q05186	Reticulocalbin-1	Rcn1
Lipocalin	Q05816	Fatty acid-binding protein, epidermal	Fabp5
ATP-synt_E	Q06185	ATP synthase subunit e, mitochondrial	Atp5i
APP_amyloid;APP_Cu_bd;APP_E2;APP_N	Q06335;Q06335-2	Amyloid-like protein 2	Aplp2
Clusterin	Q06890	Clusterin;Clusterin beta chain;Clusterin alpha chain	Clu
Annexin	Q07076	Annexin A7	Anxa7
EF-hand_6;EF-hand_7	Q08331	Calretinin	Calb2
BK_channel_a;Ion_trans;TrkA_N	Q08460;Q08460-5;Q08460-2;Q08460-4;Q08460-3	Calcium-activated potassium channel subunit alpha-1	Kcnma1
Integrin_beta	Q0VBD0	Integrin beta	Itgb8
	Q0VGU4		Vgf
ERAP1_C;Peptidase_M1	Q11011	Puromycin-sensitive aminopeptidase	Npepps
AMP_N;Peptidase_M24	Q11136	Xaa-Pro dipeptidase	Pepd
DnaJ;DUF1977	Q149L6;Q149L6-2	DnaJ homolog subfamily B member 14	Dnajb14
7tm_3;ANF_receptor;NCD3G	Q14BI2;Q68EF4;Q68EF4-2	Metabotropic glutamate receptor 2	Grm2
C2;Copine	Q1RLL3	Copine-9	Cpne9
Sec7	Q2PFD7;Q2PFD7-3;Q2PFD7-5;Q2PFD7-2;Q2PFD7-4	PH and SEC7 domain-containing protein 3	Psd3
	Q3TAS6-2;Q3TAS6	ER membrane protein complex subunit 10	Emc10
STT3	Q3TDQ1	Dolichyl-diphosphooligosaccharide--protein glycosyltransferase subunit STT3B	Stt3b
Tweety	Q3TH73;Q3TH73-2	Protein tweety homolog 2	Ttyh2
EF-hand_1;EF-hand_7	Q3THE2;Q9CQ19	Myosin regulatory light chain 12B;Myosin regulatory light polypeptide 9	Myl12b;Myl9
S-AdoMet_synt_C;S-AdoMet_synt_M;S-AdoMet_synt_N	Q3THS6;Q91X83	S-adenosylmethionine synthase isoform type-2	Mat2a

© 2023. Melachroinou K et al. Published online at http://www.aginganddisease.org/EN/10.14336/AD.2023.0614

SUPPLEMENTARY DATA

MFS_1	Q3TXX4;Q8BFU8;Q5SZA1;Q61983;Q5SZA1-2	Vesicular glutamate transporter 1	Slc17a7
CPSF_A	Q3U1J4	DNA damage-binding protein 1	Ddb1
	Q3UBX0	Transmembrane protein 109	Tmem109
Hydrolase_6	Q3UGR5;Q3UGR5-2	Haloacid dehalogenase-like hydrolase domain-containing protein 2	Hdhd2
Sugar_tr	Q3UHH2;Q3UHH2-2	Solute carrier family 22 member 23	Slc22a23
Sugar_tr	Q3UHK1	Proton myo-inositol cotransporter	Slc2a13
EGF_2;hEGF;RHS_repeat;Ten_N;Tox-GHH	Q3UHK6;Q3UHK6-2;Q3UHK6-4;Q3UHK6-3;Q9WTS6;Q9WTS6-2;Q9WTS6-3;Q9WTS6-4;Q9WTS6-6;Q9WTS6-8;Q9WTS6-5;Q9WTS6-7	Teneurin-4	Tenm4
Pkinase	Q3UHL1	CaM kinase-like vesicle-associated protein	Camkv
NAD_Gly3P_dh_C;NAD_Gly3P_dh_N	Q3ULJ0;Q3ULJ0-2	Glycerol-3-phosphate dehydrogenase 1-like protein	Gpd1l
LRR_4	Q3UM45	Protein phosphatase 1 regulatory subunit 7	Ppp1r7
Acyltransferase	Q3UN02	Lysocardiolipin acyltransferase 1	Lclat1
PGAP1	Q3UUQ7	GPI inositol-deacylase	Pgap1
Peptidase_M28	Q3UVK0;Q3UVK0-2	Endoplasmic reticulum metallopeptidase 1	Ermp1
7tm_3;ANF_receptor;GluR_Homer-bdg;NCD3G	Q3UVX5;Q3UVX5-2	Metabotropic glutamate receptor 5	Grm5
	Q3UZV7;Q3UZV7-3;Q3UZV7-2;Q3UZV7-5	UPF0577 protein KIAA1324-like homolog	
Costars	Q4KML4	Costars family protein ABRACL	Abracl
LysM;TLD	Q4KMM3;Q4KMM3-2;Q4KMM3-4;Q4KMM3-3	Oxidation resistance protein 1	Oxr1
CUB;Sushi	Q4V9Z5-2;Q4V9Z5-3;Q4V9Z5	Seizure 6-like protein 2	Sez6l2
DUF747	Q4VBD2	Transmembrane anterior posterior transformation protein 1	Tapt1
V-set	Q56A07	Sodium channel subunit beta-2	Scn2b
Lgl_C;LLGL;WD40	Q5DQR4;Q5DQR4-4;Q5DQR4-2;Q5DQR4-3;Q5DQR4-5;Q5DQR4-6	Syntaxin-binding protein 5-like	Stxbp5l
Band_3_cyto;HCO3_cotransp	Q5DTL9;Q5DTL9-9;Q5DTL9-5;Q5DTL9-2;Q5DTL9-7;Q5DTL9-3;Q5DTL9-10;Q5DTL9-6;Q5DTL9-8;Q5DTL9-4	Sodium-driven chloride bicarbonate exchanger	Slc4a10
BTB_2	Q5DTY9	BTB/POZ domain-containing protein KCTD16	Kctd16
CRAL_TRIO_2;RhoGAP	Q5FWK3	Rho GTPase-activating protein 1	Arhgap1
CNRIP1	Q5M8N0	CB1 cannabinoid receptor-interacting protein 1	Cnrip1
Ras	Q5PR73	GTP-binding protein Di-Ras2	Diras2
FragX_IP	Q5SQX6	Cytoplasmic FMR1-interacting protein 2	Cyfip2
IQ;Myosin_head;Myosin_TH1	Q5SYD0-2;Q5SYD0;Q5SUA5	Unconventional myosin-Id	Myo1d
E1_dh;Transket_pyr	Q60597;Q60597-3;Q60597-2;Q60597-4	2-oxoglutarate dehydrogenase, mitochondrial	Ogdh
ICAM_N	Q60625	Intercellular adhesion molecule 5	Icam5
SH2;SH3_1	Q60631;Q60631-2	Growth factor receptor-bound protein 2	Grb2

© 2023. Melachroinou K et al. Published online at http://www.aginganddisease.org/EN/10.14336/AD.2023.0614

Band_7	Q60634;Q60634-3;Q60634-2	Flotillin-2	Flot2
CBFNT;RRM_1	Q60668;Q60668-3;Q60668-4;Q60668-2;Q9Z130	Heterogeneous nuclear ribonucleoprotein D0;Heterogeneous nuclear ribonucleoprotein D-like	Hnrnpd;Hnrnpdl
Receptor_IA-2;RESP18;Y_phosphatase	Q60673	Receptor-type tyrosine-protein phosphatase-like N	Ptprn
Metallophos;PPP5;TPR_1	Q60676	Serine/threonine-protein phosphatase 5	Ppp5c
Proteasome	Q60692	Proteasome subunit beta type-6	Psmb6
Pkinase	Q60737	Casein kinase II subunit alpha	Csnk2a1
Cation_efflux	Q60738	Zinc transporter 1	Slc30a1
Serpin	Q60854	Serpin B6	Serpinb6
TPR_1;TPR_8	Q60864	Stress-induced-phosphoprotein 1	Stip1
TB2_DP1_HVA22	Q60870	Receptor expression-enhancing protein 5	Reep5
Porin_3	Q60930	Voltage-dependent anion-selective channel protein 2	Vdac2
Porin_3	Q60931	Voltage-dependent anion-selective channel protein 3	Vdac3
Porin_3	Q60932-2;Q60932	Voltage-dependent anion-selective channel protein 1	Vdac1
CDC37_C;CDC37_M;CDC37_N	Q61081	Hsp90 co-chaperone Cdc37;Hsp90 co-chaperone Cdc37, N-terminally processed	Cdc37
EF-hand_5;EF-hand_7	Q61112;Q61112-2;Q9BRK5;Q9BRK5-4;Q9BRK5-3;Q9BRK5-2;Q9BRK5-6	45 kDa calcium-binding protein	Sdf4
	Q61137;Q61137-2	Astrotactin-1	Astn1
Na_H_Exchanger	Q61165	Sodium/hydrogen exchanger 1	Slc9a1
1-cysPrx_C;AhpC-TSA	Q61171	Peroxiredoxin-2	Prdx2
	Q61206	Platelet-activating factor acetylhydrolase IB subunit beta	Pafah1b2
SapA;SapB_1;SapB_2	Q61207	Prosaposin	Psap
EGF_3;EGF_CA;Laminin_G_2;VWC	Q61220	Protein kinase C-binding protein NELL2	Nell2
Ca_chan_IQ;Ion_trans	Q61290	Voltage-dependent R-type calcium channel subunit alpha-1E	Cacna1e
Vinculin	Q61301;Q61301-3;Q61301-2;P26231	Catenin alpha-2	Ctnna2
HSP70	Q61316	Heat shock 70 kDa protein 4	Hspa4
SNF	Q61327	Sodium-dependent dopamine transporter	Slc6a3
fn3;I-set	Q61330	Contactin-2	Cntn2
	Q61335	B-cell receptor-associated protein 31	Bcap31
EGF;Lectin_C;Sushi;V-set;Xlink	Q61361	Brevican core protein	Bcan
Ras	Q61411;P23175;P01115;Q61411-2;P01113	GTPase HRas;GTPase HRas, N-terminally processed	Hras
BTB_2;Ion_trans;K_channel_TID	Q61423	Potassium voltage-gated channel subfamily A member 4	Kcna4
3HCDH;3HCDH_N	Q61425	Hydroxyacyl-coenzyme A dehydrogenase, mitochondrial	Hadh

© 2023. Melachroinou K et al. Published online at http://www.aginganddisease.org/EN/10.14336/AD.2023.0614

SUPPLEMENTARY DATA

PDEase_I;PDEase_I_N	Q61481;Q61481-5;Q61481-6;Q61481-2	Calcium/calmodulin-dependent 3,5-cyclic nucleotide phosphodiesterase 1A	Pde1a
C2-set_2	Q61490	CD166 antigen	Alcam
5_nucleotid_C;Metallophos	Q61503	5-nucleotidase	Nt5e
Cys_rich_FGFR	Q61543	Golgi apparatus protein 1	Glg1
ANTH	Q61548;Q61548-3;Q61548-2	Clathrin coat assembly protein AP180	Snap91
Fascin	Q61553	Fascin	Fscn1
GDI	Q61598;Q61598-2	Rab GDP dissociation inhibitor beta	Gdi2
FCH;SH3_9	Q61644	Protein kinase C and casein kinase substrate in neurons protein 1	Pacsin1
HSP70	Q61699;Q61699-2	Heat shock protein 105 kDa	Hsph1
CD47;V-set_CD47	Q61735	Leukocyte surface antigen CD47	Cd47
CD47;V-set_CD47	Q61735-2	Leukocyte surface antigen CD47	Cd47
2-Hacid_dh;2-Hacid_dh_C	Q61753	D-3-phosphoglycerate dehydrogenase	Phgdh
IQ;Myosin_head;Myosin_N;Myosin_tail_1	Q61879;O08638;O08638-2	Myosin-10	Myh10
V-set	Q61885	Myelin-oligodendrocyte glycoprotein	Mog
DED	Q62048;Q62048-2	Astrocytic phosphoprotein PEA-15	Pea15
EGF;Lectin_C;Sushi;V-set;Xlink	Q62059;Q62059-4;Q62059-3;Q62059-2	Versican core protein	Vcan
Arylesterase	Q62086	Serum paraoxonase/arylesterase 2	Pon2
Guanylate_kin;MAGUK_N_PEST;PDZ;PDZ_assoc;SH3_2	Q62108;Q62108-3;Q62108-2	Disks large homolog 4	Dlg4
PSI;Sema	Q62178	Semaphorin-4A	Sema4a
TRAP-delta	Q62186	Translocon-associated protein subunit delta	Ssr4
Amidohydro_1	Q62188	Dihydropyrimidinase-related protein 3	Dpysl3
CH;Spectrin	Q62261;Q62261-2	Spectrin beta chain, non-erythrocytic 1	Sptbn1
MARVEL	Q62277	Synaptophysin	Syp
Tetraspannin	Q62283	Tetraspanin-7	Tspan7
Translin	Q62348	Translin	Tsn
PA;Peptidase_M28;TFR_dimer	Q62351	Transferrin receptor protein 1	Tfrc
BAR;SH3_1;SH3_9	Q62420;Q62419	Endophilin-A1	Sh3gl2
B12D	Q62425	Cytochrome c oxidase subunit NDUFA4	Ndufa4
Ndr	Q62433	Protein NDRG1	Ndrg1
Synaptobrevin	Q62442;Q62442-2	Vesicle-associated membrane protein 1	Vamp1
Pentaxin	Q62443	Neuronal pentraxin-1	Nptx1
EF-hand_1;EF-hand_7	Q63810;Q63810-2;Q63811	Calcineurin subunit B type 1	Ppp3r1
LRR_4;LRR_8;LRRNT	Q63912	Oligodendrocyte-myelin glycoprotein	Omg
V-set	Q640R3	Hepatocyte cell adhesion molecule	Hepacam
adh_short	Q64105	Sepiapterin reductase	Spr

© 2023. Melachroinou K et al. Published online at http://www.aginganddisease.org/EN/10.14336/AD.2023.0614

SUPPLEMENTARY DATA

Amino_oxidase	Q64133	Amine oxidase [flavin-containing] A	Maoa
SURF4	Q64310	Surfeit locus protein 4	Surf4
CD34_antigen	Q64314;Q64314-2	Hematopoietic progenitor cell antigen CD34	Cd34
Synapsin;Synapsin_C;Synapsin_N	Q64332;Q64332-2	Synapsin-2	Syn2
Cpn10	Q64433	10 kDa heat shock protein, mitochondrial	Hspe1
Carb_anhydrase	Q64444	Carbonic anhydrase 4	Ca4
fn3;Y_phosphatase	Q64455	Receptor-type tyrosine-protein phosphatase eta	Ptprj
fn3;I-set;Y_phosphatase	Q64487;Q64487-12;Q64487-5;Q64487-7;Q64487-6;Q64487-1;Q64487-11;Q64487-9;Q64487-10;Q64487-8;Q64487-2;Q64487-3;A2A8L5	Receptor-type tyrosine-protein phosphatase delta	Ptprd
Peptidase_S8;TPPII	Q64514;Q64514-2	Tripeptidyl-peptidase 2	Tpp2
FGGY_C;FGGY_N	Q64516;Q64516-1;Q64516-2	Glycerol kinase	Gk
DAO;EF-hand_7	Q64521	Glycerol-3-phosphate dehydrogenase, mitochondrial	Gpd2
UDPGT	Q64676	2-hydroxyacylsphingosine 1-beta-galactosyltransferase	Ugt8
Sushi	Q64735;Q64735-2	Complement component receptor 1-like protein	Cr1l
Na_sulph_symp	Q67BT3	Solute carrier family 13 member 5	Slc13a5
7tm_3;ANF_receptor;NCD3G	Q68ED2	Metabotropic glutamate receptor 7	Grm7
Clathrin;Clathrin_propel;Clathrin-link	Q68FD5	Clathrin heavy chain 1	Cltc
APG9	Q68FE2	Autophagy-related protein 9A	Atg9a
fn3;I-set	Q69Z26;Q69Z26-2;Q69Z26-3;Q07409	Contactin-4	Cntn4
COesterase	Q69ZK9;B0F2B4	Neuroligin-2	Nlgn2
MFS_1;Pentapeptide_4;Sugar_tr	Q69ZS6	Synaptic vesicle glycoprotein 2C	Sv2c
	Q6GQT9	Nodal modulator 1	Nomo1
DPPIV_N;Peptidase_S9	Q6NXK7	Inactive dipeptidyl peptidase 10	Dpp10
EF-hand_7	Q6P069;Q6P069-2	Sorcin	Sri
CUB;Sushi	Q6P1D5;Q6P1D5-2	Seizure 6-like protein	Sez6l
	Q6P1F6;Q925E7;Q8BG02;Q6ZWR4;Q6ZWR4-3;Q6ZWR4-2	Serine/threonine-protein phosphatase 2A 55 kDa regulatory subunit B alpha isoform	Ppp2r2a
	Q6P2L7;Q6P2L7-3;Q6P2L7-2	Protein CASC4	Casc4
UDP-g_GGTase	Q6P5E4	UDP-glucose:glycoprotein glucosyltransferase 1	Uggt1
Tweety	Q6P5F7;Q6P5F7-2	Protein tweety homolog 3	Ttyh3
Laminin_G_2;Syndecan	Q6P9K9;Q6P9K9-2;Q8C985	Neurexin-3	Nrxn3
Glycos_transf_2;Ricin_B_lectin	Q6PB93-2;Q6PB93	Polypeptide N-acetylgalactosaminyltransferase 2;Polypeptide N-acetylgalactosaminyltransferase 2 soluble form	Galnt2
PIG-S	Q6PD26	GPI transamidase component PIG-S	Pigs
	Q6PE13	Proline-rich transmembrane protein 3	Prrt3

© 2023. Melachroinou K et al. Published online at http://www.aginganddisease.org/EN/10.14336/AD.2023.0614

SUPPLEMENTARY DATA

CH;EB1	Q6PER3	Microtubule-associated protein RP/EB family member 3	Mapre3
SNF	Q6PGE7	Sodium-dependent proline transporter	Slc6a7
Ras	Q6PHN9;Q923S9	Ras-related protein Rab-35	Rab35
Cache_1;VGCC_alpha2;VWA;VWA_N	Q6PHS9-4;Q6PHS9-2;Q6PHS9-5;Q6PHS9;Q6PHS9-3;Q6PHS9-6	Voltage-dependent calcium channel subunit alpha-2/delta-2;Voltage-dependent calcium channel subunit alpha-2-2;Voltage-dependent calcium channel subunit delta-2	Cacna2d2
	Q6PHU5;Q6PHU5-2	Sortilin	Sort1
CaMKII_AD;Pkinase	Q6PHZ2;Q6PHZ2-5;Q6PHZ2-2;Q6PHZ2-4	Calcium/calmodulin-dependent protein kinase type II subunit delta	Camk2d
Cation_ATPase_C;Cation_ATPase_N;E1-E2_ATPase;H-K_ATPase_N;Hydrolase	Q6PIC6;Q64436	Sodium/potassium-transporting ATPase subunit alpha-3	Atp1a3
Cation_ATPase_C;Cation_ATPase_N;E1-E2_ATPase;Hydrolase	Q6PIE5	Sodium/potassium-transporting ATPase subunit alpha-2	Atp1a2
ATP_Ca_trans_C;Cation_ATPase_C;Cation_ATPase_N;E1-E2_ATPase;Hydrolase	Q6Q477;Q6Q477-2	Calcium-transporting ATPase	Atp2b4
VKOR	Q6TEK5	Vitamin K epoxide reductase complex subunit 1-like protein 1	Vkorc1l1
Choline_transpo	Q6X893;Q6X893-2	Choline transporter-like protein 1	Slc44a1
TIP120	Q6ZQ38	Cullin-associated NEDD8-dissociated protein 1	Cand1
Malectin	Q6ZQI3	Malectin	Mlec
SPC22	Q6ZWQ7		Spcs3
Chon_Sulph_att;Neural_ProG_Cyt	Q71M36-2;Q71M36-3	Chondroitin sulfate proteoglycan 5	Cspg5
HEAT	Q76MZ3	Serine/threonine-protein phosphatase 2A 65 kDa regulatory subunit A alpha isoform	Ppp2r1a
	Q78IK2	Up-regulated during skeletal muscle growth protein 5	Usmg5
NAP	Q78ZA7	Nucleosome assembly protein 1-like 4	Nap1l4
Mito_carr	Q791T5;Q791T5-2	Mitochondrial carrier homolog 1	Mtch1
Mito_carr	Q791V5	Mitochondrial carrier homolog 2	Mtch2
V-set	Q7M729	Sodium channel subunit beta-4	Scn4b
	Q7TMF3	NADH dehydrogenase [ubiquinone] 1 alpha subcomplex subunit 12	Ndufa12
Tubulin;Tubulin_C	Q7TMM9	Tubulin beta-2A chain	Tubb2a
ABC_tran;ABC2_membrane	Q7TMS5	ATP-binding cassette sub-family G member 2	Abcg2
Exo_endo_phos	Q7TNC9;Q7TNC9-2		Inpp5a
V-set	Q7TNR6	Immunoglobulin superfamily member 21	Igsf21
CH;EF-hand_6;EFhand_Ca_insen;Spectrin	Q7TPR4;Q9JI91	Alpha-actinin-1	Actn1
p25-alpha	Q7TQD2	Tubulin polymerization-promoting protein	Tppp
BAR;SH3_9	Q7TQF7;D3Z6Q9	Amphiphysin	Amph

SUPPLEMENTARY DATA

Peptidase_C65	Q7TQI3	Ubiquitin thioesterase OTUB1	Otub1
AdoHcyase;AdoHcyase_NAD	Q80SW1	Putative adenosylhomocysteinase 2	Ahcyl1
7tm_3;ANF_receptor	Q80T41	Gamma-aminobutyric acid type B receptor subunit 2	Gabbr2
DUF1041;PH	Q80TJ1;Q80TJ1-4;Q80TJ1-2;Q8BYR5;Q8BYR5-2;Q8BYR5-5;Q8BYR5-4;Q8BYR5-3;Q8BYR5-6	Calcium-dependent secretion activator 1	Cadps
	Q80TL4;Q80TL4-2	Protein KIAA1045	Kiaa1045
DnaJ;Thioredoxin	Q80TN4	DnaJ homolog subfamily C member 16	Dnajc16
7tm_2;DUF3497;Gal_Lectin;GPS;HRM;Latrophilin;OLF	Q80TR1;Q80TR1-2	Latrophilin-1	Lphn1
7tm_2;DUF3497;Gal_Lectin;GPS;HRM;Latrophilin;OLF	Q80TS3;Q80TS3-2;Q80TS3-5;Q80TS3-3;Q80TS3-4;Q80TS3-6	Latrophilin-3	Lphn3
DnaJ;Pkinase;PTEN_C2	Q80TZ3;Q80TZ3-2;Q80TZ3-3;Q99KY4;Q99KY4-2	Putative tyrosine-protein phosphatase auxilin	Dnajc6
Plexin_cytopl;PSI;Sema;TIG	Q80UG2	Plexin-A4	Plxna4
Septin	Q80UG5;Q80UG5-3;Q80UG5-2	Septin-9	septin9
Glyco_hydro_63	Q80UM7	Mannosyl-oligosaccharide glucosidase	Mogs
FBA;F-box	Q80UW2	F-box only protein 2	Fbxo2
Inositol_P	Q80V26	Inositol monophosphatase 3	Impad1
ENTH;UIM	Q80VP1;Q80VP1-2	Epsin-1	Epn1
Aldedh	Q80VQ0;J3QMK6;E9Q3E1	Aldehyde dehydrogenase family 3 member B1	Aldh3b1
Peptidase_M48	Q80W54	CAAX prenyl protease 1 homolog	Zmpste24
DUF3733;LRR_4;LRR_8	Q80WG5;Q8R502	Volume-regulated anion channel subunit LRRC8A	Lrrc8a
V-set;Xlink	Q80WM4	Hyaluronan and proteoglycan link protein 4	Hapln4
PIP5K	Q80XI4	Phosphatidylinositol 5-phosphate 4-kinase type-2 beta	Pip4k2b
adh_short	Q80XN0	D-beta-hydroxybutyrate dehydrogenase, mitochondrial	Bdh1
I-set	Q80Z24	Neuronal growth regulator 1	Negr1
Ras	Q80ZJ1;Q8BU31;Q80ZJ1-2	Ras-related protein Rap-2a;Ras-related protein Rap-2c	Rap2a;Rap2c
	Q80ZW2	Protein THEM6	Them6
Bravo_FIGEY;fn3;I-set	Q810U3	Neurofascin	Nfasc
Bravo_FIGEY;fn3;I-set	Q810U4;Q810U4-3;Q810U4-2;Q810U4-4	Neuronal cell adhesion molecule	Nrcam
Guanylate_kin;L27_1;MAGUK_N_PEST;PDZ;PDZ_assoc;SH3_1	Q811D0;Q811D0-2;Q811D0-3;P70175	Disks large homolog 1	Dlg1
GTP_EFTU;GTP_EFTU_D2;GTP_EFTU_D3	Q8BFR5;Q8BFR5-2	Elongation factor Tu, mitochondrial	Tufm
EF-hand_7	Q8BFY6	Peflin	Pef1
Band_7	Q8BFZ9	Erlin-2	Erlin2
MFS_1;Pentapeptide_4;Sugar_tr	Q8BG39	Synaptic vesicle glycoprotein 2B	Sv2b
HAD_2	Q8BGB7;Q8BGB7-2	Enolase-phosphatase E1	Enoph1
Carn_acyltransf	Q8BGD5	Carnitine O-palmitoyltransferase 1, brain isoform	Cpt1c

© 2023. Melachroinou K et al. Published online at http://www.aginganddisease.org/EN/10.14336/AD.2023.0614

SUPPLEMENTARY DATA

Bac_surface_Ag	Q8BGH2	Sorting and assembly machinery component 50 homolog	Samm50
Phosphodiest	Q8BGN3	Ectonucleotide pyrophosphatase/phosphodiesterase family member 6	Enpp6
MARVEL	Q8BGN8-2;Q8BGN8	Synaptoporin	Synpr
DHHA1;tRNA_SAD;tRNA-synt_2c	Q8BGQ7	Alanine--tRNA ligase, cytoplasmic	Aars
CDP-OH_P_transf	Q8BGS7;Q8BGS7-2	Choline/ethanolaminephosphotransferase 1	Cept1
fn3	Q8BGT8;Q8BGT8-2	Phytanoyl-CoA hydroxylase-interacting protein-like	Phyhipl
Cyclin_N	Q8BGU5;Q8BGU5-2	Cyclin-Y	Ccny
EF-hand_1;EF-hand_7	Q8BGZ1	Hippocalcin-like protein 4	Hpcal4
EMP70	Q8BH24	Transmembrane 9 superfamily member 4	Tm9sf4
EF-hand_6;Mito_carr	Q8BH59	Calcium-binding mitochondrial carrier protein Aralar1	Slc25a12
ECH	Q8BH95	Enoyl-CoA hydratase, mitochondrial	Echs1
BNIP2;CRAL_TRIO_2	Q8BHE3;O54940	Caytaxin;BCL2/adenovirus E1B 19 kDa protein-interacting protein 2	Atcay;Bnip2
	Q8BHF7	CDP-diacylglycerol--glycerol-3-phosphate 3-phosphatidyltransferase, mitochondrial	Pgs1
ELO	Q8BHI7	Elongation of very long chain fatty acids protein 5	Elovl5
V-set	Q8BHK2	Sodium channel subunit beta-3	Scn3b
Gal_mutarotas_2;Glyco_hydro_31	Q8BHN3;Q8BHN3-2;Q8BHN3-3	Neutral alpha-glucosidase AB	Ganab
SNF	Q8BJI1	Sodium-dependent neutral amino acid transporter SLC6A17	Slc6a17
Tetraspannin	Q8BJU2	Tetraspanin-9	Tspan9
Aha1_N;AHSA1	Q8BK64	Activator of 90 kDa heat shock protein ATPase homolog 1	Ahsa1
Lactamase_B	Q8BL86	Metallo-beta-lactamase domain-containing protein 2	Mblac2
MFS_1	Q8BLE7	Vesicular glutamate transporter 2	Slc17a6
Abhydrolase_3	Q8BLF1	Neutral cholesterol ester hydrolase 1	Nceh1
I-set	Q8BLK3	Limbic system-associated membrane protein	Lsamp
C2-set_2;I-set;V-set	Q8BLQ9;Q8BLQ9-3;Q8BLQ9-2	Cell adhesion molecule 2	Cadm2
C2;Copine	Q8BLR2	Copine-4	Cpne4
HSP70	Q8BM72;Q8BM72-2	Heat shock 70 kDa protein 13	Hspa13
	Q8BMK4	Cytoskeleton-associated protein 4	Ckap4
EF-hand_1	Q8BNY6	Neuronal calcium sensor 1	Ncs1
tRNA_anti-codon;tRNA-synt_2	Q8BP47	Asparagine--tRNA ligase, cytoplasmic	Nars
EF-hand_5;EF-hand_7	Q8BP92	Reticulocalbin-2	Rcn2
Rav1p_C;WD40	Q8BPN8;Q8BPN8-2;Q8BPN8-3;Q6PNC0	DmX-like protein 2	Dmxl2

© 2023. Melachroinou K et al. Published online at http://www.aginganddisease.org/EN/10.14336/AD.2023.0614

SUPPLEMENTARY DATA

OST3_OST6;Thioredoxin	Q8BTV1	Tumor suppressor candidate 3	Tusc3
Band_3_cyto;HCO3_cotransp	Q8BTY2;Q8BTY2-2	Sodium bicarbonate cotransporter 3	Slc4a7
Sec62	Q8BU14	Translocation protein SEC62	Sec62
TPP_enzyme_C;TPP_enzyme_M;TPP_enzyme_N	Q8BU33;Q8BU33-3;Q8BU33-2	Acetolactate synthase-like protein	Ilvbl
MoCF_biosynth;MoeA_C;MoeA_N	Q8BUV3	Gephyrin;Molybdopterin adenylyltransferase;Molybdopterin molybdenumtransferase	Gphn
V-ATPase_H_C;V-ATPase_H_N	Q8BVE3	V-type proton ATPase subunit H	Atp6v1h
Acetyltransf_1	Q8BVG8	N-acetyltransferase 14	Nat14
adh_short	Q8BVI4	Dihydropteridine reductase	Qdpr
Abhydrolase_6	Q8BVQ5	Protein phosphatase methylesterase 1	Ppme1
DUF563	Q8BW41;Q8BW41-2	Protein O-linked-mannose beta-1,4-N-acetylglucosaminyltransferase 2	Pomgnt2
Amino_oxidase	Q8BW75	Amine oxidase [flavin-containing] B	Maob
Aldedh	Q8BWF0	Succinate-semialdehyde dehydrogenase, mitochondrial	Aldh5a1
PITH	Q8BWR2;Q8BWR2-2	PITH domain-containing protein 1	Pithd1
Lung_7-TM_R	Q8BXN9;Q8BXN9-3;Q8BXN9-2	Transmembrane protein 87A	Tmem87a
Gpi16	Q8BXQ2	GPI transamidase component PIG-T	Pigt
AA_permease_2;AA_permease_C	Q8BXR1	Probable cationic amino acid transporter	Slc7a14
	Q8BXV2	BRI3-binding protein	Bri3bp
Thioredoxin	Q8BXZ1	Protein disulfide-isomerase TMX3	Tmx3
Choline_transpo	Q8BY89-2;Q8BY89	Choline transporter-like protein 2	Slc44a2
	Q8BYI8;Q8BYI8-2	Uncharacterized protein KIAA1467	Kiaa1467
EGF_2;Fibrinogen_C;fn3	Q8BYI9;Q8BYI9-2	Tenascin-R	Tnr
COesterase	Q8BYM5	Neuroligin-3	Nlgn3
Dynamin_M;GED;PH	Q8BZ98;Q8BZ98-2	Dynamin-3	Dnm3
Thioredoxin	Q8C0L0	Thioredoxin-related transmembrane protein 4	Tmx4
Asparaginase_2	Q8C0M9	Isoaspartyl peptidase/L-asparaginase;Isoaspartyl peptidase/L-asparaginase alpha chain;Isoaspartyl peptidase/L-asparaginase beta chain	Asrgl1
ERAP1_C;Peptidase_M1	Q8C129	Leucyl-cystinyl aminopeptidase	Lnpep
Septin	Q8C1B7;Q8C1B7-3;Q8C1B7-2	Septin-11	septin11
7tm_3	Q8C419	Probable G-protein coupled receptor 158	Gpr158
	Q8C522	Endonuclease domain-containing 1 protein	Endod1
LMBR1	Q8C561;Q8C561-2;Q8C561-3	LMBR1 domain-containing protein 2	Lmbrd2
Prenylcys_lyase	Q8C7K6	Prenylcysteine oxidase-like	Pcyox1l
DUF1620	Q8C7X2;Q8C7X2-3	ER membrane protein complex subunit 1	Emc1

E2_bind;ThiF;UBA_e1_thiolCys;UBACT	Q8C878;Q8C878-2	NEDD8-activating enzyme E1 catalytic subunit	Uba3
Ank;Ank_2;Death;ZU5	Q8C8R3-2;Q8C8R3;Q8C8R3-4;Q8C8R3-5;Q8C8R3-3;Q8C8R3-7;Q8C8R3-8;Q8C8R3-6;Q02357-2;G5E8K5-6;G5E8K5-4;Q02357;G5E8K5-5;Q02357-5;Q02357-3;Q02357-4;Q02357-6	Ankyrin-2	Ank2
Shisa	Q8CA71	Protein shisa-4	Shisa4
GAF;PDEase_I	Q8CA95-3;Q8CA95;Q8CA95-2;Q8CA95-4	cAMP and cAMP-inhibited cGMP 3,5-cyclic phosphodiesterase 10A	Pde10a
PGM_PMM_I;PGM_PMM_II;PGM_PMM_III;PGM_PMM_IV	Q8CAA7	Glucose 1,6-bisphosphate synthase	Pgm2l1
Mitofilin	Q8CAQ8;Q8CAQ8-2;Q8CAQ8-5;Q8CAQ8-3	MICOS complex subunit Mic60	Immt
Thiolase_C;Thiolase_N	Q8CAY6	Acetyl-CoA acetyltransferase, cytosolic	Acat2
MFS_1;MFS_1_like	Q8CBH5-2;Q8CBH5	Major facilitator superfamily domain-containing protein 6	Mfsd6
	Q8CFV4	Neuritin	Nrn1
G-alpha	Q8CGK7	Guanine nucleotide-binding protein G(olf) subunit alpha	Gnal
DUF1866;Exo_endo_phos;Syja_N	Q8CHC4	Synaptojanin-1	Synj1
Septin	Q8CHH9;Q8CHH9-2	Septin-8	septin8
MBOAT	Q8CHK3	Lysophospholipid acyltransferase 7	Mboat7
RhoGEF;SH3_1	Q8CHT1;Q8CHT1-2	Ephexin-1	Ngef
Phosphorylase	Q8CI94	Glycogen phosphorylase, brain form	Pygb
V-set	Q8HW98	IgLON family member 5	Iglon5
L27;PDZ	Q8JZS0;O88951	Protein lin-7 homolog A	Lin7a
C2;Copine	Q8JZW4	Copine-5	Cpne5
	Q8K0S0	Phytanoyl-CoA hydroxylase-interacting protein	Phyhip
Reticulon	Q8K0T0	Reticulon-1	Rtn1
	Q8K0U4	Heat shock 70 kDa protein 12A	Hspa12a
REJ	Q8K135;Q8K135-2	Dyslexia-associated protein KIAA0319-like protein	Kiaa0319l
PfkB	Q8K183	Pyridoxal kinase	Pdxk
Dynamin_M;GED	Q8K1M6;Q8K1M6-3;Q8K1M6-2;Q8K1M6-4;Q8K1M6-5	Dynamin-1-like protein	Dnm1l
FAD_binding_2;Succ_DH_flav_C	Q8K2B3	Succinate dehydrogenase [ubiquinone] flavoprotein subunit, mitochondrial	Sdha
CS;PTPLA	Q8K2C9	Very-long-chain (3R)-3-hydroxyacyl-CoA dehydratase 3	Hacd3
PIG-U	Q8K358	Phosphatidylinositol glycan anchor biosynthesis class U protein	Pigu
Fer4_7	Q8K3J1	NADH dehydrogenase [ubiquinone] iron-sulfur protein 8, mitochondrial	Ndufs8
Lgl_C;LLGL;WD40	Q8K400;Q8K400-2	Syntaxin-binding protein 5	Stxbp5
EPTP;LRR_8	Q8K406	Leucine-rich repeat LGI family member 3	Lgi3
IP_trans	Q8K4R4;Q8K4R4-3;Q8K4R4-2;Q8K4R4-4	Cytoplasmic phosphatidylinositol transfer protein 1	Pitpnc1

© 2023. Melachroinou K et al. Published online at http://www.aginganddisease.org/EN/10.14336/AD.2023.0614

EPTP;LRR_8	Q8K4Z0-2;Q8K4Z0	Leucine-rich repeat LGI family member 2	Lgi2
Calx-beta;Na_Ca_ex	Q8K596;Q8K596-2		Slc8a2
Thiolase_C;Thiolase_N	Q8QZT1	Acetyl-CoA acetyltransferase, mitochondrial	Acat1
Tetraspannin	Q8QZY6	Tetraspanin-14	Tspan14
Peptidase_C1_2	Q8R016	Bleomycin hydrolase	Blmh
BEX	Q8R0A5	Transcription elongation factor A protein-like 3	Tceal3
Aldedh;Formyl_trans_C;Formyl_trans_N;PP-binding	Q8R0Y6;Q8K009	Cytosolic 10-formyltetrahydrofolate dehydrogenase	Aldh1l1
ERO1	Q8R180;Q8R2E9	ERO1-like protein alpha	Ero1l
MARVEL	Q8R191	Synaptogyrin-3	Syngr3
UCR_UQCRX_QCR9	Q8R1I1	Cytochrome b-c1 complex subunit 9	Uqcr10
EMP24_GP25L	Q8R1V4	Transmembrane emp24 domain-containing protein 4	Tmed4
V-set	Q8R366	Immunoglobulin superfamily member 8	Igsf8
MBOAT	Q8R3I2;Q8R3I2-2;Q8R3I2-3	Lysophospholipid acyltransferase 2	Mboat2
BAR;SH3_9	Q8R3V5;Q8R3V5-3;Q8R3V5-1	Endophilin-B2	Sh3glb2
C2-set_2;V-set	Q8R464	Cell adhesion molecule 4	Cadm4
EGF_CA;UnbV_ASPIC	Q8R555	Cartilage acidic protein 1	Crtac1
Actin	Q8R5C5	Beta-centractin	Actr1b
C2-set_2;V-set	Q8R5M8-4;Q8R5M8;Q8R5M8-3;Q8R5M8-2;Q8R5M8-5;Q8R5M8-7;Q8R5M8-6	Cell adhesion molecule 1	Cadm1
Thioredoxin	Q8VBT0	Thioredoxin-related transmembrane protein 1	Tmx1
CLPTM1	Q8VBZ3	Cleft lip and palate transmembrane protein 1 homolog	Clptm1
Peptidase_M28	Q8VCM8	Nicalin	Ncln
ADP_PFK_GK	Q8VDL4;Q8VDL4-3;Q8VDL4-2	ADP-dependent glucokinase	Adpgk
Cation_ATPase_C;Cation_ATPase_N;E1-E2_ATPase;Hydrolase	Q8VDN2	Sodium/potassium-transporting ATPase subunit alpha-1	Atp1a1
CDP-OH_P_transf	Q8VDP6	CDP-diacylglycerol--inositol 3-phosphatidyltransferase	Cdipt
SIR2	Q8VDQ8;Q8VDQ8-2;Q8VDQ8-4;Q8VDQ8-3	NAD-dependent protein deacetylase sirtuin-2	Sirt2
Arf	Q8VEH3	ADP-ribosylation factor-like protein 8A	Arl8a
PRKCSH	Q8VEH8	Endoplasmic reticulum lectin 1	Erlec1
CDC50	Q8VEK0;Q8BHG3	Cell cycle control protein 50A	Tmem30a
Mito_carr	Q8VEM8	Phosphate carrier protein, mitochondrial	Slc25a3
O-FucT	Q8VHI3	GDP-fucose protein O-fucosyltransferase 2	Pofut2
UT	Q8VHL0;Q8VHL0-2	Urea transporter 1	Slc14a1
PMP22_Claudin	Q8VHW2	Voltage-dependent calcium channel gamma-8 subunit	Cacng8
4HBT	Q91V12-2;Q91V12;Q91V12-4;Q91V12-3	Cytosolic acyl coenzyme A thioester hydrolase	Acot7
AA_permease	Q91V14;Q91V14-2	Solute carrier family 12 member 5	Slc12a5

© 2023. Melachroinou K et al. Published online at http://www.aginganddisease.org/EN/10.14336/AD.2023.0614

SUPPLEMENTARY DATA

ATP-synt_C	Q91V37	V-type proton ATPase 21 kDa proteolipid subunit	Atp6v0b
Ras	Q91V41	Ras-related protein Rab-14	Rab14
Mtc	Q91V61;Q91V61-2	Sideroflexin-3	Sfxn3
ATP-grasp_2;Citrate_synt;CoA_binding;Ligase_CoA	Q91V92	ATP-citrate synthase	Acly
DUF1982;Molybdopterin;NADH-G_4Fe-4S_3	Q91VD9	NADH-ubiquinone oxidoreductase 75 kDa subunit, mitochondrial	Ndufs1
AMP-binding;AMP-binding_C	Q91VE0	Long-chain fatty acid transport protein 4	Slc27a4
BRICHOS	Q91VK4	Integral membrane protein 2C;CT-BRI3	Itm2c
Pyrophosphatase	Q91VM9;Q91VM9-2	Inorganic pyrophosphatase 2, mitochondrial	Ppa2
ATP-synt	Q91VR2	ATP synthase subunit gamma, mitochondrial	Atp5c1
Atg8	Q91VR7	Microtubule-associated proteins 1A/1B light chain 3A	Map1lc3a
	Q91VU0	Protein FAM3C	Fam3c
Erf4	Q91W53;Q91W53-2	Golgin subfamily A member 7	Golga7
Thioredoxin	Q91W90	Thioredoxin domain-containing protein 5	Txndc5
Hist_deacetyl	Q91WA3	Histone deacetylase 11	Hdac11
Complex1_49kDa	Q91WD5	NADH dehydrogenase [ubiquinone] iron-sulfur protein 2, mitochondrial	Ndufs2
tRNA_bind;tRNA-synt_1b	Q91WQ3	Tyrosine--tRNA ligase, cytoplasmic;Tyrosine--tRNA ligase, cytoplasmic, N-terminally processed	Yars
MitoNEET_N;zf-CDGSH	Q91WS0	CDGSH iron-sulfur domain-containing protein 1	Cisd1
Band_7	Q91X78	Erlin-1	Erlin1
EF-hand_1;EF-hand_7	Q91X97	Neurocalcin-delta	Ncald
DUF3456;EGF_CA	Q91XD7	Cysteine-rich with EGF-like domain protein 1	Creld1
ATP_synt_H	Q91XE7	V-type proton ATPase subunit e 2	Atp6v0e2
Guanylate_kin;MAGUK_N_PEST;PDZ;PDZ_assoc;SH3_1	Q91XM9;Q91XM9-3;Q91XM9-6;Q91XM9-8;Q91XM9-2;Q91XM9-4;Q91XM9-7;Q91XM9-5	Disks large homolog 2	Dlg2
BASP1	Q91XV3	Brain acid soluble protein 1	Basp1
Ribophorin_I	Q91YQ5	Dolichyl-diphosphooligosaccharide--protein glycosyltransferase subunit 1	Rpn1
Complex1_51K;NADH_4Fe-4S;SLBB	Q91YT0	NADH dehydrogenase [ubiquinone] flavoprotein 1, mitochondrial	Ndufv1
DnaJ;TPR_1	Q91YW3	DnaJ homolog subfamily C member 3	Dnajc3
2-Hacid_dh;2-Hacid_dh_C	Q91Z53	Glyoxylate reductase/hydroxypyruvate reductase	Grhpr
UDPGP	Q91ZJ5;Q91ZJ5-2	UTP--glucose-1-phosphate uridylyltransferase	Ugp2
EGF_CA;Ldl_recept_a;Ldl_recept_b	Q91ZX7	Prolow-density lipoprotein receptor-related protein 1;Low-density lipoprotein receptor-related protein 1 85 kDa subunit;Low-density lipoprotein receptor-related	Lrp1

© 2023. Melachroinou K et al. Published online at http://www.aginganddisease.org/EN/10.14336/AD.2023.0614

		protein 1 515 kDa subunit;Low-density lipoprotein receptor-related protein 1 intracellular domain	
Synuclein	Q91ZZ3;Q9Z0F7	Beta-synuclein	Sncb
ETF_QO	Q921G7	Electron transfer flavoprotein-ubiquinone oxidoreductase, mitochondrial	Etfdh
Transferrin	Q921I1;Q9DBD0;CON__Q29443;CON__Q0IIK2	Serotransferrin	Tf
Ras	Q921J2	GTP-binding protein Rheb	Rheb
DUF1394	Q921M7	Protein FAM49B	Fam49b
Tetraspannin	Q922J6	Tetraspanin-2	Tspan2
Glyco_hydro_18	Q922Q9;Q922Q9-2	Chitinase domain-containing protein 1	Chid1
Thioredoxin	Q922R8	Protein disulfide-isomerase A6	Pdia6
GAF;PDEase_I	Q922S4;Q922S4-2;Q922S4-3	cGMP-dependent 3,5-cyclic phosphodiesterase	Pde2a
CaMKII_AD;Pkinase	Q923T9;Q923T9-3;Q923T9-2	Calcium/calmodulin-dependent protein kinase type II subunit gamma	Camk2g
AA_permease;KCl_Cotrans_1	Q924N4;Q924N4-2	Solute carrier family 12 member 6	Slc12a6
Mtc	Q925N0	Sideroflexin-5	Sfxn5
Transaldolase	Q93092	Transaldolase	Taldo1
adh_short	Q99J47;Q99J47-2	Dehydrogenase/reductase SDR family member 7B	Dhrs7b
Pentaxin	Q99J85	Neuronal pentraxin receptor	Nptxr
7tm_1	Q99JG2	Prosaposin receptor GPR37L1	Gpr37l1
ER_lumen_recept	Q99JH8;Q9CQM2;Q8R1L4	ER lumen protein-retaining receptor 1	Kdelr1
Ras	Q99JI6	Ras-related protein Rap-1b	Rap1b
G_glu_transpept	Q99JP7	Gamma-glutamyltransferase 7;Gamma-glutamyltransferase 7 heavy chain;Gamma-glutamyltransferase 7 light chain	Ggt7
Mtc	Q99JR1	Sideroflexin-1	Sfxn1
PAP2	Q99JY8	Lipid phosphate phosphohydrolase 3	Ppap2b
Actin	Q99JY9	Actin-related protein 3	Actr3
CH;EF-hand_7	Q99K51;Q61233;Q3V0K9	Plastin-3	Pls3
Aminotran_5	Q99K85	Phosphoserine aminotransferase	Psat1
Lactamase_B	Q99KB8;Q99KB8-2	Hydroxyacylglutathione hydrolase, mitochondrial	Hagh
EMP24_GP25L	Q99KF1	Transmembrane emp24 domain-containing protein 9	Tmed9
Aconitase;Aconitase_C	Q99KI0	Aconitate hydratase, mitochondrial	Aco2
	Q99KJ8	Dynactin subunit 2	Dctn2
CTDII;DnaJ	Q99KV1	DnaJ homolog subfamily B member 11	Dnajb11
	Q99KW9	T-cell immunomodulatory protein	Itfg1
CTP_transf_1	Q99L43	Phosphatidate cytidylyltransferase 2	Cds2

© 2023. Melachroinou K et al. Published online at http://www.aginganddisease.org/EN/10.14336/AD.2023.0614

TPR_2	Q99L47	Hsc70-interacting protein	St13
dNK	Q99LC3	NADH dehydrogenase [ubiquinone] 1 alpha subcomplex subunit 10, mitochondrial	Ndufa10
ETF;ETF_alpha	Q99LC5	Electron transfer flavoprotein subunit alpha, mitochondrial	Etfa
PSS	Q99LH2	Phosphatidylserine synthase 1	Ptdss1
Abhydrolase_5	Q99LR1;Q99LR1-2	Monoacylglycerol lipase ABHD12	Abhd12
DJ-1_PfpI	Q99LX0	Protein deglycase DJ-1	Park7
Ndufs5	Q99LY9	NADH dehydrogenase [ubiquinone] iron-sulfur protein 5;NADH dehydrogenase [ubiquinone] iron-sulfur protein 5, N-terminally processed	Ndufs5
Ependymin	Q99M71;Q99M71-2	Mammalian ependymin-related protein 1	Epdr1
C2-set_2;V-set	Q99N28	Cell adhesion molecule 3	Cadm3
Reticulon	Q99P72-1;Q99P72-4	Reticulon-4	Rtn4
I-set	Q99PJ0	Neurotrimin	Ntm
Rho_GDI	Q99PT1	Rho GDP-dissociation inhibitor 1	Arhgdia
ETC_C1_NDUFA5	Q9CPP6	NADH dehydrogenase [ubiquinone] 1 alpha subcomplex subunit 5	Ndufa5
COX6C	Q9CPQ1	Cytochrome c oxidase subunit 6C	Cox6c
ATP-synt_G	Q9CPQ8	ATP synthase subunit g, mitochondrial	Atp5l
	Q9CPS6	Histidine triad nucleotide-binding protein 3	Hint3
UPF0556	Q9CPT4	Myeloid-derived growth factor	Mydgf
Glyoxalase	Q9CPU0	Lactoylglutathione lyase	Glo1
MAPEG	Q9CPU4	Microsomal glutathione S-transferase 3	Mgst3
Glyoxalase_2	Q9CPV4;Q9CPV4-2;Q9CPV4-3	Glyoxalase domain-containing protein 4	Glod4
EGF;F5_F8_type_C;Laminin_G_2	Q9CPW0	Contactin-associated protein-like 2	Cntnap2
Peptidase_M17;Peptidase_M17_N	Q9CPY7;Q9CPY7-2	Cytosol aminopeptidase	Lap3
NDUF_C2	Q9CQ54	NADH dehydrogenase [ubiquinone] 1 subunit C2	Ndufc2
UcrQ	Q9CQ69	Cytochrome b-c1 complex subunit 8	Uqcrq
L51_S25_CI-B8	Q9CQ75	NADH dehydrogenase [ubiquinone] 1 alpha subcomplex subunit 2	Ndufa2
Fer2_3	Q9CQA3	Succinate dehydrogenase [ubiquinone] iron-sulfur subunit, mitochondrial	Sdhb
MitoNEET_N;zf-CDGSH	Q9CQB5	CDGSH iron-sulfur domain-containing protein 2	Cisd2
NDUF_B4	Q9CQC7	NADH dehydrogenase [ubiquinone] 1 beta subcomplex subunit 4	Ndufb4
Ras	Q9CQD1	Ras-related protein Rab-5A	Rab5a
Prenylcys_lyase	Q9CQF9	Prenylcysteine oxidase	Pcyox1

© 2023. Melachroinou K et al. Published online at http://www.aginganddisease.org/EN/10.14336/AD.2023.0614

SUPPLEMENTARY DATA

NDUF_B5	Q9CQH3	NADH dehydrogenase [ubiquinone] 1 beta subcomplex subunit 5, mitochondrial	Ndufb5
Cofilin_ADF	Q9CQI3;Q9ERL7	Glia maturation factor beta	Gmfb
Cofilin_ADF	Q9CQI6	Coactosin-like protein	Cotl1
Complex1_LYR	Q9CQJ8	NADH dehydrogenase [ubiquinone] 1 beta subcomplex subunit 9	Ndufb9
Mt_ATP-synt_B	Q9CQQ7	ATP synthase F(0) complex subunit B1, mitochondrial	Atp5f1
4HBT	Q9CQR4	Acyl-coenzyme A thioesterase 13;Acyl-coenzyme A thioesterase 13, N-terminally processed	Acot13
Atg8	Q9CQV6	Microtubule-associated proteins 1A/1B light chain 3B	Map1lc3b
14/3/2003	Q9CQV8;Q9CQV8-2	14-3-3 protein beta/alpha;14-3-3 protein beta/alpha, N-terminally processed	Ywhab
Arf	Q9CQW2	ADP-ribosylation factor-like protein 8B	Arl8b
Claudin_2	Q9CQX5	Claudin domain-containing protein 1	Cldnd1
Complex1_LYR	Q9CQZ5	NADH dehydrogenase [ubiquinone] 1 alpha subcomplex subunit 6	Ndufa6
NDUF_B12	Q9CQZ6	NADH dehydrogenase [ubiquinone] 1 beta subcomplex subunit 3	Ndufb3
Pro_isomerase;TPR_7	Q9CR16	Peptidyl-prolyl cis-trans isomerase D	Ppid
NDUF_B7	Q9CR61	NADH dehydrogenase [ubiquinone] 1 beta subcomplex subunit 7	Ndufb7
Mito_carr	Q9CR62	Mitochondrial 2-oxoglutarate/malate carrier protein	Slc25a11
DUF1242	Q9CR64;Q9CR64-2	Protein kish-A	Tmem167a
UPF0121	Q9CR67	Transmembrane protein 33	Tmem33
Rieske;Ubiq-Cytc-red_N;UCR_TM	Q9CR68	Cytochrome b-c1 complex subunit Rieske, mitochondrial;Cytochrome b-c1 complex subunit 11	Uqcrfs1
COPIIcoated_ERV	Q9CR89;Q9CR89-2	Endoplasmic reticulum-Golgi intermediate compartment protein 2	Ergic2
p25-alpha	Q9CRB6	Tubulin polymerization-promoting protein family member 3	Tppp3
TPR_2	Q9CRD2	ER membrane protein complex subunit 2	Emc2
GDPD	Q9CRY7	Glycerophosphodiester phosphodiesterase domain-containing protein 1	Gdpd1
Laminin_G_2;Syndecan	Q9CS84;Q9CS84-4;Q9CS84-3;Q9CS84-2;Q9CS84-5;P0DI97;Q9CS84-6	Neurexin-1	Nrxn1
P34-Arc	Q9CVB6	Actin-related protein 2/3 complex subunit 2	Arpc2
Amidinotransf	Q9CWS0	N(G),N(G)-dimethylarginine dimethylaminohydrolase 1	Ddah1
	Q9CWZ7	Gamma-soluble NSF attachment protein	Napg
EMP24_GP25L	Q9CXE7	Transmembrane emp24 domain-containing protein 5	Tmed5

© 2023. Melachroinou K et al. Published online at http://www.aginganddisease.org/EN/10.14336/AD.2023.0614

SUPPLEMENTARY DATA

Armet	Q9CXI5	Mesencephalic astrocyte-derived neurotrophic factor	Manf
Peptidase_C13	Q9CXY9	GPI-anchor transamidase	Pigk
Steroid_dh	Q9CY27	Very-long-chain enoyl-CoA reductase	Tecr
TRAP_alpha	Q9CY50	Translocon-associated protein subunit alpha	Ssr1
	Q9CYH2	Redox-regulatory protein FAM213A	Fam213a
SPC25	Q9CYN2	Signal peptidase complex subunit 2	Spcs2
Renin_r	Q9CYN9	Renin receptor	Atp6ap2
CAP_C;CAP_N	Q9CYT6	Adenylyl cyclase-associated protein 2	Cap2
Peptidase_M16;Peptidase_M16_C	Q9CZ13;Q9CXT8	Cytochrome b-c1 complex subunit 1, mitochondrial	Uqcrc1
MMR_HSR1;YchF-GTPase_C	Q9CZ30;Q9CZ30-2	Obg-like ATPase 1	Ola1
SEP;UBX	Q9CZ44;Q9CZ44-3;Q9CZ44-2	NSFL1 cofactor p47	Nsfl1c
Sdh_cyt	Q9CZB0	Succinate dehydrogenase cytochrome b560 subunit, mitochondrial	Sdhc
Peptidase_C69	Q9CZC8	Secernin-1	Scrn1
Porin_3	Q9CZR3	Mitochondrial import receptor subunit TOM40B	Tomm40l
Citrate_synt	Q9CZU6	Citrate synthase, mitochondrial	Cs
UQ_con	Q9CZY3;Q9D2M8-2;Q9CZY3-2	Ubiquitin-conjugating enzyme E2 variant 1;Ubiquitin-conjugating enzyme E2 variant 2	Ube2v1;Ube2v2
MPC	Q9D023	Mitochondrial pyruvate carrier 2	Mpc2
Lectin_leg-like	Q9D0F3	Protein ERGIC-53	Lman1
PGM_PMM_I;PGM_PMM_II;PGM_PMM_III;PGM_PMM_IV	Q9D0F9	Phosphoglucomutase-1	Pgm1
CoA_trans	Q9D0K2	Succinyl-CoA:3-ketoacid coenzyme A transferase 1, mitochondrial	Oxct1
Cytochrom_C1	Q9D0M3;Q9D0M3-2	Cytochrome c1, heme protein, mitochondrial	Cyc1
Dynein_light	Q9D0M5	Dynein light chain 2, cytoplasmic	Dynll2
Serpin	Q9D154;Q8VHP7;Q5SV42;Q9JK88	Leukocyte elastase inhibitor A	Serpinb1a
ATP1G1_PLM_MAT8	Q9D164-2;Q9D164	FXYD domain-containing ion transport regulator 6	Fxyd6
DJ-1_PfpI	Q9D172	ES1 protein homolog, mitochondrial	D10Jhu81e
M20_dimer;Peptidase_M20	Q9D1A2	Cytosolic non-specific dipeptidase	Cndp2
EMP24_GP25L	Q9D1D4;Q9D1D4-2	Transmembrane emp24 domain-containing protein 10	Tmed10
Ras	Q9D1G1	Ras-related protein Rab-1B	Rab1b
MBOAT	Q9D1G3	Protein-cysteine N-palmitoyltransferase HHAT-like protein	Hhatl
LRR_1;LRR_8	Q9D1G5	Leucine-rich repeat-containing protein 57	Lrrc57
ATP-synt_F	Q9D1K2	V-type proton ATPase subunit F	Atp6v1f
DPM3	Q9D1Q4	Dolichol-phosphate mannosyltransferase subunit 3	Dpm3
Thioredoxin	Q9D1Q6	Endoplasmic reticulum resident protein 44	Erp44

© 2023. Melachroinou K et al. Published online at http://www.aginganddisease.org/EN/10.14336/AD.2023.0614

I-set;LRR_1;LRR_8	Q9D1T0	Leucine-rich repeat and immunoglobulin-like domain-containing nogo receptor-interacting protein 1	Lingo1
UPF0220	Q9D1X9	Transmembrane protein 50B	Tmem50b
2-oxoacid_dh;Biotin_lipoyl	Q9D2G2;Q9D2G2-2	Dihydrolipoyllysine-residue succinyltransferase component of 2-oxoglutarate dehydrogenase complex, mitochondrial	Dlst
Abhydrolase_1;EHN	Q9D379	Epoxide hydrolase 1	Ephx1
Lamp	Q9D387	Lysosome-associated membrane glycoprotein 5	Lamp5
Tweety	Q9D3A9;Q9D3A9-4;Q9D3A9-5	Protein tweety homolog 1	Ttyh1
PTPLA	Q9D3B1	Very-long-chain (3R)-3-hydroxyacyl-CoA dehydratase 2	Hacd2
Erf4	Q9D428	Golgin subfamily A member 7B	GOLGA7B
Acyltransferase	Q9D517	1-acyl-sn-glycerol-3-phosphate acyltransferase gamma	Agpat3
Neur_chan_LBD;Neur_chan_memb	Q9D6F4	Gamma-aminobutyric acid receptor subunit alpha-4	Gabra4
Tubulin;Tubulin_C	Q9D6F9	Tubulin beta-4A chain	Tubb4a
NDUF_B8	Q9D6J5	NADH dehydrogenase [ubiquinone] 1 beta subcomplex subunit 8, mitochondrial	Ndufb8
Mito_carr	Q9D6M3;Q9DB41;Q9DB41-2	Mitochondrial glutamate carrier 1;Mitochondrial glutamate carrier 2	Slc25a22;Slc25a18
Iso_dh	Q9D6R2;Q9D6R2-2	Isocitrate dehydrogenase [NAD] subunit alpha, mitochondrial	Idh3a
S_100	Q9D708		S100a16
Thioredoxin	Q9D710	Thioredoxin-related transmembrane protein 2	Tmx2
Pribosyl_synth;Pribosyltran_N	Q9D7G0;Q9CS42	Ribose-phosphate pyrophosphokinase 1;Ribose-phosphate pyrophosphokinase 2	Prps1;Prps2
Str_synth	Q9D7N9	Adipocyte plasma membrane-associated protein	Apmap
DSPc	Q9D7X3	Dual specificity protein phosphatase 3	Dusp3
Pyrophosphatase	Q9D819	Inorganic pyrophosphatase	Ppa1
UCR_14kD	Q9D855	Cytochrome b-c1 complex subunit 7	Uqcrb
P16-Arc	Q9D898	Actin-related protein 2/3 complex subunit 5-like protein	Arpc5l
Far-17a_AIG1	Q9D8B1;Q9D8B1-1	Androgen-induced gene 1 protein	Aig1
Snf7	Q9D8B3;Q9D7F7	Charged multivesicular body protein 4b	Chmp4b
Tim17	Q9D8B4	NADH dehydrogenase [ubiquinone] 1 alpha subcomplex subunit 11	Ndufa11
I-set;V-set	Q9D8B7	Junctional adhesion molecule C	Jam3
EF1G;GST_C;GST_N	Q9D8N0	Elongation factor 1-gamma	Eef1g
Peptidase_A22B	Q9D8V0-4;Q9D8V0;Q9D8V0-3	Minor histocompatibility antigen H13	Hm13

© 2023. Melachroinou K et al. Published online at http://www.aginganddisease.org/EN/10.14336/AD.2023.0614

Peptidase_S24	Q9D8V7	Signal peptidase complex catalytic subunit SEC11C	Sec11c
PCI	Q9D8W5	26S proteasome non-ATPase regulatory subunit 12	Psmd12
EF-hand_7	Q9D8Y0;Q9D4J1	EF-hand domain-containing protein D2	Efhd2
Ocnus	Q9DAK9	14 kDa phosphohistidine phosphatase	Phpt1
G-gamma	Q9DAS9	Guanine nucleotide-binding protein G(I)/G(S)/G(O) subunit gamma-12	Gng12
	Q9DB05	Alpha-soluble NSF attachment protein	Napa
OSCP	Q9DB20	ATP synthase subunit O, mitochondrial	Atp5o
BACK;BTB	Q9DB72	BTB/POZ domain-containing protein 17	Btbd17
Peptidase_M16;Peptidase_M16_C	Q9DB77	Cytochrome b-c1 complex subunit 2, mitochondrial	Uqcrc2
Aldedh	Q9DBF1;Q9DBF1-2	Alpha-aminoadipic semialdehyde dehydrogenase	Aldh7a1
Adaptin_N;Alpha_adaptinC2;B2-adapt-app_C	Q9DBG3;Q9DBG3-2	AP-2 complex subunit beta	Ap2b1
Ribophorin_II	Q9DBG6	Dolichyl-diphosphooligosaccharide--protein glycosyltransferase subunit 2	Rpn2
Lectin_leg-like	Q9DBH5	Vesicular integral-membrane protein VIP36	Lman2
His_Phos_1	Q9DBJ1;O70250	Phosphoglycerate mutase 1	Pgam1
	Q9DBP5	UMP-CMP kinase	Cmpk1
DUF1625	Q9DBS1	Transmembrane protein 43	Tmem43
hSac2	Q9DBS2	Tumor protein p63-regulated gene 1-like protein	Tprg1l
AMOP;Somatomedin_B;Sushi;VWD	Q9DBX3;Q9DBX3-3;Q9DBX3-2	Sushi domain-containing protein 2	Susd2
COPIIcoated_ERV	Q9DC16	Endoplasmic reticulum-Golgi intermediate compartment protein 1	Ergic1
DnaJ;Thioredoxin	Q9DC23	DnaJ homolog subfamily C member 10	Dnajc10
G-alpha	Q9DC51	Guanine nucleotide-binding protein G(k) subunit alpha	Gnai3
NmrA	Q9DC69	NADH dehydrogenase [ubiquinone] 1 alpha subcomplex subunit 9, mitochondrial	Ndufa9
Oxidored_q6	Q9DC70	NADH dehydrogenase [ubiquinone] iron-sulfur protein 7, mitochondrial	Ndufs7
6PGD;NAD_binding_2	Q9DCD0	6-phosphogluconate dehydrogenase, decarboxylating	Pgd
CHCH	Q9DCJ5	NADH dehydrogenase [ubiquinone] 1 alpha subcomplex subunit 8	Ndufa8
AIRC;SAICAR_synt	Q9DCL9	Multifunctional protein ADE2;Phosphoribosylaminoimidazole-succinocarboxamide synthase;Phosphoribosylaminoimidazole carboxylase	Paics
Aa_trans	Q9DCP2	Sodium-coupled neutral amino acid transporter 3	Slc38a3
NDUFB10	Q9DCS9	NADH dehydrogenase [ubiquinone] 1 beta subcomplex subunit 10	Ndufb10

© 2023. Melachroinou K et al. Published online at http://www.aginganddisease.org/EN/10.14336/AD.2023.0614

SUPPLEMENTARY DATA

Complex1_30kDa	Q9DCT2	NADH dehydrogenase [ubiquinone] iron-sulfur protein 3, mitochondrial	Ndufs3
MIR	Q9DCT5	Stromal cell-derived factor 2	Sdf2
Mt_ATP-synt_D	Q9DCX2	ATP synthase subunit d, mitochondrial	Atp5h
ApoO	Q9DCZ4;Q9DCZ4-2;Q9DCZ4-3	Apolipoprotein O	Apoo
Tyr_Deacylase	Q9DD18;Q9DD18-2	D-tyrosyl-tRNA(Tyr) deacylase 1	Dtd1
DUF2012	Q9EP72	ER membrane protein complex subunit 7	Emc7
Beta-lactamase	Q9EP89	Serine beta-lactamase-like protein LACTB, mitochondrial	Lactb
Cadherin	Q9EPL2;Q9EPL2-2	Calsyntenin-1;Soluble Alc-alpha;CTF1-alpha	Clstn1
Cse1;IBN_N	Q9EPL8	Importin-7	Ipo7
PKD	Q9EPR5	VPS10 domain-containing receptor SorCS2	Sorcs2
adh_short	Q9EQ06;Q9EQ06-2;Q8VCR2;Q8VCR2-2	Estradiol 17-beta-dehydrogenase 11	Hsd17b11
Amidohydro_1	Q9EQF6	Dihydropyrimidinase-related protein 5	Dpysl5
Phosphodiest	Q9EQG7	Ectonucleotide pyrophosphatase/phosphodiesterase family member 5	Enpp5
Vps35	Q9EQH3	Vacuolar protein sorting-associated protein 35	Vps35
NAP	Q9EQU5;Q9EQU5-2	Protein SET	Set
SNARE	Q9ER00	Syntaxin-12	Stx12
Kazal_2;SPARC_Ca_bdg;Thyroglobulin_1	Q9ER58	Testican-2	Spock2
Tubulin;Tubulin_C	Q9ERD7	Tubulin beta-3 chain	Tubb3
Mesd	Q9ERE7	LDLR chaperone MESD	Mesdc2
Sep15_SelM	Q9ERR7	15 kDa selenoprotein	Σεπ-15
GRIM-19	Q9ERS2	NADH dehydrogenase [ubiquinone] 1 alpha subcomplex subunit 13	Ndufa13
PTR2	Q9ES07	Solute carrier family 15 member 2	Slc15a2
Glyco_transf_64	Q9ES89	Exostosin-like 2	Extl2
Reticulon	Q9ES97-3;Q9ES97;Q9ES97-4;Q9ES97-5;Q9ES97-2	Reticulon-3	Rtn3
EMP70	Q9ET30	Transmembrane 9 superfamily member 3	Tm9sf3
AAA_5;AAA_8;DHC_N1;DHC_N2;Dynein_heavy;MT	Q9JHU4	Cytoplasmic dynein 1 heavy chain 1	Dync1h1
Inos-1-P_synth;NAD_binding_5	Q9JHU9	Inositol-3-phosphate synthase 1	Isyna1
ANKH	Q9JHZ2	Progressive ankylosis protein	Ankh
EPTP;LRR_8	Q9JIA1	Leucine-rich glioma-inactivated protein 1	Lgi1
Methyltransf_31	Q9JIF0;Q9JIF0-3;Q9JIF0-2;Q6PAK3	Protein arginine N-methyltransferase 1;Protein arginine N-methyltransferase 8	Prmt1;Prmt8
Aldo_ket_red	Q9JII6	Alcohol dehydrogenase [NADP(+)]	Akr1a1
MFS_1;Pentapeptide_4;Sugar_tr	Q9JIS5	Synaptic vesicle glycoprotein 2A	Sv2a
Scramblase	Q9JIZ9	Phospholipid scramblase 3	Plscr3

© 2023. Melachroinou K et al. Published online at http://www.aginganddisease.org/EN/10.14336/AD.2023.0614

Glyco_transf_7C;Glycos_transf_2;Ricin_B_lectin	Q9JJ61;Q8BVG5-2;Q8BVG5	Polypeptide N-acetylgalactosaminyltransferase 16	Galnt16
MBOAT	Q9JJJ7-4;Q9JJJ7-3;Q9JJJ7-2;Q9JJJ7;Q9JJJ7-5	Protein-serine O-palmitoleoyltransferase porcupine	Porcn
LANC_like	Q9JJK2	LanC-like protein 2	Lancl2
Tmemb_9	Q9JJR8	Transmembrane protein 9B	Tmem9b
Profilin	Q9JJV2;Q9JJV2-3;Q9JJV2-2	Profilin-2	Pfn2
PMP22_Claudin	Q9JJV5	Voltage-dependent calcium channel gamma-3 subunit	Cacng3
Exo_endo_phos	Q9JJY3	Sphingomyelin phosphodiesterase 3	Smpd3
Peptidase_C12	Q9JKB1;P58321	Ubiquitin carboxyl-terminal hydrolase isozyme L3;Ubiquitin carboxyl-terminal hydrolase isozyme L4	Uchl3;Uchl4
SCAMP	Q9JKD3	Secretory carrier-associated membrane protein 5	Scamp5
C2-set_2;V-set	Q9JKF6	Nectin-1	Pvrl1
Cation_efflux	Q9JKN1	Zinc transporter 7	Slc30a7
HSP70	Q9JKR6	Hypoxia up-regulated protein 1	Hyou1
Reticulon	Q9JKW0	ADP-ribosylation factor-like protein 6-interacting protein 1	Arl6ip1
GDPD	Q9JL56	Glycerophosphodiester phosphodiesterase 1	Gde1
GLTP	Q9JL62	Glycolipid transfer protein	Gltp
Guanylate_kin;L27;PDZ;SH3_2	Q9JLB0;Q9JLB0-2	MAGUK p55 subfamily member 6	Mpp6
DCX;Pkinase	Q9JLM8;Q9JLM8-2	Serine/threonine-protein kinase DCLK1	Dclk1
Plug_translocon;SecY	Q9JLR1	Protein transport protein Sec61 subunit alpha isoform 2	Sec61a2
Cullin;Cullin_Nedd8	Q9JLV5	Cullin-3	Cul3
ECH	Q9JLZ3;Q9JLZ3-3;Q9JLZ3-2	Methylglutaconyl-CoA hydratase, mitochondrial	Auh
NT5C	Q9JM14	5(3)-deoxyribonucleotidase, cytosolic type	Nt5c
P21-Arc	Q9JM76	Actin-related protein 2/3 complex subunit 3	Arpc3
UCH	Q9JMA1	Ubiquitin carboxyl-terminal hydrolase 14	Usp14
Adaptin_N;AP3B1_C	Q9JME5	AP-3 complex subunit beta-2	Ap3b2
Pyr_redox;Pyr_redox_2;Pyr_redox_dim	Q9JMH6;Q9JMH6-2	Thioredoxin reductase 1, cytoplasmic	Txnrd1
Ras	Q9QUI0;Q62159	Transforming protein RhoA;Rho-related GTP-binding protein RhoC	Rhoa;Rhoc
Proteasome;Proteasome_A_N	Q9QUM9	Proteasome subunit alpha type-6	Psma6
V-set;Xlink	Q9QUP5	Hyaluronan and proteoglycan link protein 1	Hapln1
Peptidase_S9;Peptidase_S9_N	Q9QUR6	Prolyl endopeptidase	Prep
DUF3456	Q9QXT0	Protein canopy homolog 2	Cnpy2
ProSAAS	Q9QXV0	ProSAAS;KEP;Big SAAS;Little SAAS;Big PEN-LEN;PEN;PEN-20;PEN-19;Little LEN;Big LEN	Pcsk1n

© 2023. Melachroinou K et al. Published online at http://www.aginganddisease.org/EN/10.14336/AD.2023.0614

SUPPLEMENTARY DATA

AA_permease_2	Q9QXW9	Large neutral amino acids transporter small subunit 2	Slc7a8
	Q9QXY6;Q9WVK4	EH domain-containing protein 3	Ehd3
7tm_1	Q9QY42;Q9QY42-2;Q9QY42-3	Prosaposin receptor GPR37	Gpr37
Porin_3	Q9QYA2	Mitochondrial import receptor subunit TOM40 homolog	Tomm40
Ndr	Q9QYF9	Protein NDRG3	Ndrg3
Ndr	Q9QYG0;Q9QYG0-2	Protein NDRG2	Ndrg2
	Q9QYR6;Q9QYR6-2	Microtubule-associated protein 1A;MAP1A heavy chain;MAP1 light chain LC2	Map1a
7tm_3;ANF_receptor;NCD3G	Q9QYS2	Metabotropic glutamate receptor 3	Grm3
C2;CUE	Q9QZ06	Toll-interacting protein	Tollip
Metallophos_2	Q9QZ88;Q9QZ88-2	Vacuolar protein sorting-associated protein 29	Vps29
Plexin_cytopl;PSI;TIG	Q9QZC2	Plexin-C1	Plxnc1
Glypican	Q9QZF2	Glypican-1;Secreted glypican-1	Gpc1
Serinc	Q9QZI8	Serine incorporator 1	Serinc1
UBA;ubiquitin	Q9QZM0	Ubiquilin-2	Ubqln2
PALP	Q9QZX7;Q9QZX7-2	Serine racemase	Srr
ATP_Ca_trans_C;Cation_ATPase_C;Cation_ATPase_N;E1-E2_ATPase;Hydrolase	Q9R0K7	Plasma membrane calcium-transporting ATPase 2	Atp2b2
C2	Q9R0N3	Synaptotagmin-11	Syt11
C2	Q9R0N5	Synaptotagmin-5	Syt5
C2	Q9R0N7;Q9R0N7-3;Q9R0N7-2;Q9R0N7-4	Synaptotagmin-7	Syt7
C2	Q9R0N8;Q9R0N8-2	Synaptotagmin-6	Syt6
Esterase	Q9R0P3	S-formylglutathione hydrolase	Esd
Cofilin_ADF	Q9R0P5	Destrin	Dstn
Peptidase_S24	Q9R0P6	Signal peptidase complex catalytic subunit SEC11A	Sec11a
Peptidase_C12	Q9R0P9	Ubiquitin carboxyl-terminal hydrolase isozyme L1	Uchl1
EMP24_GP25L	Q9R0Q3	Transmembrane emp24 domain-containing protein 2	Tmed2
WD40	Q9R0Q6;Q9WV32	Actin-related protein 2/3 complex subunit 1A	Arpc1a
CS	Q9R0Q7	Prostaglandin E synthase 3	Ptges3
PQ-loop	Q9R0Q9	Mannose-P-dolichol utilization defect 1 protein	Mpdu1
	Q9R0Y5;Q9R0Y5-2	Adenylate kinase isoenzyme 1	Ak1
Amidohydro_1	Q9R111	Guanine deaminase	Gda
3Beta_HSD	Q9R1J0	Sterol-4-alpha-carboxylate 3-dehydrogenase, decarboxylating	Nsdhl
Proteasome;Proteasome_A_N	Q9R1P0	Proteasome subunit alpha type-4	Psma4
Proteasome	Q9R1P1	Proteasome subunit beta type-3	Psmb3
Proteasome;Proteasome_A_N	Q9R1P4	Proteasome subunit alpha type-1	Psma1
Calponin;CH	Q9R1Q8;Q9WVA4;P37804	Transgelin-3	Tagln3

© 2023. Melachroinou K et al. Published online at http://www.aginganddisease.org/EN/10.14336/AD.2023.0614

SUPPLEMENTARY DATA

ATP-synt_S1	Q9R1Q9	V-type proton ATPase subunit S1	Atp6ap1
Septin	Q9R1T4;Q9R1T4-2;Q9R1T4-3	Septin-6	Σεπ-06
ADAM_CR;Disintegrin;Pep_M12B_propep;Reprolysin	Q9R1V4	Disintegrin and metalloproteinase domain-containing protein 11	Adam11
ADAM_CR;Disintegrin;Pep_M12B_propep;Reprolysin	Q9R1V6;Q9R1V6-10;Q9R1V6-11;Q9R1V6-14;Q9R1V6-8;Q9R1V6-15;Q9R1V6-13;Q9R1V6-7;Q9R1V6-6;Q9R1V6-2;Q9R1V6-4;Q9R1V6-9;Q9R1V6-5;Q9R1V6-1;Q9R1V6-20;Q9R1V6-17;Q9R1V6-19;Q9R1V6-12;Q9R1V6-16;Q9R1V6-21;Q9R1V6-18	Disintegrin and metalloproteinase domain-containing protein 22	Adam22
ADAM_CR;Disintegrin;Pep_M12B_propep;Reprolysin	Q9R1V7;Q9R1V7-4;Q9R1V7-3;Q9R1V7-2	Disintegrin and metalloproteinase domain-containing protein 23	Adam23
Gaa1	Q9WTK3	Glycosylphosphatidylinositol anchor attachment 1 protein	Gpaa1
Ank_2;Ion_trans	Q9WTR1	Transient receptor potential cation channel subfamily V member 2	Trpv2
Cadherin;Cadherin_pro	Q9WTR5	Cadherin-13	Cdh13
AA_permease_2	Q9WTR6	Cystine/glutamate transporter	Slc7a11
	Q9WTT4	V-type proton ATPase subunit G 2	Atp6v1g2
Skp1;Skp1_POZ	Q9WTX5	S-phase kinase-associated protein 1	Skp1
ALIX_LYPXL_bnd;BRO1	Q9WU78;Q9WU78-3;Q9WU78-2	Programmed cell death 6-interacting protein	Pdcd6ip
PFK	Q9WUA3;Q9WUA3-2	ATP-dependent 6-phosphofructokinase, platelet type	Pfkp
Phosphorylase	Q9WUB3;Q9ET01	Glycogen phosphorylase, muscle form	Pygm
UPAR_LY6	Q9WUC3	Lymphocyte antigen 6H	Ly6h
DUF1899;DUF1900;WD40	Q9WUM3	Coronin-1B	Coro1b
DUF1899;DUF1900;WD40	Q9WUM4	Coronin-1C	Coro1c
CoA_binding;Ligase_CoA	Q9WUM5	Succinyl-CoA ligase [ADP/GDP-forming] subunit alpha, mitochondrial	Suclg1
WD40	Q9WUQ2	Prolactin regulatory element-binding protein	Preb
7tm_3;ANF_receptor;Sushi	Q9WV18;Q9WV18-2	Gamma-aminobutyric acid type B receptor subunit 1	Gabbr1
Guanylate_kin;L27;PDZ;SH3_2	Q9WV34;Q9WV34-2	MAGUK p55 subfamily member 2	Mpp2
CBAH;NAAA-beta	Q9WV54	Acid ceramidase;Acid ceramidase subunit alpha;Acid ceramidase subunit beta	Asah1
V-set	Q9WV91	Prostaglandin F2 receptor negative regulator	Ptgfrn
4_1_CTD;FA;FERM_C;FERM_M;FERM_N;SAB	Q9WV92;Q9WV92-3;Q9WV92-2;Q9WV92-6;Q9WV92-4;Q9WV92-5;Q9WV92-8;Q9WV92-7;P48193;P48193-2	Band 4.1-like protein 3;Band 4.1-like protein 3, N-terminally processed	Epb41l3
FCH;SH3_9	Q9WVE8	Protein kinase C and casein kinase substrate in neurons protein 2	Pacsin2
	Q9Z0E0;Q9Z0E0-2	Neurochondrin	Ncdn
Paralemmin	Q9Z0P4;Q9Z0P4-2	Paralemmin-1	Palm

© 2023. Melachroinou K et al. Published online at http://www.aginganddisease.org/EN/10.14336/AD.2023.0614

SUPPLEMENTARY DATA

Inositol_P	Q9Z0S1	3(2),5-bisphosphate nucleotidase 1	Bpnt1
AIF_C;Pyr_redox;Pyr_redox_2	Q9Z0X1;Q9Z0X1-2	Apoptosis-inducing factor 1, mitochondrial	Aifm1
AA_permease_2	Q9Z127;Q8BGK6;Q8BGK6-2	Large neutral amino acids transporter small subunit 1	Slc7a5
C2;Copine	Q9Z140	Copine-6	Cpne6
C2;DUF1154;EF-hand_like;PI-PLC-X;PI-PLC-Y;PLC-beta_C	Q9Z1B3;Q9Z1B3-3;Q9Z1B3-2	1-phosphatidylinositol 4,5-bisphosphate phosphodiesterase beta-1	Plcb1
V-ATPase_C	Q9Z1G3;Q99L60;Q99L60-3;Q99L60-2	V-type proton ATPase subunit C 1	Atp6v1c1
V_ATPase_I	Q9Z1G4;Q9Z1G4-3;Q9Z1G4-2;Q920R6;P15920-2	V-type proton ATPase 116 kDa subunit a isoform 1	Atp6v0a1
Cache_1;VWA_N	Q9Z1L5	Voltage-dependent calcium channel subunit alpha-2/delta-3;Voltage-dependent calcium channel subunit alpha-2-3;Voltage-dependent calcium channel subunit delta-3	Cacna2d3
DEAD;Helicase_C	Q9Z1N5;Q8VDW0;Q8VDW0-2	Spliceosome RNA helicase Ddx39b	Ddx39b
Abhydrolase_5	Q9Z1Q2;Q80YU0	Abhydrolase domain-containing protein 16A	Abhd16a
Septin	Q9Z1S5;Q9Z1S5-2	Neuronal-specific septin-3	septin3
WD40	Q9Z1Z2	Serine-threonine kinase receptor-associated protein	Strap
DPPIV_N;Peptidase_S9	Q9Z218	Dipeptidyl aminopeptidase-like protein 6	Dpp6
BTK;C2;PH;RasGAP	Q9Z268	RasGAP-activating-like protein 1	Rasal1
fn2;Sel1	Q9Z2G6;Q9Z2G6-2	Protein sel-1 homolog 1	Sel1l
DEP;G-gamma;RGS	Q9Z2H2	Regulator of G-protein signaling 6	Rgs6
4_1_CTD;FA;FERM_C;FERM_M;FERM_N;SAB	Q9Z2H5;Q9Z2H5-3;Q9Z2H5-2	Band 4.1-like protein 1	Epb41l1
ATP-grasp_2;Ligase_CoA	Q9Z2I9	Succinyl-CoA ligase [ADP-forming] subunit beta, mitochondrial	Sucla2
LCM;Septin	Q9Z2Q6;P42209;Q8BYR1;Q8BYR1-2	Septin-5	septin5
Proteasome;Proteasome_A_N	Q9Z2U0;Q9CWH6	Proteasome subunit alpha type-7	Psma7
Proteasome;Proteasome_A_N	Q9Z2U1	Proteasome subunit alpha type-5	Psma5
Peptidase_M18	Q9Z2W0	Aspartyl aminopeptidase	Dnpep
ANF_receptor;Lig_chan;Lig_chan-Glu_bd	Q9Z2W8	Glutamate receptor 4	Gria4
ANF_receptor;Lig_chan;Lig_chan-Glu_bd	Q9Z2W9	Glutamate receptor 3	Gria3

© 2023. Melachroinou K et al. Published online at http://www.aginganddisease.org/EN/10.14336/AD.2023.0614

Supplementary Table 2. DAVID Platform: Exosome-related proteins present in our dataset.

Official gene symbol	Gene name	Species
Cnp	2',3'-cyclic nucleotide 3' phosphodiesterase (Cnp)	Mus musculus
Phgdh	3-phosphoglycerate dehydrogenase (Phgdh)	Mus musculus
Abat	4-aminobutyrate aminotransferase (Abat)	Mus musculus
Nt5e	5' nucleotidase, ecto (Nt5e)	Mus musculus
Nt5c	5',3'-nucleotidase, cytosolic (Nt5c)	Mus musculus
Arf1	ADP-ribosylation factor 1(Arf1)	Mus musculus
Arf3	ADP-ribosylation factor 3(Arf3)	Mus musculus
Arf5	ADP-ribosylation factor 5(Arf5)	Mus musculus
Arf6	ADP-ribosylation factor 6(Arf6)	Mus musculus
Arl8a	ADP-ribosylation factor-like 8A(Arl8a)	Mus musculus
Arl8b	ADP-ribosylation factor-like 8B(Arl8b)	Mus musculus
Ahsa1	AHA1, activator of heat shock protein ATPase 1(Ahsa1)	Mus musculus
Actr1b	ARP1 actin-related protein 1B, centractin beta(Actr1b)	Mus musculus
Actr2	ARP2 actin-related protein 2(Actr2)	Mus musculus
Actr3	ARP3 actin-related protein 3(Actr3)	Mus musculus
Acly	ATP citrate lyase(Acly)	Mus musculus
Atp5b	ATP synthase, H+ transporting mitochondrial F1 complex, beta subunit(Atp5b)	Mus musculus
Atp5f1	ATP synthase, H+ transporting, mitochondrial F0 complex, subunit B1(Atp5f1)	Mus musculus
Atp5h	ATP synthase, H+ transporting, mitochondrial F0 complex, subunit D(Atp5h)	Mus musculus
Atp5j2	ATP synthase, H+ transporting, mitochondrial F0 complex, subunit F2(Atp5j2)	Mus musculus
Atp5l	ATP synthase, H+ transporting, mitochondrial F0 complex, subunit G(Atp5l)	Mus musculus
Atp5o	ATP synthase, H+ transporting, mitochondrial F1 complex, O subunit(Atp5o)	Mus musculus
Atp5a1	ATP synthase, H+ transporting, mitochondrial F1 complex, alpha subunit 1(Atp5a1)	Mus musculus
Atp5c1	ATP synthase, H+ transporting, mitochondrial F1 complex, gamma polypeptide 1(Atp5c1)	Mus musculus
Abcb1a	ATP-binding cassette, sub-family B (MDR/TAP), member 1A(Abcb1a)	Mus musculus
Atp2b1	ATPase, Ca++ transporting, plasma membrane 1(Atp2b1)	Mus musculus
Atp2b2	ATPase, Ca++ transporting, plasma membrane 2(Atp2b2)	Mus musculus
Atp6v0a1	ATPase, H+ transporting, lysosomal V0 subunit A1(Atp6v0a1)	Mus musculus
Atp6v0c	ATPase, H+ transporting, lysosomal V0 subunit C(Atp6v0c)	Mus musculus
Atp6v0d1	ATPase, H+ transporting, lysosomal V0 subunit D1(Atp6v0d1)	Mus musculus
Atp6v1a	ATPase, H+ transporting, lysosomal V1 subunit A(Atp6v1a)	Mus musculus
Atp6v1b2	ATPase, H+ transporting, lysosomal V1 subunit B2(Atp6v1b2)	Mus musculus
Atp6v1c1	ATPase, H+ transporting, lysosomal V1 subunit C1(Atp6v1c1)	Mus musculus
Atp6v1d	ATPase, H+ transporting, lysosomal V1 subunit D(Atp6v1d)	Mus musculus

© 2023. Melachroinou K et al. Published online at http://www.aginganddisease.org/EN/10.14336/AD.2023.0614

SUPPLEMENTARY DATA

Atp6v1e1	ATPase, H+ transporting, lysosomal V1 subunit E1(Atp6v1e1)	Mus musculus
Atp6v1f	ATPase, H+ transporting, lysosomal V1 subunit F(Atp6v1f)	Mus musculus
Atp6v1h	ATPase, H+ transporting, lysosomal V1 subunit H(Atp6v1h)	Mus musculus
Atp6ap1	ATPase, H+ transporting, lysosomal accessory protein 1(Atp6ap1)	Mus musculus
Atp1a1	ATPase, Na+/K+ transporting, alpha 1 polypeptide(Atp1a1)	Mus musculus
Atp1b1	ATPase, Na+/K+ transporting, beta 1 polypeptide(Atp1b1)	Mus musculus
Atp1b3	ATPase, Na+/K+ transporting, beta 3 polypeptide(Atp1b3)	Mus musculus
Atp8a1	ATPase, aminophospholipid transporter (APLT), class I, type 8A, member 1(Atp8a1)	Mus musculus
Cap1	CAP, adenylate cyclase-associated protein 1 (yeast)(Cap1)	Mus musculus
Cd47	CD47 antigen (Rh-related antigen, integrin-associated signal transducer)(Cd47)	Mus musculus
Cd81	CD81 antigen(Cd81)	Mus musculus
Cd82	CD82 antigen(Cd82)	Mus musculus
Cd9	CD9 antigen(Cd9)	Mus musculus
Cisd1	CDGSH iron sulfur domain 1(Cisd1)	Mus musculus
Cndp2	CNDP dipeptidase 2 (metallopeptidase M20 family)(Cndp2)	Mus musculus
Ddt	D-dopachrome tautomerase(Ddt)	Mus musculus
Dnaja1	DnaJ heat shock protein family (Hsp40) member A1(Dnaja1)	Mus musculus
Dnajc3	DnaJ heat shock protein family (Hsp40) member C3(Dnajc3)	Mus musculus
Dnajc5	DnaJ heat shock protein family (Hsp40) member C5(Dnajc5)	Mus musculus
Edil3	EGF-like repeats and discoidin I-like domains 3(Edil3)	Mus musculus
Erlin2	ER lipid raft associated 2(Erlin2)	Mus musculus
Fbxo2	F-box protein 2(Fbxo2)	Mus musculus
Fkbp1a	FK506 binding protein 1a(Fkbp1a)	Mus musculus
Fkbp2	FK506 binding protein 2(Fkbp2)	Mus musculus
Gnas	GNAS (guanine nucleotide binding protein, alpha stimulating) complex locus(Gnas)	Mus musculus
Lancl1	LanC (bacterial lantibiotic synthetase component C)-like 1(Lancl1)	Mus musculus
Asah1	N-acylsphingosine amidohydrolase 1(Asah1)	Mus musculus
Napa	N-ethylmaleimide sensitive fusion protein attachment protein alpha(Napa)	Mus musculus
Napb	N-ethylmaleimide sensitive fusion protein attachment protein beta(Napb)	Mus musculus
Napg	N-ethylmaleimide sensitive fusion protein attachment protein gamma(Napg)	Mus musculus
Nsf	N-ethylmaleimide sensitive fusion protein(Nsf)	Mus musculus
Ndrg1	N-myc downstream regulated gene 1(Ndrg1)	Mus musculus
Ndrg2	N-myc downstream regulated gene 2(Ndrg2)	Mus musculus
Ndrg3	N-myc downstream regulated gene 3(Ndrg3)	Mus musculus
Ndufa13	NADH dehydrogenase (ubiquinone) 1 alpha subcomplex, 13(Ndufa13)	Mus musculus
Ndufa4	NADH dehydrogenase (ubiquinone) 1 alpha subcomplex, 4(Ndufa4)	Mus musculus
Ndufb3	NADH dehydrogenase (ubiquinone) 1 beta subcomplex 3(Ndufb3)	Mus musculus
Ndufb4	NADH dehydrogenase (ubiquinone) 1 beta subcomplex 4(Ndufb4)	Mus musculus
Ndufb10	NADH dehydrogenase (ubiquinone) 1 beta subcomplex, 10(Ndufb10)	Mus musculus
Ndufb9	NADH dehydrogenase (ubiquinone) 1 beta subcomplex, 9(Ndufb9)	Mus musculus

© 2023. Melachroinou K et al. Published online at http://www.aginganddisease.org/EN/10.14336/AD.2023.0614

SUPPLEMENTARY DATA

Nckap1	NCK-associated protein 1(Nckap1)	Mus musculus
Nme1	NME/NM23 nucleoside diphosphate kinase 1(Nme1)	Mus musculus
Nme2	NME/NM23 nucleoside diphosphate kinase 2(Nme2)	Mus musculus
Npc1	Niemann-Pick type C1(Npc1)	Mus musculus
Otub1	OTU domain, ubiquitin aldehyde binding 1(Otub1)	Mus musculus
Ola1	Obg-like ATPase 1(Ola1)	Mus musculus
Park7	Parkinson disease (autosomal recessive, early onset) 7(Park7)	Mus musculus
Rab10	RAB10, member RAS oncogene family(Rab10)	Mus musculus
Rab11a	RAB11A, member RAS oncogene family(Rab11a)	Mus musculus
Rab11b	RAB11B, member RAS oncogene family(Rab11b)	Mus musculus
Rab14	RAB14, member RAS oncogene family(Rab14)	Mus musculus
Rab18	RAB18, member RAS oncogene family(Rab18)	Mus musculus
Rab1A	RAB1A, member RAS oncogene family(Rab1a)	Mus musculus
Rab1b	RAB1B, member RAS oncogene family(Rab1b)	Mus musculus
Rab21	RAB21, member RAS oncogene family(Rab21)	Mus musculus
Rab2a	RAB2A, member RAS oncogene family(Rab2a)	Mus musculus
Rab35	RAB35, member RAS oncogene family(Rab35)	Mus musculus
Rab5a	RAB5A, member RAS oncogene family(Rab5a)	Mus musculus
Rab5b	RAB5B, member RAS oncogene family(Rab5b)	Mus musculus
Rab5c	RAB5C, member RAS oncogene family(Rab5c)	Mus musculus
Rab6a	RAB6A, member RAS oncogene family(Rab6a)	Mus musculus
Ran	RAN, member RAS oncogene family(Ran)	Mus musculus
Rap1gds1	RAP1, GTP-GDP dissociation stimulator 1(Rap1gds1)	Mus musculus
Rap2b	RAP2B, member of RAS oncogene family(Rap2b)	Mus musculus
Rap2c	RAP2C, member of RAS oncogene family(Rap2c)	Mus musculus
Rap1b	RAS related protein 1b(Rap1b)	Mus musculus
Rap2a	RAS related protein 2a(Rap2a)	Mus musculus
Rac1	RAS-related C3 botulinum substrate 1(Rac1)	Mus musculus
Rac2	RAS-related C3 botulinum substrate 2(Rac2)	Mus musculus
Rheb	Ras homolog enriched in brain(Rheb)	Mus musculus
Arhgdia	Rho GDP dissociation inhibitor (GDI) alpha(Arhgdia)	Mus musculus
Arhgap1	Rho GTPase activating protein 1(Arhgap1)	Mus musculus
Ahcy	S-adenosylhomocysteine hydrolase(Ahcy)	Mus musculus
Ahcyl1	S-adenosylhomocysteine hydrolase-like 1(Ahcyl1)	Mus musculus
S100a16	S100 calcium binding protein A16(S100a16)	Mus musculus
Samm50	SAMM50 sorting and assembly machinery component(Samm50)	Mus musculus
Sec11a	SEC11 homolog A, signal peptidase complex subunit(Sec11a)	Mus musculus
Sh3gl2	SH3-domain GRB2-like 2(Sh3gl2)	Mus musculus
Sparcl1	SPARC-like 1(Sparcl1)	Mus musculus
Sub1	SUB1 homolog (S. cerevisiae)(Sub1)	Mus musculus

© 2023. Melachroinou K et al. Published online at http://www.aginganddisease.org/EN/10.14336/AD.2023.0614

Tufm	Tu translation elongation factor, mitochondrial(Tufm)	Mus musculus
Galnt16	UDP-N-acetyl-alpha-D-galactosamine:polypeptide N-acetylgalactosaminyltransferase 16(Galnt16)	Mus musculus
Galnt2	UDP-N-acetyl-alpha-D-galactosamine:polypeptide N-acetylgalactosaminyltransferase 2(Galnt2)	Mus musculus
Uggt1	UDP-glucose glycoprotein glucosyltransferase 1(Uggt1)	Mus musculus
Ugp2	UDP-glucose pyrophosphorylase 2(Ugp2)	Mus musculus
Vps29	VPS29 retromer complex component(Vps29)	Mus musculus
Vps35	VPS35 retromer complex component(Vps35)	Mus musculus
Wdr1	WD repeat domain 1(Wdr1)	Mus musculus
Yes1	YES proto-oncogene 1, Src family tyrosine kinase(Yes1)	Mus musculus
Adam10	a disintegrin and metallopeptidase domain 10(Adam10)	Mus musculus
Acat1	acetyl-Coenzyme A acetyltransferase 1(Acat1)	Mus musculus
Acat2	acetyl-Coenzyme A acetyltransferase 2(Acat2)	Mus musculus
Acp2	acid phosphatase 2, lysosomal(Acp2)	Mus musculus
Arpc1a	actin related protein 2/3 complex, subunit 1A(Arpc1a)	Mus musculus
Arpc2	actin related protein 2/3 complex, subunit 2(Arpc2)	Mus musculus
Arpc3	actin related protein 2/3 complex, subunit 3(Arpc3)	Mus musculus
Arpc4	actin related protein 2/3 complex, subunit 4(Arpc4)	Mus musculus
Arpc5l	actin related protein 2/3 complex, subunit 5-like(Arpc5l)	Mus musculus
Acta1	actin, alpha 1, skeletal muscle(Acta1)	Mus musculus
Acta2	actin, alpha 2, smooth muscle, aorta(Acta2)	Mus musculus
Actc1	actin, alpha, cardiac muscle 1(Actc1)	Mus musculus
Actg2	actin, gamma 2, smooth muscle, enteric(Actg2)	Mus musculus
Actg1	actin, gamma, cytoplasmic 1(Actg1)	Mus musculus
Actn1	actinin, alpha 1(Actn1)	Mus musculus
Alcam	activated leukocyte cell adhesion molecule(Alcam)	Mus musculus
Acot13	acyl-CoA thioesterase 13(Acot13)	Mus musculus
Acot7	acyl-CoA thioesterase 7(Acot7)	Mus musculus
Acyp1	acylphosphatase 1, erythrocyte (common) type(Acyp1)	Mus musculus
Ap2m1	adaptor-related protein complex 2, mu 1 subunit(Ap2m1)	Mus musculus
Ak1	adenylate kinase 1(Ak1)	Mus musculus
Adss	adenylosuccinate synthetase, non muscle(Adss)	Mus musculus
Apmap	adipocyte plasma membrane associated protein(Apmap)	Mus musculus
Aars	alanyl-tRNA synthetase(Aars)	Mus musculus
Alb	albumin(Alb)	Mus musculus
Adh5	alcohol dehydrogenase 5 (class III), chi polypeptide(Adh5)	Mus musculus
Aldh1l1	aldehyde dehydrogenase 1 family, member L1(Aldh1l1)	Mus musculus
Aldh2	aldehyde dehydrogenase 2, mitochondrial(Aldh2)	Mus musculus
Aldh3b1	aldehyde dehydrogenase 3 family, member B1(Aldh3b1)	Mus musculus
Aldh1a1	aldehyde dehydrogenase family 1, subfamily A1(Aldh1a1)	Mus musculus

© 2023. Melachroinou K et al. Published online at http://www.aginganddisease.org/EN/10.14336/AD.2023.0614

SUPPLEMENTARY DATA

Aldh3a2	aldehyde dehydrogenase family 3, subfamily A2(Aldh3a2)	Mus musculus
Aldh7a1	aldehyde dehydrogenase family 7, member A1(Aldh7a1)	Mus musculus
Akr1a1	aldo-keto reductase family 1, member A1 (aldehyde reductase)(Akr1a1)	Mus musculus
Aldoa	aldolase A, fructose-bisphosphate(Aldoa)	Mus musculus
Aldoc	aldolase C, fructose-bisphosphate(Aldoc)	Mus musculus
Alpl	alkaline phosphatase, liver/bone/kidney(Alpl)	Mus musculus
Ganab	alpha glucosidase 2 alpha neutral subunit(Ganab)	Mus musculus
Npepps	aminopeptidase puromycin sensitive(Npepps)	Mus musculus
App	amyloid beta (A4) precursor protein(App)	Mus musculus
Aplp2	amyloid beta (A4) precursor-like protein 2(Aplp2)	Mus musculus
Anxa11	annexin A11(Anxa11)	Mus musculus
Anxa2	annexin A2(Anxa2)	Mus musculus
Anxa3	annexin A3(Anxa3)	Mus musculus
Anxa4	annexin A4(Anxa4)	Mus musculus
Anxa5	annexin A5(Anxa5)	Mus musculus
Anxa6	annexin A6(Anxa6)	Mus musculus
Anxa7	annexin A7(Anxa7)	Mus musculus
Apod	apolipoprotein D(Apod)	Mus musculus
Apoe	apolipoprotein E(Apoe)	Mus musculus
Asna1	arsA arsenite transporter, ATP-binding, homolog 1 (bacterial)(Asna1)	Mus musculus
Nars	asparaginyl-tRNA synthetase(Nars)	Mus musculus
Bsg	basigin(Bsg)	Mus musculus
Bpnt1	bisphosphate 3'-nucleotidase 1(Bpnt1)	Mus musculus
Blmh	bleomycin hydrolase(Blmh)	Mus musculus
Basp1	brain abundant, membrane attached signal protein 1(Basp1)	Mus musculus
Pygb	brain glycogen phosphorylase(Pygb)	Mus musculus
Cdh13	cadherin 13(Cdh13)	Mus musculus
Cdh2	cadherin 2(Cdh2)	Mus musculus
Calb1	calbindin 1(Calb1)	Mus musculus
Cacna2d1	calcium channel, voltage-dependent, alpha2/delta subunit 1(Cacna2d1)	Mus musculus
Canx	calnexin(Canx)	Mus musculus
Capn2	calpain 2(Capn2)	Mus musculus
Capn5	calpain 5(Capn5)	Mus musculus
Calr	calreticulin(Calr)	Mus musculus
Clstn1	calsyntenin 1(Clstn1)	Mus musculus
Capza2	capping protein (actin filament) muscle Z-line, alpha 2(Capza2)	Mus musculus
Capzb	capping protein (actin filament) muscle Z-line, beta(Capzb)	Mus musculus
Cbr1	carbonyl reductase 1(Cbr1)	Mus musculus
Cpd	carboxypeptidase D(Cpd)	Mus musculus
Cpe	carboxypeptidase E(Cpe)	Mus musculus

© 2023. Melachroinou K et al. Published online at http://www.aginganddisease.org/EN/10.14336/AD.2023.0614

Crtac1	cartilage acidic protein 1(Crtac1)	Mus musculus
Cat	catalase(Cat)	Mus musculus
Ctnnb1	catenin (cadherin associated protein), beta 1(Ctnnb1)	Mus musculus
Ctsb	cathepsin B(Ctsb)	Mus musculus
Ctsd	cathepsin D(Ctsd)	Mus musculus
Cadm1	cell adhesion molecule 1(Cadm1)	Mus musculus
Cadm4	cell adhesion molecule 4(Cadm4)	Mus musculus
Chl1	cell adhesion molecule L1-like(Chl1)	Mus musculus
Cdc37	cell division cycle 37(Cdc37)	Mus musculus
Cdc42	cell division cycle 42(Cdc42)	Mus musculus
Cct2	chaperonin containing Tcp1, subunit 2 (beta)(Cct2)	Mus musculus
Cct3	chaperonin containing Tcp1, subunit 3 (gamma)(Cct3)	Mus musculus
Cct4	chaperonin containing Tcp1, subunit 4 (delta)(Cct4)	Mus musculus
Cct5	chaperonin containing Tcp1, subunit 5 (epsilon)(Cct5)	Mus musculus
Cct6a	chaperonin containing Tcp1, subunit 6a (zeta)(Cct6a)	Mus musculus
Cct7	chaperonin containing Tcp1, subunit 7 (eta)(Cct7)	Mus musculus
Cct8	chaperonin containing Tcp1, subunit 8 (theta)(Cct8)	Mus musculus
Chmp4b	charged multivesicular body protein 4B(Chmp4b)	Mus musculus
Chmp6	charged multivesicular body protein 6(Chmp6)	Mus musculus
Chid1	chitinase domain containing 1(Chid1)	Mus musculus
Cs	citrate synthase(Cs)	Mus musculus
Cltc	clathrin, heavy polypeptide (Hc)(Cltc)	Mus musculus
Clu	clusterin(Clu)	Mus musculus
Cotl1	coactosin-like 1 (Dictyostelium)(Cotl1)	Mus musculus
F3	coagulation factor III(F3)	Mus musculus
Cfl1	cofilin 1, non-muscle(Cfl1)	Mus musculus
Cfl2	cofilin 2, muscle(Cfl2)	Mus musculus
C1qc	complement component 1, q subcomponent, C chain(C1qc)	Mus musculus
C1qb	complement component 1, q subcomponent, beta polypeptide(C1qb)	Mus musculus
Cntn1	contactin 1(Cntn1)	Mus musculus
Cpne2	copine II(Cpne2)	Mus musculus
Cpne4	copine IV(Cpne4)	Mus musculus
Cpne5	copine V(Cpne5)	Mus musculus
Cpne6	copine VI(Cpne6)	Mus musculus
Cpne9	copine family member IX(Cpne9)	Mus musculus
Coro1a	coronin, actin binding protein 1A(Coro1a)	Mus musculus
Coro1b	coronin, actin binding protein 1B(Coro1b)	Mus musculus
Ckb	creatine kinase, brain(Ckb)	Mus musculus
Ckmt1	creatine kinase, mitochondrial 1, ubiquitous(Ckmt1)	Mus musculus
Cryab	crystallin, alpha B(Cryab)	Mus musculus

© 2023. Melachroinou K et al. Published online at http://www.aginganddisease.org/EN/10.14336/AD.2023.0614

Crym	crystallin, mu(Crym)	Mus musculus
Cul3	cullin 3(Cul3)	Mus musculus
Cand1	cullin associated and neddylation disassociated 1(Cand1)	Mus musculus
Ccny	cyclin Y(Ccny)	Mus musculus
Cst3	cystatin C(Cst3)	Mus musculus
Csrp1	cysteine and glycine-rich protein 1(Csrp1)	Mus musculus
Cmpk1	cytidine monophosphate (UMP-CMP) kinase 1(Cmpk1)	Mus musculus
Cox4i1	cytochrome c oxidase subunit IV isoform 1(Cox4i1)	Mus musculus
Cox7a2	cytochrome c oxidase subunit VIIa 2(Cox7a2)	Mus musculus
Cox5a	cytochrome c oxidase subunit Va(Cox5a)	Mus musculus
Cyfip2	cytoplasmic FMR1 interacting protein 2(Cyfip2)	Mus musculus
Ckap4	cytoskeleton-associated protein 4(Ckap4)	Mus musculus
Ddb1	damage specific DNA binding protein 1(Ddb1)	Mus musculus
Dad1	defender against cell death 1(Dad1)	Mus musculus
Dstn	destrin(Dstn)	Mus musculus
Dbi	diazepam binding inhibitor(Dbi)	Mus musculus
Dlst	dihydrolipoamide S-succinyltransferase (E2 component of 2-oxo-glutarate complex)(Dlst)	Mus musculus
Dpysl2	dihydropyrimidinase-like 2(Dpysl2)	Mus musculus
Ddah1	dimethylarginine dimethylaminohydrolase 1(Ddah1)	Mus musculus
Dpp6	dipeptidylpeptidase 6(Dpp6)	Mus musculus
Dlg1	discs, large homolog 1 (Drosophila)(Dlg1)	Mus musculus
Dusp3	dual specificity phosphatase 3 (vaccinia virus phosphatase VH1-related)(Dusp3)	Mus musculus
Dctn2	dynactin 2(Dctn2)	Mus musculus
Dnm1	dynamin 1(Dnm1)	Mus musculus
Dnm3	dynamin 3(Dnm3)	Mus musculus
Dync1h1	dynein cytoplasmic 1 heavy chain 1(Dync1h1)	Mus musculus
Entpd2	ectonucleoside triphosphate diphosphohydrolase 2(Entpd2)	Mus musculus
Enpp6	ectonucleotide pyrophosphatase/phosphodiesterase 6(Enpp6)	Mus musculus
Etfa	electron transferring flavoprotein, alpha polypeptide(Etfa)	Mus musculus
Endod1	endonuclease domain containing 1(Endod1)	Mus musculus
Erp29	endoplasmic reticulum protein 29(Erp29)	Mus musculus
Erp44	endoplasmic reticulum protein 44(Erp44)	Mus musculus
Eno1	enolase 1, alpha non-neuron(Eno1)	Mus musculus
Eno2	enolase 2, gamma neuronal(Eno2)	Mus musculus
Enoph1	enolase-phosphatase 1(Enoph1)	Mus musculus
Echs1	enoyl Coenzyme A hydratase, short chain, 1, mitochondrial(Echs1)	Mus musculus
Epdr1	ependymin related protein 1 (zebrafish)(Epdr1)	Mus musculus
Epb41l2	erythrocyte membrane protein band 4.1 like 2(Epb41l2)	Mus musculus
Esd	esterase D/formylglutathione hydrolase(Esd)	Mus musculus

© 2023. Melachroinou K et al. Published online at http://www.aginganddisease.org/EN/10.14336/AD.2023.0614

Eef1a1	eukaryotic translation elongation factor 1 alpha 1(Eef1a1)	Mus musculus
Eef1g	eukaryotic translation elongation factor 1 gamma(Eef1g)	Mus musculus
Eef2	eukaryotic translation elongation factor 2(Eef2)	Mus musculus
Eif4a1	eukaryotic translation initiation factor 4A1(Eif4a1)	Mus musculus
Eif5a	eukaryotic translation initiation factor 5A(Eif5a)	Mus musculus
Extl2	exostoses (multiple)-like 2(Extl2)	Mus musculus
Fam213a	family with sequence similarity 213, member A(Fam213a)	Mus musculus
Fam3c	family with sequence similarity 3, member C(Fam3c)	Mus musculus
Fam49b	family with sequence similarity 49, member B(Fam49b)	Mus musculus
Fscn1	fascin actin-bundling protein 1(Fscn1)	Mus musculus
Fabp3	fatty acid binding protein 3, muscle and heart(Fabp3)	Mus musculus
Fabp5	fatty acid binding protein 5, epidermal(Fabp5)	Mus musculus
Fasn	fatty acid synthase(Fasn)	Mus musculus
Fth1	ferritin heavy polypeptide 1(Fth1)	Mus musculus
Ftl1	ferritin light polypeptide 1(Ftl1)	Mus musculus
Flot1	flotillin 1(Flot1)	Mus musculus
Flot2	flotillin 2(Flot2)	Mus musculus
Folh1	folate hydrolase 1(Folh1)	Mus musculus
Gabrb2	gamma-aminobutyric acid (GABA) A receptor, subunit beta 2(Gabrb2)	Mus musculus
Gja1	gap junction protein, alpha 1(Gja1)	Mus musculus
Gnpda1	glucosamine-6-phosphate deaminase 1(Gnpda1)	Mus musculus
Grm5	glutamate receptor, metabotropic 5(Grm5)	Mus musculus
Glul	glutamate-ammonia ligase (glutamine synthetase)(Glul)	Mus musculus
Got2	glutamatic-oxaloacetic transaminase 2, mitochondrial(Got2)	Mus musculus
Got1	glutamic-oxaloacetic transaminase 1, soluble(Got1)	Mus musculus
Gstm5	glutathione S-transferase, mu 5(Gstm5)	Mus musculus
Gstp1	glutathione S-transferase, pi 1(Gstp1)	Mus musculus
Gstp2	glutathione S-transferase, pi 2(Gstp2)	Mus musculus
Gpx4	glutathione peroxidase 4(Gpx4)	Mus musculus
Gapdh	glyceraldehyde-3-phosphate dehydrogenase(Gapdh)	Mus musculus
Gpd1	glycerol-3-phosphate dehydrogenase 1 (soluble)(Gpd1)	Mus musculus
Gpd1l	glycerol-3-phosphate dehydrogenase 1-like(Gpd1l)	Mus musculus
Gltp	glycolipid transfer protein(Gltp)	Mus musculus
Gpm6a	glycoprotein m6a(Gpm6a)	Mus musculus
Glo1	glyoxalase 1(Glo1)	Mus musculus
Glod4	glyoxalase domain containing 4(Glod4)	Mus musculus
Grhpr	glyoxylate reductase/hydroxypyruvate reductase(Grhpr)	Mus musculus
Gpc1	glypican 1(Gpc1)	Mus musculus
Glg1	golgi apparatus protein 1(Glg1)	Mus musculus
Golga7	golgi autoantigen, golgin subfamily a, 7(Golga7)	Mus musculus

© 2023. Melachroinou K et al. Published online at http://www.aginganddisease.org/EN/10.14336/AD.2023.0614

SUPPLEMENTARY DATA

Grb2	growth factor receptor bound protein 2(Grb2)	Mus musculus
Gda	guanine deaminase(Gda)	Mus musculus
Gnai1	guanine nucleotide binding protein (G protein), alpha inhibiting 1(Gnai1)	Mus musculus
Gnai2	guanine nucleotide binding protein (G protein), alpha inhibiting 2(Gnai2)	Mus musculus
Gnai3	guanine nucleotide binding protein (G protein), alpha inhibiting 3(Gnai3)	Mus musculus
Gnb1	guanine nucleotide binding protein (G protein), beta 1(Gnb1)	Mus musculus
Gnb2	guanine nucleotide binding protein (G protein), beta 2(Gnb2)	Mus musculus
Gnb4	guanine nucleotide binding protein (G protein), beta 4(Gnb4)	Mus musculus
Gng12	guanine nucleotide binding protein (G protein), gamma 12(Gng12)	Mus musculus
Gng2	guanine nucleotide binding protein (G protein), gamma 2(Gng2)	Mus musculus
Gna11	guanine nucleotide binding protein, alpha 11(Gna11)	Mus musculus
Gna13	guanine nucleotide binding protein, alpha 13(Gna13)	Mus musculus
Gnaq	guanine nucleotide binding protein, alpha q polypeptide(Gnaq)	Mus musculus
Gnal	guanine nucleotide binding protein, alpha stimulating, olfactory type(Gnal)	Mus musculus
Gnaz	guanine nucleotide binding protein, alpha z subunit(Gnaz)	Mus musculus
Gdi2	guanosine diphosphate (GDP) dissociation inhibitor 2(Gdi2)	Mus musculus
Hdhd2	haloacid dehalogenase-like hydrolase domain containing 2(Hdhd2)	Mus musculus
Hsph1	heat shock 105kDa/110kDa protein 1(Hsph1)	Mus musculus
Hspe1	heat shock protein 1 (chaperonin 10)(Hspe1)	Mus musculus
Hspd1	heat shock protein 1 (chaperonin)(Hspd1)	Mus musculus
Hspa12a	heat shock protein 12A(Hspa12a)	Mus musculus
Hspa4	heat shock protein 4(Hspa4)	Mus musculus
Hspa5	heat shock protein 5(Hspa5)	Mus musculus
Hspa13	heat shock protein 70 family, member 13(Hspa13)	Mus musculus
Hspa8	heat shock protein 8(Hspa8)	Mus musculus
Hspa9	heat shock protein 9(Hspa9)	Mus musculus
Hsp90ab1	heat shock protein 90 alpha (cytosolic), class B member 1(Hsp90ab1)	Mus musculus
Hsp90aa1	heat shock protein 90, alpha (cytosolic), class A member 1(Hsp90aa1)	Mus musculus
Hsp90b1	heat shock protein 90, beta (Grp94), member 1(Hsp90b1)	Mus musculus
Hnrnpa1	heterogeneous nuclear ribonucleoprotein A1(Hnrnpa1)	Mus musculus
Hnrnpa2b1	heterogeneous nuclear ribonucleoprotein A2/B1(Hnrnpa2b1)	Mus musculus
Hnrnpd	heterogeneous nuclear ribonucleoprotein D(Hnrnpd)	Mus musculus
Hnrnpdl	heterogeneous nuclear ribonucleoprotein D-like(Hnrnpdl)	Mus musculus
Hnrnpk	heterogeneous nuclear ribonucleoprotein K(Hnrnpk)	Mus musculus
Hexb	hexosaminidase B(Hexb)	Mus musculus
Hpcal1	hippocalcin-like 1(Hpcal1)	Mus musculus
Hint1	histidine triad nucleotide binding protein 1(Hint1)	Mus musculus
Hint3	histidine triad nucleotide binding protein 3(Hint3)	Mus musculus
Hist1h4a	histone cluster 1, H4a(Hist1h4a)	Mus musculus
Hdac11	histone deacetylase 11(Hdac11)	Mus musculus

© 2023. Melachroinou K et al. Published online at http://www.aginganddisease.org/EN/10.14336/AD.2023.0614

SUPPLEMENTARY DATA

Hagh	hydroxyacyl glutathione hydrolase(Hagh)	Mus musculus
Hyou1	hypoxia up-regulated 1(Hyou1)	Mus musculus
Igsf8	immunoglobulin superfamily, member 8(Igsf8)	Mus musculus
Impa1	inositol (myo)-1(or 4)-monophosphatase 1(Impa1)	Mus musculus
Inpp5a	inositol polyphosphate-5-phosphatase A(Inpp5a)	Mus musculus
Itm2c	integral membrane protein 2C(Itm2c)	Mus musculus
Itfg1	integrin alpha FG-GAP repeat containing 1(Itfg1)	Mus musculus
Itgam	integrin alpha M(Itgam)	Mus musculus
Itgav	integrin alpha V(Itgav)	Mus musculus
Itgb1	integrin beta 1 (fibronectin receptor beta)(Itgb1)	Mus musculus
Itgb8	integrin beta 8(Itgb8)	Mus musculus
Idh1	isocitrate dehydrogenase 1 (NADP+), soluble(Idh1)	Mus musculus
Idh2	isocitrate dehydrogenase 2 (NADP+), mitochondrial(Idh2)	Mus musculus
Jup	junction plakoglobin(Jup)	Mus musculus
Kpnb1	karyopherin (importin) beta 1(Kpnb1)	Mus musculus
Ldha	lactate dehydrogenase A(Ldha)	Mus musculus
Ldhb	lactate dehydrogenase B(Ldhb)	Mus musculus
Lxn	latexin(Lxn)	Mus musculus
Lman2	lectin, mannose-binding 2(Lman2)	Mus musculus
Lman1	lectin, mannose-binding, 1(Lman1)	Mus musculus
Lap3	leucine aminopeptidase 3(Lap3)	Mus musculus
Lrrc57	leucine rich repeat containing 57(Lrrc57)	Mus musculus
Lta4h	leukotriene A4 hydrolase(Lta4h)	Mus musculus
Lin7a	lin-7 homolog A (C. elegans)(Lin7a)	Mus musculus
Lin7c	lin-7 homolog C (C. elegans)(Lin7c)	Mus musculus
Lamp1	lysosomal-associated membrane protein 1(Lamp1)	Mus musculus
Lamp2	lysosomal-associated membrane protein 2(Lamp2)	Mus musculus
Mif	macrophage migration inhibitory factor(Mif)	Mus musculus
Mdh1	malate dehydrogenase 1, NAD (soluble)(Mdh1)	Mus musculus
Mdh2	malate dehydrogenase 2, NAD (mitochondrial)(Mdh2)	Mus musculus
Mpdu1	mannose-P-dolichol utilization defect 1(Mpdu1)	Mus musculus
Mogs	mannosyl-oligosaccharide glucosidase(Mogs)	Mus musculus
Mpp6	membrane protein, palmitoylated 6 (MAGUK p55 subfamily member 6)(Mpp6)	Mus musculus
Mblac2	metallo-beta-lactamase domain containing 2(Mblac2)	Mus musculus
Mgst3	microsomal glutathione S-transferase 3(Mgst3)	Mus musculus
Mfge8	milk fat globule-EGF factor 8 protein(Mfge8)	Mus musculus
Mtch2	mitochondrial carrier 2(Mtch2)	Mus musculus
Mapk1	mitogen-activated protein kinase 1(Mapk1)	Mus musculus
Map2k1	mitogen-activated protein kinase kinase 1(Map2k1)	Mus musculus
Msn	moesin(Msn)	Mus musculus

© 2023. Melachroinou K et al. Published online at http://www.aginganddisease.org/EN/10.14336/AD.2023.0614

SUPPLEMENTARY DATA

Maob	monoamine oxidase B(Maob)	Mus musculus
Mras	muscle and microspikes RAS(Mras)	Mus musculus
Pygm	muscle glycogen phosphorylase(Pygm)	Mus musculus
Mydgf	myeloid derived growth factor(Mydgf)	Mus musculus
Myadm	myeloid-associated differentiation marker(Myadm)	Mus musculus
Myo1d	myosin ID(Myo1d)	Mus musculus
Myh10	myosin, heavy polypeptide 10, non-muscle(Myh10)	Mus musculus
Myl12b	myosin, light chain 12B, regulatory(Myl12b)	Mus musculus
Mtpn	myotrophin(Mtpn)	Mus musculus
Marcks	myristoylated alanine rich protein kinase C substrate(Marcks)	Mus musculus
Ncam1	neural cell adhesion molecule 1(Ncam1)	Mus musculus
Ncald	neurocalcin delta(Ncald)	Mus musculus
Nfasc	neurofascin(Nfasc)	Mus musculus
Ncs1	neuronal calcium sensor 1(Ncs1)	Mus musculus
Negr1	neuronal growth regulator 1(Negr1)	Mus musculus
Ncstn	nicastrin(Ncstn)	Mus musculus
Nutf2	nuclear transport factor 2(Nutf2)	Mus musculus
Nucb1	nucleobindin 1(Nucb1)	Mus musculus
Nucb2	nucleobindin 2(Nucb2)	Mus musculus
Ppt1	palmitoyl-protein thioesterase 1(Ppt1)	Mus musculus
Pvalb	parvalbumin(Pvalb)	Mus musculus
Pef1	penta-EF hand domain containing 1(Pef1)	Mus musculus
Pepd	peptidase D(Pepd)	Mus musculus
Pam	peptidylglycine alpha-amidating monooxygenase(Pam)	Mus musculus
Ppia	peptidylprolyl isomerase A(Ppia)	Mus musculus
Ppib	peptidylprolyl isomerase B(Ppib)	Mus musculus
Prdx1	peroxiredoxin 1(Prdx1)	Mus musculus
Prdx2	peroxiredoxin 2(Prdx2)	Mus musculus
Prdx3	peroxiredoxin 3(Prdx3)	Mus musculus
Prdx4	peroxiredoxin 4(Prdx4)	Mus musculus
Prdx5	peroxiredoxin 5(Prdx5)	Mus musculus
Prdx6	peroxiredoxin 6(Prdx6)	Mus musculus
Pebp1	phosphatidylethanolamine binding protein 1(Pebp1)	Mus musculus
Pi4ka	phosphatidylinositol 4-kinase, catalytic, alpha polypeptide(Pi4ka)	Mus musculus
Pitpna	phosphatidylinositol transfer protein, alpha(Pitpna)	Mus musculus
Pitpnb	phosphatidylinositol transfer protein, beta(Pitpnb)	Mus musculus
Pfkl	phosphofructokinase, liver, B-type(Pfkl)	Mus musculus
Pfkm	phosphofructokinase, muscle(Pfkm)	Mus musculus
Pfkp	phosphofructokinase, platelet(Pfkp)	Mus musculus
Pgm1	phosphoglucomutase 1(Pgm1)	Mus musculus

© 2023. Melachroinou K et al. Published online at http://www.aginganddisease.org/EN/10.14336/AD.2023.0614

Pgd	phosphogluconate dehydrogenase(Pgd)	Mus musculus
Pgk1	phosphoglycerate kinase 1(Pgk1)	Mus musculus
Pgam1	phosphoglycerate mutase 1(Pgam1)	Mus musculus
Phpt1	phosphohistidine phosphatase 1(Phpt1)	Mus musculus
Plcb1	phospholipase C, beta 1(Plcb1)	Mus musculus
Pld3	phospholipase D family, member 3(Pld3)	Mus musculus
Prps2	phosphoribosyl pyrophosphate synthetase 2(Prps2)	Mus musculus
Paics	phosphoribosylaminoimidazole carboxylase, phosphoribosylaminoribosylaminoimidazole, succinocarboxamide synthetase(Paics)	Mus musculus
Psat1	phosphoserine aminotransferase 1(Psat1)	Mus musculus
Pafah1b1	platelet-activating factor acetylhydrolase, isoform 1b, subunit 1(Pafah1b1)	Mus musculus
Pafah1b2	platelet-activating factor acetylhydrolase, isoform 1b, subunit 2(Pafah1b2)	Mus musculus
Plxna1	plexin A1(Plxna1)	Mus musculus
Plxnb2	plexin B2(Plxnb2)	Mus musculus
Pcbp1	poly(rC) binding protein 1(Pcbp1)	Mus musculus
Kcnma1	potassium large conductance calcium-activated channel, subfamily M, alpha member 1(Kcnma1)	Mus musculus
Pcyox1	prenylcysteine oxidase 1(Pcyox1)	Mus musculus
Prnp	prion protein(Prnp)	Mus musculus
Pfn2	profilin 2(Pfn2)	Mus musculus
Pdcd6ip	programmed cell death 6 interacting protein(Pdcd6ip)	Mus musculus
Pdcd6	programmed cell death 6(Pdcd6)	Mus musculus
Phb2	prohibitin 2(Phb2)	Mus musculus
Phb	prohibitin(Phb)	Mus musculus
P4hb	prolyl 4-hydroxylase, beta polypeptide(P4hb)	Mus musculus
Prom1	prominin 1(Prom1)	Mus musculus
Pcsk1n	proprotein convertase subtilisin/kexin type 1 inhibitor(Pcsk1n)	Mus musculus
Psap	prosaposin(Psap)	Mus musculus
Ptges3	prostaglandin E synthase 3 (cytosolic)(Ptges3)	Mus musculus
Psmd12	proteasome (prosome, macropain) 26S subunit, non-ATPase, 12(Psmd12)	Mus musculus
Psma1	proteasome (prosome, macropain) subunit, alpha type 1(Psma1)	Mus musculus
Psma2	proteasome (prosome, macropain) subunit, alpha type 2(Psma2)	Mus musculus
Psma3	proteasome (prosome, macropain) subunit, alpha type 3(Psma3)	Mus musculus
Psma4	proteasome (prosome, macropain) subunit, alpha type 4(Psma4)	Mus musculus
Psma5	proteasome (prosome, macropain) subunit, alpha type 5(Psma5)	Mus musculus
Psma6	proteasome (prosome, macropain) subunit, alpha type 6(Psma6)	Mus musculus
Psma7	proteasome (prosome, macropain) subunit, alpha type 7(Psma7)	Mus musculus
Psmb1	proteasome (prosome, macropain) subunit, beta type 1(Psmb1)	Mus musculus
Psmb3	proteasome (prosome, macropain) subunit, beta type 3(Psmb3)	Mus musculus
Psmb4	proteasome (prosome, macropain) subunit, beta type 4(Psmb4)	Mus musculus
Psmb5	proteasome (prosome, macropain) subunit, beta type 5(Psmb5)	Mus musculus

© 2023. Melachroinou K et al. Published online at http://www.aginganddisease.org/EN/10.14336/AD.2023.0614

Psmb6	proteasome (prosome, macropain) subunit, beta type 6(Psmb6)	Mus musculus
Psmb7	proteasome (prosome, macropain) subunit, beta type 7(Psmb7)	Mus musculus
Pdia3	protein disulfide isomerase associated 3(Pdia3)	Mus musculus
Pdia6	protein disulfide isomerase associated 6(Pdia6)	Mus musculus
Pacsin2	protein kinase C and casein kinase substrate in neurons 2(Pacsin2)	Mus musculus
Prkca	protein kinase C, alpha(Prkca)	Mus musculus
Prkcb	protein kinase C, beta(Prkcb)	Mus musculus
Prkar2a	protein kinase, cAMP dependent regulatory, type II alpha(Prkar2a)	Mus musculus
Prkar2b	protein kinase, cAMP dependent regulatory, type II beta(Prkar2b)	Mus musculus
Prkaca	protein kinase, cAMP dependent, catalytic, alpha(Prkaca)	Mus musculus
Prkacb	protein kinase, cAMP dependent, catalytic, beta(Prkacb)	Mus musculus
Ppp1ca	protein phosphatase 1, catalytic subunit, alpha isoform(Ppp1ca)	Mus musculus
Ppp1cb	protein phosphatase 1, catalytic subunit, beta isoform(Ppp1cb)	Mus musculus
Ppp1r7	protein phosphatase 1, regulatory (inhibitor) subunit 7(Ppp1r7)	Mus musculus
Ppp2ca	protein phosphatase 2 (formerly 2A), catalytic subunit, alpha isoform(Ppp2ca)	Mus musculus
Ppp2r1a	protein phosphatase 2, regulatory subunit A, alpha(Ppp2r1a)	Mus musculus
Ptpra	protein tyrosine phosphatase, receptor type, A(Ptpra)	Mus musculus
Ptprd	protein tyrosine phosphatase, receptor type, D(Ptprd)	Mus musculus
Ptprj	protein tyrosine phosphatase, receptor type, J(Ptprj)	Mus musculus
Ptprs	protein tyrosine phosphatase, receptor type, S(Ptprs)	Mus musculus
Ptma	prothymosin alpha(Ptma)	Mus musculus
Pdxk	pyridoxal (pyridoxine, vitamin B6) kinase(Pdxk)	Mus musculus
Pdxp	pyridoxal (pyridoxine, vitamin B6) phosphatase(Pdxp)	Mus musculus
Ppa1	pyrophosphatase (inorganic) 1(Ppa1)	Mus musculus
Ppa2	pyrophosphatase (inorganic) 2(Ppa2)	Mus musculus
Pkm	pyruvate kinase, muscle(Pkm)	Mus musculus
Qdpr	quinoid dihydropteridine reductase(Qdpr)	Mus musculus
Rdx	radixin(Rdx)	Mus musculus
Rhoa	ras homolog family member A(Rhoa)	Mus musculus
Rhob	ras homolog family member B(Rhob)	Mus musculus
Rhoc	ras homolog family member C(Rhoc)	Mus musculus
Rhog	ras homolog family member G(Rhog)	Mus musculus
Reep5	receptor accessory protein 5(Reep5)	Mus musculus
Rras2	related RAS viral (r-ras) oncogene 2(Rras2)	Mus musculus
Rras	related RAS viral (r-ras) oncogene(Rras)	Mus musculus
Rtn3	reticulon 3(Rtn3)	Mus musculus
Rtn4	reticulon 4(Rtn4)	Mus musculus
Rpl10a	ribosomal protein L10A(Rpl10a)	Mus musculus
Rps27a	ribosomal protein S27A(Rps27a)	Mus musculus
Ryr2	ryanodine receptor 2, cardiac(Ryr2)	Mus musculus

© 2023. Melachroinou K et al. Published online at http://www.aginganddisease.org/EN/10.14336/AD.2023.0614

Scarb2	scavenger receptor class B, member 2(Scarb2)	Mus musculus
Spr	sepiapterin reductase(Spr)	Mus musculus
Serpina1a	serine (or cysteine) peptidase inhibitor, clade A, member 1A(Serpina1a)	Mus musculus
Serpina1c	serine (or cysteine) peptidase inhibitor, clade A, member 1C(Serpina1c)	Mus musculus
Serpina1e	serine (or cysteine) peptidase inhibitor, clade A, member 1E(Serpina1e)	Mus musculus
Serpina3k	serine (or cysteine) peptidase inhibitor, clade A, member 3K(Serpina3k)	Mus musculus
Serpinb1a	serine (or cysteine) peptidase inhibitor, clade B, member 1a(Serpinb1a)	Mus musculus
Serpini1	serine (or cysteine) peptidase inhibitor, clade I, member 1(Serpini1)	Mus musculus
Serinc1	serine incorporator 1(Serinc1)	Mus musculus
Sars	seryl-aminoacyl-tRNA synthetase(Sars)	Mus musculus
Ssr4	signal sequence receptor, delta(Ssr4)	Mus musculus
Sirpa	signal-regulatory protein alpha(Sirpa)	Mus musculus
Slc1a4	solute carrier family 1 (glutamate/neutral amino acid transporter), member 4(Slc1a4)	Mus musculus
Slc1a1	solute carrier family 1 (neuronal/epithelial high affinity glutamate transporter, system Xag), member 1(Slc1a1)	Mus musculus
Slc12a2	solute carrier family 12, member 2(Slc12a2)	Mus musculus
Slc15a2	solute carrier family 15 (H+/peptide transporter), member 2(Slc15a2)	Mus musculus
Slc16a1	solute carrier family 16 (monocarboxylic acid transporters), member 1(Slc16a1)	Mus musculus
Slc2a1	solute carrier family 2 (facilitated glucose transporter), member 1(Slc2a1)	Mus musculus
Slc2a3	solute carrier family 2 (facilitated glucose transporter), member 3(Slc2a3)	Mus musculus
Slc25a5	solute carrier family 25 (mitochondrial carrier, adenine nucleotide translocator), member 5(Slc25a5)	Mus musculus
Slc25a3	solute carrier family 25 (mitochondrial carrier, phosphate carrier), member 3(Slc25a3)	Mus musculus
Slc3a2	solute carrier family 3 (activators of dibasic and neutral amino acid transport), member 2(Slc3a2)	Mus musculus
Slc30a7	solute carrier family 30 (zinc transporter), member 7(Slc30a7)	Mus musculus
Slc4a4	solute carrier family 4 (anion exchanger), member 4(Slc4a4)	Mus musculus
Slc44a1	solute carrier family 44, member 1(Slc44a1)	Mus musculus
Slc44a2	solute carrier family 44, member 2(Slc44a2)	Mus musculus
Slc7a5	solute carrier family 7 (cationic amino acid transporter, y+ system), member 5(Slc7a5)	Mus musculus
Slc7a8	solute carrier family 7 (cationic amino acid transporter, y+ system), member 8(Slc7a8)	Mus musculus
Slc9a1	solute carrier family 9 (sodium/hydrogen exchanger), member 1(Slc9a1)	Mus musculus
Slc9a3r1	solute carrier family 9 (sodium/hydrogen exchanger), member 3 regulator 1(Slc9a3r1)	Mus musculus
Sri	sorcin(Sri)	Mus musculus
Sorl1	sortilin-related receptor, LDLR class A repeats-containing(Sorl1)	Mus musculus
Sptan1	spectrin alpha, non-erythrocytic 1(Sptan1)	Mus musculus
Sptbn1	spectrin beta, non-erythrocytic 1(Sptbn1)	Mus musculus
Smpdl3b	sphingomyelin phosphodiesterase, acid-like 3B(Smpdl3b)	Mus musculus
Stmn1	stathmin 1(Stmn1)	Mus musculus
Sdf4	stromal cell derived factor 4(Sdf4)	Mus musculus
Sdhb	succinate dehydrogenase complex, subunit B, iron sulfur (Ip)(Sdhb)	Mus musculus
Suclg1	succinate-CoA ligase, GDP-forming, alpha subunit(Suclg1)	Mus musculus

Sucla2	succinate-Coenzyme A ligase, ADP-forming, beta subunit(Sucla2)	Mus musculus
Sod1	superoxide dismutase 1, soluble(Sod1)	Mus musculus
Sod2	superoxide dismutase 2, mitochondrial(Sod2)	Mus musculus
St13	suppression of tumorigenicity 13(St13)	Mus musculus
Susd2	sushi domain containing 2(Susd2)	Mus musculus
Syt7	synaptotagmin VII(Syt7)	Mus musculus
Sdcbp	syndecan binding protein(Sdcbp)	Mus musculus
Stx12	syntaxin 12(Stx12)	Mus musculus
Stxbp1	syntaxin binding protein 1(Stxbp1)	Mus musculus
Tcp1	t-complex protein 1(Tcp1)	Mus musculus
Txndc5	thioredoxin domain containing 5(Txndc5)	Mus musculus
Txnrd1	thioredoxin reductase 1(Txnrd1)	Mus musculus
Thy1	thymus cell antigen 1, theta(Thy1)	Mus musculus
Tollip	toll interacting protein(Tollip)	Mus musculus
Taldo1	transaldolase 1(Taldo1)	Mus musculus
Tfrc	transferrin receptor(Tfrc)	Mus musculus
Tkt	transketolase(Tkt)	Mus musculus
Tomm40	translocase of outer mitochondrial membrane 40 homolog (yeast)(Tomm40)	Mus musculus
Tm9sf2	transmembrane 9 superfamily member 2(Tm9sf2)	Mus musculus
Tmed10	transmembrane p24 trafficking protein 10(Tmed10)	Mus musculus
Tmed4	transmembrane p24 trafficking protein 4(Tmed4)	Mus musculus
Tmed9	transmembrane p24 trafficking protein 9(Tmed9)	Mus musculus
Tmem109	transmembrane protein 109(Tmem109)	Mus musculus
Tmem33	transmembrane protein 33(Tmem33)	Mus musculus
Ttr	transthyretin(Ttr)	Mus musculus
Tpi1	triosephosphate isomerase 1(Tpi1)	Mus musculus
Tpm3	tropomyosin 3, gamma(Tpm3)	Mus musculus
Wars	tryptophanyl-tRNA synthetase(Wars)	Mus musculus
Tppp3	tubulin polymerization-promoting protein family member 3(Tppp3)	Mus musculus
Tuba1a	tubulin, alpha 1A(Tuba1a)	Mus musculus
Tuba1b	tubulin, alpha 1B(Tuba1b)	Mus musculus
Tuba4a	tubulin, alpha 4A(Tuba4a)	Mus musculus
Tubb2a	tubulin, beta 2A class IIA(Tubb2a)	Mus musculus
Tubb3	tubulin, beta 3 class III(Tubb3)	Mus musculus
Tubb4a	tubulin, beta 4A class IVA(Tubb4a)	Mus musculus
Tubb4b	tubulin, beta 4B class IVB(Tubb4b)	Mus musculus
Tubb5	tubulin, beta 5 class I(Tubb5)	Mus musculus
Tpt1	tumor protein, translationally-controlled 1(Tpt1)	Mus musculus
Ttyh3	tweety family member 3(Ttyh3)	Mus musculus

© 2023. Melachroinou K et al. Published online at http://www.aginganddisease.org/EN/10.14336/AD.2023.0614

Ywhab	tyrosine 3-monooxygenase/tryptophan 5-monooxygenase activation protein, beta polypeptide(Ywhab)	Mus musculus
Ywhae	tyrosine 3-monooxygenase/tryptophan 5-monooxygenase activation protein, epsilon polypeptide(Ywhae)	Mus musculus
Ywhah	tyrosine 3-monooxygenase/tryptophan 5-monooxygenase activation protein, eta polypeptide(Ywhah)	Mus musculus
Ywhag	tyrosine 3-monooxygenase/tryptophan 5-monooxygenase activation protein, gamma polypeptide(Ywhag)	Mus musculus
Ywhaq	tyrosine 3-monooxygenase/tryptophan 5-monooxygenase activation protein, theta polypeptide(Ywhaq)	Mus musculus
Ywhaz	tyrosine 3-monooxygenase/tryptophan 5-monooxygenase activation protein, zeta polypeptide(Ywhaz)	Mus musculus
Uqcrc2	ubiquinol cytochrome c reductase core protein 2(Uqcrc2)	Mus musculus
Uqcr10	ubiquinol-cytochrome c reductase, complex III subunit X(Uqcr10)	Mus musculus
Ubc	ubiquitin C(Ubc)	Mus musculus
Uchl1	ubiquitin carboxy-terminal hydrolase L1(Uchl1)	Mus musculus
Uchl3	ubiquitin carboxyl-terminal esterase L3 (ubiquitin thiolesterase)(Uchl3)	Mus musculus
Usp14	ubiquitin specific peptidase 14(Usp14)	Mus musculus
Ube2v1	ubiquitin-conjugating enzyme E2 variant 1(Ube2v1)	Mus musculus
Ube2v2	ubiquitin-conjugating enzyme E2 variant 2(Ube2v2)	Mus musculus
Ube2n	ubiquitin-conjugating enzyme E2N(Ube2n)	Mus musculus
Uba1	ubiquitin-like modifier activating enzyme 1(Uba1)	Mus musculus
Usmg5	upregulated during skeletal muscle growth 5(Usmg5)	Mus musculus
Rala	v-ral simian leukemia viral oncogene A (ras related)(Rala)	Mus musculus
Vcp	valosin containing protein(Vcp)	Mus musculus
Vcam1	vascular cell adhesion molecule 1(Vcam1)	Mus musculus
Vamp2	vesicle-associated membrane protein 2(Vamp2)	Mus musculus
Vdac1	voltage-dependent anion channel 1(Vdac1)	Mus musculus
Vdac2	voltage-dependent anion channel 2(Vdac2)	Mus musculus
Vdac3	voltage-dependent anion channel 3(Vdac3)	Mus musculus
Zmpste24	zinc metallopeptidase, STE24(Zmpste24)	Mus musculus

© 2023. Melachroinou K et al. Published online at http://www.aginganddisease.org/EN/10.14336/AD.2023.0614

SUPPLEMENTARY DATA

Supplementary Table 3. DAVID Platform: Brain tissue-related proteins present in our dataset.

Official gene symbol	Gene name	Species
Cnp	2',3'-cyclic nucleotide 3' phosphodiesterase(Cnp)	Mus musculus
Hacd2	3-hydroxyacyl-CoA dehydratase 2(Hacd2)	Mus musculus
Hacd3	3-hydroxyacyl-CoA dehydratase 3(Hacd3)	Mus musculus
Bdh1	3-hydroxybutyrate dehydrogenase, type 1(Bdh1)	Mus musculus
Oxct1	3-oxoacid CoA transferase 1(Oxct1)	Mus musculus
Phgdh	3-phosphoglycerate dehydrogenase(Phgdh)	Mus musculus
Abat	4-aminobutyrate aminotransferase(Abat)	Mus musculus
Nt5e	5' nucleotidase, ecto(Nt5e)	Mus musculus
Arf1	ADP-ribosylation factor 1(Arf1)	Mus musculus
Arf2	ADP-ribosylation factor 2(Arf2)	Mus musculus
Arf3	ADP-ribosylation factor 3(Arf3)	Mus musculus
Arf5	ADP-ribosylation factor 5(Arf5)	Mus musculus
Arf6	ADP-ribosylation factor 6(Arf6)	Mus musculus
Arl6ip1	ADP-ribosylation factor-like 6 interacting protein 1(Arl6ip1)	Mus musculus
Actr1b	ARP1 actin-related protein 1B, centractin beta(Actr1b)	Mus musculus
Actr2	ARP2 actin-related protein 2(Actr2)	Mus musculus
Actr3	ARP3 actin-related protein 3(Actr3)	Mus musculus
Acly	ATP citrate lyase(Acly)	Mus musculus
Atp5b	ATP synthase, H+ transporting mitochondrial F1 complex, beta subunit(Atp5b)	Mus musculus
Atp5f1	ATP synthase, H+ transporting, mitochondrial F0 complex, subunit B1(Atp5f1)	Mus musculus
Atp5h	ATP synthase, H+ transporting, mitochondrial F0 complex, subunit D(Atp5h)	Mus musculus
Atp5j2	ATP synthase, H+ transporting, mitochondrial F0 complex, subunit F2(Atp5j2)	Mus musculus
Atp5l	ATP synthase, H+ transporting, mitochondrial F0 complex, subunit G(Atp5l)	Mus musculus
Atp5o	ATP synthase, H+ transporting, mitochondrial F1 complex, O subunit(Atp5o)	Mus musculus
Atp5a1	ATP synthase, H+ transporting, mitochondrial F1 complex, alpha subunit 1(Atp5a1)	Mus musculus
Atp5c1	ATP synthase, H+ transporting, mitochondrial F1 complex, gamma polypeptide 1(Atp5c1)	Mus musculus
Atp2a2	ATPase, Ca++ transporting, cardiac muscle, slow twitch 2(Atp2a2)	Mus musculus
Atp2b2	ATPase, Ca++ transporting, plasma membrane 2(Atp2b2)	Mus musculus
Atp6v0a1	ATPase, H+ transporting, lysosomal V0 subunit A1(Atp6v0a1)	Mus musculus
Atp6v0c	ATPase, H+ transporting, lysosomal V0 subunit C(Atp6v0c)	Mus musculus
Atp6v0d1	ATPase, H+ transporting, lysosomal V0 subunit D1(Atp6v0d1)	Mus musculus
Atp6v1a	ATPase, H+ transporting, lysosomal V1 subunit A(Atp6v1a)	Mus musculus
Atp6v1b2	ATPase, H+ transporting, lysosomal V1 subunit B2(Atp6v1b2)	Mus musculus
Atp6v1c1	ATPase, H+ transporting, lysosomal V1 subunit C1(Atp6v1c1)	Mus musculus
Atp6v1d	ATPase, H+ transporting, lysosomal V1 subunit D(Atp6v1d)	Mus musculus

© 2023. Melachroinou K et al. Published online at http://www.aginganddisease.org/EN/10.14336/AD.2023.0614

SUPPLEMENTARY DATA

Atp6v1e1	ATPase, H+ transporting, lysosomal V1 subunit E1(Atp6v1e1)	Mus musculus
Atp6v1h	ATPase, H+ transporting, lysosomal V1 subunit H(Atp6v1h)	Mus musculus
Atp6ap1	ATPase, H+ transporting, lysosomal accessory protein 1(Atp6ap1)	Mus musculus
Atp1a1	ATPase, Na+/K+ transporting, alpha 1 polypeptide(Atp1a1)	Mus musculus
Atp1a2	ATPase, Na+/K+ transporting, alpha 2 polypeptide(Atp1a2)	Mus musculus
Atp1a3	ATPase, Na+/K+ transporting, alpha 3 polypeptide(Atp1a3)	Mus musculus
Atp1b1	ATPase, Na+/K+ transporting, beta 1 polypeptide(Atp1b1)	Mus musculus
Atp1b2	ATPase, Na+/K+ transporting, beta 2 polypeptide(Atp1b2)	Mus musculus
Atp1b3	ATPase, Na+/K+ transporting, beta 3 polypeptide(Atp1b3)	Mus musculus
Atp8a1	ATPase, aminophospholipid transporter (APLT), class I, type 8A, member 1(Atp8a1)	Mus musculus
Atp9a	ATPase, class II, type 9A(Atp9a)	Mus musculus
Auh	AU RNA binding protein/enoyl-coenzyme A hydratase(Auh)	Mus musculus
Cap2	CAP, adenylate cyclase-associated protein, 2 (yeast)(Cap2)	Mus musculus
Cd200	CD200 antigen(Cd200)	Mus musculus
Cd47	CD47 antigen (Rh-related antigen, integrin-associated signal transducer)(Cd47)	Mus musculus
Cd81	CD81 antigen(Cd81)	Mus musculus
Cd82	CD82 antigen(Cd82)	Mus musculus
Cd9	CD9 antigen(Cd9)	Mus musculus
Cisd1	CDGSH iron sulfur domain 1(Cisd1)	Mus musculus
Cdipt	CDP-diacylglycerol--inositol 3-phosphatidyltransferase (phosphatidylinositol synthase)(Cdipt)	Mus musculus
Cops5	COP9 signalosome subunit 5(Cops5)	Mus musculus
Cadps	Ca2+-dependent secretion activator(Cadps)	Mus musculus
Camkv	CaM kinase-like vesicle-associated(Camkv)	Mus musculus
Ddx39b	DEAD (Asp-Glu-Ala-Asp) box polypeptide 39B(Ddx39b)	Mus musculus
Diras2	DIRAS family, GTP-binding RAS-like 2(Diras2)	Mus musculus
D10Jhu81e	DNA segment, Chr 10, Johns Hopkins University 81 expressed(D10Jhu81e)	Mus musculus
Dmxl2	Dmx-like 2(Dmxl2)	Mus musculus
Dnaja1	DnaJ heat shock protein family (Hsp40) member A1(Dnaja1)	Mus musculus
Dnajc16	DnaJ heat shock protein family (Hsp40) member C16(Dnajc16)	Mus musculus
Dnajc5	DnaJ heat shock protein family (Hsp40) member C5(Dnajc5)	Mus musculus
Dnajc6	DnaJ heat shock protein family (Hsp40) member C6(Dnajc6)	Mus musculus
Efhd2	EF hand domain containing 2(Efhd2)	Mus musculus
Edil3	EGF-like repeats and discoidin I-like domains 3(Edil3)	Mus musculus
Ehd3	EH-domain containing 3(Ehd3)	Mus musculus
Emc1	ER membrane protein complex subunit 1(Emc1)	Mus musculus
Epha4	Eph receptor A4(Epha4)	Mus musculus
Fxyd6	FXYD domain-containing ion transport regulator 6(Fxyd6)	Mus musculus
Fyn	Fyn proto-oncogene(Fyn)	Mus musculus
Gpr158	G protein-coupled receptor 158(Gpr158)	Mus musculus

© 2023. Melachroinou K et al. Published online at http://www.aginganddisease.org/EN/10.14336/AD.2023.0614

Gpr37	G protein-coupled receptor 37(Gpr37)	Mus musculus
Gnas	GNAS (guanine nucleotide binding protein, alpha stimulating) complex locus(Gnas)	Mus musculus
Hras	Harvey rat sarcoma virus oncogene(Hras)	Mus musculus
Kras	Kirsten rat sarcoma viral oncogene homolog(Kras)	Mus musculus
L1cam	L1 cell adhesion molecule(L1cam)	Mus musculus
Lmbrd2	LMBR1 domain containing 2(Lmbrd2)	Mus musculus
Lancl1	LanC (bacterial lantibiotic synthetase component C)-like 1(Lancl1)	Mus musculus
Lancl2	LanC (bacterial lantibiotic synthetase component C)-like 2(Lancl2)	Mus musculus
Asah1	N-acylsphingosine amidohydrolase 1(Asah1)	Mus musculus
Napa	N-ethylmaleimide sensitive fusion protein attachment protein alpha(Napa)	Mus musculus
Napb	N-ethylmaleimide sensitive fusion protein attachment protein beta(Napb)	Mus musculus
Nsf	N-ethylmaleimide sensitive fusion protein(Nsf)	Mus musculus
Ndrg1	N-myc downstream regulated gene 1(Ndrg1)	Mus musculus
Ndrg2	N-myc downstream regulated gene 2(Ndrg2)	Mus musculus
Ndufa10	NADH dehydrogenase (ubiquinone) 1 alpha subcomplex 10(Ndufa10)	Mus musculus
Ndufa11	NADH dehydrogenase (ubiquinone) 1 alpha subcomplex 11(Ndufa11)	Mus musculus
Ndufa13	NADH dehydrogenase (ubiquinone) 1 alpha subcomplex, 13(Ndufa13)	Mus musculus
Ndufa2	NADH dehydrogenase (ubiquinone) 1 alpha subcomplex, 2(Ndufa2)	Mus musculus
Ndufa4	NADH dehydrogenase (ubiquinone) 1 alpha subcomplex, 4(Ndufa4)	Mus musculus
Ndufa5	NADH dehydrogenase (ubiquinone) 1 alpha subcomplex, 5(Ndufa5)	Mus musculus
Ndufa6	NADH dehydrogenase (ubiquinone) 1 alpha subcomplex, 6 (B14)(Ndufa6)	Mus musculus
Ndufa8	NADH dehydrogenase (ubiquinone) 1 alpha subcomplex, 8(Ndufa8)	Mus musculus
Ndufa9	NADH dehydrogenase (ubiquinone) 1 alpha subcomplex, 9(Ndufa9)	Mus musculus
Ndufb3	NADH dehydrogenase (ubiquinone) 1 beta subcomplex 3(Ndufb3)	Mus musculus
Ndufb4	NADH dehydrogenase (ubiquinone) 1 beta subcomplex 4(Ndufb4)	Mus musculus
Ndufb8	NADH dehydrogenase (ubiquinone) 1 beta subcomplex 8(Ndufb8)	Mus musculus
Ndufb10	NADH dehydrogenase (ubiquinone) 1 beta subcomplex, 10(Ndufb10)	Mus musculus
Ndufb11	NADH dehydrogenase (ubiquinone) 1 beta subcomplex, 11(Ndufb11)	Mus musculus
Ndufb5	NADH dehydrogenase (ubiquinone) 1 beta subcomplex, 5(Ndufb5)	Mus musculus
Ndufb7	NADH dehydrogenase (ubiquinone) 1 beta subcomplex, 7(Ndufb7)	Mus musculus
Ndufb9	NADH dehydrogenase (ubiquinone) 1 beta subcomplex, 9(Ndufb9)	Mus musculus
Ndufc2	NADH dehydrogenase (ubiquinone) 1, subcomplex unknown, 2(Ndufc2)	Mus musculus
Ndufs1	NADH dehydrogenase (ubiquinone) Fe-S protein 1(Ndufs1)	Mus musculus
Ndufs2	NADH dehydrogenase (ubiquinone) Fe-S protein 2(Ndufs2)	Mus musculus
Ndufs3	NADH dehydrogenase (ubiquinone) Fe-S protein 3(Ndufs3)	Mus musculus
Ndufs5	NADH dehydrogenase (ubiquinone) Fe-S protein 5(Ndufs5)	Mus musculus
Ndufs7	NADH dehydrogenase (ubiquinone) Fe-S protein 7(Ndufs7)	Mus musculus
Ndufs8	NADH dehydrogenase (ubiquinone) Fe-S protein 8(Ndufs8)	Mus musculus
Ndufv1	NADH dehydrogenase (ubiquinone) flavoprotein 1(Ndufv1)	Mus musculus
Nckap1	NCK-associated protein 1(Nckap1)	Mus musculus

© 2023. Melachroinou K et al. Published online at http://www.aginganddisease.org/EN/10.14336/AD.2023.0614

SUPPLEMENTARY DATA

Nme1	NME/NM23 nucleoside diphosphate kinase 1(Nme1)	Mus musculus
Nme2	NME/NM23 nucleoside diphosphate kinase 2(Nme2)	Mus musculus
Nsfl1c	NSFL1 (p97) cofactor (p47)(Nsfl1c)	Mus musculus
Npc1	Niemann-Pick type C1(Npc1)	Mus musculus
Ola1	Obg-like ATPase 1(Ola1)	Mus musculus
Pcp4	Purkinje cell protein 4(Pcp4)	Mus musculus
Rab10	RAB10, member RAS oncogene family(Rab10)	Mus musculus
Rab11a	RAB11A, member RAS oncogene family(Rab11a)	Mus musculus
Rab11b	RAB11B, member RAS oncogene family(Rab11b)	Mus musculus
Rab14	RAB14, member RAS oncogene family(Rab14)	Mus musculus
Rab18	RAB18, member RAS oncogene family(Rab18)	Mus musculus
Rab1A	RAB1A, member RAS oncogene family(Rab1a)	Mus musculus
Rab1b	RAB1B, member RAS oncogene family(Rab1b)	Mus musculus
Rab21	RAB21, member RAS oncogene family(Rab21)	Mus musculus
Rab2a	RAB2A, member RAS oncogene family(Rab2a)	Mus musculus
Rab35	RAB35, member RAS oncogene family(Rab35)	Mus musculus
Rab3a	RAB3A, member RAS oncogene family(Rab3a)	Mus musculus
Rab3c	RAB3C, member RAS oncogene family(Rab3c)	Mus musculus
Rab5a	RAB5A, member RAS oncogene family(Rab5a)	Mus musculus
Rab5b	RAB5B, member RAS oncogene family(Rab5b)	Mus musculus
Rab5c	RAB5C, member RAS oncogene family(Rab5c)	Mus musculus
Rab6a	RAB6A, member RAS oncogene family(Rab6a)	Mus musculus
Rab6b	RAB6B, member RAS oncogene family(Rab6b)	Mus musculus
Rad23b	RAD23 homolog B, nucleotide excision repair protein(Rad23b)	Mus musculus
Ranbp1	RAN binding protein 1(Ranbp1)	Mus musculus
Ran	RAN, member RAS oncogene family(Ran)	Mus musculus
Rap2b	RAP2B, member of RAS oncogene family(Rap2b)	Mus musculus
Rap1b	RAS related protein 1b(Rap1b)	Mus musculus
Rap2a	RAS related protein 2a(Rap2a)	Mus musculus
Rasl2-9	RAS-like, family 2, locus 9(Rasl2-9)	Mus musculus
Rap1gap	Rap1 GTPase-activating protein(Rap1gap)	Mus musculus
Arhgdia	Rho GDP dissociation inhibitor (GDI) alpha(Arhgdia)	Mus musculus
Arhgap1	Rho GTPase activating protein 1(Arhgap1)	Mus musculus
Ahcy	S-adenosylhomocysteine hydrolase(Ahcy)	Mus musculus
Ahcyl1	S-adenosylhomocysteine hydrolase-like 1(Ahcyl1)	Mus musculus
S100b	S100 protein, beta polypeptide, neural(S100b)	Mus musculus
Samm50	SAMM50 sorting and assembly machinery component(Samm50)	Mus musculus
Sec22b	SEC22 homolog B, vesicle trafficking protein(Sec22b)	Mus musculus
Sh3gl2	SH3-domain GRB2-like 2(Sh3gl2)	Mus musculus
Sh3glb2	SH3-domain GRB2-like endophilin B2(Sh3glb2)	Mus musculus

© 2023. Melachroinou K et al. Published online at http://www.aginganddisease.org/EN/10.14336/AD.2023.0614

SUPPLEMENTARY DATA

Sparcl1	SPARC-like 1(Sparcl1)	Mus musculus
Stt3b	STT3, subunit of the oligosaccharyltransferase complex, homolog B (S. cerevisiae)(Stt3b)	Mus musculus
Tufm	Tu translation elongation factor, mitochondrial(Tufm)	Mus musculus
Galnt16	UDP-N-acetyl-alpha-D-galactosamine:polypeptide N-acetylgalactosaminyltransferase 16(Galnt16)	Mus musculus
Galnt2	UDP-N-acetyl-alpha-D-galactosamine:polypeptide N-acetylgalactosaminyltransferase 2(Galnt2)	Mus musculus
Uggt1	UDP-glucose glycoprotein glucosyltransferase 1(Uggt1)	Mus musculus
Vgf	VGF nerve growth factor inducible(Vgf)	Mus musculus
Vps35	VPS35 retromer complex component(Vps35)	Mus musculus
Wdr1	WD repeat domain 1(Wdr1)	Mus musculus
Yes1	YES proto-oncogene 1, Src family tyrosine kinase(Yes1)	Mus musculus
Adam11	a disintegrin and metallopeptidase domain 11(Adam11)	Mus musculus
Adam22	a disintegrin and metallopeptidase domain 22(Adam22)	Mus musculus
Adam23	a disintegrin and metallopeptidase domain 23(Adam23)	Mus musculus
Abhd16a	abhydrolase domain containing 16A(Abhd16a)	Mus musculus
Abi2	abl-interactor 2(Abi2)	Mus musculus
Acat1	acetyl-Coenzyme A acetyltransferase 1(Acat1)	Mus musculus
Anp32a	acidic (leucine-rich) nuclear phosphoprotein 32 family, member A(Anp32a)	Mus musculus
Aco2	aconitase 2, mitochondrial(Aco2)	Mus musculus
Arpc2	actin related protein 2/3 complex, subunit 2(Arpc2)	Mus musculus
Arpc4	actin related protein 2/3 complex, subunit 4(Arpc4)	Mus musculus
Acta1	actin, alpha 1, skeletal muscle(Acta1)	Mus musculus
Acta2	actin, alpha 2, smooth muscle, aorta(Acta2)	Mus musculus
Actg1	actin, gamma, cytoplasmic 1(Actg1)	Mus musculus
Alcam	activated leukocyte cell adhesion molecule(Alcam)	Mus musculus
Acot7	acyl-CoA thioesterase 7(Acot7)	Mus musculus
Ap1b1	adaptor protein complex AP-1, beta 1 subunit(Ap1b1)	Mus musculus
Ap2a1	adaptor-related protein complex 2, alpha 1 subunit(Ap2a1)	Mus musculus
Ap2a2	adaptor-related protein complex 2, alpha 2 subunit(Ap2a2)	Mus musculus
Ap2b1	adaptor-related protein complex 2, beta 1 subunit(Ap2b1)	Mus musculus
Ap2m1	adaptor-related protein complex 2, mu 1 subunit(Ap2m1)	Mus musculus
Ap3b2	adaptor-related protein complex 3, beta 2 subunit(Ap3b2)	Mus musculus
Ap3d1	adaptor-related protein complex 3, delta 1 subunit(Ap3d1)	Mus musculus
Adcy5	adenylate cyclase 5(Adcy5)	Mus musculus
Adcy9	adenylate cyclase 9(Adcy9)	Mus musculus
Adss	adenylosuccinate synthetase, non muscle(Adss)	Mus musculus
Apmap	adipocyte plasma membrane associated protein(Apmap)	Mus musculus
Aars	alanyl-tRNA synthetase(Aars)	Mus musculus
Alb	albumin(Alb)	Mus musculus
Adh5	alcohol dehydrogenase 5 (class III), chi polypeptide(Adh5)	Mus musculus

© 2023. Melachroinou K et al. Published online at http://www.aginganddisease.org/EN/10.14336/AD.2023.0614

SUPPLEMENTARY DATA

Aldh2	aldehyde dehydrogenase 2, mitochondrial(Aldh2)	Mus musculus
Aldh1a7	aldehyde dehydrogenase family 1, subfamily A7(Aldh1a7)	Mus musculus
Aldh5a1	aldhehyde dehydrogenase family 5, subfamily A1(Aldh5a1)	Mus musculus
Akr1a1	aldo-keto reductase family 1, member A1 (aldehyde reductase)(Akr1a1)	Mus musculus
Aldoa	aldolase A, fructose-bisphosphate(Aldoa)	Mus musculus
Aldoc	aldolase C, fructose-bisphosphate(Aldoc)	Mus musculus
Alpl	alkaline phosphatase, liver/bone/kidney(Alpl)	Mus musculus
Ganab	alpha glucosidase 2 alpha neutral subunit(Ganab)	Mus musculus
Npepps	aminopeptidase puromycin sensitive(Npepps)	Mus musculus
Amph	amphiphysin(Amph)	Mus musculus
App	amyloid beta (A4) precursor protein(App)	Mus musculus
Aplp1	amyloid beta (A4) precursor-like protein 1(Aplp1)	Mus musculus
Aplp2	amyloid beta (A4) precursor-like protein 2(Aplp2)	Mus musculus
Ank2	ankyrin 2, brain(Ank2)	Mus musculus
Anxa3	annexin A3(Anxa3)	Mus musculus
Anxa5	annexin A5(Anxa5)	Mus musculus
Anxa6	annexin A6(Anxa6)	Mus musculus
Apod	apolipoprotein D(Apod)	Mus musculus
Apoe	apolipoprotein E(Apoe)	Mus musculus
Aqp4	aquaporin 4(Aqp4)	Mus musculus
Astn1	astrotactin 1(Astn1)	Mus musculus
Atg9a	autophagy related 9A(Atg9a)	Mus musculus
Bsg	basigin(Bsg)	Mus musculus
Bace1	beta-site APP cleaving enzyme 1(Bace1)	Mus musculus
Pygb	brain glycogen phosphorylase(Pygb)	Mus musculus
Bcan	brevican(Bcan)	Mus musculus
Bin1	bridging integrator 1(Bin1)	Mus musculus
Cdh13	cadherin 13(Cdh13)	Mus musculus
Calb1	calbindin 1(Calb1)	Mus musculus
Cacna1e	calcium channel, voltage-dependent, R type, alpha 1E subunit(Cacna1e)	Mus musculus
Cacna2d2	calcium channel, voltage-dependent, alpha 2/delta subunit 2(Cacna2d2)	Mus musculus
Cacna2d1	calcium channel, voltage-dependent, alpha2/delta subunit 1(Cacna2d1)	Mus musculus
Cacna2d3	calcium channel, voltage-dependent, alpha2/delta subunit 3(Cacna2d3)	Mus musculus
Cacng3	calcium channel, voltage-dependent, gamma subunit 3(Cacng3)	Mus musculus
Camk2a	calcium/calmodulin-dependent protein kinase II alpha(Camk2a)	Mus musculus
Camk2g	calcium/calmodulin-dependent protein kinase II gamma(Camk2g)	Mus musculus
Camk2b	calcium/calmodulin-dependent protein kinase II, beta(Camk2b)	Mus musculus
Camk2d	calcium/calmodulin-dependent protein kinase II, delta(Camk2d)	Mus musculus
Canx	calnexin(Canx)	Mus musculus
Capn2	calpain 2(Capn2)	Mus musculus

© 2023. Melachroinou K et al. Published online at http://www.aginganddisease.org/EN/10.14336/AD.2023.0614

SUPPLEMENTARY DATA

Calr	calreticulin(Calr)	Mus musculus
Clstn1	calsyntenin 1(Clstn1)	Mus musculus
Casc4	cancer susceptibility candidate 4(Casc4)	Mus musculus
Cnr1	cannabinoid receptor 1 (brain)(Cnr1)	Mus musculus
Cnrip1	cannabinoid receptor interacting protein 1(Cnrip1)	Mus musculus
Capza2	capping protein (actin filament) muscle Z-line, alpha 2(Capza2)	Mus musculus
Capzb	capping protein (actin filament) muscle Z-line, beta(Capzb)	Mus musculus
Cbr1	carbonyl reductase 1(Cbr1)	Mus musculus
Cpe	carboxypeptidase E(Cpe)	Mus musculus
Crtac1	cartilage acidic protein 1(Crtac1)	Mus musculus
Csnk2a1	casein kinase 2, alpha 1 polypeptide(Csnk2a1)	Mus musculus
Ctnna2	catenin (cadherin associated protein), alpha 2(Ctnna2)	Mus musculus
Ctnnb1	catenin (cadherin associated protein), beta 1(Ctnnb1)	Mus musculus
Ctsd	cathepsin D(Ctsd)	Mus musculus
Cadm1	cell adhesion molecule 1(Cadm1)	Mus musculus
Cdc37	cell division cycle 37(Cdc37)	Mus musculus
Cdc42	cell division cycle 42(Cdc42)	Mus musculus
Cct2	chaperonin containing Tcp1, subunit 2 (beta)(Cct2)	Mus musculus
Cct3	chaperonin containing Tcp1, subunit 3 (gamma)(Cct3)	Mus musculus
Cct4	chaperonin containing Tcp1, subunit 4 (delta)(Cct4)	Mus musculus
Cct5	chaperonin containing Tcp1, subunit 5 (epsilon)(Cct5)	Mus musculus
Cct6a	chaperonin containing Tcp1, subunit 6a (zeta)(Cct6a)	Mus musculus
Cct7	chaperonin containing Tcp1, subunit 7 (eta)(Cct7)	Mus musculus
Cct8	chaperonin containing Tcp1, subunit 8 (theta)(Cct8)	Mus musculus
Chmp6	charged multivesicular body protein 6(Chmp6)	Mus musculus
Chid1	chitinase domain containing 1(Chid1)	Mus musculus
Clcn3	chloride channel, voltage-sensitive 3(Clcn3)	Mus musculus
Cspg5	chondroitin sulfate proteoglycan 5(Cspg5)	Mus musculus
Cntfr	ciliary neurotrophic factor receptor(Cntfr)	Mus musculus
Cs	citrate synthase(Cs)	Mus musculus
Cltc	clathrin, heavy polypeptide (Hc)(Cltc)	Mus musculus
Cldnd1	claudin domain containing 1(Cldnd1)	Mus musculus
Cfl1	cofilin 1, non-muscle(Cfl1)	Mus musculus
Cfl2	cofilin 2, muscle(Cfl2)	Mus musculus
Crmp1	collapsin response mediator protein 1(Crmp1)	Mus musculus
C1qbp	complement component 1, q subcomponent binding protein(C1qbp)	Mus musculus
Cplx2	complexin 2(Cplx2)	Mus musculus
Cntn1	contactin 1(Cntn1)	Mus musculus
Cntn2	contactin 2(Cntn2)	Mus musculus
Cntnap2	contactin associated protein-like 2(Cntnap2)	Mus musculus

Cpne4	copine IV(Cpne4)	Mus musculus
Cpne6	copine VI(Cpne6)	Mus musculus
Cpne9	copine family member IX(Cpne9)	Mus musculus
Coro1b	coronin, actin binding protein 1B(Coro1b)	Mus musculus
Coro1c	coronin, actin binding protein 1C(Coro1c)	Mus musculus
Ckb	creatine kinase, brain(Ckb)	Mus musculus
Ckmt1	creatine kinase, mitochondrial 1, ubiquitous(Ckmt1)	Mus musculus
Crym	crystallin, mu(Crym)	Mus musculus
Ccny	cyclin Y(Ccny)	Mus musculus
Cst3	cystatin C(Cst3)	Mus musculus
Csrp1	cysteine and glycine-rich protein 1(Csrp1)	Mus musculus
Cmpk1	cytidine monophosphate (UMP-CMP) kinase 1(Cmpk1)	Mus musculus
Cox4i1	cytochrome c oxidase subunit IV isoform 1(Cox4i1)	Mus musculus
Cox7a2	cytochrome c oxidase subunit VIIa 2(Cox7a2)	Mus musculus
Cox7c	cytochrome c oxidase subunit VIIc(Cox7c)	Mus musculus
Cox6c	cytochrome c oxidase subunit VIc(Cox6c)	Mus musculus
Cox5a	cytochrome c oxidase subunit Va(Cox5a)	Mus musculus
Cox5b	cytochrome c oxidase subunit Vb(Cox5b)	Mus musculus
Cox6b1	cytochrome c oxidase, subunit VIb polypeptide 1(Cox6b1)	Mus musculus
Cycs	cytochrome c, somatic(Cycs)	Mus musculus
Cyc1	cytochrome c-1(Cyc1)	Mus musculus
Cyfip2	cytoplasmic FMR1 interacting protein 2(Cyfip2)	Mus musculus
Ddb1	damage specific DNA binding protein 1(Ddb1)	Mus musculus
Dad1	defender against cell death 1(Dad1)	Mus musculus
Dstn	destrin(Dstn)	Mus musculus
Dbi	diazepam binding inhibitor(Dbi)	Mus musculus
Dld	dihydrolipoamide dehydrogenase(Dld)	Mus musculus
Dpysl2	dihydropyrimidinase-like 2(Dpysl2)	Mus musculus
Dpysl3	dihydropyrimidinase-like 3(Dpysl3)	Mus musculus
Dpysl4	dihydropyrimidinase-like 4(Dpysl4)	Mus musculus
Dpysl5	dihydropyrimidinase-like 5(Dpysl5)	Mus musculus
Ddah1	dimethylarginine dimethylaminohydrolase 1(Ddah1)	Mus musculus
Dpp10	dipeptidylpeptidase 10(Dpp10)	Mus musculus
Dpp6	dipeptidylpeptidase 6(Dpp6)	Mus musculus
Dlg1	discs, large homolog 1 (Drosophila)(Dlg1)	Mus musculus
Dlg2	discs, large homolog 2 (Drosophila)(Dlg2)	Mus musculus
Dlg4	discs, large homolog 4 (Drosophila)(Dlg4)	Mus musculus
Dpm1	dolichol-phosphate (beta-D) mannosyltransferase 1(Dpm1)	Mus musculus
Ddost	dolichyl-di-phosphooligosaccharide-protein glycotransferase(Ddost)	Mus musculus
Dclk1	doublecortin-like kinase 1(Dclk1)	Mus musculus

© 2023. Melachroinou K et al. Published online at http://www.aginganddisease.org/EN/10.14336/AD.2023.0614

Dpy19l1	dpy-19-like 1 (C. elegans)(Dpy19l1)	Mus musculus
Dctn1	dynactin 1(Dctn1)	Mus musculus
Dctn2	dynactin 2(Dctn2)	Mus musculus
Dnm1	dynamin 1(Dnm1)	Mus musculus
Dnm1l	dynamin 1-like(Dnm1l)	Mus musculus
Dnm3	dynamin 3(Dnm3)	Mus musculus
Dync1h1	dynein cytoplasmic 1 heavy chain 1(Dync1h1)	Mus musculus
Dynll2	dynein light chain LC8-type 2(Dynll2)	Mus musculus
Enpp5	ectonucleotide pyrophosphatase/phosphodiesterase 5(Enpp5)	Mus musculus
Etfa	electron transferring flavoprotein, alpha polypeptide(Etfa)	Mus musculus
Etfdh	electron transferring flavoprotein, dehydrogenase(Etfdh)	Mus musculus
Endod1	endonuclease domain containing 1(Endod1)	Mus musculus
Erp29	endoplasmic reticulum protein 29(Erp29)	Mus musculus
Ergic1	endoplasmic reticulum-golgi intermediate compartment (ERGIC) 1(Ergic1)	Mus musculus
Eno1	enolase 1, alpha non-neuron(Eno1)	Mus musculus
Eno2	enolase 2, gamma neuronal(Eno2)	Mus musculus
Echs1	enoyl Coenzyme A hydratase, short chain, 1, mitochondrial(Echs1)	Mus musculus
Ephx1	epoxide hydrolase 1, microsomal(Ephx1)	Mus musculus
Epb41l1	erythrocyte membrane protein band 4.1 like 1(Epb41l1)	Mus musculus
Epb41l2	erythrocyte membrane protein band 4.1 like 2(Epb41l2)	Mus musculus
Epb41l3	erythrocyte membrane protein band 4.1 like 3(Epb41l3)	Mus musculus
Esd	esterase D/formylglutathione hydrolase(Esd)	Mus musculus
Eef1a1	eukaryotic translation elongation factor 1 alpha 1(Eef1a1)	Mus musculus
Eef1a2	eukaryotic translation elongation factor 1 alpha 2(Eef1a2)	Mus musculus
Eef1g	eukaryotic translation elongation factor 1 gamma(Eef1g)	Mus musculus
Eef2	eukaryotic translation elongation factor 2(Eef2)	Mus musculus
Eif4a2	eukaryotic translation initiation factor 4A2(Eif4a2)	Mus musculus
Eif5a	eukaryotic translation initiation factor 5A(Eif5a)	Mus musculus
Fam213a	family with sequence similarity 213, member A(Fam213a)	Mus musculus
Fam3c	family with sequence similarity 3, member C(Fam3c)	Mus musculus
Fscn1	fascin actin-bundling protein 1(Fscn1)	Mus musculus
Fasn	fatty acid synthase(Fasn)	Mus musculus
Frrs1l	ferric-chelate reductase 1 like(Frrs1l)	Mus musculus
Ftl1	ferritin light polypeptide 1(Ftl1)	Mus musculus
Flot2	flotillin 2(Flot2)	Mus musculus
Folh1	folate hydrolase 1(Folh1)	Mus musculus
Gabra1	gamma-aminobutyric acid (GABA) A receptor, subunit alpha 1(Gabra1)	Mus musculus
Gabra2	gamma-aminobutyric acid (GABA) A receptor, subunit alpha 2(Gabra2)	Mus musculus
Gabra3	gamma-aminobutyric acid (GABA) A receptor, subunit alpha 3(Gabra3)	Mus musculus
Gabra4	gamma-aminobutyric acid (GABA) A receptor, subunit alpha 4(Gabra4)	Mus musculus

© 2023. Melachroinou K et al. Published online at http://www.aginganddisease.org/EN/10.14336/AD.2023.0614

SUPPLEMENTARY DATA

Gabrb1	gamma-aminobutyric acid (GABA) A receptor, subunit beta 1(Gabrb1)	Mus musculus
Gabrb2	gamma-aminobutyric acid (GABA) A receptor, subunit beta 2(Gabrb2)	Mus musculus
Gabrb3	gamma-aminobutyric acid (GABA) A receptor, subunit beta 3(Gabrb3)	Mus musculus
Gabrg2	gamma-aminobutyric acid (GABA) A receptor, subunit gamma 2(Gabrg2)	Mus musculus
Gabbr1	gamma-aminobutyric acid (GABA) B receptor, 1(Gabbr1)	Mus musculus
Ggt7	gamma-glutamyltransferase 7(Ggt7)	Mus musculus
Gja1	gap junction protein, alpha 1(Gja1)	Mus musculus
Gphn	gephyrin(Gphn)	Mus musculus
Gmfb	glia maturation factor, beta(Gmfb)	Mus musculus
Gad1	glutamate decarboxylase 1(Gad1)	Mus musculus
Glud1	glutamate dehydrogenase 1(Glud1)	Mus musculus
Gria1	glutamate receptor, ionotropic, AMPA1 (alpha 1)(Gria1)	Mus musculus
Gria2	glutamate receptor, ionotropic, AMPA2 (alpha 2)(Gria2)	Mus musculus
Gria3	glutamate receptor, ionotropic, AMPA3 (alpha 3)(Gria3)	Mus musculus
Gria4	glutamate receptor, ionotropic, AMPA4 (alpha 4)(Gria4)	Mus musculus
Grin2a	glutamate receptor, ionotropic, NMDA2A (epsilon 1)(Grin2a)	Mus musculus
Grin2b	glutamate receptor, ionotropic, NMDA2B (epsilon 2)(Grin2b)	Mus musculus
Grik2	glutamate receptor, ionotropic, kainate 2 (beta 2)(Grik2)	Mus musculus
Grm1	glutamate receptor, metabotropic 1(Grm1)	Mus musculus
Grm3	glutamate receptor, metabotropic 3(Grm3)	Mus musculus
Grm5	glutamate receptor, metabotropic 5(Grm5)	Mus musculus
Grm7	glutamate receptor, metabotropic 7(Grm7)	Mus musculus
Glul	glutamate-ammonia ligase (glutamine synthetase)(Glul)	Mus musculus
Got2	glutamatic-oxaloacetic transaminase 2, mitochondrial(Got2)	Mus musculus
Gad2	glutamic acid decarboxylase 2(Gad2)	Mus musculus
Got1	glutamic-oxaloacetic transaminase 1, soluble(Got1)	Mus musculus
Gstm1	glutathione S-transferase, mu 1(Gstm1)	Mus musculus
Gstm5	glutathione S-transferase, mu 5(Gstm5)	Mus musculus
Gstp1	glutathione S-transferase, pi 1(Gstp1)	Mus musculus
Gstp2	glutathione S-transferase, pi 2(Gstp2)	Mus musculus
Gapdh	glyceraldehyde-3-phosphate dehydrogenase(Gapdh)	Mus musculus
Gpd2	glycerol phosphate dehydrogenase 2, mitochondrial(Gpd2)	Mus musculus
Gpd1	glycerol-3-phosphate dehydrogenase 1 (soluble)(Gpd1)	Mus musculus
Gpd1l	glycerol-3-phosphate dehydrogenase 1-like(Gpd1l)	Mus musculus
Gpm6a	glycoprotein m6a(Gpm6a)	Mus musculus
Gpm6b	glycoprotein m6b(Gpm6b)	Mus musculus
Glo1	glyoxalase 1(Glo1)	Mus musculus
Glod4	glyoxalase domain containing 4(Glod4)	Mus musculus
Gpc1	glypican 1(Gpc1)	Mus musculus
Golga7	golgi autoantigen, golgin subfamily a, 7(Golga7)	Mus musculus

© 2023. Melachroinou K et al. Published online at http://www.aginganddisease.org/EN/10.14336/AD.2023.0614

SUPPLEMENTARY DATA

GOLGA7B	golgi autoantigen, golgin subfamily a, 7B(Golga7b)	Mus musculus
Gap43	growth associated protein 43(Gap43)	Mus musculus
Grb2	growth factor receptor bound protein 2(Grb2)	Mus musculus
Gda	guanine deaminase(Gda)	Mus musculus
Gnai2	guanine nucleotide binding protein (G protein), alpha inhibiting 2(Gnai2)	Mus musculus
Gnai3	guanine nucleotide binding protein (G protein), alpha inhibiting 3(Gnai3)	Mus musculus
Gnb1	guanine nucleotide binding protein (G protein), beta 1(Gnb1)	Mus musculus
Gnb2	guanine nucleotide binding protein (G protein), beta 2(Gnb2)	Mus musculus
Gnb4	guanine nucleotide binding protein (G protein), beta 4(Gnb4)	Mus musculus
Gnb5	guanine nucleotide binding protein (G protein), beta 5(Gnb5)	Mus musculus
Gng3	guanine nucleotide binding protein (G protein), gamma 3(Gng3)	Mus musculus
Gna11	guanine nucleotide binding protein, alpha 11(Gna11)	Mus musculus
Gna13	guanine nucleotide binding protein, alpha 13(Gna13)	Mus musculus
Gnao1	guanine nucleotide binding protein, alpha O(Gnao1)	Mus musculus
Gnaq	guanine nucleotide binding protein, alpha q polypeptide(Gnaq)	Mus musculus
Gnal	guanine nucleotide binding protein, alpha stimulating, olfactory type(Gnal)	Mus musculus
Gdi1	guanosine diphosphate (GDP) dissociation inhibitor 1(Gdi1)	Mus musculus
Gdi2	guanosine diphosphate (GDP) dissociation inhibitor 2(Gdi2)	Mus musculus
Gucy1b3	guanylate cyclase 1, soluble, beta 3(Gucy1b3)	Mus musculus
Hsph1	heat shock 105kDa/110kDa protein 1(Hsph1)	Mus musculus
Hspe1	heat shock protein 1 (chaperonin 10)(Hspe1)	Mus musculus
Hspd1	heat shock protein 1 (chaperonin)(Hspd1)	Mus musculus
Hspa1l	heat shock protein 1-like(Hspa1l)	Mus musculus
Hspa12a	heat shock protein 12A(Hspa12a)	Mus musculus
Hspa4l	heat shock protein 4 like(Hspa4l)	Mus musculus
Hspa4	heat shock protein 4(Hspa4)	Mus musculus
Hspa5	heat shock protein 5(Hspa5)	Mus musculus
Hspa8	heat shock protein 8(Hspa8)	Mus musculus
Hspa9	heat shock protein 9(Hspa9)	Mus musculus
Hsp90ab1	heat shock protein 90 alpha (cytosolic), class B member 1(Hsp90ab1)	Mus musculus
Hsp90aa1	heat shock protein 90, alpha (cytosolic), class A member 1(Hsp90aa1)	Mus musculus
Hsp90b1	heat shock protein 90, beta (Grp94), member 1(Hsp90b1)	Mus musculus
Hbb-b1	hemoglobin, beta adult major chain(Hbb-b1)	Mus musculus
Hepacam	hepatocyte cell adhesion molecule(Hepacam)	Mus musculus
Hnrnpa1	heterogeneous nuclear ribonucleoprotein A1(Hnrnpa1)	Mus musculus
Hnrnpa2b1	heterogeneous nuclear ribonucleoprotein A2/B1(Hnrnpa2b1)	Mus musculus
Hnrnpd	heterogeneous nuclear ribonucleoprotein D(Hnrnpd)	Mus musculus
Hnrnpk	heterogeneous nuclear ribonucleoprotein K(Hnrnpk)	Mus musculus
Hk1	hexokinase 1(Hk1)	Mus musculus
Hmgb1	high mobility group box 1(Hmgb1)	Mus musculus

© 2023. Melachroinou K et al. Published online at http://www.aginganddisease.org/EN/10.14336/AD.2023.0614

SUPPLEMENTARY DATA

Hpca	hippocalcin(Hpca)	Mus musculus
Hpcal1	hippocalcin-like 1(Hpcal1)	Mus musculus
Hpcal4	hippocalcin-like 4(Hpcal4)	Mus musculus
Hint1	histidine triad nucleotide binding protein 1(Hint1)	Mus musculus
Hist1h4a	histone cluster 1, H4a(Hist1h4a)	Mus musculus
Hapln4	hyaluronan and proteoglycan link protein 4(Hapln4)	Mus musculus
Hsd17b4	hydroxysteroid (17-beta) dehydrogenase 4(Hsd17b4)	Mus musculus
Hcn2	hyperpolarization-activated, cyclic nucleotide-gated K+ 2(Hcn2)	Mus musculus
Hyou1	hypoxia up-regulated 1(Hyou1)	Mus musculus
Ilvbl	ilvB (bacterial acetolactate synthase)-like(Ilvbl)	Mus musculus
Igsf21	immunoglobulin superfamily, member 21(Igsf21)	Mus musculus
Immt	inner membrane protein, mitochondrial(Immt)	Mus musculus
Impa1	inositol (myo)-1(or 4)-monophosphatase 1(Impa1)	Mus musculus
Itpr1	inositol 1,4,5-trisphosphate receptor 1(Itpr1)	Mus musculus
Impad1	inositol monophosphatase domain containing 1(Impad1)	Mus musculus
Inpp5a	inositol polyphosphate-5-phosphatase A(Inpp5a)	Mus musculus
Itgb1	integrin beta 1 (fibronectin receptor beta)(Itgb1)	Mus musculus
Itgb8	integrin beta 8(Itgb8)	Mus musculus
Icam5	intercellular adhesion molecule 5, telencephalin(Icam5)	Mus musculus
Idh1	isocitrate dehydrogenase 1 (NADP+), soluble(Idh1)	Mus musculus
Idh2	isocitrate dehydrogenase 2 (NADP+), mitochondrial(Idh2)	Mus musculus
Idh3a	isocitrate dehydrogenase 3 (NAD+) alpha(Idh3a)	Mus musculus
Idh3g	isocitrate dehydrogenase 3 (NAD+), gamma(Idh3g)	Mus musculus
Jam3	junction adhesion molecule 3(Jam3)	Mus musculus
Kpnb1	karyopherin (importin) beta 1(Kpnb1)	Mus musculus
Kif5a	kinesin family member 5A(Kif5a)	Mus musculus
Kif5c	kinesin family member 5C(Kif5c)	Mus musculus
Lactb	lactamase, beta(Lactb)	Mus musculus
Ldha	lactate dehydrogenase A(Ldha)	Mus musculus
Ldhb	lactate dehydrogenase B(Ldhb)	Mus musculus
Lxn	latexin(Lxn)	Mus musculus
Lman2	lectin, mannose-binding 2(Lman2)	Mus musculus
Lap3	leucine aminopeptidase 3(Lap3)	Mus musculus
Lingo1	leucine rich repeat and Ig domain containing 1(Lingo1)	Mus musculus
Lrrc57	leucine rich repeat containing 57(Lrrc57)	Mus musculus
Lrrc8a	leucine rich repeat containing 8A(Lrrc8a)	Mus musculus
Lgi1	leucine-rich repeat LGI family, member 1(Lgi1)	Mus musculus
Lgi2	leucine-rich repeat LGI family, member 2(Lgi2)	Mus musculus
Lgi3	leucine-rich repeat LGI family, member 3(Lgi3)	Mus musculus
Lsamp	limbic system-associated membrane protein(Lsamp)	Mus musculus

© 2023. Melachroinou K et al. Published online at http://www.aginganddisease.org/EN/10.14336/AD.2023.0614

SUPPLEMENTARY DATA

Lin7a	lin-7 homolog A (C. elegans)(Lin7a)	Mus musculus
Lin7c	lin-7 homolog C (C. elegans)(Lin7c)	Mus musculus
Lrpap1	low density lipoprotein receptor-related protein associated protein 1(Lrpap1)	Mus musculus
Ly6h	lymphocyte antigen 6 complex, locus H(Ly6h)	Mus musculus
Lclat1	lysocardiolipin acyltransferase 1(Lclat1)	Mus musculus
Lamp1	lysosomal-associated membrane protein 1(Lamp1)	Mus musculus
Lamp2	lysosomal-associated membrane protein 2(Lamp2)	Mus musculus
Mdh1	malate dehydrogenase 1, NAD (soluble)(Mdh1)	Mus musculus
Mdh2	malate dehydrogenase 2, NAD (mitochondrial)(Mdh2)	Mus musculus
Mpp2	membrane protein, palmitoylated 2 (MAGUK p55 subfamily member 2)(Mpp2)	Mus musculus
Mpp6	membrane protein, palmitoylated 6 (MAGUK p55 subfamily member 6)(Mpp6)	Mus musculus
Mat2a	methionine adenosyltransferase II, alpha(Mat2a)	Mus musculus
Mgst3	microsomal glutathione S-transferase 3(Mgst3)	Mus musculus
Map1a	microtubule-associated protein 1 A(Map1a)	Mus musculus
Map1lc3b	microtubule-associated protein 1 light chain 3 beta(Map1lc3b)	Mus musculus
Map1b	microtubule-associated protein 1B(Map1b)	Mus musculus
Map2	microtubule-associated protein 2(Map2)	Mus musculus
Mapt	microtubule-associated protein tau(Mapt)	Mus musculus
Mapre3	microtubule-associated protein, RP/EB family, member 3(Mapre3)	Mus musculus
Mtch2	mitochondrial carrier 2(Mtch2)	Mus musculus
Mpc1	mitochondrial pyruvate carrier 1(Mpc1)	Mus musculus
Mapk1	mitogen-activated protein kinase 1(Mapk1)	Mus musculus
Map2k1	mitogen-activated protein kinase kinase 1(Map2k1)	Mus musculus
Maoa	monoamine oxidase A(Maoa)	Mus musculus
Pygm	muscle glycogen phosphorylase(Pygm)	Mus musculus
Mal	myelin and lymphocyte protein, T cell differentiation protein(Mal)	Mus musculus
Mbp	myelin basic protein(Mbp)	Mus musculus
Mog	myelin oligodendrocyte glycoprotein(Mog)	Mus musculus
Mag	myelin-associated glycoprotein(Mag)	Mus musculus
Myadm	myeloid-associated differentiation marker(Myadm)	Mus musculus
Isyna1	myo-inositol 1-phosphate synthase A1(Isyna1)	Mus musculus
Myo1d	myosin ID(Myo1d)	Mus musculus
Myh10	myosin, heavy polypeptide 10, non-muscle(Myh10)	Mus musculus
Myl12b	myosin, light chain 12B, regulatory(Myl12b)	Mus musculus
Mtpn	myotrophin(Mtpn)	Mus musculus
Marcks	myristoylated alanine rich protein kinase C substrate(Marcks)	Mus musculus
Neo1	neogenin(Neo1)	Mus musculus
Ncam1	neural cell adhesion molecule 1(Ncam1)	Mus musculus
Nrxn1	neurexin I(Nrxn1)	Mus musculus
Nrxn2	neurexin II(Nrxn2)	Mus musculus

© 2023. Melachroinou K et al. Published online at http://www.aginganddisease.org/EN/10.14336/AD.2023.0614

SUPPLEMENTARY DATA

Nrxn3	neurexin III(Nrxn3)	Mus musculus
Ncan	neurocan(Ncan)	Mus musculus
Ncdn	neurochondrin(Ncdn)	Mus musculus
Nfasc	neurofascin(Nfasc)	Mus musculus
Nlgn2	neuroligin 2(Nlgn2)	Mus musculus
Nlgn3	neuroligin 3(Nlgn3)	Mus musculus
Ncs1	neuronal calcium sensor 1(Ncs1)	Mus musculus
Nrcam	neuronal cell adhesion molecule(Nrcam)	Mus musculus
Negr1	neuronal growth regulator 1(Negr1)	Mus musculus
Ngef	neuronal guanine nucleotide exchange factor(Ngef)	Mus musculus
Nptx1	neuronal pentraxin 1(Nptx1)	Mus musculus
Nptxr	neuronal pentraxin receptor(Nptxr)	Mus musculus
Nptn	neuroplastin(Nptn)	Mus musculus
Ntm	neurotrimin(Ntm)	Mus musculus
Ntrk2	neurotrophic tyrosine kinase, receptor, type 2(Ntrk2)	Mus musculus
Ncstn	nicastrin(Ncstn)	Mus musculus
Nipsnap1	nipsnap homolog 1 (C. elegans)(Nipsnap1)	Mus musculus
Nomo1	nodal modulator 1(Nomo1)	Mus musculus
Nutf2	nuclear transport factor 2(Nutf2)	Mus musculus
Nucb1	nucleobindin 1(Nucb1)	Mus musculus
Nap1l1	nucleosome assembly protein 1-like 1(Nap1l1)	Mus musculus
Nap1l4	nucleosome assembly protein 1-like 4(Nap1l4)	Mus musculus
Olfm1	olfactomedin 1(Olfm1)	Mus musculus
Opa1	optic atrophy 1(Opa1)	Mus musculus
Ogdh	oxoglutarate (alpha-ketoglutarate) dehydrogenase (lipoamide)(Ogdh)	Mus musculus
Pak1	p21 protein (Cdc42/Rac)-activated kinase 1(Pak1)	Mus musculus
Ppt1	palmitoyl-protein thioesterase 1(Ppt1)	Mus musculus
Palm	paralemmin(Palm)	Mus musculus
Pepd	peptidase D(Pepd)	Mus musculus
Ppia	peptidylprolyl isomerase A(Ppia)	Mus musculus
Ppid	peptidylprolyl isomerase D (cyclophilin D)(Ppid)	Mus musculus
Prdx1	peroxiredoxin 1(Prdx1)	Mus musculus
Prdx2	peroxiredoxin 2(Prdx2)	Mus musculus
Prdx3	peroxiredoxin 3(Prdx3)	Mus musculus
Prdx4	peroxiredoxin 4(Prdx4)	Mus musculus
Prdx5	peroxiredoxin 5(Prdx5)	Mus musculus
Prdx6	peroxiredoxin 6(Prdx6)	Mus musculus
Pebp1	phosphatidylethanolamine binding protein 1(Pebp1)	Mus musculus
Pgs1	phosphatidylglycerophosphate synthase 1(Pgs1)	Mus musculus
Pi4ka	phosphatidylinositol 4-kinase, catalytic, alpha polypeptide(Pi4ka)	Mus musculus

© 2023. Melachroinou K et al. Published online at http://www.aginganddisease.org/EN/10.14336/AD.2023.0614

SUPPLEMENTARY DATA

Pigk	phosphatidylinositol glycan anchor biosynthesis, class K(Pigk)	Mus musculus
Pigs	phosphatidylinositol glycan anchor biosynthesis, class S(Pigs)	Mus musculus
Pigt	phosphatidylinositol glycan anchor biosynthesis, class T(Pigt)	Mus musculus
Pigu	phosphatidylinositol glycan anchor biosynthesis, class U(Pigu)	Mus musculus
Pitpna	phosphatidylinositol transfer protein, alpha(Pitpna)	Mus musculus
Pitpnc1	phosphatidylinositol transfer protein, cytoplasmic 1(Pitpnc1)	Mus musculus
Pip4k2b	phosphatidylinositol-5-phosphate 4-kinase, type II, beta(Pip4k2b)	Mus musculus
Pde1a	phosphodiesterase 1A, calmodulin-dependent(Pde1a)	Mus musculus
Pde1b	phosphodiesterase 1B, Ca2+-calmodulin dependent(Pde1b)	Mus musculus
Pde2a	phosphodiesterase 2A, cGMP-stimulated(Pde2a)	Mus musculus
Pfkl	phosphofructokinase, liver, B-type(Pfkl)	Mus musculus
Pfkm	phosphofructokinase, muscle(Pfkm)	Mus musculus
Pfkp	phosphofructokinase, platelet(Pfkp)	Mus musculus
Pgm1	phosphoglucomutase 1(Pgm1)	Mus musculus
Pgm2l1	phosphoglucomutase 2-like 1(Pgm2l1)	Mus musculus
Pgk1	phosphoglycerate kinase 1(Pgk1)	Mus musculus
Pgam1	phosphoglycerate mutase 1(Pgam1)	Mus musculus
Phpt1	phosphohistidine phosphatase 1(Phpt1)	Mus musculus
Plcb1	phospholipase C, beta 1(Plcb1)	Mus musculus
Plch2	phospholipase C, eta 2(Plch2)	Mus musculus
Pld3	phospholipase D family, member 3(Pld3)	Mus musculus
Prps1	phosphoribosyl pyrophosphate synthetase 1(Prps1)	Mus musculus
Paics	phosphoribosylaminoimidazole carboxylase, phosphoribosylaminoribosylaminoimidazole, succinocarboxamide synthetase(Paics)	Mus musculus
Psat1	phosphoserine aminotransferase 1(Psat1)	Mus musculus
Phyhip	phytanoyl-CoA hydroxylase interacting protein(Phyhip)	Mus musculus
Pls3	plastin 3 (T-isoform)(Pls3)	Mus musculus
Pafah1b1	platelet-activating factor acetylhydrolase, isoform 1b, subunit 1(Pafah1b1)	Mus musculus
Psd3	pleckstrin and Sec7 domain containing 3(Psd3)	Mus musculus
Plxna1	plexin A1(Plxna1)	Mus musculus
Plxnb2	plexin B2(Plxnb2)	Mus musculus
Porcn	porcupine homolog (Drosophila)(Porcn)	Mus musculus
Kctd16	potassium channel tetramerisation domain containing 16(Kctd16)	Mus musculus
Kcnma1	potassium large conductance calcium-activated channel, subfamily M, alpha member 1(Kcnma1)	Mus musculus
Kcnab2	potassium voltage-gated channel, shaker-related subfamily, beta member 2(Kcnab2)	Mus musculus
Kcna1	potassium voltage-gated channel, shaker-related subfamily, member 1(Kcna1)	Mus musculus
Kcna2	potassium voltage-gated channel, shaker-related subfamily, member 2(Kcna2)	Mus musculus
Pcyox1l	prenylcysteine oxidase 1 like(Pcyox1l)	Mus musculus
Pcyox1	prenylcysteine oxidase 1(Pcyox1)	Mus musculus
Prnp	prion protein(Prnp)	Mus musculus

© 2023. Melachroinou K et al. Published online at http://www.aginganddisease.org/EN/10.14336/AD.2023.0614

Pfn2	profilin 2(Pfn2)	Mus musculus
Pdcd6ip	programmed cell death 6 interacting protein(Pdcd6ip)	Mus musculus
Phb2	prohibitin 2(Phb2)	Mus musculus
Phb	prohibitin(Phb)	Mus musculus
Preb	prolactin regulatory element binding(Preb)	Mus musculus
Prrt3	proline-rich transmembrane protein 3(Prrt3)	Mus musculus
Prep	prolyl endopeptidase(Prep)	Mus musculus
Prom1	prominin 1(Prom1)	Mus musculus
Pcsk2	proprotein convertase subtilisin/kexin type 2(Pcsk2)	Mus musculus
Psap	prosaposin(Psap)	Mus musculus
Ptges3	prostaglandin E synthase 3 (cytosolic)(Ptges3)	Mus musculus
Ptgfrn	prostaglandin F2 receptor negative regulator(Ptgfrn)	Mus musculus
Psma1	proteasome (prosome, macropain) subunit, alpha type 1(Psma1)	Mus musculus
Psma2	proteasome (prosome, macropain) subunit, alpha type 2(Psma2)	Mus musculus
Psma3	proteasome (prosome, macropain) subunit, alpha type 3(Psma3)	Mus musculus
Psma4	proteasome (prosome, macropain) subunit, alpha type 4(Psma4)	Mus musculus
Psma5	proteasome (prosome, macropain) subunit, alpha type 5(Psma5)	Mus musculus
Psma6	proteasome (prosome, macropain) subunit, alpha type 6(Psma6)	Mus musculus
Psma7	proteasome (prosome, macropain) subunit, alpha type 7(Psma7)	Mus musculus
Psmb1	proteasome (prosome, macropain) subunit, beta type 1(Psmb1)	Mus musculus
Psmb3	proteasome (prosome, macropain) subunit, beta type 3(Psmb3)	Mus musculus
Psmb4	proteasome (prosome, macropain) subunit, beta type 4(Psmb4)	Mus musculus
Psmb5	proteasome (prosome, macropain) subunit, beta type 5(Psmb5)	Mus musculus
Psmb6	proteasome (prosome, macropain) subunit, beta type 6(Psmb6)	Mus musculus
Psmb7	proteasome (prosome, macropain) subunit, beta type 7(Psmb7)	Mus musculus
Pofut2	protein O-fucosyltransferase 2(Pofut2)	Mus musculus
Prmt1	protein arginine N-methyltransferase 1(Prmt1)	Mus musculus
Prmt8	protein arginine N-methyltransferase 8(Prmt8)	Mus musculus
Pdia3	protein disulfide isomerase associated 3(Pdia3)	Mus musculus
Pdia4	protein disulfide isomerase associated 4(Pdia4)	Mus musculus
Pdia6	protein disulfide isomerase associated 6(Pdia6)	Mus musculus
Pacsin1	protein kinase C and casein kinase substrate in neurons 1(Pacsin1)	Mus musculus
Prkca	protein kinase C, alpha(Prkca)	Mus musculus
Prkcb	protein kinase C, beta(Prkcb)	Mus musculus
Prkcg	protein kinase C, gamma(Prkcg)	Mus musculus
Prkar2a	protein kinase, cAMP dependent regulatory, type II alpha(Prkar2a)	Mus musculus
Prkar2b	protein kinase, cAMP dependent regulatory, type II beta(Prkar2b)	Mus musculus
Prkaca	protein kinase, cAMP dependent, catalytic, alpha(Prkaca)	Mus musculus
Prkacb	protein kinase, cAMP dependent, catalytic, beta(Prkacb)	Mus musculus
Ppp1cb	protein phosphatase 1, catalytic subunit, beta isoform(Ppp1cb)	Mus musculus

© 2023. Melachroinou K et al. Published online at http://www.aginganddisease.org/EN/10.14336/AD.2023.0614

Ppp1cc	protein phosphatase 1, catalytic subunit, gamma isoform(Ppp1cc)	Mus musculus
Ppp1r7	protein phosphatase 1, regulatory (inhibitor) subunit 7(Ppp1r7)	Mus musculus
Ppp2ca	protein phosphatase 2 (formerly 2A), catalytic subunit, alpha isoform(Ppp2ca)	Mus musculus
Ppp2r2a	protein phosphatase 2, regulatory subunit B, alpha(Ppp2r2a)	Mus musculus
Ppp3ca	protein phosphatase 3, catalytic subunit, alpha isoform(Ppp3ca)	Mus musculus
Ppp3r1	protein phosphatase 3, regulatory subunit B, alpha isoform (calcineurin B, type I)(Ppp3r1)	Mus musculus
Ptpn11	protein tyrosine phosphatase, non-receptor type 11(Ptpn11)	Mus musculus
Ptpn9	protein tyrosine phosphatase, non-receptor type 9(Ptpn9)	Mus musculus
Ptprz1	protein tyrosine phosphatase, receptor type Z, polypeptide 1(Ptprz1)	Mus musculus
Ptpra	protein tyrosine phosphatase, receptor type, A(Ptpra)	Mus musculus
Ptprd	protein tyrosine phosphatase, receptor type, D(Ptprd)	Mus musculus
Ptprn2	protein tyrosine phosphatase, receptor type, N polypeptide 2(Ptprn2)	Mus musculus
Ptprn	protein tyrosine phosphatase, receptor type, N(Ptprn)	Mus musculus
Ptprs	protein tyrosine phosphatase, receptor type, S(Ptprs)	Mus musculus
Pcmt1	protein-L-isoaspartate (D-aspartate) O-methyltransferase 1(Pcmt1)	Mus musculus
Plp1	proteolipid protein (myelin) 1(Plp1)	Mus musculus
Ptma	prothymosin alpha(Ptma)	Mus musculus
Pura	purine rich element binding protein A(Pura)	Mus musculus
Pdxk	pyridoxal (pyridoxine, vitamin B6) kinase(Pdxk)	Mus musculus
Pdxp	pyridoxal (pyridoxine, vitamin B6) phosphatase(Pdxp)	Mus musculus
Pkm	pyruvate kinase, muscle(Pkm)	Mus musculus
Qdpr	quinoid dihydropteridine reductase(Qdpr)	Mus musculus
Rph3a	rabphilin 3A(Rph3a)	Mus musculus
Rhoa	ras homolog family member A(Rhoa)	Mus musculus
Rhob	ras homolog family member B(Rhob)	Mus musculus
Reep5	receptor accessory protein 5(Reep5)	Mus musculus
Rgs7	regulator of G protein signaling 7(Rgs7)	Mus musculus
Rras	related RAS viral (r-ras) oncogene(Rras)	Mus musculus
Rtn1	reticulon 1(Rtn1)	Mus musculus
Rtn3	reticulon 3(Rtn3)	Mus musculus
Rtn4	reticulon 4(Rtn4)	Mus musculus
Rpn2	ribophorin II(Rpn2)	Mus musculus
Rpl10a	ribosomal protein L10A(Rpl10a)	Mus musculus
Rps27a	ribosomal protein S27A(Rps27a)	Mus musculus
Ryr2	ryanodine receptor 2, cardiac(Ryr2)	Mus musculus
Scarb2	scavenger receptor class B, member 2(Scarb2)	Mus musculus
Scrn1	secernin 1(Scrn1)	Mus musculus
Sez6l2	seizure related 6 homolog like 2(Sez6l2)	Mus musculus
Sez6l	seizure related 6 homolog like(Sez6l)	Mus musculus

© 2023. Melachroinou K et al. Published online at http://www.aginganddisease.org/EN/10.14336/AD.2023.0614

Sel1l	sel-1 suppressor of lin-12-like (C. elegans)(Sel1l)	Mus musculus
Sema4a	sema domain, immunoglobulin domain (Ig), transmembrane domain (TM) and short cytoplasmic domain, (semaphorin) 4A(Sema4a)	Mus musculus
Serpini1	serine (or cysteine) peptidase inhibitor, clade I, member 1(Serpini1)	Mus musculus
Serinc1	serine incorporator 1(Serinc1)	Mus musculus
Srr	serine racemase(Srr)	Mus musculus
Strap	serine/threonine kinase receptor associated protein(Strap)	Mus musculus
Sfxn1	sideroflexin 1(Sfxn1)	Mus musculus
Sfxn3	sideroflexin 3(Sfxn3)	Mus musculus
Sfxn5	sideroflexin 5(Sfxn5)	Mus musculus
Sigmar1	sigma non-opioid intracellular receptor 1(Sigmar1)	Mus musculus
Spcs3	signal peptidase complex subunit 3 homolog (S. cerevisiae)(Spcs3)	Mus musculus
Ssr4	signal sequence receptor, delta(Ssr4)	Mus musculus
Sirpa	signal-regulatory protein alpha(Sirpa)	Mus musculus
Sirt2	sirtuin 2(Sirt2)	Mus musculus
Scn4b	sodium channel, type IV, beta(Scn4b)	Mus musculus
Scn1b	sodium channel, voltage-gated, type I, beta(Scn1b)	Mus musculus
Scn2b	sodium channel, voltage-gated, type II, beta(Scn2b)	Mus musculus
Scn3b	sodium channel, voltage-gated, type III, beta(Scn3b)	Mus musculus
Slc1a2	solute carrier family 1 (glial high affinity glutamate transporter), member 2(Slc1a2)	Mus musculus
Slc1a3	solute carrier family 1 (glial high affinity glutamate transporter), member 3(Slc1a3)	Mus musculus
Slc1a4	solute carrier family 1 (glutamate/neutral amino acid transporter), member 4(Slc1a4)	Mus musculus
Slc1a1	solute carrier family 1 (neuronal/epithelial high affinity glutamate transporter, system Xag), member 1(Slc1a1)	Mus musculus
Slc12a5	solute carrier family 12, member 5(Slc12a5)	Mus musculus
Slc12a6	solute carrier family 12, member 6(Slc12a6)	Mus musculus
Slc15a2	solute carrier family 15 (H+/peptide transporter), member 2(Slc15a2)	Mus musculus
Slc16a1	solute carrier family 16 (monocarboxylic acid transporters), member 1(Slc16a1)	Mus musculus
Slc17a6	solute carrier family 17 (sodium-dependent inorganic phosphate cotransporter), member 6(Slc17a6)	Mus musculus
Slc2a1	solute carrier family 2 (facilitated glucose transporter), member 1(Slc2a1)	Mus musculus
Slc2a13	solute carrier family 2 (facilitated glucose transporter), member 13(Slc2a13)	Mus musculus
Slc2a3	solute carrier family 2 (facilitated glucose transporter), member 3(Slc2a3)	Mus musculus
Slc22a23	solute carrier family 22, member 23(Slc22a23)	Mus musculus
Slc25a11	solute carrier family 25 (mitochondrial carrier oxoglutarate carrier), member 11(Slc25a11)	Mus musculus
Slc25a18	solute carrier family 25 (mitochondrial carrier), member 18(Slc25a18)	Mus musculus
Slc25a12	solute carrier family 25 (mitochondrial carrier, Aralar), member 12(Slc25a12)	Mus musculus
Slc25a4	solute carrier family 25 (mitochondrial carrier, adenine nucleotide translocator), member 4(Slc25a4)	Mus musculus
Slc25a5	solute carrier family 25 (mitochondrial carrier, adenine nucleotide translocator), member 5(Slc25a5)	Mus musculus
Slc25a22	solute carrier family 25 (mitochondrial carrier, glutamate), member 22(Slc25a22)	Mus musculus
Slc25a3	solute carrier family 25 (mitochondrial carrier, phosphate carrier), member 3(Slc25a3)	Mus musculus

© 2023. Melachroinou K et al. Published online at http://www.aginganddisease.org/EN/10.14336/AD.2023.0614

Slc3a2	solute carrier family 3 (activators of dibasic and neutral amino acid transport), member 2(Slc3a2)	Mus musculus
Slc30a1	solute carrier family 30 (zinc transporter), member 1(Slc30a1)	Mus musculus
Slc30a3	solute carrier family 30 (zinc transporter), member 3(Slc30a3)	Mus musculus
Slc30a7	solute carrier family 30 (zinc transporter), member 7(Slc30a7)	Mus musculus
Slc32a1	solute carrier family 32 (GABA vesicular transporter), member 1(Slc32a1)	Mus musculus
Slc38a3	solute carrier family 38, member 3(Slc38a3)	Mus musculus
Slc4a4	solute carrier family 4 (anion exchanger), member 4(Slc4a4)	Mus musculus
Slc4a7	solute carrier family 4, sodium bicarbonate cotransporter, member 7(Slc4a7)	Mus musculus
Slc4a10	solute carrier family 4, sodium bicarbonate cotransporter-like, member 10(Slc4a10)	Mus musculus
Slc6a17	solute carrier family 6 (neurotransmitter transporter), member 17(Slc6a17)	Mus musculus
Slc6a1	solute carrier family 6 (neurotransmitter transporter, GABA), member 1(Slc6a1)	Mus musculus
Slc6a11	solute carrier family 6 (neurotransmitter transporter, GABA), member 11(Slc6a11)	Mus musculus
Slc6a7	solute carrier family 6 (neurotransmitter transporter, L-proline), member 7(Slc6a7)	Mus musculus
Slc6a3	solute carrier family 6 (neurotransmitter transporter, dopamine), member 3(Slc6a3)	Mus musculus
Slc6a9	solute carrier family 6 (neurotransmitter transporter, glycine), member 9(Slc6a9)	Mus musculus
Slc7a10	solute carrier family 7 (cationic amino acid transporter, y+ system), member 10(Slc7a10)	Mus musculus
Slc7a11	solute carrier family 7 (cationic amino acid transporter, y+ system), member 11(Slc7a11)	Mus musculus
Slc7a8	solute carrier family 7 (cationic amino acid transporter, y+ system), member 8(Slc7a8)	Mus musculus
Slc8a1	solute carrier family 8 (sodium/calcium exchanger), member 1(Slc8a1)	Mus musculus
Slc8a2	solute carrier family 8 (sodium/calcium exchanger), member 2(Slc8a2)	Mus musculus
Slc9a1	solute carrier family 9 (sodium/hydrogen exchanger), member 1(Slc9a1)	Mus musculus
Slc9a3r1	solute carrier family 9 (sodium/hydrogen exchanger), member 3 regulator 1(Slc9a3r1)	Mus musculus
Sort1	sortilin 1(Sort1)	Mus musculus
Sorcs2	sortilin-related VPS10 domain containing receptor 2(Sorcs2)	Mus musculus
Sorl1	sortilin-related receptor, LDLR class A repeats-containing(Sorl1)	Mus musculus
Spock2	sparc/osteonectin, cwcv and kazal-like domains proteoglycan 2(Spock2)	Mus musculus
Sptan1	spectrin alpha, non-erythrocytic 1(Sptan1)	Mus musculus
Sptbn1	spectrin beta, non-erythrocytic 1(Sptbn1)	Mus musculus
Smpd3	sphingomyelin phosphodiesterase 3, neutral(Smpd3)	Mus musculus
Stmn1	stathmin 1(Stmn1)	Mus musculus
Sdf2	stromal cell derived factor 2(Sdf2)	Mus musculus
Sdf4	stromal cell derived factor 4(Sdf4)	Mus musculus
Sdha	succinate dehydrogenase complex, subunit A, flavoprotein (Fp)(Sdha)	Mus musculus
Sdhb	succinate dehydrogenase complex, subunit B, iron sulfur (Ip)(Sdhb)	Mus musculus
Sdhc	succinate dehydrogenase complex, subunit C, integral membrane protein(Sdhc)	Mus musculus
Suclg1	succinate-CoA ligase, GDP-forming, alpha subunit(Suclg1)	Mus musculus
Sucla2	succinate-Coenzyme A ligase, ADP-forming, beta subunit(Sucla2)	Mus musculus
Sod2	superoxide dismutase 2, mitochondrial(Sod2)	Mus musculus

© 2023. Melachroinou K et al. Published online at http://www.aginganddisease.org/EN/10.14336/AD.2023.0614

SUPPLEMENTARY DATA

Syn1	synapsin I(Syn1)	Mus musculus
Syn2	synapsin II(Syn2)	Mus musculus
Sv2a	synaptic vesicle glycoprotein 2 a(Sv2a)	Mus musculus
Sv2b	synaptic vesicle glycoprotein 2 b(Sv2b)	Mus musculus
Sv2c	synaptic vesicle glycoprotein 2c(Sv2c)	Mus musculus
Syngr1	synaptogyrin 1(Syngr1)	Mus musculus
Syngr3	synaptogyrin 3(Syngr3)	Mus musculus
Synj1	synaptojanin 1(Synj1)	Mus musculus
Syp	synaptophysin(Syp)	Mus musculus
Synpr	synaptoporin(Synpr)	Mus musculus
Snap25	synaptosomal-associated protein 25(Snap25)	Mus musculus
Snap91	synaptosomal-associated protein 91(Snap91)	Mus musculus
Syt1	synaptotagmin I(Syt1)	Mus musculus
Syt2	synaptotagmin II(Syt2)	Mus musculus
Syt3	synaptotagmin III(Syt3)	Mus musculus
Syt5	synaptotagmin V(Syt5)	Mus musculus
Syt6	synaptotagmin VI(Syt6)	Mus musculus
Syt7	synaptotagmin VII(Syt7)	Mus musculus
Syt11	synaptotagmin XI(Syt11)	Mus musculus
Stx1a	syntaxin 1A (brain)(Stx1a)	Mus musculus
Stx1b	syntaxin 1B(Stx1b)	Mus musculus
Stxbp1	syntaxin binding protein 1(Stxbp1)	Mus musculus
Stxbp5	syntaxin binding protein 5 (tomosyn)(Stxbp5)	Mus musculus
Stxbp5l	syntaxin binding protein 5-like(Stxbp5l)	Mus musculus
Snca	synuclein, alpha(Snca)	Mus musculus
Tcp1	t-complex protein 1(Tcp1)	Mus musculus
Tnr	tenascin R(Tnr)	Mus musculus
Tenm4	teneurin transmembrane protein 4(Tenm4)	Mus musculus
Tspan2	tetraspanin 2(Tspan2)	Mus musculus
Tspan7	tetraspanin 7(Tspan7)	Mus musculus
Txndc5	thioredoxin domain containing 5(Txndc5)	Mus musculus
Tmx3	thioredoxin-related transmembrane protein 3(Tmx3)	Mus musculus
Tmx4	thioredoxin-related transmembrane protein 4(Tmx4)	Mus musculus
Taldo1	transaldolase 1(Taldo1)	Mus musculus
Tfrc	transferrin receptor(Tfrc)	Mus musculus
Tagln3	transgelin 3(Tagln3)	Mus musculus
Tkt	transketolase(Tkt)	Mus musculus
Timm13	translocase of inner mitochondrial membrane 13(Timm13)	Mus musculus
Tomm40	translocase of outer mitochondrial membrane 40 homolog (yeast)(Tomm40)	Mus musculus
Tmed2	transmembrane p24 trafficking protein 2(Tmed2)	Mus musculus

© 2023. Melachroinou K et al. Published online at http://www.aginganddisease.org/EN/10.14336/AD.2023.0614

Tmed5	transmembrane p24 trafficking protein 5(Tmed5)	Mus musculus
Tmem109	transmembrane protein 109(Tmem109)	Mus musculus
Tmem33	transmembrane protein 33(Tmem33)	Mus musculus
Tpi1	triosephosphate isomerase 1(Tpi1)	Mus musculus
Wars	tryptophanyl-tRNA synthetase(Wars)	Mus musculus
Tppp	tubulin polymerization promoting protein(Tppp)	Mus musculus
Tuba1a	tubulin, alpha 1A(Tuba1a)	Mus musculus
Tuba1b	tubulin, alpha 1B(Tuba1b)	Mus musculus
Tuba3a	tubulin, alpha 3A(Tuba3a)	Mus musculus
Tuba4a	tubulin, alpha 4A(Tuba4a)	Mus musculus
Tubb2a	tubulin, beta 2A class IIA(Tubb2a)	Mus musculus
Tubb3	tubulin, beta 3 class III(Tubb3)	Mus musculus
Tubb4a	tubulin, beta 4A class IVA(Tubb4a)	Mus musculus
Tubb4b	tubulin, beta 4B class IVB(Tubb4b)	Mus musculus
Tubb5	tubulin, beta 5 class I(Tubb5)	Mus musculus
Tpt1	tumor protein, translationally-controlled 1(Tpt1)	Mus musculus
Ttyh1	tweety family member 1(Ttyh1)	Mus musculus
Ttyh3	tweety family member 3(Ttyh3)	Mus musculus
Ywhab	tyrosine 3-monooxygenase/tryptophan 5-monooxygenase activation protein, beta polypeptide(Ywhab)	Mus musculus
Ywhae	tyrosine 3-monooxygenase/tryptophan 5-monooxygenase activation protein, epsilon polypeptide(Ywhae)	Mus musculus
Ywhah	tyrosine 3-monooxygenase/tryptophan 5-monooxygenase activation protein, eta polypeptide(Ywhah)	Mus musculus
Ywhag	tyrosine 3-monooxygenase/tryptophan 5-monooxygenase activation protein, gamma polypeptide(Ywhag)	Mus musculus
Ywhaq	tyrosine 3-monooxygenase/tryptophan 5-monooxygenase activation protein, theta polypeptide(Ywhaq)	Mus musculus
Ywhaz	tyrosine 3-monooxygenase/tryptophan 5-monooxygenase activation protein, zeta polypeptide(Ywhaz)	Mus musculus
Ubqln2	ubiquilin 2(Ubqln2)	Mus musculus
Uqcrc2	ubiquinol cytochrome c reductase core protein 2(Uqcrc2)	Mus musculus
Uqcrb	ubiquinol-cytochrome c reductase binding protein(Uqcrb)	Mus musculus
Uqcrc1	ubiquinol-cytochrome c reductase core protein 1(Uqcrc1)	Mus musculus
Uqcrh	ubiquinol-cytochrome c reductase hinge protein(Uqcrh)	Mus musculus
Uqcrfs1	ubiquinol-cytochrome c reductase, Rieske iron-sulfur polypeptide 1(Uqcrfs1)	Mus musculus
Uqcrq	ubiquinol-cytochrome c reductase, complex III subunit VII(Uqcrq)	Mus musculus
Uqcr10	ubiquinol-cytochrome c reductase, complex III subunit X(Uqcr10)	Mus musculus
Uba52	ubiquitin A-52 residue ribosomal protein fusion product 1(Uba52)	Mus musculus
Ubb	ubiquitin B(Ubb)	Mus musculus
Ubc	ubiquitin C(Ubc)	Mus musculus
Uchl1	ubiquitin carboxy-terminal hydrolase L1(Uchl1)	Mus musculus
Uchl4	ubiquitin carboxyl-terminal esterase L4(Uchl4)	Mus musculus
Usp14	ubiquitin specific peptidase 14(Usp14)	Mus musculus

© 2023. Melachroinou K et al. Published online at http://www.aginganddisease.org/EN/10.14336/AD.2023.0614

SUPPLEMENTARY DATA

Usp5	ubiquitin specific peptidase 5 (isopeptidase T)(Usp5)	Mus musculus
Ube2n	ubiquitin-conjugating enzyme E2N(Ube2n)	Mus musculus
Uba1	ubiquitin-like modifier activating enzyme 1(Uba1)	Mus musculus
Usmg5	upregulated during skeletal muscle growth 5(Usmg5)	Mus musculus
Rala	v-ral simian leukemia viral oncogene A (ras related)(Rala)	Mus musculus
Vcp	valosin containing protein(Vcp)	Mus musculus
Vcan	versican(Vcan)	Mus musculus
Vamp1	vesicle-associated membrane protein 1(Vamp1)	Mus musculus
Vamp2	vesicle-associated membrane protein 2(Vamp2)	Mus musculus
Vamp3	vesicle-associated membrane protein 3(Vamp3)	Mus musculus
Vsnl1	visinin-like 1(Vsnl1)	Mus musculus
Vdac1	voltage-dependent anion channel 1(Vdac1)	Mus musculus
Vdac2	voltage-dependent anion channel 2(Vdac2)	Mus musculus
Vdac3	voltage-dependent anion channel 3(Vdac3)	Mus musculus
Zmpste24	zinc metallopeptidase, STE24(Zmpste24)	Mus musculus

Supplementary Table 4A. WT-PFF vs WT-PBS, $p<0.05$

T: Protein names	T: Gene names	p-value	N: Student's T-test Difference WT_PFF_WT_PBS
FXYD domain-containing ion transport regulator 6	Fxyd6	9,54E-06	-1,49151
V-type proton ATPase 16 kDa proteolipid subunit	Atp6v0c	0,0187788	-1,36233
Vitamin K epoxide reductase complex subunit 1-like protein 1	Vkorc1l1	0,0371741	-1,27503
Leukocyte surface antigen CD47	Cd47	0,0415083	-1,27032
Calcium/calmodulin-dependent protein kinase type II subunit delta	Camk2d	0,0105429	-1,01205
ES1 protein homolog, mitochondrial	D10Jhu81e	0,0445597	-1,00389
Activated RNA polymerase II transcriptional coactivator p15	Sub1	0,0115368	-0,989217
Prostaglandin E synthase 3	Ptges3	0,014796	-0,94448
Synaptic vesicle glycoprotein 2B	Sv2b	4,01E-05	-0,878362
ER lumen protein-retaining receptor 1	Kdelr1	0,029555	-0,875156
Synaptic vesicle glycoprotein 2C	Sv2c	0,000400792	-0,872033
Synaptophysin	Syp	0,0425388	-0,810893
Cytochrome c oxidase subunit 1	Mtco1	0,0287111	-0,756265
Synaptogyrin-3	Syngr3	0,0193059	-0,74211
Translocon-associated protein subunit alpha	Ssr1	0,00318869	-0,703674
Ras-related protein Rab-35	Rab35	0,0197242	-0,671762
Sodium-dependent proline transporter	Slc6a7	0,0405211	-0,661181

© 2023. Melachroinou K et al. Published online at http://www.aginganddisease.org/EN/10.14336/AD.2023.0614

SUPPLEMENTARY DATA

Transmembrane protein 9B	Tmem9b	0,00516143	-0,643358
Lysophospholipid acyltransferase 7	Mboat7	0,00411008	-0,638856
Tetraspanin-14	Tspan14	0,0439249	-0,631043
Protein-serine O-palmitoleoyltransferase porcupine	Porcn	0,000408832	-0,623231
EGF-like repeat and discoidin I-like domain-containing protein 3	Edil3	0,0162579	-0,602658
CD82 antigen	Cd82	3,88E-05	-0,600763
Synaptic vesicle glycoprotein 2A	Sv2a	3,40E-05	-0,599348
Wolframin	Wfs1	0,0108699	-0,588158
Proton myo-inositol cotransporter	Slc2a13	0,000310516	-0,585425
Vesicular inhibitory amino acid transporter	Slc32a1	0,000181915	-0,578771
Minor histocompatibility antigen H13	Hm13	0,0369369	-0,574092
Calpain-2 catalytic subunit	Capn2	0,0232283	-0,546404
Hyaluronan and proteoglycan link protein 1	Hapln1	0,000185541	-0,541986
Heterogeneous nuclear ribonucleoprotein A1;Heterogeneous nuclear ribonucleoprotein A1, N-terminally processed	Hnrnpa1	0,0327911	-0,533033
Synaptoporin	Synpr	0,00204681	-0,530895
CD81 antigen	Cd81	0,000199642	-0,524076
F-box only protein 2	Fbxo2	1,95E-06	-0,522158
Phosphatidate cytidylyltransferase 2	Cds2	0,00452057	-0,521939
Versican core protein	Vcan	2,17E-05	-0,515589
Peptidyl-glycine alpha-amidating monooxygenase;Peptidylglycine alpha-hydroxylating monooxygenase;Peptidyl-alpha-hydroxyglycine alpha-amidating lyase	Pam	0,013519	-0,509539
Reticulon-3	Rtn3	0,0239601	-0,500438
Vesicular integral-membrane protein VIP36	Lman2	0,000459893	-0,495477
Vesicle-associated membrane protein 2;Vesicle-associated membrane protein 3	Vamp2;Vamp3	0,0014504	-0,482781
Myelin proteolipid protein	Plp1	0,00161421	-0,476665
Testican-2	Spock2	0,000187286	-0,466429
Lysosome-associated membrane glycoprotein 1	Lamp1	0,00185689	-0,455204
Sodium- and chloride-dependent GABA transporter 3	Slc6a11	0,000748835	-0,449916
Vesicle-associated membrane protein 1	Vamp1	0,000558004	-0,437785
Solute carrier family 2, facilitated glucose transporter member 1	Slc2a1	0,0320502	-0,434549
Lipid phosphate phosphohydrolase 3	Ppap2b	0,00200251	-0,43239
Nucleobindin-2;Nesfatin-1	Nucb2	0,0438185	-0,431645
Vesicular glutamate transporter 2	Slc17a6	0,000167767	-0,430577
Protein canopy homolog 2	Cnpy2	0,00200873	-0,43
Choline transporter-like protein 1	Slc44a1	0,000247457	-0,429364
Vesicular glutamate transporter 1	Slc17a7	0,000537312	-0,429029
Receptor-type tyrosine-protein phosphatase eta	Ptprj	0,0357804	-0,418557

© 2023. Melachroinou K et al. Published online at http://www.aginganddisease.org/EN/10.14336/AD.2023.0614

SUPPLEMENTARY DATA

Cell cycle control protein 50A	Tmem30a	0,00181081	-0,416028
Large neutral amino acids transporter small subunit 2	Slc7a8	0,00138121	-0,414866
Protein kinase C beta type	Prkcb	0,00617344	-0,411249
Major prion protein	Prnp	0,0287849	-0,408547
Phospholipid hydroperoxide glutathione peroxidase, mitochondrial	Gpx4	0,000120633	-0,397795
Cathepsin B;Cathepsin B light chain;Cathepsin B heavy chain	Ctsb	0,0272632	-0,393795
Sodium- and chloride-dependent GABA transporter 1	Slc6a1	0,00141876	-0,389411
Leukocyte surface antigen CD47	Cd47	0,0299456	-0,386457
Mammalian ependymin-related protein 1	Epdr1	0,0319671	-0,380023
Choline transporter-like protein 2	Slc44a2	0,00158766	-0,377591
Zinc transporter 7	Slc30a7	0,0380496	-0,373542
1-acyl-sn-glycerol-3-phosphate acyltransferase gamma	Agpat3	0,000166675	-0,372851
RasGAP-activating-like protein 1	Rasal1	0,0147433	-0,365455
Sortilin	Sort1	0,0435849	-0,365001
Calsyntenin-1;Soluble Alc-alpha;CTF1-alpha	Clstn1	0,0139328	-0,357298
Myelin-oligodendrocyte glycoprotein	Mog	0,00449075	-0,354827
NADH-ubiquinone oxidoreductase chain 4	Mtnd4	0,0481246	-0,352002
Protein ERGIC-53	Lman1	0,00613756	-0,351348
Cation-dependent mannose-6-phosphate receptor	M6pr	0,0399084	-0,351323
LMBR1 domain-containing protein 2	Lmbrd2	0,0178484	-0,348872
Sodium/calcium exchanger 1	Slc8a1	0,0120078	-0,347932
Calnexin	Canx	0,00272656	-0,339254
Synaptotagmin-6	Syt6	0,00625574	-0,338711
Adipocyte plasma membrane-associated protein	Apmap	0,0014907	-0,327901
Glycerophosphodiester phosphodiesterase 1	Gde1	8,94E-05	-0,326875
Complement C1q subcomponent subunit B	C1qb	0,0458818	-0,324519
Neurocan core protein	Ncan	0,0259454	-0,323012
Coactosin-like protein	Cotl1	0,0135496	-0,313434
Seizure 6-like protein	Sez6l	0,0059714	-0,312532
Fatty aldehyde dehydrogenase	Aldh3a2	0,000715107	-0,310583
NADH-ubiquinone oxidoreductase chain 5	Mtnd5	0,0480591	-0,307791
Histone deacetylase 11	Hdac11	0,0392313	-0,304612
Reticulon-1	Rtn1	0,0362946	-0,300543
Phosphatidylinositol transfer protein alpha isoform	Pitpna	0,0259323	-0,300199
BDNF/NT-3 growth factors receptor	Ntrk2	0,0116264	-0,298212
Calreticulin	Calr	2,02E-06	-0,297306
Transforming protein RhoA;Rho-related GTP-binding protein RhoC	Rhoa;Rhoc	0,00145806	-0,296901
OX-2 membrane glycoprotein	Cd200	0,0129486	-0,296732

© 2023. Melachroinou K et al. Published online at http://www.aginganddisease.org/EN/10.14336/AD.2023.0614

SUPPLEMENTARY DATA

Receptor-type tyrosine-protein phosphatase zeta	Ptprz1	0,00913895	-0,295259
Electrogenic sodium bicarbonate cotransporter 1	Slc4a4	0,00696435	-0,294361
Metallo-beta-lactamase domain-containing protein 2	Mblac2	0,00256795	-0,289748
Superoxide dismutase [Cu-Zn]	Sod1	0,000958982	-0,28261
Sodium-driven chloride bicarbonate exchanger	Slc4a10	0,0210161	-0,280824
VIP36-like protein	Lman2l	0,0404633	-0,280123
Sodium-dependent neutral amino acid transporter SLC6A17	Slc6a17	0,00634699	-0,279623
LDLR chaperone MESD	Mesdc2	0,000633798	-0,276967
Malectin	Mlec	0,0420262	-0,276619
Neuroplastin	Nptn	0,000833853	-0,273702
Transmembrane anterior posterior transformation protein 1	Tapt1	0,000952583	-0,269638
Serotransferrin	Tf	0,000925889	-0,268287
Translocon-associated protein subunit delta	Ssr4	0,000925914	-0,265836
Neuroligin-3	Nlgn3	0,0115132	-0,257396
Thy-1 membrane glycoprotein	Thy1	1,63E-05	-0,255935
Neuritin	Nrn1	0,0250513	-0,24884
Disintegrin and metalloproteinase domain-containing protein 10	Adam10	0,00704405	-0,231585
Copine-6	Cpne6	0,00206259	-0,224158
Peptidyl-prolyl cis-trans isomerase B	Ppib	0,0152842	-0,221758
VPS10 domain-containing receptor SorCS2	Sorcs2	0,00665485	-0,220219
Isocitrate dehydrogenase [NAD] subunit gamma 1, mitochondrial	Idh3g	0,0264954	-0,219021
	Slc8a2	0,0235718	-0,21891
Annexin A2	Anxa2	0,0309055	-0,217128
Gamma-aminobutyric acid type B receptor subunit 1	Gabbr1	0,043548	-0,206122
Calumenin	Calu	0,0442553	-0,205351
Solute carrier family 15 member 2	Slc15a2	0,0400082	-0,203035
Copine-2	Cpne2	0,0471587	-0,202485
Probable phospholipid-transporting ATPase IIA	Atp9a	0,00107839	-0,197225
DnaJ homolog subfamily C member 5	Dnajc5	0,0357776	-0,18004
Glutamate receptor 2	Gria2	0,00169103	-0,172306
Solute carrier family 12 member 5	Slc12a5	0,0343282	-0,170692
Endoplasmin	Hsp90b1	0,00306544	-0,145062
Prolactin regulatory element-binding protein	Preb	0,0490008	-0,134829
Protein disulfide-isomerase A3	Pdia3	0,0450888	0,127046
Synapsin-1	Syn1	0,00628246	0,152398
Aspartate aminotransferase, mitochondrial	Got2	0,0442888	0,181829
cAMP-dependent protein kinase type II-beta regulatory subunit	Prkar2b	0,0294583	0,19547
Cytoplasmic FMR1-interacting protein 2	Cyfip2	0,0467279	0,196776

© 2023. Melachroinou K et al. Published online at http://www.aginganddisease.org/EN/10.14336/AD.2023.0614

SUPPLEMENTARY DATA

Nck-associated protein 1	Nckap1	0,0492714	0,199383
Glycerophosphodiester phosphodiesterase domain-containing protein 1	Gdpd1	0,0338613	0,20286
Cytosol aminopeptidase	Lap3	0,00191361	0,2115
Plexin-A4	Plxna4	0,0107601	0,2117
Glycogen phosphorylase, brain form	Pygb	0,0254589	0,216621
Glyceraldehyde-3-phosphate dehydrogenase	Gapdh	0,033488	0,221876
Puromycin-sensitive aminopeptidase	Npepps	0,0153863	0,222261
Proteasome subunit alpha type-4	Psma4	0,00481505	0,224378
2-oxoglutarate dehydrogenase, mitochondrial	Ogdh	0,00856472	0,229626
Phosphoglycerate mutase 1	Pgam1	0,0068678	0,243425
AP-2 complex subunit beta	Ap2b1	0,0132486	0,244782
Synaptojanin-1	Synj1	0,0192514	0,2468
cGMP-dependent 3,5-cyclic phosphodiesterase	Pde2a	0,00298158	0,250251
Proteasome subunit beta type-1	Psmb1	0,0196729	0,250533
Rab GDP dissociation inhibitor alpha	Gdi1	0,0105221	0,251526
Transketolase	Tkt	0,024793	0,251665
Aconitate hydratase, mitochondrial	Aco2	0,036009	0,253626
Tripeptidyl-peptidase 2	Tpp2	0,01155	0,253681
Aspartate aminotransferase, cytoplasmic	Got1	0,0307411	0,254863
T-complex protein 1 subunit eta	Cct7	0,00868637	0,255004
Ubiquitin thioesterase OTUB1	Otub1	0,0117815	0,255182
Pyridoxal phosphate phosphatase	Pdxp	0,0221705	0,264516
V-type proton ATPase subunit B, brain isoform	Atp6v1b2	0,0430248	0,264796
Glutamine synthetase	Glul	0,0139078	0,2688
Dihydropyrimidinase-related protein 1	Crmp1	0,0434174	0,272341
Succinate-semialdehyde dehydrogenase, mitochondrial	Aldh5a1	0,0351954	0,276262
Serum albumin	Alb	0,0266473	0,278005
Ankyrin-2	Ank2	0,0310507	0,280763
Neuronal-specific septin-3	septin3	0,0258174	0,283168
Leucine-rich repeat LGI family member 3	Lgi3	0,0440521	0,292939
Ectonucleoside triphosphate diphosphohydrolase 2	Entpd2	0,043532	0,299003
Neurochondrin	Ncdn	0,0363669	0,29937
Plastin-3	Pls3	0,000758252	0,300234
Cytochrome c oxidase subunit 5A, mitochondrial	Cox5a	0,0409579	0,302832
Calcium-dependent secretion activator 1	Cadps	0,00420131	0,30807
Protein phosphatase 1 regulatory subunit 7	Ppp1r7	0,00695303	0,308827
GTP-binding nuclear protein Ran;GTP-binding nuclear protein Ran, testis-specific isoform	Ran;Rasl2-9	0,00314378	0,309265
Transgelin-3	Tagln3	0,0201966	0,310323
Serine/threonine-protein kinase PAK 1	Pak1	0,0143686	0,310904

© 2023. Melachroinou K et al. Published online at http://www.aginganddisease.org/EN/10.14336/AD.2023.0614

T-complex protein 1 subunit alpha	Tcp1	0,00218294	0,312593
Gamma-enolase	Eno2	0,00238598	0,312814
Glucose-6-phosphate isomerase	Gpi	0,00150873	0,327198
LanC-like protein 2	Lancl2	0,0413219	0,338553
Septin-11	Septin 11	0,0037634	0,339436
Stress-70 protein, mitochondrial	Hspa9	0,0189987	0,340007
Receptor-type tyrosine-protein phosphatase alpha	Ptpra	0,0149543	0,345342
Annexin A7	Anxa7	4,33E-07	0,345957
Adenylate kinase isoenzyme 1	Ak1	0,04342	0,350232
L-lactate dehydrogenase B chain	Ldhb	0,0171563	0,351408
Microtubule-associated protein 1A;MAP1A heavy chain;MAP1 light chain LC2	Map1a	0,00378644	0,352457
Sepiapterin reductase	Spr	0,0172131	0,352652
Syntaxin-binding protein 1	Stxbp1	0,000135837	0,354962
Malate dehydrogenase, mitochondrial	Mdh2	0,00934282	0,355384
Heat shock cognate 71 kDa protein	Hspa8	0,00104647	0,368092
Glucose 1,6-bisphosphate synthase	Pgm2l1	0,00782415	0,368603
Adenylyl cyclase-associated protein 2	Cap2	0,000102191	0,371693
Dynamin-1-like protein	Dnm1l	0,00287795	0,372199
Asparagine--tRNA ligase, cytoplasmic	Nars	0,0158695	0,374424
Peripheral plasma membrane protein CASK	Cask	0,00434089	0,376783
Ferritin light chain 1;Ferritin light chain 2	Ftl1;Ftl2	0,00836573	0,377582
Protein NDRG3	Ndrg3	0,00117687	0,382596
Calbindin	Calb1	0,0457353	0,388447
AP-2 complex subunit alpha-1	Ap2a1	0,00611205	0,389236
NAD-dependent protein deacetylase sirtuin-2	Sirt2	0,000327368	0,390787
Clathrin heavy chain 1	Cltc	0,000961608	0,393729
Sideroflexin-1	Sfxn1	0,0427279	0,394024
Fatty acid synthase;[Acyl-carrier-protein] S-acetyltransferase;[Acyl-carrier-protein] S-malonyltransferase;3-oxoacyl-[acyl-carrier-protein] synthase;3-oxoacyl-[acyl-carrier-protein] reductase;3-hydroxyacyl-[acyl-carrier-protein] dehydratase;Enoyl-[acyl-carrier-protein] reductase;Oleoyl-[acyl-carrier-protein] hydrolase	Fasn	0,0275734	0,394264
Actin-related protein 2/3 complex subunit 4	Arpc4	0,0104574	0,39585
Oligodendrocyte-myelin glycoprotein	Omg	0,00309066	0,396282
V-type proton ATPase subunit H	Atp6v1h	0,000170326	0,396813
Spectrin alpha chain, non-erythrocytic 1	Sptan1	0,000751801	0,398356
EF-hand domain-containing protein D2	Efhd2	0,0131318	0,400243
Cullin-associated NEDD8-dissociated protein 1	Cand1	8,31E-06	0,401598
Nucleoside diphosphate kinase A	Nme1	0,000317849	0,404543
Actin-related protein 3	Actr3	0,000499882	0,404721

© 2023. Melachroinou K et al. Published online at http://www.aginganddisease.org/EN/10.14336/AD.2023.0614

SUPPLEMENTARY DATA

Sideroflexin-3	Sfxn3	0,0154109	0,406693
Creatine kinase B-type	Ckb	0,00727274	0,411031
Calcium/calmodulin-dependent 3,5-cyclic nucleotide phosphodiesterase 1B	Pde1b	0,0447582	0,411787
14-3-3 protein gamma;14-3-3 protein gamma, N-terminally processed	Ywhag	0,0324066	0,41542
14-3-3 protein zeta/delta	Ywhaz	0,0151828	0,41671
Protein FAM49B	Fam49b	0,023702	0,417568
Endophilin-A1	Sh3gl2	4,92E-05	0,418188
Adenosylhomocysteinase	Ahcy	0,00198116	0,420219
N(G),N(G)-dimethylarginine dimethylaminohydrolase 1	Ddah1	7,19E-05	0,420595
Glycolipid transfer protein	Gltp	0,0120891	0,425322
Dynamin-1	Dnm1	2,54E-05	0,426923
Isoaspartyl peptidase/L-asparaginase;Isoaspartyl peptidase/L-asparaginase alpha chain;Isoaspartyl peptidase/L-asparaginase beta chain	Asrgl1	0,00637641	0,428198
Heat shock protein 105 kDa	Hsph1	0,000384299	0,430151
Cytoskeleton-associated protein 4	Ckap4	0,0121184	0,431569
Phosphoglucomutase-1	Pgm1	0,00847452	0,439041
ADP/ATP translocase 1	Slc25a4	0,00205245	0,43944
Dynactin subunit 1	Dctn1	0,0440181	0,441881
Dihydropteridine reductase	Qdpr	0,000694842	0,446637
14-3-3 protein epsilon	Ywhae	0,00515894	0,448738
T-complex protein 1 subunit delta	Cct4	0,000780786	0,450316
Beta-soluble NSF attachment protein	Napb	2,63E-05	0,452127
Cytochrome b-c1 complex subunit 7	Uqcrb	0,0169594	0,453009
Oxygen-dependent coproporphyrinogen-III oxidase, mitochondrial	Cpox	0,044947	0,45466
	Pi4ka	0,0219533	0,464752
Transitional endoplasmic reticulum ATPase	Vcp	0,00243508	0,464807
AP-3 complex subunit beta-2	Ap3b2	0,0256224	0,469591
Proteasome subunit alpha type-7	Psma7	0,00191513	0,469615
Citrate synthase, mitochondrial	Cs	2,21E-06	0,471481
Protein-L-isoaspartate(D-aspartate) O-methyltransferase	Pcmt1	0,00459129	0,472147
Calcium-activated potassium channel subunit alpha-1	Kcnma1	0,009918	0,480657
Septin-7	Septin7	8,56E-05	0,481258
ATP-citrate synthase	Acly	0,000246838	0,48161
Aldose reductase	Akr1b1	0,0113542	0,485765
T-complex protein 1 subunit gamma	Cct3	0,000168866	0,48621
Alpha-1-antitrypsin 1-3;Alpha-1-antitrypsin 1-1	Serpina1c;Serpina1a	5,54E-05	0,491736
Myotrophin	Mtpn	0,0177333	0,491884
Protein disulfide-isomerase A6	Pdia6	4,07E-05	0,496739

© 2023. Melachroinou K et al. Published online at http://www.aginganddisease.org/EN/10.14336/AD.2023.0614

SUPPLEMENTARY DATA

Cytoplasmic dynein 1 heavy chain 1	Dync1h1	0,0001423	0,50266
Serine/threonine-protein phosphatase 2A 65 kDa regulatory subunit A alpha isoform	Ppp2r1a	4,13E-06	0,50769
Actin, cytoplasmic 2;Actin, cytoplasmic 2, N-terminally processed	Actg1	3,90E-05	0,50885
Peroxiredoxin-5, mitochondrial	Prdx5	1,51E-05	0,510156
Serine/threonine-protein phosphatase 2A activator	Ppp2r4	0,0119225	0,511246
Coronin-1C	Coro1c	0,0423629	0,514247
ATP synthase subunit d, mitochondrial	Atp5h	0,00139212	0,515256
ATP synthase subunit gamma, mitochondrial	Atp5c1	0,0103822	0,516166
14-3-3 protein eta	Ywhah	0,000779527	0,517593
NADH dehydrogenase [ubiquinone] iron-sulfur protein 3, mitochondrial	Ndufs3	0,0365672	0,520595
Heterogeneous nuclear ribonucleoprotein K	Hnrnpk	0,0064026	0,526251
L-lactate dehydrogenase A chain	Ldha	0,0172894	0,5272
Peroxiredoxin-6	Prdx6	0,00534494	0,532777
NADH dehydrogenase [ubiquinone] 1 beta subcomplex subunit 4	Ndufb4	0,00128975	0,534693
Flotillin-1	Flot1	0,000834587	0,534844
Stress-induced-phosphoprotein 1	Stip1	0,0017079	0,54071
Endoplasmic reticulum resident protein 29	Erp29	7,58E-05	0,545627
Catenin beta-1	Ctnnb1	0,000190861	0,547346
Glyoxalase domain-containing protein 4	Glod4	0,0238438	0,547575
Heat shock 70 kDa protein 4	Hspa4	2,97E-05	0,561926
V-type proton ATPase catalytic subunit A	Atp6v1a	5,24E-06	0,562685
Phosphoglycerate kinase 1	Pgk1	0,000207664	0,570183
Ferritin heavy chain;Ferritin heavy chain, N-terminally processed	Fth1	0,000127678	0,574652
	Rap1gds1	2,38E-05	0,575078
Regulator of G-protein signaling 7	Rgs7	2,02E-05	0,577662
ATP synthase subunit alpha, mitochondrial	Atp5a1	0,00424607	0,582673
Microtubule-associated protein tau	Mapt	1,65E-05	0,584386
Spectrin beta chain, non-erythrocytic 1	Sptbn1	9,08E-08	0,5845
Leucine-rich repeat-containing protein 57	Lrrc57	0,000560997	0,591595
Serine/threonine-protein phosphatase 2B catalytic subunit alpha isoform	Ppp3ca	2,26E-06	0,59394
Carbonyl reductase [NADPH] 1	Cbr1	0,000795306	0,599431
Alpha-synuclein	Snca	0,0196293	0,607903
Alpha-soluble NSF attachment protein	Napa	4,50E-07	0,618211
Hemoglobin subunit beta-1	Hbb-b1	4,79E-05	0,622062
Septin-6	septin6	0,0362116	0,628115
V-type proton ATPase subunit C 1	Atp6v1c1	1,67E-05	0,628165
Retinal dehydrogenase 1;Aldehyde dehydrogenase, cytosolic 1	Aldh1a1;Aldh1a7	0,0130772	0,636948
Adenylosuccinate synthetase isozyme 2	Adss	0,0148927	0,638455

© 2023. Melachroinou K et al. Published online at http://www.aginganddisease.org/EN/10.14336/AD.2023.0614

Phosphoserine aminotransferase	Psat1	0,000318779	0,650101
Gamma-soluble NSF attachment protein	Napg	0,000200101	0,651157
Serine-threonine kinase receptor-associated protein	Strap	0,035619	0,654308
Regulator of G-protein signaling 6	Rgs6	0,0141693	0,654316
Syntaxin-binding protein 5	Stxbp5	0,00147625	0,668075
Costars family protein ABRACL	Abracl	0,0465521	0,687604
Mitochondrial import inner membrane translocase subunit Tim13	Timm13	0,0228607	0,690147
ATP synthase subunit beta, mitochondrial	Atp5b	0,000229986	0,693999
NADH-ubiquinone oxidoreductase 75 kDa subunit, mitochondrial	Ndufs1	0,000750706	0,697835
Protein phosphatase methylesterase 1	Ppme1	0,00277136	0,703677
Protein lin-7 homolog A	Lin7a	5,44E-08	0,70903
Septin-5	Septin7	0,00766679	0,710393
CDP-diacylglycerol--glycerol-3-phosphate 3-phosphatidyltransferase, mitochondrial	Pgs1	0,0206099	0,711467
DnaJ homolog subfamily C member 10	Dnajc10	0,0278923	0,71899
	Ttc7b	0,00255409	0,723217
Heat shock 70 kDa protein 4L	Hspa4l	3,21E-05	0,729302
14-3-3 protein beta/alpha;14-3-3 protein beta/alpha, N-terminally processed	Ywhab	0,00225476	0,730512
Protein deglycase DJ-1	Park7	0,000865152	0,733671
Septin-2	septin2	0,0212672	0,774709
Succinate dehydrogenase [ubiquinone] iron-sulfur subunit, mitochondrial	Sdhb	0,0357843	0,774997
ATP synthase subunit O, mitochondrial	Atp5o	0,00314901	0,797715
Up-regulated during skeletal muscle growth protein 5	Usmg5	0,0397152	0,809934
	Inpp5a	0,0253914	0,811742
Beta-centractin	Actr1b	0,00342579	0,825498
Cyclin-Y	Ccny	0,00121568	0,86048
Brain acid soluble protein 1	Basp1	0,00526843	0,865791
Inorganic pyrophosphatase	Ppa1	0,00916029	0,869925
Alpha-1-antitrypsin 1-5	Serpina1e	3,73E-07	0,876827
Aspartyl aminopeptidase	Dnpep	0,0363321	0,883718
Activator of 90 kDa heat shock protein ATPase homolog 1	Ahsa1	0,0124826	0,949377
Cysteine and glycine-rich protein 1	Csrp1	0,0210094	1,06391
V-type proton ATPase subunit G 2	Atp6v1g2	0,000442645	1,26971
Complexin-2	Cplx2	0,00487043	1,38436
Hemoglobin subunit alpha	Hba	7,31E-06	1,39752
Myc box-dependent-interacting protein 1	Bin1	0,00595215	1,70645
Cytochrome c oxidase subunit 7C, mitochondrial	Cox7c	0,00426945	2,10618

© 2023. Melachroinou K et al. Published online at http://www.aginganddisease.org/EN/10.14336/AD.2023.0614

SUPPLEMENTARY DATA

Supplementary Table 4B. WT-PFF vs WT-PBS, p<0.05,FC>1.2 (log2FC>0.263 or <-0.263).

T: Protein names	T: Gene names	p-value	N: Student's T-test Difference WT_PFF_WT_PBS
FXYD domain-containing ion transport regulator 6	Fxyd6	9,54E-06	-1,49151
V-type proton ATPase 16 kDa proteolipid subunit	Atp6v0c	0,0187788	-1,36233
Vitamin K epoxide reductase complex subunit 1-like protein 1	Vkorc1l1	0,0371741	-1,27503
Leukocyte surface antigen CD47	Cd47	0,0415083	-1,27032
Calcium/calmodulin-dependent protein kinase type II subunit delta	Camk2d	0,0105429	-1,01205
ES1 protein homolog, mitochondrial	D10Jhu81e	0,0445597	-1,00389
Activated RNA polymerase II transcriptional coactivator p15	Sub1	0,0115368	-0,989217
Prostaglandin E synthase 3	Ptges3	0,014796	-0,94448
Synaptic vesicle glycoprotein 2B	Sv2b	4,01E-05	-0,878362
ER lumen protein-retaining receptor 1	Kdelr1	0,029555	-0,875156
Synaptic vesicle glycoprotein 2C	Sv2c	0,000400792	-0,872033
Synaptophysin	Syp	0,0425388	-0,810893
Cytochrome c oxidase subunit 1	Mtco1	0,0287111	-0,756265
Synaptogyrin-3	Syngr3	0,0193059	-0,74211
Translocon-associated protein subunit alpha	Ssr1	0,00318869	-0,703674
Ras-related protein Rab-35	Rab35	0,0197242	-0,671762
Sodium-dependent proline transporter	Slc6a7	0,0405211	-0,661181
Transmembrane protein 9B	Tmem9b	0,00516143	-0,643358
Lysophospholipid acyltransferase 7	Mboat7	0,00411008	-0,638856
Tetraspanin-14	Tspan14	0,0439249	-0,631043
Protein-serine O-palmitoleoyltransferase porcupine	Porcn	0,000408832	-0,623231
EGF-like repeat and discoidin I-like domain-containing protein 3	Edil3	0,0162579	-0,602658
CD82 antigen	Cd82	3,88E-05	-0,600763
Synaptic vesicle glycoprotein 2A	Sv2a	3,40E-05	-0,599348
Wolframin	Wfs1	0,0108699	-0,588158
Proton myo-inositol cotransporter	Slc2a13	0,000310516	-0,585425
Vesicular inhibitory amino acid transporter	Slc32a1	0,000181915	-0,578771
Minor histocompatibility antigen H13	Hm13	0,0369369	-0,574092
Calpain-2 catalytic subunit	Capn2	0,0232283	-0,546404
Hyaluronan and proteoglycan link protein 1	Hapln1	0,000185541	-0,541986
Heterogeneous nuclear ribonucleoprotein A1;Heterogeneous nuclear ribonucleoprotein A1, N-terminally processed	Hnrnpa1	0,0327911	-0,533033
Synaptoporin	Synpr	0,00204681	-0,530895
CD81 antigen	Cd81	0,000199642	-0,524076
F-box only protein 2	Fbxo2	1,95E-06	-0,522158

© 2023. Melachroinou K et al. Published online at http://www.aginganddisease.org/EN/10.14336/AD.2023.0614

SUPPLEMENTARY DATA

Phosphatidate cytidylyltransferase 2	Cds2	0,00452057	-0,521939
Versican core protein	Vcan	2,17E-05	-0,515589
Peptidyl-glycine alpha-amidating monooxygenase;Peptidylglycine alpha-hydroxylating monooxygenase;Peptidyl-alpha-hydroxyglycine alpha-amidating lyase	Pam	0,013519	-0,509539
Reticulon-3	Rtn3	0,0239601	-0,500438
Vesicular integral-membrane protein VIP36	Lman2	0,000459893	-0,495477
Vesicle-associated membrane protein 2;Vesicle-associated membrane protein 3	Vamp2;Vamp3	0,0014504	-0,482781
Myelin proteolipid protein	Plp1	0,00161421	-0,476665
Testican-2	Spock2	0,000187286	-0,466429
Lysosome-associated membrane glycoprotein 1	Lamp1	0,00185689	-0,455204
Sodium- and chloride-dependent GABA transporter 3	Slc6a11	0,000748835	-0,449916
Vesicle-associated membrane protein 1	Vamp1	0,000558004	-0,437785
Solute carrier family 2, facilitated glucose transporter member 1	Slc2a1	0,0320502	-0,434549
Lipid phosphate phosphohydrolase 3	Ppap2b	0,00200251	-0,43239
Nucleobindin-2;Nesfatin-1	Nucb2	0,0438185	-0,431645
Vesicular glutamate transporter 2	Slc17a6	0,000167767	-0,430577
Protein canopy homolog 2	Cnpy2	0,00200873	-0,43
Choline transporter-like protein 1	Slc44a1	0,000247457	-0,429364
Vesicular glutamate transporter 1	Slc17a7	0,000537312	-0,429029
Receptor-type tyrosine-protein phosphatase eta	Ptprj	0,0357804	-0,418557
Cell cycle control protein 50A	Tmem30a	0,00181081	-0,416028
Large neutral amino acids transporter small subunit 2	Slc7a8	0,00138121	-0,414866
Protein kinase C beta type	Prkcb	0,00617344	-0,411249
Major prion protein	Prnp	0,0287849	-0,408547
Phospholipid hydroperoxide glutathione peroxidase, mitochondrial	Gpx4	0,000120633	-0,397795
Cathepsin B;Cathepsin B light chain;Cathepsin B heavy chain	Ctsb	0,0272632	-0,393795
Sodium- and chloride-dependent GABA transporter 1	Slc6a1	0,00141876	-0,389411
Leukocyte surface antigen CD47	Cd47	0,0299456	-0,386457
Mammalian ependymin-related protein 1	Epdr1	0,0319671	-0,380023
Choline transporter-like protein 2	Slc44a2	0,00158766	-0,377591
Zinc transporter 7	Slc30a7	0,0380496	-0,373542
1-acyl-sn-glycerol-3-phosphate acyltransferase gamma	Agpat3	0,000166675	-0,372851
RasGAP-activating-like protein 1	Rasal1	0,0147433	-0,365455
Sortilin	Sort1	0,0435849	-0,365001
Calsyntenin-1;Soluble Alc-alpha;CTF1-alpha	Clstn1	0,0139328	-0,357298
Myelin-oligodendrocyte glycoprotein	Mog	0,00449075	-0,354827
NADH-ubiquinone oxidoreductase chain 4	Mtnd4	0,0481246	-0,352002
Protein ERGIC-53	Lman1	0,00613756	-0,351348

© 2023. Melachroinou K et al. Published online at http://www.aginganddisease.org/EN/10.14336/AD.2023.0614

SUPPLEMENTARY DATA

Cation-dependent mannose-6-phosphate receptor	M6pr	0,0399084	-0,351323
LMBR1 domain-containing protein 2	Lmbrd2	0,0178484	-0,348872
Sodium/calcium exchanger 1	Slc8a1	0,0120078	-0,347932
Calnexin	Canx	0,00272656	-0,339254
Synaptotagmin-6	Syt6	0,00625574	-0,338711
Adipocyte plasma membrane-associated protein	Apmap	0,0014907	-0,327901
Glycerophosphodiester phosphodiesterase 1	Gde1	8,94E-05	-0,326875
Complement C1q subcomponent subunit B	C1qb	0,0458818	-0,324519
Neurocan core protein	Ncan	0,0259454	-0,323012
Coactosin-like protein	Cotl1	0,0135496	-0,313434
Seizure 6-like protein	Sez6l	0,0059714	-0,312532
Fatty aldehyde dehydrogenase	Aldh3a2	0,000715107	-0,310583
NADH-ubiquinone oxidoreductase chain 5	Mtnd5	0,0480591	-0,307791
Histone deacetylase 11	Hdac11	0,0392313	-0,304612
Reticulon-1	Rtn1	0,0362946	-0,300543
Phosphatidylinositol transfer protein alpha isoform	Pitpna	0,0259323	-0,300199
BDNF/NT-3 growth factors receptor	Ntrk2	0,0116264	-0,298212
Calreticulin	Calr	2,02E-06	-0,297306
Transforming protein RhoA;Rho-related GTP-binding protein RhoC	Rhoa;Rhoc	0,00145806	-0,296901
OX-2 membrane glycoprotein	Cd200	0,0129486	-0,296732
Receptor-type tyrosine-protein phosphatase zeta	Ptprz1	0,00913895	-0,295259
Electrogenic sodium bicarbonate cotransporter 1	Slc4a4	0,00696435	-0,294361
Metallo-beta-lactamase domain-containing protein 2	Mblac2	0,00256795	-0,289748
Superoxide dismutase [Cu-Zn]	Sod1	0,000958982	-0,28261
Sodium-driven chloride bicarbonate exchanger	Slc4a10	0,0210161	-0,280824
VIP36-like protein	Lman2l	0,0404633	-0,280123
Sodium-dependent neutral amino acid transporter SLC6A17	Slc6a17	0,00634699	-0,279623
LDLR chaperone MESD	Mesdc2	0,000633798	-0,276967
Malectin	Mlec	0,0420262	-0,276619
Neuroplastin	Nptn	0,000833853	-0,273702
Transmembrane anterior posterior transformation protein 1	Tapt1	0,000952583	-0,269638
Serotransferrin	Tf	0,000925889	-0,268287
Translocon-associated protein subunit delta	Ssr4	0,000925914	-0,265836
Pyridoxal phosphate phosphatase	Pdxp	0,0221705	0,264516
V-type proton ATPase subunit B, brain isoform	Atp6v1b2	0,0430248	0,264796
Glutamine synthetase	Glul	0,0139078	0,2688
Dihydropyrimidinase-related protein 1	Crmp1	0,0434174	0,272341
Succinate-semialdehyde dehydrogenase, mitochondrial	Aldh5a1	0,0351954	0,276262

© 2023. Melachroinou K et al. Published online at http://www.aginganddisease.org/EN/10.14336/AD.2023.0614

SUPPLEMENTARY DATA

Serum albumin	Alb	0,0266473	0,278005
Ankyrin-2	Ank2	0,0310507	0,280763
Neuronal-specific septin-3	septin3	0,0258174	0,283168
Leucine-rich repeat LGI family member 3	Lgi3	0,0440521	0,292939
Ectonucleoside triphosphate diphosphohydrolase 2	Entpd2	0,043532	0,299003
Neurochondrin	Ncdn	0,0363669	0,29937
Plastin-3	Pls3	0,000758252	0,300234
Cytochrome c oxidase subunit 5A, mitochondrial	Cox5a	0,0409579	0,302832
Calcium-dependent secretion activator 1	Cadps	0,00420131	0,30807
Protein phosphatase 1 regulatory subunit 7	Ppp1r7	0,00695303	0,308827
GTP-binding nuclear protein Ran;GTP-binding nuclear protein Ran, testis-specific isoform	Ran;Rasl2-9	0,00314378	0,309265
Transgelin-3	Tagln3	0,0201966	0,310323
Serine/threonine-protein kinase PAK 1	Pak1	0,0143686	0,310904
T-complex protein 1 subunit alpha	Tcp1	0,00218294	0,312593
Gamma-enolase	Eno2	0,00238598	0,312814
Glucose-6-phosphate isomerase	Gpi	0,00150873	0,327198
LanC-like protein 2	Lancl2	0,0413219	0,338553
Septin-11	Septin 11	0,0037634	0,339436
Stress-70 protein, mitochondrial	Hspa9	0,0189987	0,340007
Receptor-type tyrosine-protein phosphatase alpha	Ptpra	0,0149543	0,345342
Annexin A7	Anxa7	4,33E-07	0,345957
Adenylate kinase isoenzyme 1	Ak1	0,04342	0,350232
L-lactate dehydrogenase B chain	Ldhb	0,0171563	0,351408
Microtubule-associated protein 1A;MAP1A heavy chain;MAP1 light chain LC2	Map1a	0,00378644	0,352457
Sepiapterin reductase	Spr	0,0172131	0,352652
Syntaxin-binding protein 1	Stxbp1	0,000135837	0,354962
Malate dehydrogenase, mitochondrial	Mdh2	0,00934282	0,355384
Heat shock cognate 71 kDa protein	Hspa8	0,00104647	0,368092
Glucose 1,6-bisphosphate synthase	Pgm2l1	0,00782415	0,368603
Adenylyl cyclase-associated protein 2	Cap2	0,000102191	0,371693
Dynamin-1-like protein	Dnm1l	0,00287795	0,372199
Asparagine--tRNA ligase, cytoplasmic	Nars	0,0158695	0,374424
Peripheral plasma membrane protein CASK	Cask	0,00434089	0,376783
Ferritin light chain 1;Ferritin light chain 2	Ftl1;Ftl2	0,00836573	0,377582
Protein NDRG3	Ndrg3	0,00117687	0,382596
Calbindin	Calb1	0,0457353	0,388447
AP-2 complex subunit alpha-1	Ap2a1	0,00611205	0,389236
NAD-dependent protein deacetylase sirtuin-2	Sirt2	0,000327368	0,390787
Clathrin heavy chain 1	Cltc	0,000961608	0,393729

© 2023. Melachroinou K et al. Published online at http://www.aginganddisease.org/EN/10.14336/AD.2023.0614

Sideroflexin-1	Sfxn1	0,0427279	0,394024
Fatty acid synthase;[Acyl-carrier-protein] S-acetyltransferase;[Acyl-carrier-protein] S-malonyltransferase;3-oxoacyl-[acyl-carrier-protein] synthase;3-oxoacyl-[acyl-carrier-protein] reductase;3-hydroxyacyl-[acyl-carrier-protein] dehydratase;Enoyl-[acyl-carrier-protein] reductase;Oleoyl-[acyl-carrier-protein] hydrolase	Fasn	0,0275734	0,394264
Actin-related protein 2/3 complex subunit 4	Arpc4	0,0104574	0,39585
Oligodendrocyte-myelin glycoprotein	Omg	0,00309066	0,396282
V-type proton ATPase subunit H	Atp6v1h	0,000170326	0,396813
Spectrin alpha chain, non-erythrocytic 1	Sptan1	0,000751801	0,398356
EF-hand domain-containing protein D2	Efhd2	0,0131318	0,400243
Cullin-associated NEDD8-dissociated protein 1	Cand1	8,31E-06	0,401598
Nucleoside diphosphate kinase A	Nme1	0,000317849	0,404543
Actin-related protein 3	Actr3	0,000499882	0,404721
Sideroflexin-3	Sfxn3	0,0154109	0,406693
Creatine kinase B-type	Ckb	0,00727274	0,411031
Calcium/calmodulin-dependent 3,5-cyclic nucleotide phosphodiesterase 1B	Pde1b	0,0447582	0,411787
14-3-3 protein gamma;14-3-3 protein gamma, N-terminally processed	Ywhag	0,0324066	0,41542
14-3-3 protein zeta/delta	Ywhaz	0,0151828	0,41671
Protein FAM49B	Fam49b	0,023702	0,417568
Endophilin-A1	Sh3gl2	4,92E-05	0,418188
Adenosylhomocysteinase	Ahcy	0,00198116	0,420219
N(G),N(G)-dimethylarginine dimethylaminohydrolase 1	Ddah1	7,19E-05	0,420595
Glycolipid transfer protein	Gltp	0,0120891	0,425322
Dynamin-1	Dnm1	2,54E-05	0,426923
Isoaspartyl peptidase/L-asparaginase;Isoaspartyl peptidase/L-asparaginase alpha chain;Isoaspartyl peptidase/L-asparaginase beta chain	Asrgl1	0,00637641	0,428198
Heat shock protein 105 kDa	Hsph1	0,000384299	0,430151
Cytoskeleton-associated protein 4	Ckap4	0,0121184	0,431569
Phosphoglucomutase-1	Pgm1	0,00847452	0,439041
ADP/ATP translocase 1	Slc25a4	0,00205245	0,43944
Dynactin subunit 1	Dctn1	0,0440181	0,441881
Dihydropteridine reductase	Qdpr	0,000694842	0,446637
14-3-3 protein epsilon	Ywhae	0,00515894	0,448738
T-complex protein 1 subunit delta	Cct4	0,000780786	0,450316
Beta-soluble NSF attachment protein	Napb	2,63E-05	0,452127
Cytochrome b-c1 complex subunit 7	Uqcrb	0,0169594	0,453009
Oxygen-dependent coproporphyrinogen-III oxidase, mitochondrial	Cpox	0,044947	0,45466
	Pi4ka	0,0219533	0,464752

© 2023. Melachroinou K et al. Published online at http://www.aginganddisease.org/EN/10.14336/AD.2023.0614

SUPPLEMENTARY DATA

Transitional endoplasmic reticulum ATPase	Vcp	0,00243508	0,464807
AP-3 complex subunit beta-2	Ap3b2	0,0256224	0,469591
Proteasome subunit alpha type-7	Psma7	0,00191513	0,469615
Citrate synthase, mitochondrial	Cs	2,21E-06	0,471481
Protein-L-isoaspartate(D-aspartate) O-methyltransferase	Pcmt1	0,00459129	0,472147
Calcium-activated potassium channel subunit alpha-1	Kcnma1	0,009918	0,480657
Septin-7	Septin7	8,56E-05	0,481258
ATP-citrate synthase	Acly	0,000246838	0,48161
Aldose reductase	Akr1b1	0,0113542	0,485765
T-complex protein 1 subunit gamma	Cct3	0,000168866	0,48621
Alpha-1-antitrypsin 1-3;Alpha-1-antitrypsin 1-1	Serpina1c;Serpina1a	5,54E-05	0,491736
Myotrophin	Mtpn	0,0177333	0,491884
Protein disulfide-isomerase A6	Pdia6	4,07E-05	0,496739
Cytoplasmic dynein 1 heavy chain 1	Dync1h1	0,0001423	0,50266
Serine/threonine-protein phosphatase 2A 65 kDa regulatory subunit A alpha isoform	Ppp2r1a	4,13E-06	0,50769
Actin, cytoplasmic 2;Actin, cytoplasmic 2, N-terminally processed	Actg1	3,90E-05	0,50885
Peroxiredoxin-5, mitochondrial	Prdx5	1,51E-05	0,510156
Serine/threonine-protein phosphatase 2A activator	Ppp2r4	0,0119225	0,511246
Coronin-1C	Coro1c	0,0423629	0,514247
ATP synthase subunit d, mitochondrial	Atp5h	0,00139212	0,515256
ATP synthase subunit gamma, mitochondrial	Atp5c1	0,0103822	0,516166
14-3-3 protein eta	Ywhah	0,000779527	0,517593
NADH dehydrogenase [ubiquinone] iron-sulfur protein 3, mitochondrial	Ndufs3	0,0365672	0,520595
Heterogeneous nuclear ribonucleoprotein K	Hnrnpk	0,0064026	0,526251
L-lactate dehydrogenase A chain	Ldha	0,0172894	0,5272
Peroxiredoxin-6	Prdx6	0,00534494	0,532777
NADH dehydrogenase [ubiquinone] 1 beta subcomplex subunit 4	Ndufb4	0,00128975	0,534693
Flotillin-1	Flot1	0,000834587	0,534844
Stress-induced-phosphoprotein 1	Stip1	0,0017079	0,54071
Endoplasmic reticulum resident protein 29	Erp29	7,58E-05	0,545627
Catenin beta-1	Ctnnb1	0,000190861	0,547346
Glyoxalase domain-containing protein 4	Glod4	0,0238438	0,547575
Heat shock 70 kDa protein 4	Hspa4	2,97E-05	0,561926
V-type proton ATPase catalytic subunit A	Atp6v1a	5,24E-06	0,562685
Phosphoglycerate kinase 1	Pgk1	0,000207664	0,570183
Ferritin heavy chain;Ferritin heavy chain, N-terminally processed	Fth1	0,000127678	0,574652
	Rap1gds1	2,38E-05	0,575078
Regulator of G-protein signaling 7	Rgs7	2,02E-05	0,577662

© 2023. Melachroinou K et al. Published online at http://www.aginganddisease.org/EN/10.14336/AD.2023.0614

SUPPLEMENTARY DATA

ATP synthase subunit alpha, mitochondrial	Atp5a1	0,00424607	0,582673
Microtubule-associated protein tau	Mapt	1,65E-05	0,584386
Spectrin beta chain, non-erythrocytic 1	Sptbn1	9,08E-08	0,5845
Leucine-rich repeat-containing protein 57	Lrrc57	0,000560997	0,591595
Serine/threonine-protein phosphatase 2B catalytic subunit alpha isoform	Ppp3ca	2,26E-06	0,59394
Carbonyl reductase [NADPH] 1	Cbr1	0,000795306	0,599431
Alpha-synuclein	Snca	0,0196293	0,607903
Alpha-soluble NSF attachment protein	Napa	4,50E-07	0,618211
Hemoglobin subunit beta-1	Hbb-b1	4,79E-05	0,622062
Septin-6	septin6	0,0362116	0,628115
V-type proton ATPase subunit C 1	Atp6v1c1	1,67E-05	0,628165
Retinal dehydrogenase 1;Aldehyde dehydrogenase, cytosolic 1	Aldh1a1;Aldh1a7	0,0130772	0,636948
Adenylosuccinate synthetase isozyme 2	Adss	0,0148927	0,638455
Phosphoserine aminotransferase	Psat1	0,000318779	0,650101
Gamma-soluble NSF attachment protein	Napg	0,000200101	0,651157
Serine-threonine kinase receptor-associated protein	Strap	0,035619	0,654308
Regulator of G-protein signaling 6	Rgs6	0,0141693	0,654316
Syntaxin-binding protein 5	Stxbp5	0,00147625	0,668075
Costars family protein ABRACL	Abracl	0,0465521	0,687604
Mitochondrial import inner membrane translocase subunit Tim13	Timm13	0,0228607	0,690147
ATP synthase subunit beta, mitochondrial	Atp5b	0,000229986	0,693999
NADH-ubiquinone oxidoreductase 75 kDa subunit, mitochondrial	Ndufs1	0,000750706	0,697835
Protein phosphatase methylesterase 1	Ppme1	0,00277136	0,703677
Protein lin-7 homolog A	Lin7a	5,44E-08	0,70903
Septin-5	Septin7	0,00766679	0,710393
CDP-diacylglycerol--glycerol-3-phosphate 3-phosphatidyltransferase, mitochondrial	Pgs1	0,0206099	0,711467
DnaJ homolog subfamily C member 10	Dnajc10	0,0278923	0,71899
	Ttc7b	0,00255409	0,723217
Heat shock 70 kDa protein 4L	Hspa4l	3,21E-05	0,729302
14-3-3 protein beta/alpha;14-3-3 protein beta/alpha, N-terminally processed	Ywhab	0,00225476	0,730512
Protein deglycase DJ-1	Park7	0,000865152	0,733671
Septin-2	septin2	0,0212672	0,774709
Succinate dehydrogenase [ubiquinone] iron-sulfur subunit, mitochondrial	Sdhb	0,0357843	0,774997
ATP synthase subunit O, mitochondrial	Atp5o	0,00314901	0,797715
Up-regulated during skeletal muscle growth protein 5	Usmg5	0,0397152	0,809934
	Inpp5a	0,0253914	0,811742
Beta-centractin	Actr1b	0,00342579	0,825498
Cyclin-Y	Ccny	0,00121568	0,86048

© 2023. Melachroinou K et al. Published online at http://www.aginganddisease.org/EN/10.14336/AD.2023.0614

Brain acid soluble protein 1	Basp1	0,00526843	0,865791
Inorganic pyrophosphatase	Ppa1	0,00916029	0,869925
Alpha-1-antitrypsin 1-5	Serpina1e	3,73E-07	0,876827
Aspartyl aminopeptidase	Dnpep	0,0363321	0,883718
Activator of 90 kDa heat shock protein ATPase homolog 1	Ahsa1	0,0124826	0,949377
Cysteine and glycine-rich protein 1	Csrp1	0,0210094	1,06391
V-type proton ATPase subunit G 2	Atp6v1g2	0,000442645	1,26971
Complexin-2	Cplx2	0,00487043	1,38436
Hemoglobin subunit alpha	Hba	7,31E-06	1,39752
Myc box-dependent-interacting protein 1	Bin1	0,00595215	1,70645
Cytochrome c oxidase subunit 7C, mitochondrial	Cox7c	0,00426945	2,10618

Supplementary Table 5A. KO-PFF vs KO-PBS, p<0.05.

T: Protein names	T: Gene names	p-value	N: Student's T-test Difference KO_PFF_KO_PBS
Acyl-CoA-binding protein	Dbi	0,00817609	-2,09456
Guanine nucleotide-binding protein G(I)/G(S)/G(O) subunit gamma-3	Gng3	0,00244823	-1,91068
Sodium bicarbonate cotransporter 3	Slc4a7	0,0140806	-1,56295
Actin, alpha cardiac muscle 1;Actin, alpha skeletal muscle;Actin, aortic smooth muscle;Actin, gamma-enteric smooth muscle	Actc1;Acta1;Acta2;Actg2	0,0272533	-1,55483
Platelet-activating factor acetylhydrolase IB subunit beta	Pafah1b2	0,0338423	-1,52741
Histidine triad nucleotide-binding protein 1	Hint1	0,0171272	-1,43318
Importin subunit beta-1	Kpnb1	0,00039086	-1,43178
Astrocytic phosphoprotein PEA-15	Pea15	0,0299752	-1,34819
Macrophage migration inhibitory factor	Mif	1,48E-05	-1,16353
GTP-binding protein Rheb	Rheb	0,0108055	-1,10672
Protein-L-isoaspartate(D-aspartate) O-methyltransferase	Pcmt1	3,01E-05	-1,05985
Complexin-2	Cplx2	0,0463788	-1,0514
Inositol polyphosphate 1-phosphatase	Inpp1	0,0018729	-1,03296
Protein NDRG2	Ndrg2	2,26E-05	-1,0303
Coronin-1B	Coro1b	0,00529367	-0,997421
Cofilin-2	Cfl2	0,00099888	-0,951125
ES1 protein homolog, mitochondrial	D10Jhu81e	0,0481777	-0,943491
Protein NDRG3	Ndrg3	0,00012264	-0,942878
Leucine-rich repeat-containing protein 57	Lrrc57	0,00010475	-0,935154
Protein NipSnap homolog 1	Nipsnap1	0,0164158	-0,931698
Ribonuclease UK114	Hrsp12	0,0280827	-0,931002
Serum paraoxonase/arylesterase 2	Pon2	0,0173761	-0,912513

© 2023. Melachroinou K et al. Published online at http://www.aginganddisease.org/EN/10.14336/AD.2023.0614

Sodium-dependent dopamine transporter	Slc6a3	0,0440341	-0,901412
Sodium channel subunit beta-3	Scn3b	0,0449803	-0,879667
Leukotriene A-4 hydrolase	Lta4h	0,0141554	-0,877377
Carbonyl reductase [NADPH] 1	Cbr1	2,33E-06	-0,844957
Neuroendocrine convertase 2	Pcsk2	0,0293817	-0,83938
Cysteine and glycine-rich protein 1	Csrp1	0,0223411	-0,837882
Proteasome subunit alpha type-5	Psma5	0,0362792	-0,836941
Guanine nucleotide-binding protein G(olf) subunit alpha	Gnal	0,0482619	-0,828856
Sepiapterin reductase	Spr	0,00084869	-0,818392
Phosphoserine aminotransferase	Psat1	0,00048323	-0,782838
Cell division control protein 42 homolog	Cdc42	8,78E-05	-0,771018
CD9 antigen	Cd9	0,012934	-0,74967
D-3-phosphoglycerate dehydrogenase	Phgdh	0,00086742	-0,745486
Ras-related protein Rab-14	Rab14	0,00699523	-0,736525
Neurochondrin	Ncdn	0,00017128	-0,727688
Glyceraldehyde-3-phosphate dehydrogenase	Gapdh	0,00155551	-0,718235
Protein deglycase DJ-1	Park7	0,00093718	-0,691818
Fatty acid-binding protein, heart	Fabp3	0,049605	-0,687711
Tubulin beta-3 chain	Tubb3	0,00540736	-0,679958
Major prion protein	Prnp	0,0271513	-0,679542
Myotrophin	Mtpn	0,00295595	-0,677114
Ras-related protein Rab-3C	Rab3c	0,00166913	-0,677108
Cystatin-C	Cst3	0,00096186	-0,675519
Phosphatidylethanolamine-binding protein 1;Hippocampal cholinergic neurostimulating peptide	Pebp1	8,36E-07	-0,67432
GTP-binding nuclear protein Ran;GTP-binding nuclear protein Ran, testis-specific isoform	Ran;Rasl2-9	0,0300006	-0,671871
Brain acid soluble protein 1	Basp1	0,00613399	-0,66224
Disintegrin and metalloproteinase domain-containing protein 11	Adam11	0,0243403	-0,660918
Glyoxylate reductase/hydroxypyruvate reductase	Grhpr	0,00057559	-0,652848
L-lactate dehydrogenase A chain	Ldha	0,00015044	-0,650262
Neuroligin-2	Nlgn2	0,00180754	-0,625692
Syntaxin-binding protein 5-like	Stxbp5l	0,00704924	-0,61817
Glyoxalase domain-containing protein 4	Glod4	0,00013523	-0,613326
Flotillin-2	Flot2	2,26E-06	-0,60818
Serine/threonine-protein phosphatase 5	Ppp5c	0,0236876	-0,606544
Peroxiredoxin-6	Prdx6	6,70E-07	-0,605497
Syntaxin-binding protein 5	Stxbp5	0,00015339	-0,599482
Ras-related protein M-Ras	Mras	0,0159464	-0,598251
Calcium/calmodulin-dependent 3,5-cyclic nucleotide phosphodiesterase 1B	Pde1b	0,0301852	-0,597213
Haloacid dehalogenase-like hydrolase domain-containing protein 2	Hdhd2	0,032443	-0,591879

© 2023. Melachroinou K et al. Published online at http://www.aginganddisease.org/EN/10.14336/AD.2023.0614

SUPPLEMENTARY DATA

Tubulin beta-4B chain	Tubb4b	0,0017794	-0,590352
Tubulin beta-2A chain	Tubb2a	0,015937	-0,584839
Dynamin-1-like protein	Dnm1l	0,00017378	-0,584426
Calcium-activated potassium channel subunit alpha-1	Kcnma1	0,0208398	-0,580834
Septin-5	Σεπ-05	0,0184016	-0,577302
Guanine nucleotide-binding protein G(I)/G(S)/G(T) subunit beta-2	Gnb2	0,0119167	-0,576919
ATP-dependent 6-phosphofructokinase, liver type	Pfkl	0,00194578	-0,576255
Malate dehydrogenase, mitochondrial	Mdh2	0,00013134	-0,57241
Phosphoglycerate kinase 1	Pgk1	1,48E-08	-0,556076
Neuronal cell adhesion molecule	Nrcam	0,00156922	-0,555821
Ketimine reductase mu-crystallin	Crym	0,00019154	-0,555447
V-type proton ATPase subunit d 1	Atp6v0d1	0,0384586	-0,553741
Dihydropyrimidinase-related protein 1	Crmp1	0,0102016	-0,54886
Dual specificity mitogen-activated protein kinase kinase 1	Map2k1	0,028886	-0,544706
Phosphoglucomutase-1	Pgm1	7,72E-06	-0,54276
14-3-3 protein gamma;14-3-3 protein gamma, N-terminally processed	Ywhag	0,0166587	-0,540637
Rho-related GTP-binding protein RhoB	Rhob	0,00583397	-0,537586
Flotillin-1	Flot1	3,29E-05	-0,535885
Solute carrier family 2, facilitated glucose transporter member 3	Slc2a3	0,0217972	-0,534663
Peptidyl-prolyl cis-trans isomerase FKBP1A	Fkbp1a	0,0309809	-0,532517
Septin-6	Σεπ-06	0,0105743	-0,530615
Fructose-bisphosphate aldolase A	Aldoa	3,87E-07	-0,530046
Acetyl-CoA acetyltransferase, mitochondrial	Acat1	0,0002381	-0,527613
Fructose-bisphosphate aldolase C	Aldoc	4,06E-06	-0,526711
Prosaposin receptor GPR37L1	Gpr37l1	0,0313664	-0,523607
Ras-related protein Rab-5A	Rab5a	0,00219528	-0,520837
Ubiquitin carboxyl-terminal hydrolase isozyme L1	Uchl1	0,00035186	-0,510066
Aldose reductase	Akr1b1	0,0244747	-0,507752
AP-2 complex subunit alpha-1	Ap2a1	0,0242061	-0,507577
Neuroligin-3	Nlgn3	0,00796357	-0,505842
Tubulin alpha-4A chain	Tuba4a	0,00395975	-0,505331
Actin, cytoplasmic 2;Actin, cytoplasmic 2, N-terminally processed	Actg1	0,00146253	-0,501407
Lymphocyte antigen 6H	Ly6h	0,0490953	-0,501158
Ras-related C3 botulinum toxin substrate 1;Ras-related C3 botulinum toxin substrate 2	Rac1;Rac2	0,0208637	-0,500521
Ephrin type-A receptor 4	Epha4	5,76E-05	-0,498359
T-complex protein 1 subunit gamma	Cct3	0,00597445	-0,49482
Dihydropteridine reductase	Qdpr	0,0120558	-0,49181
Ras-related protein Rap-2a;Ras-related protein Rap-2c	Rap2a;Rap2c	0,00358804	-0,490649

© 2023. Melachroinou K et al. Published online at http://www.aginganddisease.org/EN/10.14336/AD.2023.0614

cAMP-dependent protein kinase type II-beta regulatory subunit	Prkar2b	0,0395885	-0,489111
4-aminobutyrate aminotransferase, mitochondrial	Abat	0,00011522	-0,488835
Glycerol-3-phosphate dehydrogenase 1-like protein	Gpd1l	0,0336162	-0,488586
Thioredoxin	Txn	0,0167098	-0,476328
Ubiquitin-like modifier-activating enzyme 1	Uba1	0,00024045	-0,47531
T-complex protein 1 subunit delta	Cct4	0,00645813	-0,47463
Gamma-aminobutyric acid receptor subunit beta-1	Gabrb1	0,00665247	-0,47445
Neuronal growth regulator 1	Negr1	0,000211	-0,47021
Elongation factor 1-alpha 2	Eef1a2	0,00478054	-0,467722
Serine/threonine-protein phosphatase PP1-beta catalytic subunit;Serine/threonine-protein phosphatase PP1-alpha catalytic subunit;Serine/threonine-protein phosphatase PP1-gamma catalytic subunit	Ppp1cb;Ppp1ca;Ppp1cc	0,00015993	-0,467268
Ras-related protein Rab-7a	Rab7a	0,00517159	-0,464121
Cofilin-1	Cfl1	0,0477064	-0,463641
Fatty acid synthase;[Acyl-carrier-protein] S-acetyltransferase;[Acyl-carrier-protein] S-malonyltransferase;3-oxoacyl-[acyl-carrier-protein] synthase;3-oxoacyl-[acyl-carrier-protein] reductase;3-hydroxyacyl-[acyl-carrier-protein] dehydratase;Enoyl-[acyl-carrier-protein] reductase;Oleoyl-[acyl-carrier-protein] hydrolase	Fasn	0,0427446	-0,463297
Sortilin	Sort1	0,0147674	-0,45391
Carbonic anhydrase 2	Ca2	0,00043591	-0,452523
V-type proton ATPase subunit B, brain isoform	Atp6v1b2	0,00198056	-0,449637
Peroxiredoxin-5, mitochondrial	Prdx5	0,00131965	-0,446826
Vesicle-fusing ATPase	Nsf	0,00062561	-0,446817
Adenosylhomocysteinase	Ahcy	5,31E-05	-0,443701
Lactoylglutathione lyase	Glo1	0,0135791	-0,443697
Ras-related protein Rab-3A	Rab3a	0,0304531	-0,442746
Isocitrate dehydrogenase [NADP] cytoplasmic	Idh1	0,0095195	-0,440855
Ubiquitin-conjugating enzyme E2 variant 1;Ubiquitin-conjugating enzyme E2 variant 2	Ube2v1;Ube2v2	0,0154793	-0,430401
LanC-like protein 1	Lancl1	0,0125916	-0,429955
Beta-synuclein	Sncb	0,0225067	-0,427728
Nucleoside diphosphate kinase A	Nme1	3,94E-05	-0,427659
Adenylate kinase isoenzyme 1	Ak1	0,0271057	-0,426385
Intercellular adhesion molecule 5	Icam5	0,0286459	-0,426317
Neurotrimin	Ntm	0,00232226	-0,42341
Neurofascin	Nfasc	8,30E-05	-0,423123
Rab GDP dissociation inhibitor alpha	Gdi1	5,03E-07	-0,42267
Coronin-1A	Coro1a	0,0238384	-0,422566
Serine/threonine-protein phosphatase 2B catalytic subunit alpha isoform	Ppp3ca	6,49E-06	-0,418474

© 2023. Melachroinou K et al. Published online at http://www.aginganddisease.org/EN/10.14336/AD.2023.0614

SUPPLEMENTARY DATA

Tubulin alpha-1B chain	Tuba1b	0,0090378	-0,418199
AP-2 complex subunit alpha-2	Ap2a2	0,00012579	-0,415173
Calsyntenin-1;Soluble Alc-alpha;CTF1-alpha	Clstn1	0,0115235	-0,412784
ATP-citrate synthase	Acly	0,00136721	-0,411176
Protein tweety homolog 1	Ttyh1	0,047638	-0,408899
NAD-dependent protein deacetylase sirtuin-2	Sirt2	0,0130025	-0,406427
Glucose 1,6-bisphosphate synthase	Pgm2l1	0,00045203	-0,406136
Dual specificity protein phosphatase 3	Dusp3	0,00749279	-0,403811
Immunoglobulin superfamily member 8	Igsf8	0,0218314	-0,398635
Alcohol dehydrogenase class-3	Adh5	0,0181444	-0,394361
Poly(rC)-binding protein 1	Pcbp1	0,0337833	-0,393804
Citrate synthase, mitochondrial	Cs	0,0268807	-0,385009
ER membrane protein complex subunit 1	Emc1	0,0315282	-0,384979
Isoaspartyl peptidase/L-asparaginase;Isoaspartyl peptidase/L-asparaginase alpha chain;Isoaspartyl peptidase/L-asparaginase beta chain	Asrgl1	0,00425301	-0,383783
Glutamate receptor ionotropic, NMDA 2A	Grin2a	0,0482563	-0,382026
Prolow-density lipoprotein receptor-related protein 1;Low-density lipoprotein receptor-related protein 1 85 kDa subunit;Low-density lipoprotein receptor-related protein 1 515 kDa subunit;Low-density lipoprotein receptor-related protein 1 intracellular domain	Lrp1	5,79E-05	-0,376531
Protein KIAA1045	Kiaa1045	0,00250537	-0,37543
Neural cell adhesion molecule L1	L1cam	0,0140013	-0,374525
Cell adhesion molecule 1	Cadm1	0,00561133	-0,373428
Protein FAM49B	Fam49b	0,00052499	-0,373423
V-type proton ATPase catalytic subunit A	Atp6v1a	0,00011807	-0,371904
Aspartate aminotransferase, cytoplasmic	Got1	0,00253987	-0,370361
Heat shock cognate 71 kDa protein	Hspa8	9,36E-05	-0,368101
Serine/threonine-protein phosphatase 2A catalytic subunit alpha isoform	Ppp2ca	1,66E-05	-0,365635
D-dopachrome decarboxylase	Ddt	0,0156622	-0,363324
Cell adhesion molecule 4	Cadm4	0,00287226	-0,357646
Serine-threonine kinase receptor-associated protein	Strap	0,0298398	-0,35353
Isocitrate dehydrogenase [NAD] subunit alpha, mitochondrial	Idh3a	0,0248359	-0,351014
Coactosin-like protein	Cotl1	0,0143664	-0,350022
Glycogen phosphorylase, brain form	Pygb	0,0200512	-0,348934
Versican core protein	Vcan	0,0080338	-0,345983
Metabotropic glutamate receptor 2	Grm2	0,0221661	-0,341079
Seizure 6-like protein	Sez6l	0,0295732	-0,340528
Guanine nucleotide-binding protein subunit alpha-11	Gna11	0,0001408	-0,340195
Clathrin heavy chain 1	Cltc	0,00056118	-0,335357
Sphingomyelin phosphodiesterase 3	Smpd3	0,00372672	-0,334701

© 2023. Melachroinou K et al. Published online at http://www.aginganddisease.org/EN/10.14336/AD.2023.0614

Cytosolic acyl coenzyme A thioester hydrolase	Acot7	0,0410091	-0,333623
Transitional endoplasmic reticulum ATPase	Vcp	2,53E-05	-0,330465
Adenylyl cyclase-associated protein 2	Cap2	0,00021804	-0,324674
Leucine-rich repeat and immunoglobulin-like domain-containing nogo receptor-interacting protein 1	Lingo1	0,00509864	-0,323042
Aconitate hydratase, mitochondrial	Aco2	0,00363147	-0,317393
AP-2 complex subunit beta	Ap2b1	0,0346899	-0,316829
Ectonucleoside triphosphate diphosphohydrolase 2	Entpd2	0,0308525	-0,313722
L-lactate dehydrogenase B chain	Ldhb	0,00065215	-0,313295
Syntaxin-binding protein 1	Stxbp1	0,00428081	-0,312118
Ubiquitin thioesterase OTUB1	Otub1	0,0408591	-0,311559
Cullin-associated NEDD8-dissociated protein 1	Cand1	0,00184347	-0,310369
Heat shock 70 kDa protein 12A	Hspa12a	0,0405681	-0,299874
Dynamin-1	Dnm1	0,00113804	-0,299489
Actin-related protein 2/3 complex subunit 4	Arpc4	0,0216264	-0,29682
Hematopoietic progenitor cell antigen CD34	Cd34	0,00978274	-0,293865
Synaptic vesicle glycoprotein 2A	Sv2a	0,025674	-0,293646
Transketolase	Tkt	0,0286508	-0,293236
Protein kinase C gamma type	Prkcg	0,0329119	-0,289643
Glutamine synthetase	Glul	0,0194567	-0,285758
Receptor-type tyrosine-protein phosphatase S	Ptprs	0,0135101	-0,281629
Plasma membrane calcium-transporting ATPase 2	Atp2b2	0,0445413	-0,280751
Seizure 6-like protein 2	Sez6l2	0,0343279	-0,280645
Pyridoxal phosphate phosphatase	Pdxp	0,00186561	-0,278858
Triosephosphate isomerase	Tpi1	0,00964891	-0,277064
Peptidyl-prolyl cis-trans isomerase A;Peptidyl-prolyl cis-trans isomerase A, N-terminally processed	Ppia	0,0214661	-0,276348
Microtubule-associated protein 1B;MAP1B heavy chain;MAP1 light chain LC1	Map1b	0,0390513	-0,276253
Tubulin beta-4A chain	Tubb4a	0,0358826	-0,275682
1-phosphatidylinositol 4,5-bisphosphate phosphodiesterase beta-1	Plcb1	0,00200629	-0,275423
S-formylglutathione hydrolase	Esd	0,0402813	-0,273135
Peptidyl-glycine alpha-amidating monooxygenase;Peptidylglycine alpha-hydroxylating monooxygenase;Peptidyl-alpha-hydroxyglycine alpha-amidating lyase	Pam	0,0265435	-0,270907
Contactin-2	Cntn2	7,03E-05	-0,270561
Pyruvate kinase PKM	Pkm	0,00020225	-0,266366
Glycerol-3-phosphate dehydrogenase [NAD(+)], cytoplasmic	Gpd1	0,0389357	-0,26103
Guanine deaminase	Gda	0,0237298	-0,259609
Protein lin-7 homolog A	Lin7a	0,0206621	-0,25809
Synapsin-2	Syn2	0,00153716	-0,257813
Serine/threonine-protein kinase PAK 1	Pak1	0,0447768	-0,256448

© 2023. Melachroinou K et al. Published online at http://www.aginganddisease.org/EN/10.14336/AD.2023.0614

SUPPLEMENTARY DATA

Actin-related protein 3	Actr3	0,0333353	-0,25577
Synaptojanin-1	Synj1	0,0138448	-0,252625
Dihydropyrimidinase-related protein 5	Dpysl5	0,0435425	-0,248357
Complement C1q subcomponent subunit C	C1qc	0,0135942	-0,247862
Cell adhesion molecule 2	Cadm2	0,0110909	-0,246252
10 kDa heat shock protein, mitochondrial	Hspe1	0,0327487	-0,245597
Plasma membrane calcium-transporting ATPase 1	Atp2b1	0,0176576	-0,237075
Limbic system-associated membrane protein	Lsamp	0,0304522	-0,226448
Hepatocyte cell adhesion molecule	Hepacam	0,0480177	-0,223953
Peroxiredoxin-2	Prdx2	0,00070274	-0,214137
Peptidyl-prolyl cis-trans isomerase FKBP2	Fkbp2	0,0368394	-0,211584
Gamma-enolase	Eno2	0,0121139	-0,207732
Alpha-enolase	Eno1	0,0262533	-0,206327
Heat shock 70 kDa protein 4	Hspa4	0,00577777	-0,203736
Disintegrin and metalloproteinase domain-containing protein 22	Adam22	0,0472605	-0,202838
Myelin proteolipid protein	Plp1	0,0498824	-0,192176
Phytanoyl-CoA hydroxylase-interacting protein	Phyhip	0,0192238	-0,187138
Metabotropic glutamate receptor 3	Grm3	0,0253043	-0,186429
cAMP and cAMP-inhibited cGMP 3,5-cyclic phosphodiesterase 10A	Pde10a	0,0378899	-0,180001
Phosphoglycerate mutase 1	Pgam1	0,00984372	-0,175351
Sodium/potassium-transporting ATPase subunit beta-1	Atp1b1	0,0303247	0,125028
AP-3 complex subunit beta-2	Ap3b2	0,0418043	0,190163
Spectrin alpha chain, non-erythrocytic 1	Sptan1	0,0336763	0,206639
Lipid phosphate phosphohydrolase 3	Ppap2b	0,0389911	0,234509
Protein canopy homolog 2	Cnpy2	0,0305676	0,237334
Calreticulin	Calr	0,0112935	0,247385
Glutamate receptor ionotropic, NMDA 1	Grin1	0,00181066	0,261528
Kinesin heavy chain isoform 5C;Kinesin heavy chain isoform 5A	Kif5c;Kif5a	0,0285377	0,273813
Calnexin	Canx	0,046713	0,288438
Glucosidase 2 subunit beta	Prkcsh	0,0311902	0,292059
Guanine nucleotide-binding protein G(q) subunit alpha	Gnaq	0,0281511	0,312723
Glycerophosphodiester phosphodiesterase 1	Gde1	0,0195668	0,313217
Protein disulfide-isomerase A4	Pdia4	0,0150703	0,329101
Phospholipid hydroperoxide glutathione peroxidase, mitochondrial	Gpx4	0,0392214	0,333228
Hsc70-interacting protein	St13	0,0100564	0,333854
Heat shock protein 105 kDa	Hsph1	0,046409	0,334838
Vesicular glutamate transporter 2	Slc17a6	1,14E-05	0,335764
Peroxisomal multifunctional enzyme type 2;(3R)-hydroxyacyl-CoA dehydrogenase;Enoyl-CoA hydratase 2	Hsd17b4	0,0434217	0,336483

SUPPLEMENTARY DATA

Myelin-oligodendrocyte glycoprotein	Mog	0,00361114	0,337285
Solute carrier family 12 member 2	Slc12a2	0,0351091	0,341658
Phospholipid scramblase 3	Plscr3	0,042463	0,351989
Spectrin beta chain, non-erythrocytic 1	Sptbn1	0,0400787	0,354102
Dolichyl-diphosphooligosaccharide--protein glycosyltransferase subunit 1	Rpn1	0,00315349	0,360593
CD82 antigen	Cd82	0,00741263	0,362056
Ferritin light chain 1;Ferritin light chain 2	Ftl1;Ftl2	0,036461	0,375354
Receptor expression-enhancing protein 5	Reep5	0,0328789	0,376331
Charged multivesicular body protein 4b	Chmp4b	0,0482874	0,381227
Transmembrane anterior posterior transformation protein 1	Tapt1	0,0453768	0,383714
Calcium/calmodulin-dependent protein kinase type II subunit beta	Camk2b	0,00094263	0,388403
Alpha-2-macroglobulin receptor-associated protein	Lrpap1	3,65E-05	0,388539
Apolipoprotein E	Apoe	6,43E-05	0,402266
RasGAP-activating-like protein 1	Rasal1	0,0186051	0,429266
Pyridoxal kinase	Pdxk	0,00183165	0,443192
1-acyl-sn-glycerol-3-phosphate acyltransferase gamma	Agpat3	0,01459	0,455274
Reticulon-1	Rtn1	0,00153596	0,464526
Cytochrome c oxidase subunit 5A, mitochondrial	Cox5a	0,0230363	0,51129
Nicastrin	Ncstn	0,0268334	0,523058
Translocon-associated protein subunit alpha	Ssr1	0,0310374	0,539502
Dehydrogenase/reductase SDR family member 7B	Dhrs7b	0,00279116	0,565834
Lactadherin	Mfge8	0,00131446	0,574727
Synaptosomal-associated protein 25	Snap25	0,00553082	0,622547
Vesicle-trafficking protein SEC22b	Sec22b	0,0320022	0,625122
Electron transfer flavoprotein-ubiquinone oxidoreductase, mitochondrial	Etfdh	0,0335154	0,640536
Vesicle-associated membrane protein 1	Vamp1	0,00120657	0,655267
LMBR1 domain-containing protein 2	Lmbrd2	0,00712755	0,66335
Cytochrome c oxidase subunit 4 isoform 1, mitochondrial	Cox4i1	0,0175318	0,671607
Reticulon-4	Rtn4	0,00106566	0,682019
DnaJ homolog subfamily B member 14	Dnajb14	0,0235187	0,6901
NADH-ubiquinone oxidoreductase chain 4	Mtnd4	0,012359	0,693097
Mitochondrial carrier homolog 1	Mtch1	0,018545	0,714011
Lysocardiolipin acyltransferase 1	Lclat1	6,55E-05	0,71987
ATP synthase F(0) complex subunit B1, mitochondrial	Atp5f1	0,00197803	0,722794
Cytochrome c oxidase subunit 6C	Cox6c	0,0441294	0,724306
ATP synthase subunit d, mitochondrial	Atp5h	0,0248417	0,766777
Tropomyosin alpha-3 chain	Tpm3	0,00040458	0,789339
CDGSH iron-sulfur domain-containing protein 1	Cisd1	0,00854357	0,806722

© 2023. Melachroinou K et al. Published online at http://www.aginganddisease.org/EN/10.14336/AD.2023.0614

D-beta-hydroxybutyrate dehydrogenase, mitochondrial	Bdh1	0,00600024	0,81104
Cytochrome b-c1 complex subunit 8	Uqcrq	0,0301921	0,816102
NADH dehydrogenase [ubiquinone] 1 alpha subcomplex subunit 13	Ndufa13	0,0110922	0,816972
NADH dehydrogenase [ubiquinone] 1 alpha subcomplex subunit 2	Ndufa2	0,0193902	0,839355
Reticulon-3	Rtn3	0,00673608	0,846282
Vesicle-associated membrane protein 2;Vesicle-associated membrane protein 3	Vamp2;Vamp3	0,00049857	0,850677
Dynamin-like 120 kDa protein, mitochondrial;Dynamin-like 120 kDa protein, form S1	Opa1	0,0145228	0,899061
Cytochrome b-c1 complex subunit 7	Uqcrb	0,00563258	0,901214
NADH dehydrogenase [ubiquinone] 1 alpha subcomplex subunit 8	Ndufa8	0,0105289	0,910365
Syntenin-1	Sdcbp	0,0204242	0,917818
3-beta-hydroxysteroid-Delta(8),Delta(7)-isomerase	Ebp	0,0453309	0,972259
Signal peptidase complex catalytic subunit SEC11A	Sec11a	0,0253314	1,05461
ATP synthase subunit f, mitochondrial	Atp5j2	0,0394922	1,07322
Serine beta-lactamase-like protein LACTB, mitochondrial	Lactb	0,0125943	1,09783
ATP synthase subunit gamma, mitochondrial	Atp5c1	0,0213714	1,13459
Estradiol 17-beta-dehydrogenase 11	Hsd17b11	0,00978256	1,14358
Vesicle transport through interaction with t-SNAREs homolog 1B	Vti1b	0,0121504	1,22727
Sodium channel subunit beta-4	Scn4b	0,0495827	1,34848
NADH dehydrogenase [ubiquinone] 1 beta subcomplex subunit 3	Ndufb3	0,022548	1,61427
NADH dehydrogenase [ubiquinone] 1 beta subcomplex subunit 5, mitochondrial	Ndufb5	0,0102803	1,71037
Myelin and lymphocyte protein	Mal	0,00019388	1,84202
ADP-ribosylation factor-like protein 6-interacting protein 1	Arl6ip1	0,00188507	2,09409

Supplementary Table 5B. KO-PFF vs KO-PBS, p<0.05, FC>1.2 (log2FC>0.263 or <-0.263).

**KO-PFF vs KO-PBS, p<0.05,FC>1.2 (log2FC>0.263 or <-0.263)**			
T: Protein names	T: Gene names	p-value	N: Student's T-test Difference KO_PFF_KO_PBS
Acyl-CoA-binding protein	Dbi	0,00817609	-2,09456
Guanine nucleotide-binding protein G(I)/G(S)/G(O) subunit gamma-3	Gng3	0,00244823	-1,91068
Sodium bicarbonate cotransporter 3	Slc4a7	0,0140806	-1,56295
Actin, alpha cardiac muscle 1;Actin, alpha skeletal muscle;Actin, aortic smooth muscle;Actin, gamma-enteric smooth muscle	Actc1;Acta1;Acta2;Actg2	0,0272533	-1,55483
Platelet-activating factor acetylhydrolase IB subunit beta	Pafah1b2	0,0338423	-1,52741

© 2023. Melachroinou K et al. Published online at http://www.aginganddisease.org/EN/10.14336/AD.2023.0614

Histidine triad nucleotide-binding protein 1	Hint1	0,0171272	-1,43318
Importin subunit beta-1	Kpnb1	0,00039086	-1,43178
Astrocytic phosphoprotein PEA-15	Pea15	0,0299752	-1,34819
Macrophage migration inhibitory factor	Mif	1,48E-05	-1,16353
GTP-binding protein Rheb	Rheb	0,0108055	-1,10672
Protein-L-isoaspartate(D-aspartate) O-methyltransferase	Pcmt1	3,01E-05	-1,05985
Complexin-2	Cplx2	0,0463788	-1,0514
Inositol polyphosphate 1-phosphatase	Inpp1	0,0018729	-1,03296
Protein NDRG2	Ndrg2	2,26E-05	-1,0303
Coronin-1B	Coro1b	0,00529367	-0,997421
Cofilin-2	Cfl2	0,00099888	-0,951125
ES1 protein homolog, mitochondrial	D10Jhu81e	0,0481777	-0,943491
Protein NDRG3	Ndrg3	0,00012264	-0,942878
Leucine-rich repeat-containing protein 57	Lrrc57	0,00010475	-0,935154
Protein NipSnap homolog 1	Nipsnap1	0,0164158	-0,931698
Ribonuclease UK114	Hrsp12	0,0280827	-0,931002
Serum paraoxonase/arylesterase 2	Pon2	0,0173761	-0,912513
Sodium-dependent dopamine transporter	Slc6a3	0,0440341	-0,901412
Sodium channel subunit beta-3	Scn3b	0,0449803	-0,879667
Leukotriene A-4 hydrolase	Lta4h	0,0141554	-0,877377
Carbonyl reductase [NADPH] 1	Cbr1	2,33E-06	-0,844957
Neuroendocrine convertase 2	Pcsk2	0,0293817	-0,83938
Cysteine and glycine-rich protein 1	Csrp1	0,0223411	-0,837882
Proteasome subunit alpha type-5	Psma5	0,0362792	-0,836941
Guanine nucleotide-binding protein G(olf) subunit alpha	Gnal	0,0482619	-0,828856
Sepiapterin reductase	Spr	0,00084869	-0,818392
Phosphoserine aminotransferase	Psat1	0,00048323	-0,782838
Cell division control protein 42 homolog	Cdc42	8,78E-05	-0,771018
CD9 antigen	Cd9	0,012934	-0,74967
D-3-phosphoglycerate dehydrogenase	Phgdh	0,00086742	-0,745486
Ras-related protein Rab-14	Rab14	0,00699523	-0,736525
Neurochondrin	Ncdn	0,00017128	-0,727688
Glyceraldehyde-3-phosphate dehydrogenase	Gapdh	0,00155551	-0,718235
Protein deglycase DJ-1	Park7	0,00093718	-0,691818
Fatty acid-binding protein, heart	Fabp3	0,049605	-0,687711
Tubulin beta-3 chain	Tubb3	0,00540736	-0,679958
Major prion protein	Prnp	0,0271513	-0,679542
Myotrophin	Mtpn	0,00295595	-0,677114
Ras-related protein Rab-3C	Rab3c	0,00166913	-0,677108
Cystatin-C	Cst3	0,00096186	-0,675519
Phosphatidylethanolamine-binding protein 1;Hippocampal cholinergic neurostimulating peptide	Pebp1	8,36E-07	-0,67432

© 2023. Melachroinou K et al. Published online at http://www.aginganddisease.org/EN/10.14336/AD.2023.0614

GTP-binding nuclear protein Ran;GTP-binding nuclear protein Ran, testis-specific isoform	Ran;Rasl2-9	0,0300006	-0,671871
Brain acid soluble protein 1	Basp1	0,00613399	-0,66224
Disintegrin and metalloproteinase domain-containing protein 11	Adam11	0,0243403	-0,660918
Glyoxylate reductase/hydroxypyruvate reductase	Grhpr	0,00057559	-0,652848
L-lactate dehydrogenase A chain	Ldha	0,00015044	-0,650262
Neuroligin-2	Nlgn2	0,00180754	-0,625692
Syntaxin-binding protein 5-like	Stxbp5l	0,00704924	-0,61817
Glyoxalase domain-containing protein 4	Glod4	0,00013523	-0,613326
Flotillin-2	Flot2	2,26E-06	-0,60818
Serine/threonine-protein phosphatase 5	Ppp5c	0,0236876	-0,606544
Peroxiredoxin-6	Prdx6	6,70E-07	-0,605497
Syntaxin-binding protein 5	Stxbp5	0,00015339	-0,599482
Ras-related protein M-Ras	Mras	0,0159464	-0,598251
Calcium/calmodulin-dependent 3,5-cyclic nucleotide phosphodiesterase 1B	Pde1b	0,0301852	-0,597213
Haloacid dehalogenase-like hydrolase domain-containing protein 2	Hdhd2	0,032443	-0,591879
Tubulin beta-4B chain	Tubb4b	0,0017794	-0,590352
Tubulin beta-2A chain	Tubb2a	0,015937	-0,584839
Dynamin-1-like protein	Dnm1l	0,00017378	-0,584426
Calcium-activated potassium channel subunit alpha-1	Kcnma1	0,0208398	-0,580834
Septin-5	Σεπ-05	0,0184016	-0,577302
Guanine nucleotide-binding protein G(I)/G(S)/G(T) subunit beta-2	Gnb2	0,0119167	-0,576919
ATP-dependent 6-phosphofructokinase, liver type	Pfkl	0,00194578	-0,576255
Malate dehydrogenase, mitochondrial	Mdh2	0,00013134	-0,57241
Phosphoglycerate kinase 1	Pgk1	1,48E-08	-0,556076
Neuronal cell adhesion molecule	Nrcam	0,00156922	-0,555821
Ketimine reductase mu-crystallin	Crym	0,00019154	-0,555447
V-type proton ATPase subunit d 1	Atp6v0d1	0,0384586	-0,553741
Dihydropyrimidinase-related protein 1	Crmp1	0,0102016	-0,54886
Dual specificity mitogen-activated protein kinase kinase 1	Map2k1	0,028886	-0,544706
Phosphoglucomutase-1	Pgm1	7,72E-06	-0,54276
14-3-3 protein gamma;14-3-3 protein gamma, N-terminally processed	Ywhag	0,0166587	-0,540637
Rho-related GTP-binding protein RhoB	Rhob	0,00583397	-0,537586
Flotillin-1	Flot1	3,29E-05	-0,535885
Solute carrier family 2, facilitated glucose transporter member 3	Slc2a3	0,0217972	-0,534663
Peptidyl-prolyl cis-trans isomerase FKBP1A	Fkbp1a	0,0309809	-0,532517
Septin-6	Σεπ-06	0,0105743	-0,530615
Fructose-bisphosphate aldolase A	Aldoa	3,87E-07	-0,530046

© 2023. Melachroinou K et al. Published online at http://www.aginganddisease.org/EN/10.14336/AD.2023.0614

Acetyl-CoA acetyltransferase, mitochondrial	Acat1	0,0002381	-0,527613
Fructose-bisphosphate aldolase C	Aldoc	4,06E-06	-0,526711
Prosaposin receptor GPR37L1	Gpr37l1	0,0313664	-0,523607
Ras-related protein Rab-5A	Rab5a	0,00219528	-0,520837
Ubiquitin carboxyl-terminal hydrolase isozyme L1	Uchl1	0,00035186	-0,510066
Aldose reductase	Akr1b1	0,0244747	-0,507752
AP-2 complex subunit alpha-1	Ap2a1	0,0242061	-0,507577
Neuroligin-3	Nlgn3	0,00796357	-0,505842
Tubulin alpha-4A chain	Tuba4a	0,00395975	-0,505331
Actin, cytoplasmic 2;Actin, cytoplasmic 2, N-terminally processed	Actg1	0,00146253	-0,501407
Lymphocyte antigen 6H	Ly6h	0,0490953	-0,501158
Ras-related C3 botulinum toxin substrate 1;Ras-related C3 botulinum toxin substrate 2	Rac1;Rac2	0,0208637	-0,500521
Ephrin type-A receptor 4	Epha4	5,76E-05	-0,498359
T-complex protein 1 subunit gamma	Cct3	0,00597445	-0,49482
Dihydropteridine reductase	Qdpr	0,0120558	-0,49181
Ras-related protein Rap-2a;Ras-related protein Rap-2c	Rap2a;Rap2c	0,00358804	-0,490649
cAMP-dependent protein kinase type II-beta regulatory subunit	Prkar2b	0,0395885	-0,489111
4-aminobutyrate aminotransferase, mitochondrial	Abat	0,00011522	-0,488835
Glycerol-3-phosphate dehydrogenase 1-like protein	Gpd1l	0,0336162	-0,488586
Thioredoxin	Txn	0,0167098	-0,476328
Ubiquitin-like modifier-activating enzyme 1	Uba1	0,00024045	-0,47531
T-complex protein 1 subunit delta	Cct4	0,00645813	-0,47463
Gamma-aminobutyric acid receptor subunit beta-1	Gabrb1	0,00665247	-0,47445
Neuronal growth regulator 1	Negr1	0,000211	-0,47021
Elongation factor 1-alpha 2	Eef1a2	0,00478054	-0,467722
Serine/threonine-protein phosphatase PP1-beta catalytic subunit;Serine/threonine-protein phosphatase PP1-alpha catalytic subunit;Serine/threonine-protein phosphatase PP1-gamma catalytic subunit	Ppp1cb;Ppp1ca;Ppp1cc	0,00015993	-0,467268
Ras-related protein Rab-7a	Rab7a	0,00517159	-0,464121
Cofilin-1	Cfl1	0,0477064	-0,463641
Fatty acid synthase;[Acyl-carrier-protein] S-acetyltransferase;[Acyl-carrier-protein] S-malonyltransferase;3-oxoacyl-[acyl-carrier-protein] synthase;3-oxoacyl-[acyl-carrier-protein] reductase;3-hydroxyacyl-[acyl-carrier-protein] dehydratase;Enoyl-[acyl-carrier-protein] reductase;Oleoyl-[acyl-carrier-protein] hydrolase	Fasn	0,0427446	-0,463297
Sortilin	Sort1	0,0147674	-0,45391
Carbonic anhydrase 2	Ca2	0,00043591	-0,452523

© 2023. Melachroinou K et al. Published online at http://www.aginganddisease.org/EN/10.14336/AD.2023.0614

SUPPLEMENTARY DATA

V-type proton ATPase subunit B, brain isoform	Atp6v1b2	0,00198056	-0,449637
Peroxiredoxin-5, mitochondrial	Prdx5	0,00131965	-0,446826
Vesicle-fusing ATPase	Nsf	0,00062561	-0,446817
Adenosylhomocysteinase	Ahcy	5,31E-05	-0,443701
Lactoylglutathione lyase	Glo1	0,0135791	-0,443697
Ras-related protein Rab-3A	Rab3a	0,0304531	-0,442746
Isocitrate dehydrogenase [NADP] cytoplasmic	Idh1	0,0095195	-0,440855
Ubiquitin-conjugating enzyme E2 variant 1;Ubiquitin-conjugating enzyme E2 variant 2	Ube2v1;Ube2v2	0,0154793	-0,430401
LanC-like protein 1	Lancl1	0,0125916	-0,429955
Beta-synuclein	Sncb	0,0225067	-0,427728
Nucleoside diphosphate kinase A	Nme1	3,94E-05	-0,427659
Adenylate kinase isoenzyme 1	Ak1	0,0271057	-0,426385
Intercellular adhesion molecule 5	Icam5	0,0286459	-0,426317
Neurotrimin	Ntm	0,00232226	-0,42341
Neurofascin	Nfasc	8,30E-05	-0,423123
Rab GDP dissociation inhibitor alpha	Gdi1	5,03E-07	-0,42267
Coronin-1A	Coro1a	0,0238384	-0,422566
Serine/threonine-protein phosphatase 2B catalytic subunit alpha isoform	Ppp3ca	6,49E-06	-0,418474
Tubulin alpha-1B chain	Tuba1b	0,0090378	-0,418199
AP-2 complex subunit alpha-2	Ap2a2	0,00012579	-0,415173
Calsyntenin-1;Soluble Alc-alpha;CTF1-alpha	Clstn1	0,0115235	-0,412784
ATP-citrate synthase	Acly	0,00136721	-0,411176
Protein tweety homolog 1	Ttyh1	0,047638	-0,408899
NAD-dependent protein deacetylase sirtuin-2	Sirt2	0,0130025	-0,406427
Glucose 1,6-bisphosphate synthase	Pgm2l1	0,00045203	-0,406136
Dual specificity protein phosphatase 3	Dusp3	0,00749279	-0,403811
Immunoglobulin superfamily member 8	Igsf8	0,0218314	-0,398635
Alcohol dehydrogenase class-3	Adh5	0,0181444	-0,394361
Poly(rC)-binding protein 1	Pcbp1	0,0337833	-0,393804
Citrate synthase, mitochondrial	Cs	0,0268807	-0,385009
ER membrane protein complex subunit 1	Emc1	0,0315282	-0,384979
Isoaspartyl peptidase/L-asparaginase;Isoaspartyl peptidase/L-asparaginase alpha chain;Isoaspartyl peptidase/L-asparaginase beta chain	Asrgl1	0,00425301	-0,383783
Glutamate receptor ionotropic, NMDA 2A	Grin2a	0,0482563	-0,382026
Prolow-density lipoprotein receptor-related protein 1;Low-density lipoprotein receptor-related protein 1 85 kDa subunit;Low-density lipoprotein receptor-related protein 1 515 kDa subunit;Low-density lipoprotein receptor-related protein 1 intracellular domain	Lrp1	5,79E-05	-0,376531

© 2023. Melachroinou K et al. Published online at http://www.aginganddisease.org/EN/10.14336/AD.2023.0614

Protein KIAA1045	Kiaa1045	0,00250537	-0,37543
Neural cell adhesion molecule L1	L1cam	0,0140013	-0,374525
Cell adhesion molecule 1	Cadm1	0,00561133	-0,373428
Protein FAM49B	Fam49b	0,00052499	-0,373423
V-type proton ATPase catalytic subunit A	Atp6v1a	0,00011807	-0,371904
Aspartate aminotransferase, cytoplasmic	Got1	0,00253987	-0,370361
Heat shock cognate 71 kDa protein	Hspa8	9,36E-05	-0,368101
Serine/threonine-protein phosphatase 2A catalytic subunit alpha isoform	Ppp2ca	1,66E-05	-0,365635
D-dopachrome decarboxylase	Ddt	0,0156622	-0,363324
Cell adhesion molecule 4	Cadm4	0,00287226	-0,357646
Serine-threonine kinase receptor-associated protein	Strap	0,0298398	-0,35353
Isocitrate dehydrogenase [NAD] subunit alpha, mitochondrial	Idh3a	0,0248359	-0,351014
Coactosin-like protein	Cotl1	0,0143664	-0,350022
Glycogen phosphorylase, brain form	Pygb	0,0200512	-0,348934
Versican core protein	Vcan	0,0080338	-0,345983
Metabotropic glutamate receptor 2	Grm2	0,0221661	-0,341079
Seizure 6-like protein	Sez6l	0,0295732	-0,340528
Guanine nucleotide-binding protein subunit alpha-11	Gna11	0,0001408	-0,340195
Clathrin heavy chain 1	Cltc	0,00056118	-0,335357
Sphingomyelin phosphodiesterase 3	Smpd3	0,00372672	-0,334701
Cytosolic acyl coenzyme A thioester hydrolase	Acot7	0,0410091	-0,333623
Transitional endoplasmic reticulum ATPase	Vcp	2,53E-05	-0,330465
Adenylyl cyclase-associated protein 2	Cap2	0,00021804	-0,324674
Leucine-rich repeat and immunoglobulin-like domain-containing nogo receptor-interacting protein 1	Lingo1	0,00509864	-0,323042
Aconitate hydratase, mitochondrial	Aco2	0,00363147	-0,317393
AP-2 complex subunit beta	Ap2b1	0,0346899	-0,316829
Ectonucleoside triphosphate diphosphohydrolase 2	Entpd2	0,0308525	-0,313722
L-lactate dehydrogenase B chain	Ldhb	0,00065215	-0,313295
Syntaxin-binding protein 1	Stxbp1	0,00428081	-0,312118
Ubiquitin thioesterase OTUB1	Otub1	0,0408591	-0,311559
Cullin-associated NEDD8-dissociated protein 1	Cand1	0,00184347	-0,310369
Heat shock 70 kDa protein 12A	Hspa12a	0,0405681	-0,299874
Dynamin-1	Dnm1	0,00113804	-0,299489
Actin-related protein 2/3 complex subunit 4	Arpc4	0,0216264	-0,29682
Hematopoietic progenitor cell antigen CD34	Cd34	0,00978274	-0,293865
Synaptic vesicle glycoprotein 2A	Sv2a	0,025674	-0,293646
Transketolase	Tkt	0,0286508	-0,293236
Protein kinase C gamma type	Prkcg	0,0329119	-0,289643

© 2023. Melachroinou K et al. Published online at http://www.aginganddisease.org/EN/10.14336/AD.2023.0614

Glutamine synthetase	Glul	0,0194567	-0,285758
Receptor-type tyrosine-protein phosphatase S	Ptprs	0,0135101	-0,281629
Plasma membrane calcium-transporting ATPase 2	Atp2b2	0,0445413	-0,280751
Seizure 6-like protein 2	Sez6l2	0,0343279	-0,280645
Pyridoxal phosphate phosphatase	Pdxp	0,00186561	-0,278858
Triosephosphate isomerase	Tpi1	0,00964891	-0,277064
Peptidyl-prolyl cis-trans isomerase A;Peptidyl-prolyl cis-trans isomerase A, N-terminally processed	Ppia	0,0214661	-0,276348
Microtubule-associated protein 1B;MAP1B heavy chain;MAP1 light chain LC1	Map1b	0,0390513	-0,276253
Tubulin beta-4A chain	Tubb4a	0,0358826	-0,275682
1-phosphatidylinositol 4,5-bisphosphate phosphodiesterase beta-1	Plcb1	0,00200629	-0,275423
S-formylglutathione hydrolase	Esd	0,0402813	-0,273135
Peptidyl-glycine alpha-amidating monooxygenase;Peptidylglycine alpha-hydroxylating monooxygenase;Peptidyl-alpha-hydroxyglycine alpha-amidating lyase	Pam	0,0265435	-0,270907
Contactin-2	Cntn2	7,03E-05	-0,270561
Pyruvate kinase PKM	Pkm	0,00020225	-0,266366
Kinesin heavy chain isoform 5C;Kinesin heavy chain isoform 5A	Kif5c;Kif5a	0,0285377	0,273813
Calnexin	Canx	0,046713	0,288438
Glucosidase 2 subunit beta	Prkcsh	0,0311902	0,292059
Guanine nucleotide-binding protein G(q) subunit alpha	Gnaq	0,0281511	0,312723
Glycerophosphodiester phosphodiesterase 1	Gde1	0,0195668	0,313217
Protein disulfide-isomerase A4	Pdia4	0,0150703	0,329101
Phospholipid hydroperoxide glutathione peroxidase, mitochondrial	Gpx4	0,0392214	0,333228
Hsc70-interacting protein	St13	0,0100564	0,333854
Heat shock protein 105 kDa	Hsph1	0,046409	0,334838
Vesicular glutamate transporter 2	Slc17a6	1,14E-05	0,335764
Peroxisomal multifunctional enzyme type 2;(3R)-hydroxyacyl-CoA dehydrogenase;Enoyl-CoA hydratase 2	Hsd17b4	0,0434217	0,336483
Myelin-oligodendrocyte glycoprotein	Mog	0,00361114	0,337285
Solute carrier family 12 member 2	Slc12a2	0,0351091	0,341658
Phospholipid scramblase 3	Plscr3	0,042463	0,351989
Spectrin beta chain, non-erythrocytic 1	Sptbn1	0,0400787	0,354102
Dolichyl-diphosphooligosaccharide--protein glycosyltransferase subunit 1	Rpn1	0,00315349	0,360593
CD82 antigen	Cd82	0,00741263	0,362056
Ferritin light chain 1;Ferritin light chain 2	Ftl1;Ftl2	0,036461	0,375354
Receptor expression-enhancing protein 5	Reep5	0,0328789	0,376331
Charged multivesicular body protein 4b	Chmp4b	0,0482874	0,381227

© 2023. Melachroinou K et al. Published online at http://www.aginganddisease.org/EN/10.14336/AD.2023.0614

Transmembrane anterior posterior transformation protein 1	Tapt1	0,0453768	0,383714
Calcium/calmodulin-dependent protein kinase type II subunit beta	Camk2b	0,00094263	0,388403
Alpha-2-macroglobulin receptor-associated protein	Lrpap1	3,65E-05	0,388539
Apolipoprotein E	Apoe	6,43E-05	0,402266
RasGAP-activating-like protein 1	Rasal1	0,0186051	0,429266
Pyridoxal kinase	Pdxk	0,00183165	0,443192
1-acyl-sn-glycerol-3-phosphate acyltransferase gamma	Agpat3	0,01459	0,455274
Reticulon-1	Rtn1	0,00153596	0,464526
Cytochrome c oxidase subunit 5A, mitochondrial	Cox5a	0,0230363	0,51129
Nicastrin	Ncstn	0,0268334	0,523058
Translocon-associated protein subunit alpha	Ssr1	0,0310374	0,539502
Dehydrogenase/reductase SDR family member 7B	Dhrs7b	0,00279116	0,565834
Lactadherin	Mfge8	0,00131446	0,574727
Synaptosomal-associated protein 25	Snap25	0,00553082	0,622547
Vesicle-trafficking protein SEC22b	Sec22b	0,0320022	0,625122
Electron transfer flavoprotein-ubiquinone oxidoreductase, mitochondrial	Etfdh	0,0335154	0,640536
Vesicle-associated membrane protein 1	Vamp1	0,00120657	0,655267
LMBR1 domain-containing protein 2	Lmbrd2	0,00712755	0,66335
Cytochrome c oxidase subunit 4 isoform 1, mitochondrial	Cox4i1	0,0175318	0,671607
Reticulon-4	Rtn4	0,00106566	0,682019
DnaJ homolog subfamily B member 14	Dnajb14	0,0235187	0,6901
NADH-ubiquinone oxidoreductase chain 4	Mtnd4	0,012359	0,693097
Mitochondrial carrier homolog 1	Mtch1	0,018545	0,714011
Lysocardiolipin acyltransferase 1	Lclat1	6,55E-05	0,71987
ATP synthase F(0) complex subunit B1, mitochondrial	Atp5f1	0,00197803	0,722794
Cytochrome c oxidase subunit 6C	Cox6c	0,0441294	0,724306
ATP synthase subunit d, mitochondrial	Atp5h	0,0248417	0,766777
Tropomyosin alpha-3 chain	Tpm3	0,00040458	0,789339
CDGSH iron-sulfur domain-containing protein 1	Cisd1	0,00854357	0,806722
D-beta-hydroxybutyrate dehydrogenase, mitochondrial	Bdh1	0,00600024	0,81104
Cytochrome b-c1 complex subunit 8	Uqcrq	0,0301921	0,816102
NADH dehydrogenase [ubiquinone] 1 alpha subcomplex subunit 13	Ndufa13	0,0110922	0,816972
NADH dehydrogenase [ubiquinone] 1 alpha subcomplex subunit 2	Ndufa2	0,0193902	0,839355
Reticulon-3	Rtn3	0,00673608	0,846282
Vesicle-associated membrane protein 2;Vesicle-associated membrane protein 3	Vamp2;Vamp3	0,00049857	0,850677

© 2023. Melachroinou K et al. Published online at http://www.aginganddisease.org/EN/10.14336/AD.2023.0614

Dynamin-like 120 kDa protein, mitochondrial;Dynamin-like 120 kDa protein, form S1	Opa1	0,0145228	0,899061
Cytochrome b-c1 complex subunit 7	Uqcrb	0,00563258	0,901214
NADH dehydrogenase [ubiquinone] 1 alpha subcomplex subunit 8	Ndufa8	0,0105289	0,910365
Syntenin-1	Sdcbp	0,0204242	0,917818
3-beta-hydroxysteroid-Delta(8),Delta(7)-isomerase	Ebp	0,0453309	0,972259
Signal peptidase complex catalytic subunit SEC11A	Sec11a	0,0253314	1,05461
ATP synthase subunit f, mitochondrial	Atp5j2	0,0394922	1,07322
Serine beta-lactamase-like protein LACTB, mitochondrial	Lactb	0,0125943	1,09783
ATP synthase subunit gamma, mitochondrial	Atp5c1	0,0213714	1,13459
Estradiol 17-beta-dehydrogenase 11	Hsd17b11	0,00978256	1,14358
Vesicle transport through interaction with t-SNAREs homolog 1B	Vti1b	0,0121504	1,22727
Sodium channel subunit beta-4	Scn4b	0,0495827	1,34848
NADH dehydrogenase [ubiquinone] 1 beta subcomplex subunit 3	Ndufb3	0,022548	1,61427
NADH dehydrogenase [ubiquinone] 1 beta subcomplex subunit 5, mitochondrial	Ndufb5	0,0102803	1,71037
Myelin and lymphocyte protein	Mal	0,00019388	1,84202
ADP-ribosylation factor-like protein 6-interacting protein 1	Arl6ip1	0,00188507	2,09409

Supplementary Table 6. Antibodies.

Primary Antibodies	Company	Species	Cat. No.	Working Dilution
anti-α-Synuclein C20 (human, mouse, rat)	Santa Cruz, polyclonal	Rabbit	sc-70-11	1:1000 WB
anti-α-Synuclein Syn1 (human, mouse, rat)	BD biosciences, monoclonal	Mouse	610787	1:1000 WB
anti-pS129 α-Synuclein	Abcam, monoclonal	Rabbit	ab51235	1:1000 WB
				1:2000 IHC
anti-pS129 α-Synuclein (Clone 5B9)	Gift from Omar el Agnaf, monoclonal*	Mouse		1:1000 IHC
anti-Flotillin-1	Santa Cruz, monoclonal	Mouse	sc-133153	1:1000 WB
anti-α-Syn conformation-specific MJFR-14-6-4-2	Abcam, monoclonal	Rabbit	ab209538	1:50.000, Dot Blot
				1:2000, IHC
anti-Tyrosine hydroxylase	Merck-Millipore, polyclonal	Rabbit	AB152	1:2000 IHC
anti-Tyrosine hydroxylase	Merck-Millipore, monoclonal	Mouse	MAB318	1:2000 IHC
anti-GFAP	DAKO, polyclonal	Rabbit	Z033401-2	1:750 IHC
anti-Iba-1	Merck-Millipore, monoclonal	Mouse	MABN92	1:500, IHC
anti-PSD-95	Cell Signaling, monoclonal	Rabbit	D27E11	1:500, IHC

© 2023. Melachroinou K et al. Published online at http://www.aginganddisease.org/EN/10.14336/AD.2023.0614

SUPPLEMENTARY DATA

anti-synaptobrevin (VAMP2)	SySy, monoclonal	Mouse	104 211	1:500, IHC
anti-p62 (SQSTM1)	MBL, polyclonal	Rabbit	PM045	1:1000, IHC
anti-Ubiquitin	Abcam, polyclonal	Rabbit	ab7780	1:200, IHC
anti-Neuronal Class III β-Tubulin (TUJ1)	Covance, monoclonal	Mouse	MMS-435P	1:2000 IHC

* https://doi.org/10.1016/j.neulet.2020.134899

**Secondary Antibodies**	**Company**	**Cat. No.**	**Working Dilution**
Alexa Fluor 488 Donkey anti-Mouse	Invitrogen-Thermo Fischer Scientific	A21202	1:2000 IHC
Alexa Fluor 568 Donkey anti-Mouse	Invitrogen-Thermo Fischer Scientific	A10037	1:2000 IHC
Alexa Fuor 488 Goat anti-Rabbit	Invitrogen-Thermo Fischer Scientific	A11034	1:2000 IHC
Alexa Fluor 568 Donkey anti-Rabbit	Invitrogen-Thermo Fischer Scientific	A10042	1:2000 IHC
Goat anti-Mouse HRP	Merck-Millipore	AP124P	1:5000 WB, Dot blot
Goat anti-Rabbit HRP	Merck-Millipore	AP132P	1:5000 WB, Dot blot

© 2023. Melachroinou K et al. Published online at http://www.aginganddisease.org/EN/10.14336/AD.2023.0614
